# Ultrathin nano-layered boron nitride stabilizing perovskite quantum dot-based light-emitting diodes

**DOI:** 10.1038/s41467-026-77166-z

**Published:** 2026-09-01

**Authors:** Jindi Wang, Hongdan Ren, Bo Cai, Mingyang Li, Wenxuan Fan, Leimeng Xu, Jisong Yao, Shalong Wang, Jizhong Song

**Affiliations:** 1 Key Laboratory of Materials Physics of Ministry of Education, Laboratory of Zhongyuan Light, School of Physics, Zhengzhou University, Daxue Road 75, Zhengzhou, 450052 China; 2 State Key Laboratory of Organic Electronics and Information Displays, Nanjing University of Posts & Telecommunications, Nanjing, 210023 China

**Keywords:** Inorganic LEDs, Quantum dots, Inorganic LEDs

## Abstract

Perovskite quantum dot-based light-emitting diodes (QLEDs) have been regarded as promising emitters for lighting and displays. However, the operational stability still greatly hinders their practical applications. Here, we introduce ultrathin nano-layered boron nitride (BN) as buried interfacial layer to enhance device stability. As an effective blocking layer, BN not only interacts with perovskite to passivate interface defects and eliminate ion migration channel, but also directly suppresses ion migration. Meanwhile, BN makes exciton recombination behavior and electrical transport properties of perovskite QDs less sensitive to the heat. Resultantly, BN-optimized QLEDs achieved an average EQE of 30.05% and extrapolated operational lifetime *T*_50_ of 25,263 h at an initial luminance (*L*_0_) of 100 cd m^−2^, which is 1.7-fold and about 100-fold improvement compared to control devices, respectively. In this work, integrating 2D materials into perovskites through interface engineering provides a pragmatic tactic to enhance the performance of halide perovskite-based LEDs.

## Abstract (web summary)

Perovskite quantum dot light-emitting diodes are promising for lighting and displays, but poor operational stability limits their use. Wang et al. added an ultrathin boron nitride interlayer to suppress defects, ion migration, and heat sensitivity, improving efficiency and lifetime.

## Introduction

Solution-processed perovskite quantum dots (QDs) are emerging as promising materials in next-generation light-emitting diodes (LEDs) due to the strong carrier confinement, tunable emission peaks (400–700 nm), and good substrate compatibility^1–3^. Since the first room-temperature perovskite QD-based LED (QLED) was reported, the technology has been developed for over a decade, and significant breakthroughs have been achieved in the EQE of the devices (EQE exceeding 30%)^4–8^. However, the stability of current perovskite-based QLEDs still lags far behind that of OLEDs and traditional QLEDs^9,10^. Although advanced encapsulation techniques can effectively mitigate the influence of external factors such as moisture and oxygen^11^, the intrinsic instability of perovskite QDs under operational stressors of heat and electric field remains one of the obstacles to achieving commercial applications^12^. This is principally because the soft ionic lattice nature and the highly dynamic binding of ligands would produce ionic defects under heat and electrical stresses, driving degradation of the QD films. Importantly, these defects are easily activated by heat and electrical stress, and severe ion migration in the devices under continuous operation^13^. Therefore, suppressing ion migration is crucial for enhancing the long-term stability of QLEDs^14^.

In recent years, substantial research efforts have been devoted to suppressing ion migration. Among them, the interface engineering is crucial to passivate defects and eliminate ion migration pathways^15–18^. For instance, Lee et al. reduced the defect density on the surface of perovskite by bonding the under-coordinated surface lead centers with benzylphosphonic acid (BPA) shell layer^15^. Wang et al. achieved high-performance perovskite LEDs by dual anchoring of lead ions with multifunctional conjugated organic molecules, reducing the defects of perovskites and effectively suppressing the ion migration^19^. These passivators mainly form chemical interactions with perovskites to passivate the negative or positive-charged defects and reduce the migration of cations and anions. Nevertheless, the reported passivators as soft organic molecular ligands can desorb from the surface of perovskite and produce defects during prolonged device operation, which ultimately compromises long-term performance^20,21^.

Relatively speaking, inorganic passivators, especially two-dimensional (2D) graphene-like materials characterized by atoms oriented along a plane, can serve as ideal intermediate layers to prevent ion migration, which has been relatively demonstrated in perovskite solar cells. Notably, 2D nanosheet of materials like molybdenum disulfide (MoS_2_) have been implemented as effective diffusion barriers at perovskite interfaces to prevent ion migration between the perovskite layer and adjacent functional layers^22–25^. Zhou et al. integrated wafer-scale continuous monolayer MoS_2_ buffers at both the top and bottom interfaces of a perovskite layer through a transfer process, which not only provides a physical barrier against ion migration of perovskite into carrier transport layers, but also chemically stabilizes the formamidinium lead iodide phase through strong coordination interaction^26^. However, a critical limitation of these previously used 2D materials lies in relatively narrow bandgap characteristics, which can adversely affect the recombination of confined excitons in the luminescent layer and the realization of high-performance devices.

As a member of 2D materials, boron nitride (BN), also known as white graphene, possesses excellent chemical and thermal properties and can meet the requirements of an ideal intermediate layer candidate. The advantages including (i) dangling free surfaces, which stabilizes the perovskite through interaction; (ii) the ultrathin structure, which minimizes carrier transport loss; (iii) continuous 2D morphology, which maximally blocks ion migration; (iv) good thermal stability, which reduces the sensitivity of the exciton recombination behavior and electrical transport properties of perovskite QDs to heat; (v) large band gap, which is beneficial for the recombination of confined excitons^26–28^.

Therefore, in this work, we conducted a systematic study of BN as a buried interfacial layer to enhance the operational stability of perovskite QLEDs. We propose introducing 2D ultrathin nano-layered BN between the hole transport layer (HTL) and the perovskite QDs. Density functional theory (DFT) and experiment results show that BN could spontaneously adsorb onto QDs and generate strong bonding interactions, stabilize the perovskite structure, reduce defects (the source of ion migration), and achieve a high PLQY of 87%. Moreover, the introduction of a BN ion-blocking layer increases the migration barrier energy and migration activation energy of Br^-^, and the time-of-flight secondary ion mass spectrometry (ToF-SIMS) depth profile analysis intuitively shows that the migration of Br^-^ towards HTL is significantly inhibited. Importantly, the excellent heat dissipation performance of BN reduces the sensitivity of radiative recombination behavior and electrical transport properties of QDs to heat. Resultantly, the champion BN-optimized QLEDs achieved an average EQE of 30.05% and extrapolated operational lifetime *T*_50_ (defined as the time for the luminance to decrease to half of the initial luminance) of 25,263 h at an initial luminance (*L*_0_) of 100 cd m^−2^, which is significantly better than the control QLEDs (EQE = 18.5% and *T*_50_ = 242 h). Our research links perovskite QDs to high-performance 2D materials through interface engineering, offering a more efficient and practical approach to improving perovskite-based LEDs.

## Results

### BN-optimized QLED structure

**Fig. 1:**
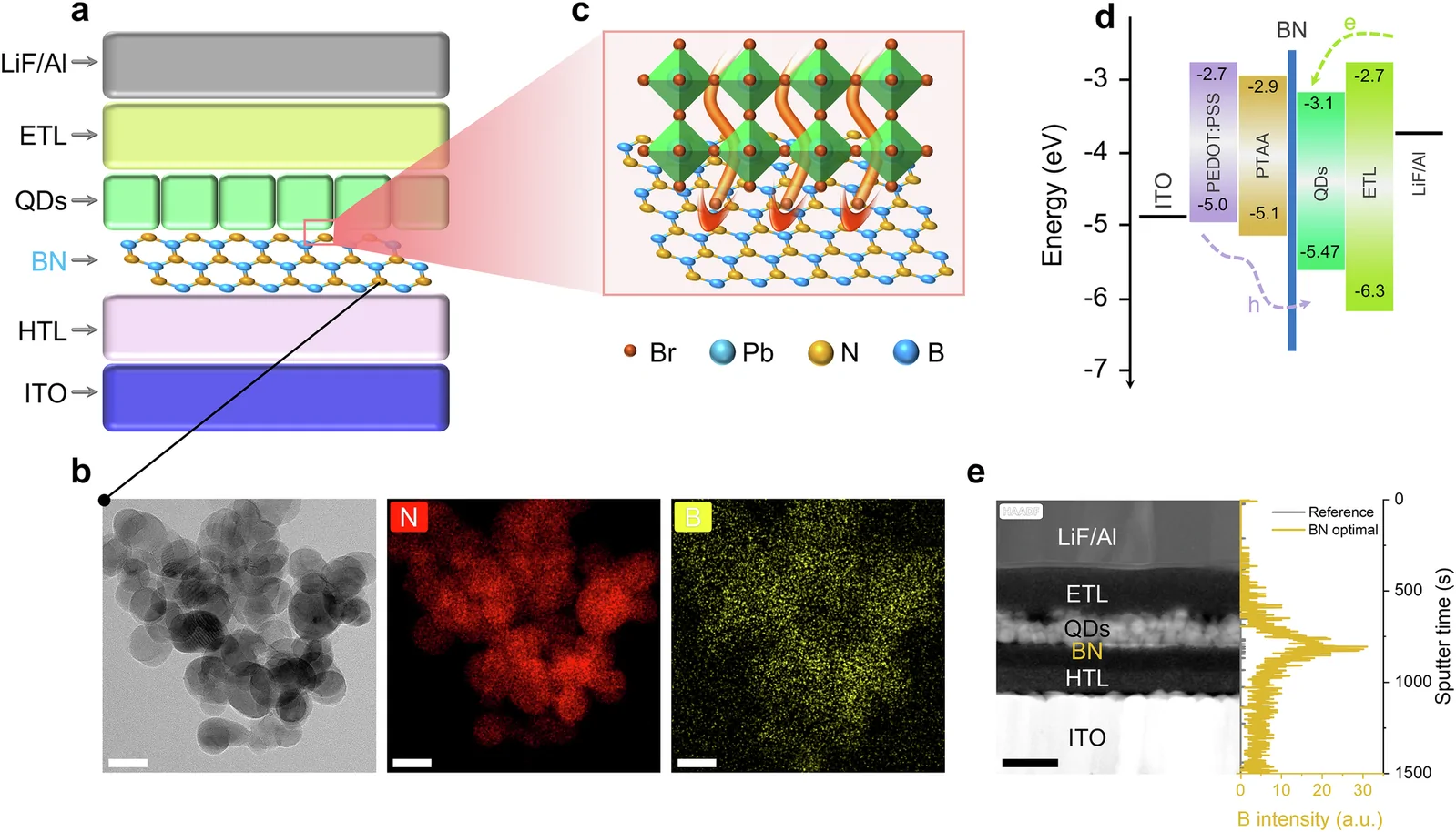
BN-optimized QLEDs structure. **a** The schematic diagram of BN-adsorbed CsPbBr_3_ QLEDs structure. **b** The morphology and EDS spectra of BN. **c** Illustration of BN suppressing ion migration. **d** The energy level diagram of the fabricated QLEDs device. **e** The cross-sectional transmission electron microscopy (TEM) image of the multi-layer CsPbBr_3_ QLEDs structure and the element intensity distribution of B element with sputtering time analyzed by the depth profiles of ToF-SIMS. Scale bars: 50 nm in (**b**) and (**e**).

As shown in Fig. 1a, we fabricated green perovskite QLEDs based on CsPbBr_3_ QDs with a structure of indium tin oxide (ITO)/PEDOT:PSS/PTAA/BN/QDs/TPBi/PO-T2T/LiF/Al to investigate the effect of BN interlayer on the performance of QLED devices. The QDs employed were synthesized according to the previous literature reports with minor modifications^29,30^, as detailed in the experimental section and Supplementary Fig. 1. Nano-layered BN ethanol solution (Supplementary Fig. 2) was spin-coated onto HTL to suppress ion migration. Figure 1b shows BN with a size of about 20 nm, and the elements of B and N are uniformly distributed on the nanosheets. The root mean square roughness of BN films is only ~0.34 nm (Supplementary Fig. 3), serving as an ultra-thin nanolayer to prevent ion migration as presented in Fig. 1c and Supplementary Fig. 4. Importantly, such thin layers will not significantly deteriorate the charge injection performance to worsen the device performance. Furthermore, BN serves as a semiconductor material with a wide bandgap (*E*_g_ of approximately 6.0 eV), which is beneficial for radiative recombination in the QD luminescent layer and improves device performance as illustrated in Fig. 1d and Supplementary Fig. 5. The cross-sectional transmission electron microscopy (TEM) image of the multi-layer CsPbBr_3_ QLED structure and the element intensity distribution of B with sputtering time analyzed by the depth profiles of ToF-SIMS are shown in Fig. 1e. Evidently, there is a significant increase in B content between the HTL and QDs layers. These results indicate successful fabrication of QLEDs containing ultrathin nano-layered BN interlayers.

### Defect passivation of BN on perovskite QDs

**Fig. 2:**
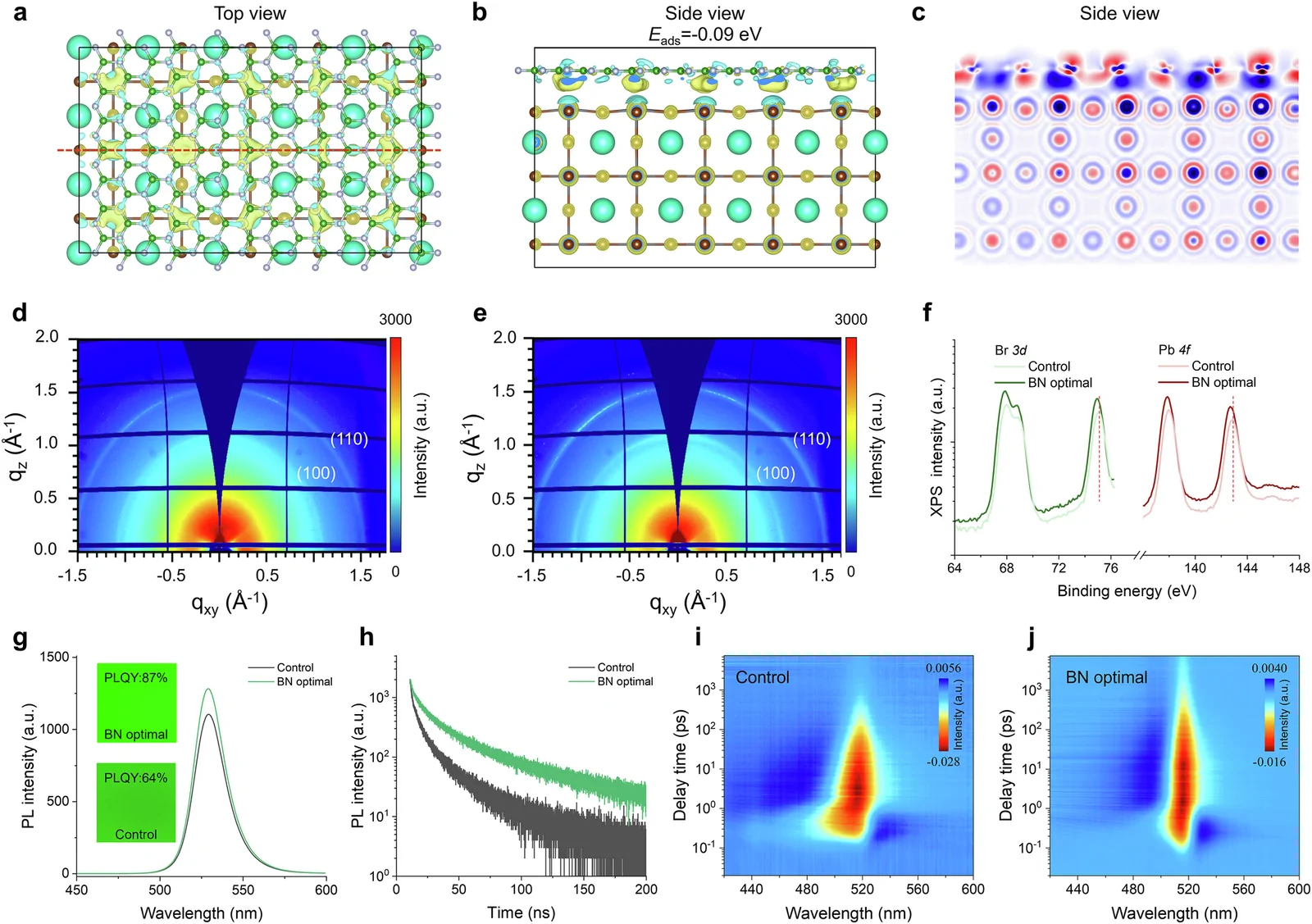
Defect passivation of BN on perovskite QDs. **a**–**c** 3D and 2D slice of charge density differences of PbBr_2_-3 terminated CsPbBr_3_. GIWAXS 2D patterns of the control (**d**) and BN-optimized QD films (**e**). **f** High resolution XPS spectra of Pb 4*f* and Br 3*d* for control and 25 nm etching of BN-optimized QD films. **g** Steady state PL spectra, and photographs (ultraviolet lamp excitation) of control and BN-optimized QD films. **h** TRPL delay curve of control and BN-optimized QD films. Pseudo-color TA spectra and pump-detection delay time for control (**i**) and BN-optimized (**j**) QD films.

In order to explore the effect of BN on perovskite, density functional theory (DFT) calculations were conducted on the CsPbBr_3_ system before and after BN adsorption. From Fig. 2a–c and Supplementary Fig. 6, it can be observed that there is charge redistribution between perovskite surface and BN, and the adsorption energies in different configurations are negative, indicating that the BN spontaneously adsorbs onto the perovskite surfaces. Similar results can be found in CsBr-terminated CsPbBr_3_, as shown in Supplementary Fig. 7. The bonding interaction between BN and the surface of QDs contributes to stabilize the perovskite structure, reduce surface defects, and suppress ion migration^31^. Moreover, the BN-optimized perovskite film shows more pronounced Bragg diffraction rings on the (100) and (110) crystal planes at q_xy_ = 1.1 Å^-1^ and q_xy_ = 1.53 Å^-1^ respectively (Fig. 2d, e and Supplementary Fig. 8). This structural improvement suggests that BN incorporation promotes both crystallinity enhancement and preferential orientation of perovskite crystals^32,33^. Comparative morphological analysis further indicates that BN-optimized QD films exhibit excellent uniformity and compactness, compared with QD films (Supplementary Fig. 9). Moreover, the introduction of a BN layer significantly enhanced the light collection intensity (Supplementary Fig. 10), which indicates that BN can effectively promote light capture, optimize the light output coupling efficiency, and thereby improve the device efficiency^34,35^.

Furthermore, X-ray photoelectron spectroscopy (XPS) and Fourier transform infrared (FTIR) spectroscopy analyses experimentally confirmed the bonding interactions between BN and the surface of QDs. As shown in Fig. 2f, compared to QD films, the characteristic peaks of Pb 4*f* and Br 3*d* in BN-optimized QD films shift to lower binding energies, as well as a higher binding energy shift for the N 1*s* characteristic peak (Supplementary Fig. 11), implicating charge donations from BN to Pb and Br in the perovskite^36,37^. The FTIR spectra of QDs, BN, and BN-optimized QDs provided further details of the bonding interactions between BN and the perovskites (Supplementary Fig. 12). The BN-optimized QD films showed the in-plane B-N stretching mode blue-shifted to 1375 cm^–1^ and an out-of-plane bending mode blue-shifted to 810 cm^-1^, which are different from that of pristine BN (1368 and 801 cm^–1^), indicating that it produces bonding interactions between perovskite and BN^38^. These results indicate that nano-layered BN can interact with QDs, aligning well with the findings from DFT calculations.

Photophysical properties of QDs and BN-optimized QD films were detected to reveal the influence of BN on perovskites. Steady-state PL spectra and spatially resolved PL mapping show that BN-optimized QD films exhibit higher and more uniform PL intensity compared to control samples (Fig. 2g and Supplementary Fig. 13). Meanwhile, the more dazzling green emission under 365 nm UV light excitation and higher absolute PLQY (87%) further indicate that BN improves the radiative recombination of QDs (Fig. 2g inset and Supplementary Fig. 14). In addition, the exciton dynamics are further analyzed using time-resolved PL (TRPL), as shown in Fig. 2h and Supplementary Table 1. The BN-optimized QD films show a PL lifetime of 29 ns, longer than that of 7 ns for the control QD films. The prolonged PL lifetime in BN-optimized QD films indicates enhanced radiative recombination. These results indicate that the introduction of BN could effectively passivate the defect states of QDs and promote radiative recombination.

Meanwhile, the exciton recombination behaviors of QD films were analyzed by pseudocolor transient absorption (TA) spectroscopy as shown in Fig. 2i, j. Obviously, the redshift of the transient bleach minimum to band tail bleaching for BN-optimized QD films is significantly inhibited compared to that observed in the QD samples. This is mainly attributed to the fewer defect states in BN-optimized QD films, which effectively reduce the non-radiative recombination pathways and improves the radiative recombination during the process of release energy to lower energy states when photoexcited carriers release from higher to lower energy states^39^. The defect states are further quantitatively analyzed by space-charge-limited current (SCLC) via a hole-only device (Supplementary Fig. 15)^40,41^. The electron trap density of BN-optimized QD films is 2.38 × 10^18 ^cm^3^, smaller than that of 3.05 × 10^18 ^cm^3^ for QD samples. These results indicate that the introduction of ultrathin nano-layered BN passivates the defects, reduces ion migration channels, and improves the stability and luminescence performance of perovskite QDs.

### BN inhibits ion migration

**Fig. 3:**
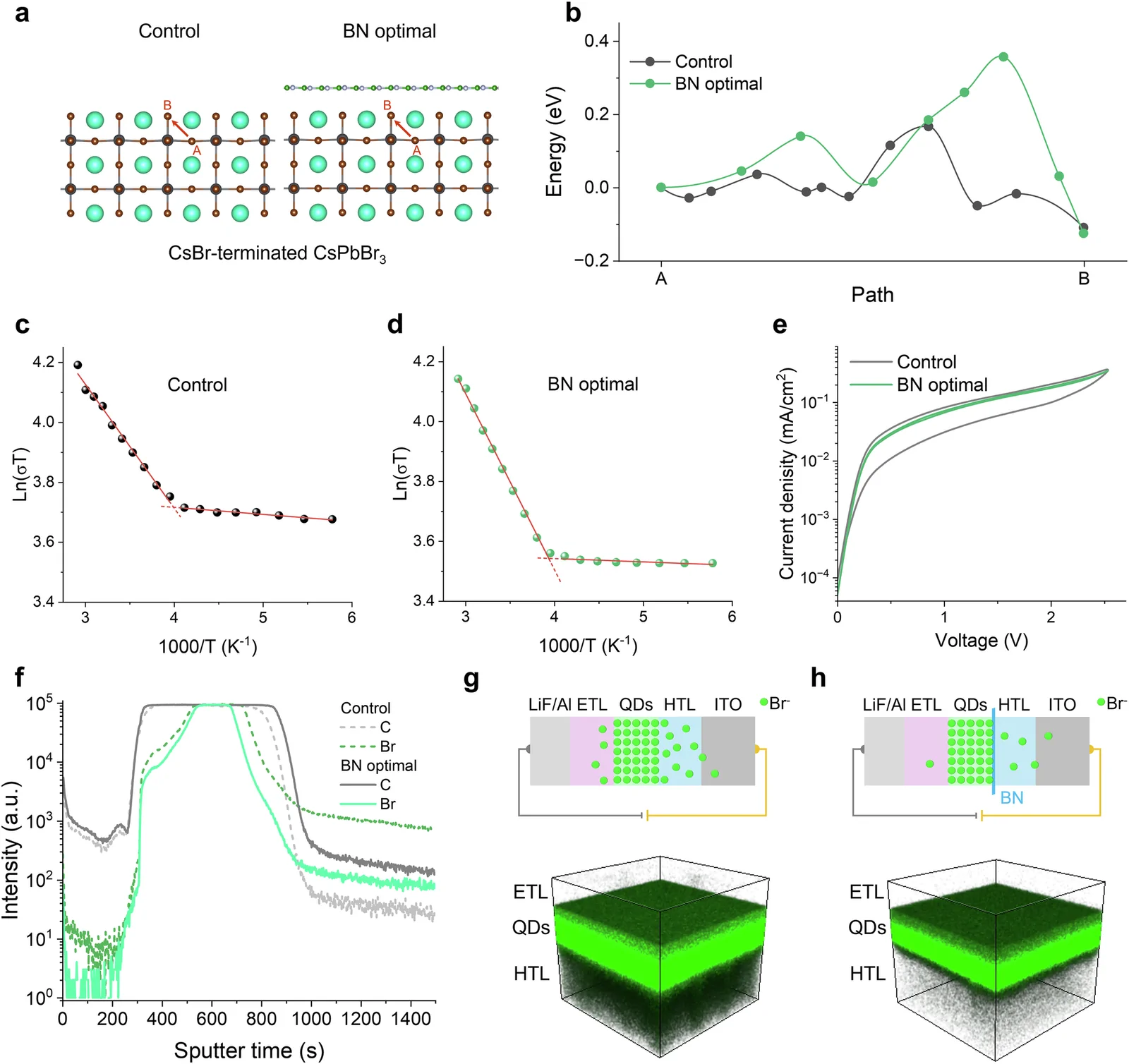
BN inhibits ion migration. The simulation path **a** and potential barriers **b** of CsBr-terminated surface CsPbBr_3_ without/with BN adsorption. Temperature-dependence conductivity σ(T) for control (**c**) and BN-optimized devices (**d**). Filled circles are measured data, while dashed lines are fitted curves. **e** Forward and reverse *J-V* curves of control and BN-optimized devices. **f** The element intensity distribution with sputtering time for ToF-SIMS depth profile of the control and BN-optimized devices. 3D distribution map and migration schematic of Br element in control (**g**) and BN-optimized devices (**h**) by ToF-SIMS depth profile.

BN inhibiting ion migration was first analyzed by the simulation path and potential barriers of CsPbBr_3_ without/with BN as shown in Fig. 3a, b and Supplementary Fig. 16. Figure 3b shows the corresponding energy barriers are ~0.20 eV and ~0.36 eV for perovskite without and with BN, respectively, indicating that BN plays a positive role in suppressing the migration of Br^-^. Furthermore, the influence of BN on the activation energy (*E*_a_) of ion migration was experimentally evaluated by measuring the temperature-dependent conductivity of perovskite films (Supplementary Fig. 17). The *E*_a_ for ion migration can be extracted by a linear fitting of the data based on the Nernst-Einstein equation^42,43^, given by $\sigma \left(T\right)=\frac{{\sigma }_{0}}{T}\exp \left(\frac{-{E}_{a}}{{k}_{B}T}\right)$, where σ(T) is the conductivity as a function of temperature *T*, σ_0_ is a constant and *k*_B_ is the Boltzmann constant. The calculated *E*_a_ value (Fig. 3c, d) for BN-optimized device is 0.58 eV, higher than that of 0.41 eV for the control device, which confirms the migration of charged defects is suppressed by the introduction of BN. Furthermore, the time-dependent PL evolution shows that after a prolonged bias of 200 s, the PL intensity of the ITO/QDs/Al sample rapidly decreased. In contrast, the ITO/BN/QDs/Al sample maintained a stable PL intensity throughout the measurement period (Supplementary Fig. 18). This result further indicates that the introduction of the BN intermediate layer significantly inhibits the ionic migration in the perovskite QD films.

In addition, the electrical stability of BN-optimized QDs also indirectly indicates that BN significantly inhibits ion migration. As shown in Fig. 3e, the control device shows a significant hysteresis under forward and reverse voltage scanning, while the device with BN adsorption exhibits relatively less hysteresis. Meanwhile, the time-current response of applying pulse voltage shown in Supplementary Fig. 19 also demonstrates BN layer can effectively suppress ion migration evidenced by relatively stable current along the increase of time. In addition, the time-dependent change in current under constant bias voltage also supports the suppressed hysteresis results (Supplementary Fig. 20). Under constant bias voltage of 3.0 V, the device with BN optimization maintains a relatively stable current during testing, which was due to the inhibition of ion migration by the ultrathin nano-layered BN, keeping the local electric field at the interface stable^44,45^.

Furthermore, ToF-SIMS was graphically demonstrated to show the inhibition of BN on ions migration (Fig. 3f and Supplementary Fig. 21). From the depth profile of the control and BN-optimized devices (Fig. 3f), it can be observed that compared to the control devices, the signals of Br^-^ on the HTL and ITO layer in the BN-optimized devices are significantly reduced. The corresponding 3D images for Al, C, B, Br, and In element distribution shown in Supplementary Fig. 22. Notably, the corresponding 3D images for Br^-^ distribution further validates that there is very slight downward diffusion of Br^-^ in BN-optimized devices, indicating effective suppression of negatively charged Br^-^ migration towards the HTL sides under external electric field (Fig. 3g and h).

### BN enhances the thermal stability

**Fig. 4:**
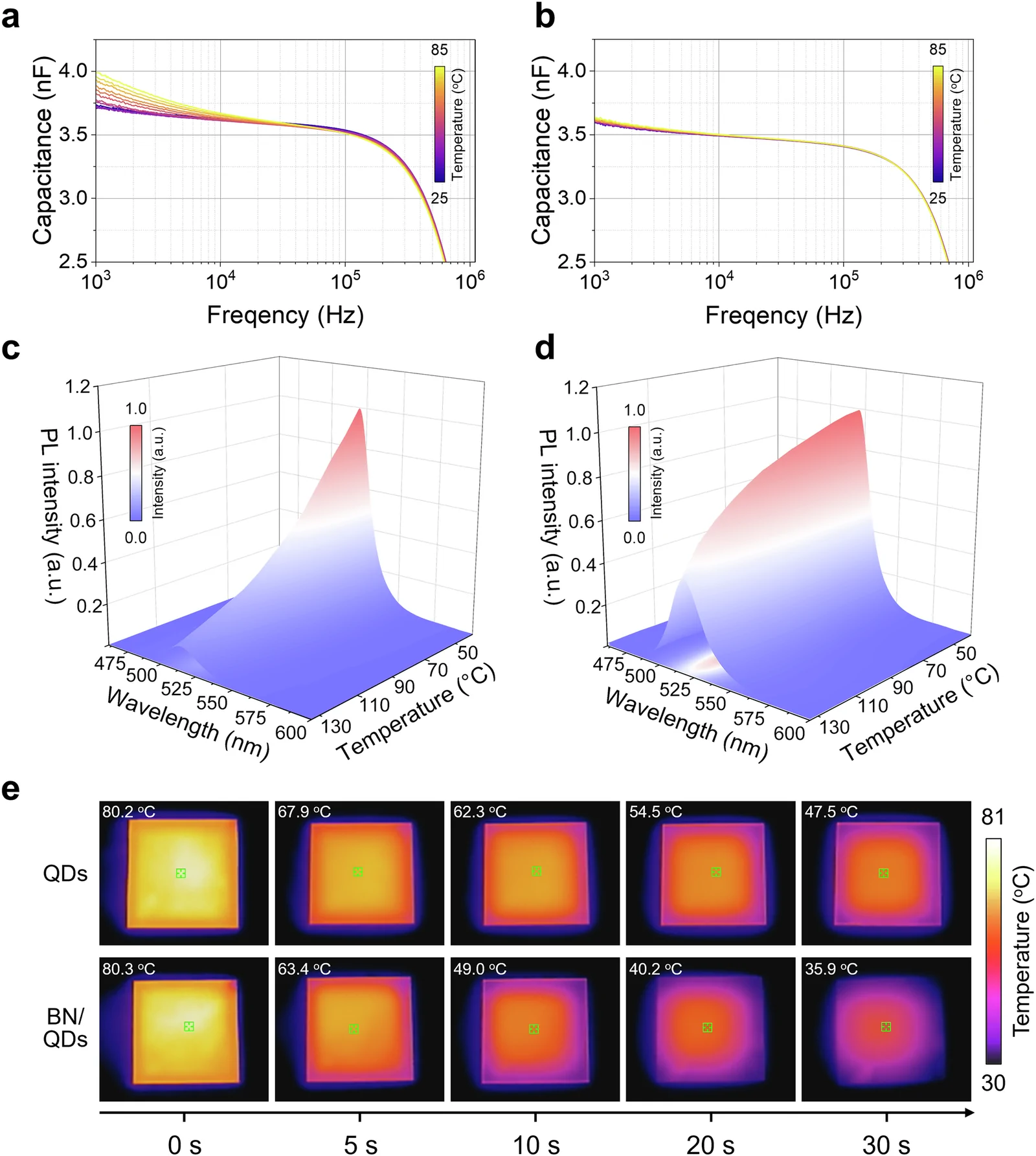
BN enhancing the thermal stability. Frequency-dependent capacitance plot of control (**a**) and BN-optimized devices (**b**) at different temperatures. Temperature-dependent PL spectra of control (**c**) and BN-optimized (**d**) QD films. **e** Infrared camera images of control and BN-optimized QD films as a function of time in air after heating to 80 °C.

As is well known, the Joule heat generated during the operation of the device would significantly deteriorate the stability of the LEDs. The BN thermally excited hyperbolic phonon polariton (HPhP) modes serve as an efficient heat management channel and can effectively improve the heat dissipation issue during the operation of the device^46^. Figure 4a, b shows the temperature-dependent capacitance-frequency characteristics of QLEDs before and after BN optimization. As the test temperature increases, the thermally activated ion migration becomes pronounced, particularly in the frequency range around 10³ Hz, resulting in an increase in device capacitance^47,48^. In contrast, BN-optimized QLEDs show negligible variation even at elevated temperatures up to 85 °C. This demonstrates that introducing BN improves the thermal stability of QLEDs and reduces ion migration caused by thermal induction. The temperature-dependent stability of QD films with and without BN is shown in Fig. 4c, d, and Supplementary Fig. 23. When heated to 100 °C, BN-optimized QD films could retain 78% of their original PL intensity, which is higher than that of only 30% for control QD films, indicating that the BN could improve the exciton recombination stability of QDs under thermal stress.

In addition, from the infrared camera images of the control and BN-optimized QD films after heating to 80 °C in air, it can be observed that after 30 s, the temperature of the control film decreased to 47.5 °C, while that of the BN-optimized QD films had already dropped to 35.9 °C (Fig. 4e and Supplementary Fig. 24). Furthermore, after running at 35 W cm^-2^ for 60 s, the temperature of the control device reached 53.4 °C, while the temperature of the BN optimized device was only 46.9 °C (Supplementary Fig. 25). These results indicate that introducing BN facilitates rapid heat diffusion, reducing thermal quenching of QDs, and lowers the temperature sensitivity of the exciton recombination behavior and electrical transport properties of QDs, which is crucial for achieving high operational stability of QLEDs.

### Performance of BN-optimized QLEDs

**Fig. 5:**
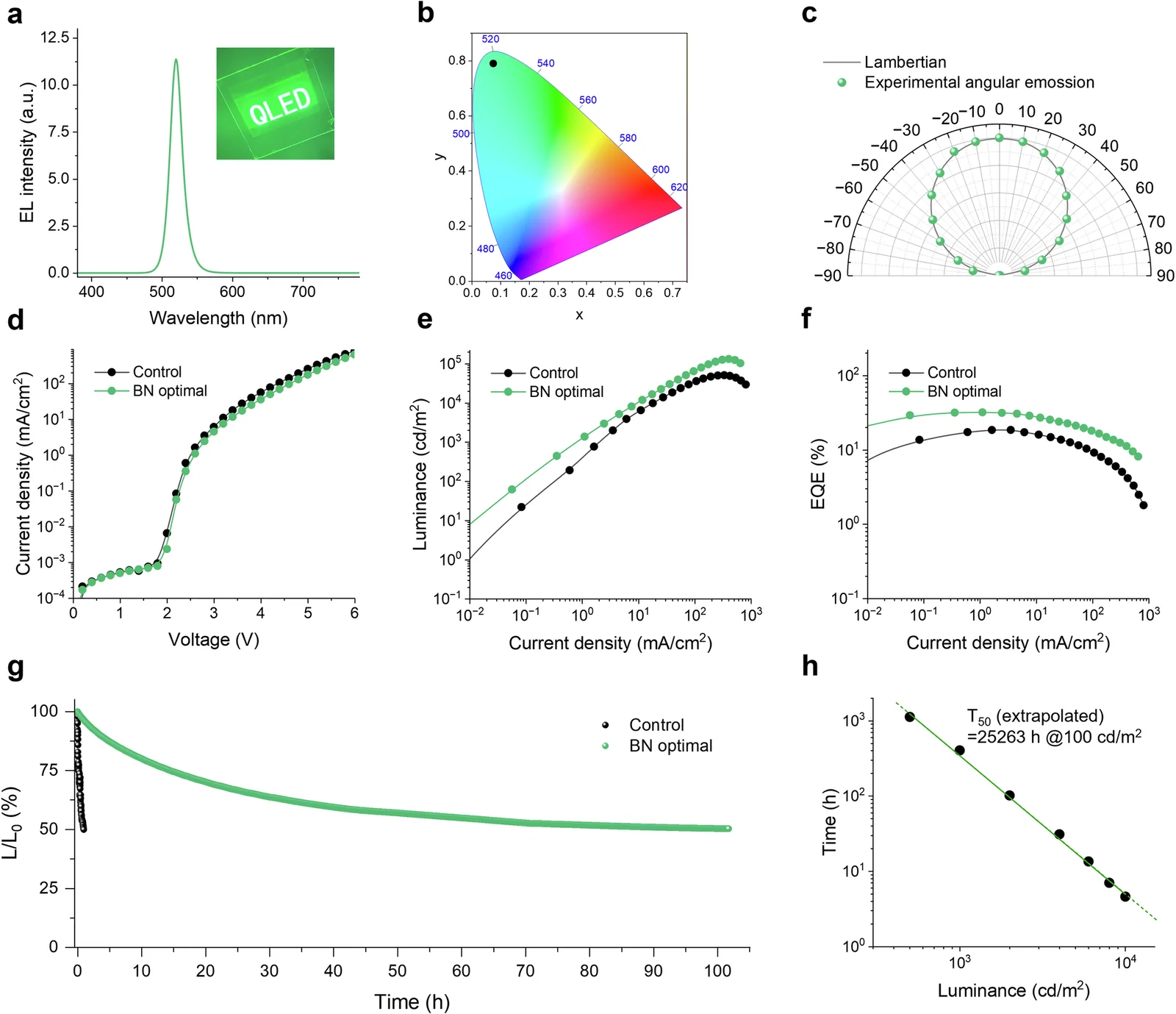
Performance of BN-optimized QLEDs. **a** EL spectra at an operation voltage of 4.0 V for QLEDs based on BN-optimized; the inset is a photograph of working QLED with a “QLED” logo under a 4.0 V bias voltage. The CIE coordinates (**b**) and angle-dependent EL (**c**) for BN-optimized QLEDs. Current density (**d**), luminance (**e**), and EQE curves (**f**) of control and BN-optimized QLEDs. **g** The operation lifetime of QLEDs based on control and BN-optimized QLEDs under an initial luminance of 2000 cd m^−2^. **h** The relationship between *T*_50_ and luminance in the accelerated lifetime experiment of BN-optimized QLEDs.

The performance of BN-optimized QLEDs was presented in Fig. 5. As displayed in Fig. 5a, the device emits green light with a FWHM of 18 nm at an EL peak of 520 nm. The working image of a typical device with a “QLED” logo under 4.0 V shows that the device emits bright and uniform green light (Fig. 5a inset). The CIE coordinates of green QLEDs are (0.075, 0.79) (Fig. 5b) and the angle-dependent EL results show that the emission of the QLEDs is a Lambertian pattern (Fig. 5c and Supplementary Fig. 26). The QLEDs before and after BN optimization exhibited similar *J-V* curves (Fig. 5d), indicating that devices maintain the original electrical properties after the introduction of BN ultrathin interlayer. The luminance-current density (*L-J*) and luminance-voltage (*L*-*V*) curves of the control and BN-optimized QLEDs are depicted in Fig. 5e and Supplementary Fig. 27. The luminance of the BN-optimized QLEDs increased progressively with increasing current density, reaching a maximal value of 132,326 cd m^-2^, which is 2.6 times higher than that of the control device (51,031 cd m^−2^). The BN-optimized QLEDs exhibited a higher luminance than that of the control device under identical carrier injection conditions, indicating that BN-optimized devices have higher electro-optical conversion efficiency (Supplementary Fig. 28).

After introducing BN, the QLEDs exhibited the champion peak EQE of 32% (verified EQE of 30.8% by other laboratory), which is 1.7-fold higher than that of the control device (18.5%) (Fig. 5f, Supplementary Fig. 29 and Supplementary Fig. 30). Moreover, the BN-optimized device has good reproducibility and the average peak EQE is 30.05%, which is also obviously higher than that of control QLEDs (17.8%) (Supplementary Fig. 31). Correspondingly, the BN-optimized device reaches a peak current efficiency of 125 cd A^-1^, much higher than the control device with a peak current efficiency of 68 cd A^-1^ (Supplementary Fig. 32). The high efficiency of BN-optimized device can be attributed to two factors: firstly, optical simulations reveal that the light extraction efficiency (LEE) of QLEDs is improved to some degree after introducing of BN (Supplementary Figs. 33–37 and Supplementary Table 2); secondly, the BN-optimized device exhibits better carrier injection balance than the control device (Supplementary Fig. 38). Importantly, the BN-optimized QLEDs exhibit a significant improvement in operational stability, with an operational lifetime of up to 102 h at *L*_0_ of 2000 cd m^-2^ (verified *T*_50_ of 99.7 h at *L*_0_ of 2000 cd m^-2^ by other laboratory) (Fig. 5g and Supplementary Fig. 39). Moreover, using the acceleration lifetime equation *L*_0_^n^*T*_50_ = constant (where n is the acceleration factor) to fit the lifetimes of LEDs under different initial luminance experiments (Supplementary Fig. 40 and Supplementary Table 3)^49,50^, the acceleration factor n = 1.84 can be obtained, as shown in Fig. 5h. By calculation, it can be determined that the extrapolated lifetime of BN-optimized QLEDs at 100 cd m^-2^ is 25,263 h, which is 104-fold improvement over that of the control device (242 h). This achievement indicates that BN modulation establishes one of the most efficient and stable devices in the field of perovskite green LEDs to date (Supplementary Table 4).

## Discussion

In summary, this study introduced ultrathin nano-layered BN to enhance the operational stability of CsPbBr_3_ perovskite QLEDs. First, the chemical adsorption of BN onto perovskite QDs is beneficial for passivating interface defects, reducing ion migration channels. Second, employing BN as a blocking layer effectively increases the migration barrier and activation energy of Br^-^, significantly impeding the migration of perovskite ions into the HTL, thereby stabilizing the perovskite structure and fundamentally alleviating performance degradation during device operation. Moreover, the excellent heat dissipation of BN reduces the sensitivity of radiative recombination behavior and electrical transport of perovskite QDs to heat, which not only reduces thermally induced non-radiative recombination but also suppresses thermally induced ion-accelerated migration, further synergistically improving the operational stability of the device. Meanwhile, the BN-optimized QLEDs exhibit an excellent carrier transport balance compared to the control device, thereby suppressing exciton quenching at interfaces and favoring device stability. Resultantly, BN-optimized QLEDs achieved an extrapolated operational lifetime *T*_50_ of 25,263 h at an *L*_0_ of 100 cd m^-2^, which is about 100-fold improvement compared to control devices (*T*_50_ = 242 h).

In addition, the BN-optimized QLEDs exhibit an average EQE of 30.05%, significantly higher than that of the control devices (EQE = 17.8%). According to the equation *η*_EQE_ = *χ·φ·γ*, where *χ* represents the light extraction efficiency, *φ* represents the radiative recombination efficiency, and *γ* represents the charge balance factor^51^. The EQE enhancement is attributed to the following three aspects. First, BN reduces the defects of QDs and directly enhances the PLQY of QDs (optimized *φ*). Then, the BN-optimized QLEDs exhibit excellent carrier transport balance compared to the control device (optimized *γ*), which enables the excitons to effectively recombine in the QD emissive layer and high exciton recombination efficiency. Furthermore, the photon recycling (PR) based on a small Stokes shift (e.g., large absorption/emission overlap) supports the cycling of re-absorption and re-emission, which is favorable for coupling and emission of photons (optimized *χ*).

This study demonstrates that integrating 2D materials into perovskites through interface engineering can significantly enhance the efficiency and stability of QLEDs, providing a pragmatic approach to improving the performance of halide perovskite-based LEDs. Currently, there are relatively few reports on the use of 2D layered inorganic materials for optimizing the performance of perovskite LEDs. This work highlights the excellent characteristics of 2D materials for perovskite optoelectronic devices, offering valuable insights to researchers for optimizing the efficiency and stability of perovskite devices.

## Methods

### Materials

Cs_2_CO_3_ (99.9%), formamidine acetate (FA(Ac), 99%), PbBr_2_ (99.99%), CsBr (99.99%) and oleic acid (OA, AR) were purchased from Macklin Inc. Tetraoctylammonium bromide (TOAB, 98%), octanoic acid (OTAc, 99%), 4-dodecylbenzenesulfonic acid (DBSA, 95%), didodecyldimethylammonium bromide (DDAB, 98%) were purchased from Aladdin Reagent Co., Ltd. Toluene and ethyl acetate were analytical grade. BN were purchased from Shanghai Yaoge Alloy Materials Co., Ltd. Poly (bis(4-phenyl)(2,4,6-trimethylphenyl) amine) (PTAA) were purchased from Xi’an Polymer Light Technology Corp. 1,3,5-Tris(1-phenyl−1H-benzimidazol-2-yl)benzene (TPBi), 2,4,6-Tris[3-(diphenylphosphinyl) phenyl]−1,3,5-triazine (PO-T2T) and poly(ethylene dioxythiophene):polystyrenesulphonate (PEDOT:PSS, AI 4083) were purchased from Vizuchem Co., Ltd. All reagents were used as received without further purification.

### Synthesis and purification of QDs

First, the cesium precursor was prepared by mixing 1 mmol Cs_2_CO_3_, 10 mL OTAc, and 16 mmol DBSA into a 20 mL vial, and the mixture was stirred at room temperature for 8 h. The PbBr_2_ precursor solution was prepared by dissolving 1 mmol of PbBr_2_, 0.03 mmol of ZnBr_2_, and 3 mmol of TOAB in toluene. FA precursor was prepared by stirring 0.015 mmol of FA(Ac) with 1 mL of OTAc. For the synthesis of FA_x_Cs_1-x_PbBr_3_ QDs, 0.85 mL of cesium precursor and 0.15 mL of FA precursor were swiftly added into 9 mL PbBr_2_ precursor solution, and reacted in open air for 2 min at room temperature. Subsequently, 3 mL of DDAB (12 mg mL^-1^) solution was added. After stirring for 2 min, the crude solution was purified twice by centrifugation with 6376 × *g* (8000 rpm) ethyl acetate for 1 min each time (the volume ratio of the crude solution to ethyl acetate was 1:2). The final obtained precipitate was re-dispersed into n-octane for further use.

### Device construction

The patterned ITO was treated with deionized water, isopropyl alcohol and acetone by ultrasonic treatment for 15 mins, and then treated with ultraviolet-ozone for 15 min before use. PEDOT:PSS were filtered by 0.25 µm filter, and then spin-coated on ITO coated glass substrate at 3000 rpm for 45 s and then heated at 120 °C for 15 min. Subsequently, it was transferred to an N_2_ atmosphere glovebox. The PTAA solution (5 mg mL^-1^) was spin-coated onto the PEDOT:PSS with a spin speed of 3000 rpm (with an acceleration of 1500 rpm s^-1^) for 45 s. The substrates were then heated at 120 °C for 20 min to remove the residual solvents. After the films had cooled to room temperature, the BN ethanol solution (0.2 mg mL^-1^, 0.5 mg mL^-1^, 1 mg mL^-1^, 2 mg mL^-1^) was spin-coated onto the PTAA with a spin speed of 3000 rpm (with an acceleration of 1500 rpm s^-1^) for 45 s. The substrates were then heated at 80 °C for 5 min. After the films had cooled to room temperature, the QDs solution (0.1 mol L^-1^) was spin-coated onto the BN layer with a spin speed of 3000 rpm (with an acceleration of 1500 rpm s^-1^) for 45 s. The substrates were then heated at 80 °C for 5 min. After annealing, the films were moved to a thermal evaporation system under a vacuum of ~5 × 10^-4 ^Pa to sequentially deposit TPBi (5 nm)/PO-T2T (35 nm) and LiF/Al electrodes (1 nm/100 nm). The light-emitting area of the device was 9 mm^2^ as defined by the overlapping area of ITO and Al electrodes.

The details of the preparation of the PTAA (5 mg mL^-1^) solution are as follows: PTAA with a relative molecular weight of 20,000 is dissolved in chlorobenzene (heated at 40 °C and placed on a magnetic stirrer at 800 rpm for 8 h to ensure complete dissolution and uniform mixing), and then the solution is filtered through an organic filter with a pore size of 0.25 µm before use.

The preparation method of BN (0.2 mg mL^-1^, 0.5 mg mL^-1^, 1 mg mL^-1^, 2 mg mL^-1^) solution is as follows: 0.1 g of BN nanosheets with an average size of ~20 nm is dispersed in 10 mL of anhydrous ethanol, then the mixture is subjected to ultrasonic treatment in an ultrasonic instrument for 2 h to ensure complete dispersion. After ultrasonic treatment, the dispersion is centrifuged at 892 × *g* for 2 min in a centrifuge and the supernatant is carefully collected as the solution for preparing BN films.

### Device encapsulation

First, using a UV-curing packaging instrument, and package the already-deposited QLED device with a glass cover plate in an N_2_ glove box. The specific process was as follows: a UV curing adhesive was pre-applied between the glass cover plate and the QLED device substrate, then placed in the packaging instrument, and UV irradiation with a duration of 15 s was applied to rapidly cross-link and solidify the UV curing adhesive, achieving a firm and airtight bond between the glass cover plate and the QLED device substrate.

### Optical characterization

The PL spectra were measured by a fluorescence spectrometer (F-7000, Hitachi). The PLQY were measured using a Horiba Fluorolog system equipped with a single grating and a Quanta-Phi integrating sphere coupled to the Fluorolog system. The ultrafast TA measurements were performed on a femtosecond (fs) pump−probe system (Helios, Ultrafast System LLC) under ambient conditions. TEM images were obtained on a Talos F200X microscope. Time-resolved PL (TRPL) decay curves of the films were obtained using an FLS-1000 spectrofluorometer. A ToF-SIMS (ION-ToF ToF-SIMS V) was used to depth-profile the perovskite QLED devices. Before the ToF-SIMS deep analysis of QLEDs, the prepared QLEDs with or without BN interlayers need to be powered on at a luminance of 2000 cd m^-2^ for 40 min for electrical aging.

### Device performance test

The performance of the encapsulated QLEDs was tested using the testing system provided by Suzhou Fushida Scientific Instrument Co., Ltd. (Model 2602B source meter and CS-2000 spectrophotometer). Firstly, the luminance-current-voltage (*L-J-V*) curve was measured by sweeping the voltage from 0 to 6 V with a step of 0.2 V to determine the initial luminance (*L*_0_) and the corresponding current. Then, with this current as the constant driving condition, the luminance decay (*L*_t_) was monitored in real time using the CS-2000. The test was stopped when *L*_t_ dropped to 50% of *L*_0_, and the total time (*T*_50_) was recorded as the lifetime. The test conditions are at a temperature of 25 °C and a humidity of 25%. The data was automatically collected and analyzed by the accompanying software. All QLEDs were tested after being encapsulated in N_2_.

### Structural characterization

The atomic force microscopy (AFM) images and roughness analysis were obtained using the AFM system (INNOVA AFM, Bruker). The etching X-ray photoelectron spectroscopy (XPS) analyses were performed on a ESCALAB 250xi spectrometer. The incident monochromatic X-ray beam from the Al target (1486.6 eV, 10 mA) was focused on a 0.2 mm spot surface. The electron energy analyzer was operated at a pass energy of 50 eV. The step size of 0.05 eV was employed, and each peak was scanned five times. The C1s photoelectron line (BE = 284.8 eV) was used to calibrate the binding energies of the photoelectrons. The etching depth of BN-optimized samples is set to 25 nm. Fourier transform infrared (FTIR) spectra were collected on Nicolet IS10.

### DFT calculations

All the density functional theory calculations are performed by using the VASP codes and PAW pseudopotentials. Structures are fully relaxed under the Generalized Gradient Approximation (GGA) with Perdew-Burke-Erenzerhof (GGA-PBE) exchange-correlation. A kinetic energy cutoff of 500 eV is set on a grid of 1×1×1 k-points for perovskite/BN combined systems. In order to consider the vdW force between BN and halide perovskites, DFT-D3 correction is used for all the calculations. The energy barriers are extracted through a state-of-the-art method, the Artificial Intelligence-Driven Nudged Elastic Band (AID-NEB) algorithm. The ‘improved-tangent’ tag is selected as the neb-method. The maximum force and uncertainty for convergence are set to be smaller than 0.05 eV Å^-1^ and 0.05 eV, respectively^52,53^.

The adsorption energy is calculated as the following equation

1 $${E}_{{ads}}=({E}_{{tot}}\left({perovskites}+{BN}\right)-{E}_{{tot}}\left({BN}\right)-{E}_{{tot}}\left({perovskites}\right))/n$$

where the *E*_tot_(perovskites+BN), *E*_tot_(BN), *E*_tot_(perovskites), and n are the total energy of adsorption system, total energy of isolating BN nanoplate, total energy of isolating perovskites surface, and the number of perovskites unit cell, respectively.

### Optical simulation

Finite-Difference Time-Domain (FDTD) simulation is conducted using the Lumerical FDTD software package. The boundary conditions for the four lateral boundaries are assumed to be perfect mirrors, enabling the limited lateral dimension to be treated as infinite. The top and bottom boundary conditions are set to a perfectly matched layer (PML), which absorbs incident electromagnetic energy. The lateral dimension of the computational domain is set to 2 × 2 μm^2^. A single dipole source with a peak emission wavelength of 520 nm is placed in the QD layer^54–56^.

## Supplementary information

Supplementary Information

Transparent Peer Review file

## Source data

Source data

## Data Availability

The authors declare that the main data supporting the findings of this study are available within the article and its supplementary information files. Source data are provided with this paper.
