# Supplementary material for: Ultrathin nano-layered boron nitride stabilizing perovskite quantum dot-based light-emitting diodes
Source: Nat Commun. 2026 Sep 1;17:10415. doi: 10.1038/s41467-026-77166-z (PMC13631234; doi:10.1038/s41467-026-77166-z)
Supplement: Supplementary file 1 [file 41467_2026_77166_MOESM1_ESM.pdf]

Supplementary Materials for

Ultrathin nano-layered boron nitride stabilizing perovskite quantum dot-based light-emitting diodes

Jindi Wang[1], Hongdan Ren[1], Bo Cai[2], Mingyang Li[1], Wenxuan Fan[1], Leimeng Xu[1], Jisong Yao[1], Shalong Wang[1], Jizhong Song[1,*]

[1]*Key Laboratory of Materials Physics of Ministry of Education, Laboratory of Zhongyuan Light, School of Physics, Zhengzhou University, Daxue Road 75, Zhengzhou 450052, China.*

[2]*State Key Laboratory of Organic Electronics and Information Displays, Nanjing University of Posts & Telecommunications, Nanjing 210023, China*

E-mail: songjizhong@zzu.edu.cn

Keywords: perovskite QDs, QLEDs, boron nitride, inhibit ion migration, stability

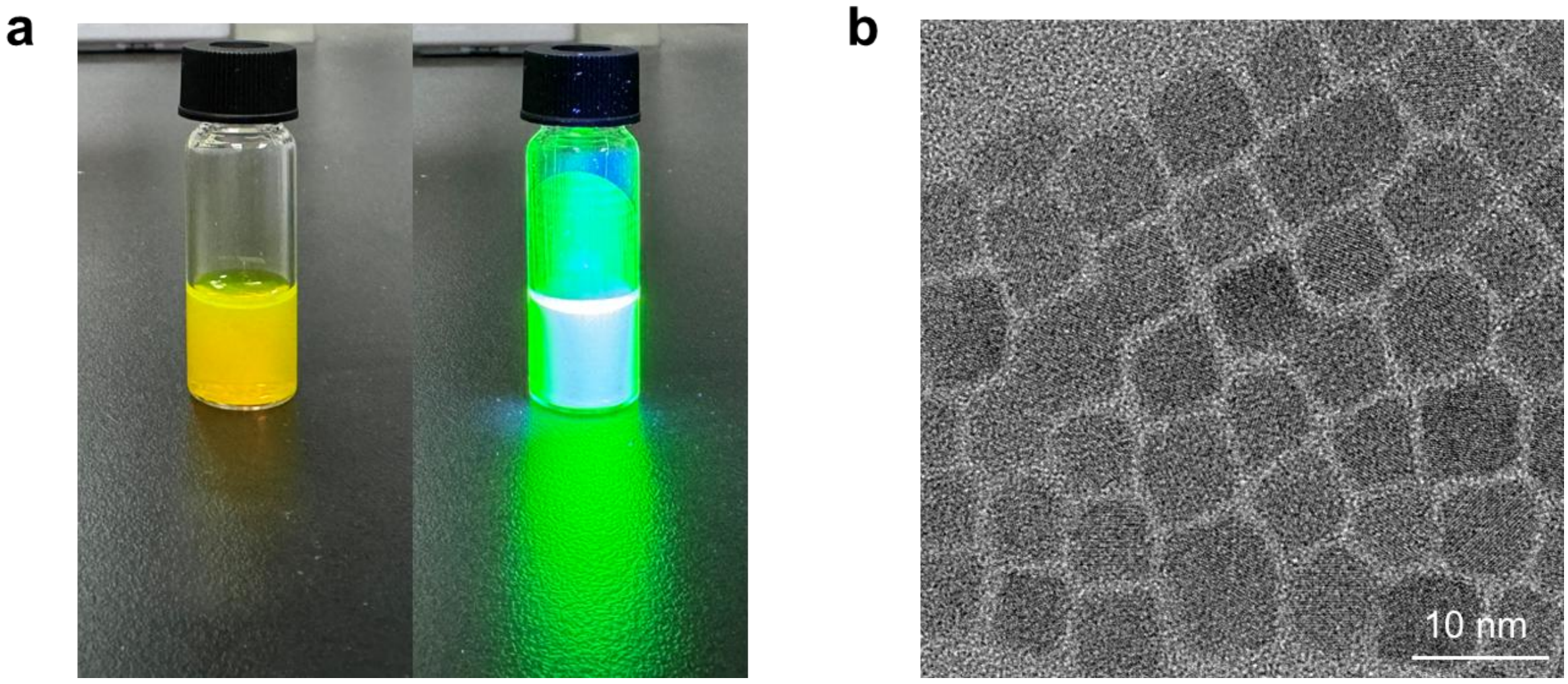

Supplementary Fig. 1 | Optical and TEM characterizations of QDs and QD ink. (**a**) The photos of QDs ink under natural light and 365 nm ultraviolet light; (**b)** the TEM images of QDs.

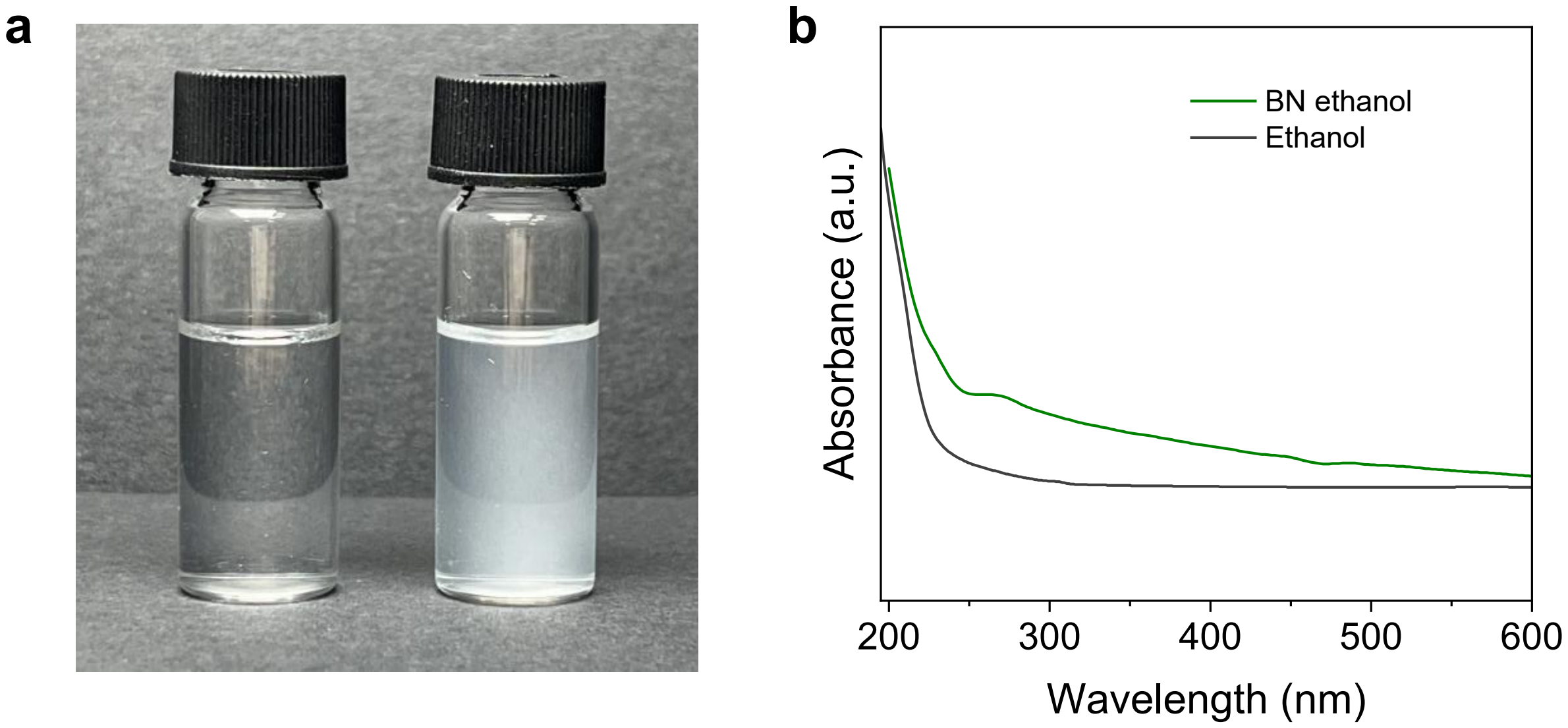

Supplementary Fig. 2 | Photographs and absorption spectra of BN pre- and post-dispersion. The photos **(a)** and absorption spectra **(b)** before and after BN dispersion.

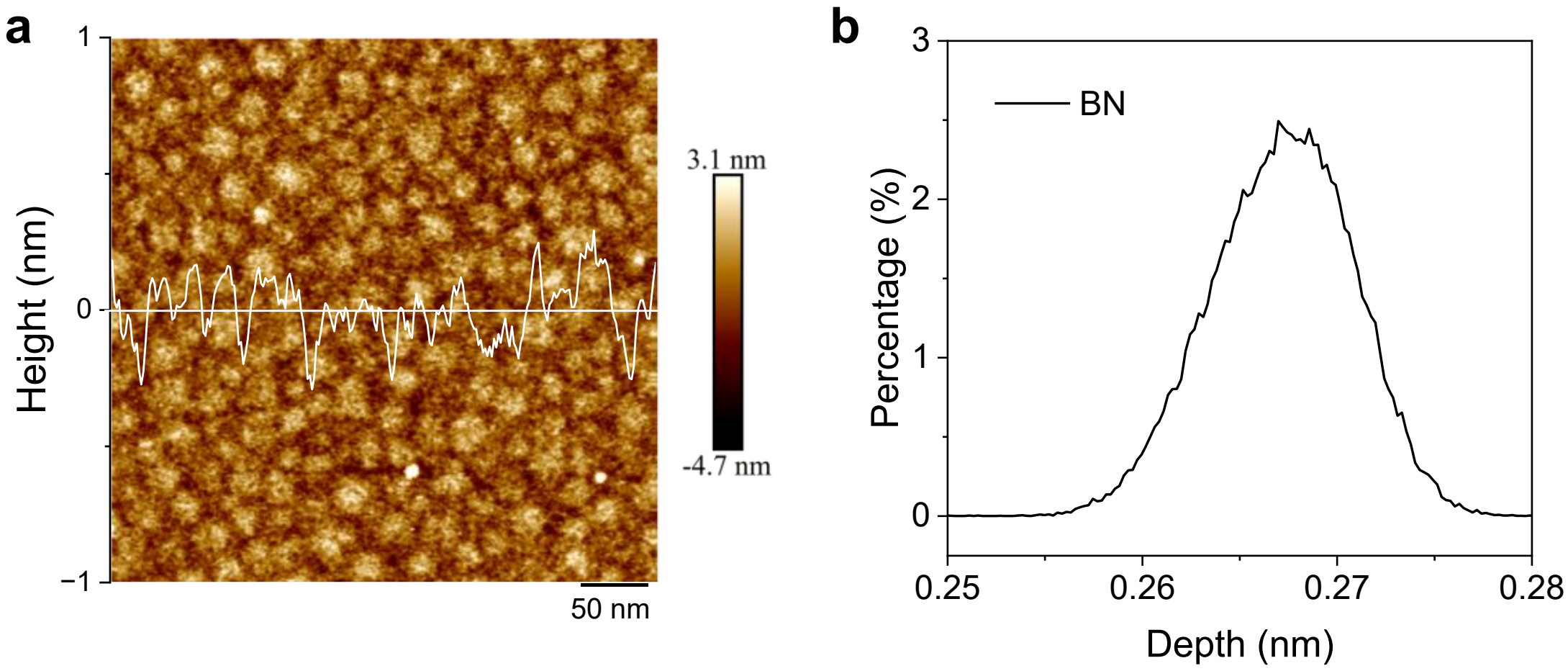

Supplementary Fig. 3 | Morphology and depth analysis of high-concentration BN films. The AFM image **(a)** and its corresponding depth statistical chart **(b)** of the high concentration BN films.

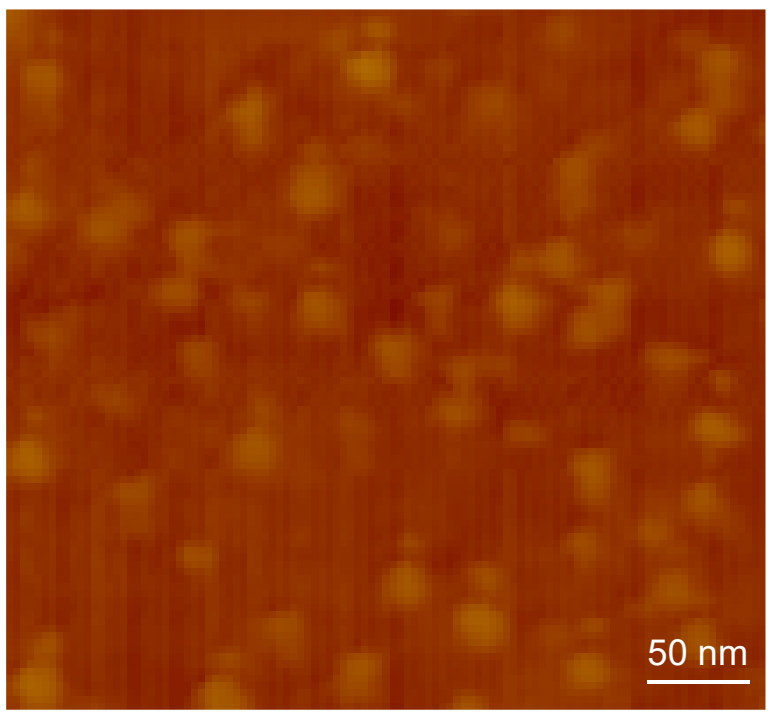

Supplementary Fig. 4 | The AFM image of ultra-thin nanolayer BN films.

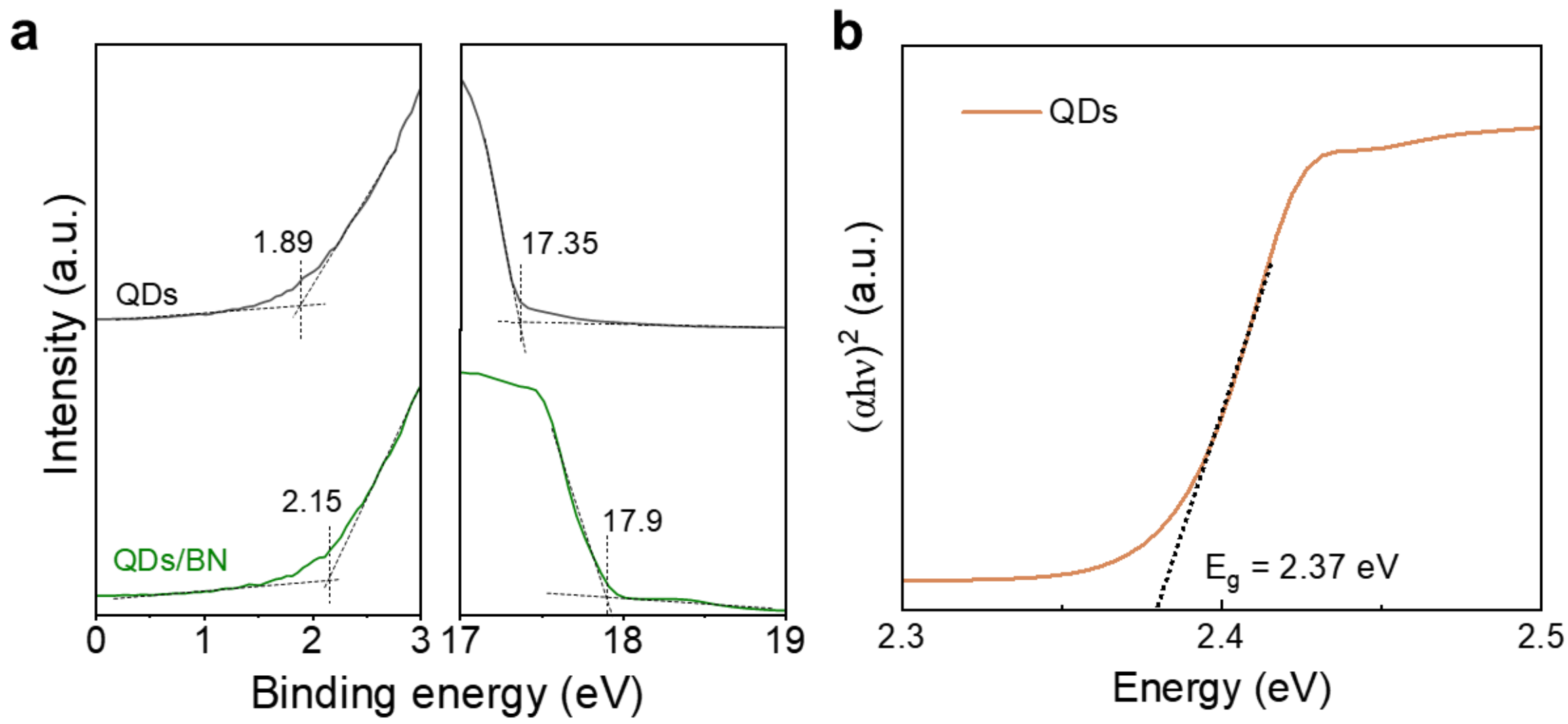

Supplementary Fig. 5 | UPS and optical bandgap characterizations. (**a**) High-binding-energy secondary electron cutoff and valence-band edge regions of QDs and QDs/BN film measured by UPS; (**b**) Bandgap values of QD films from absorption spectra.

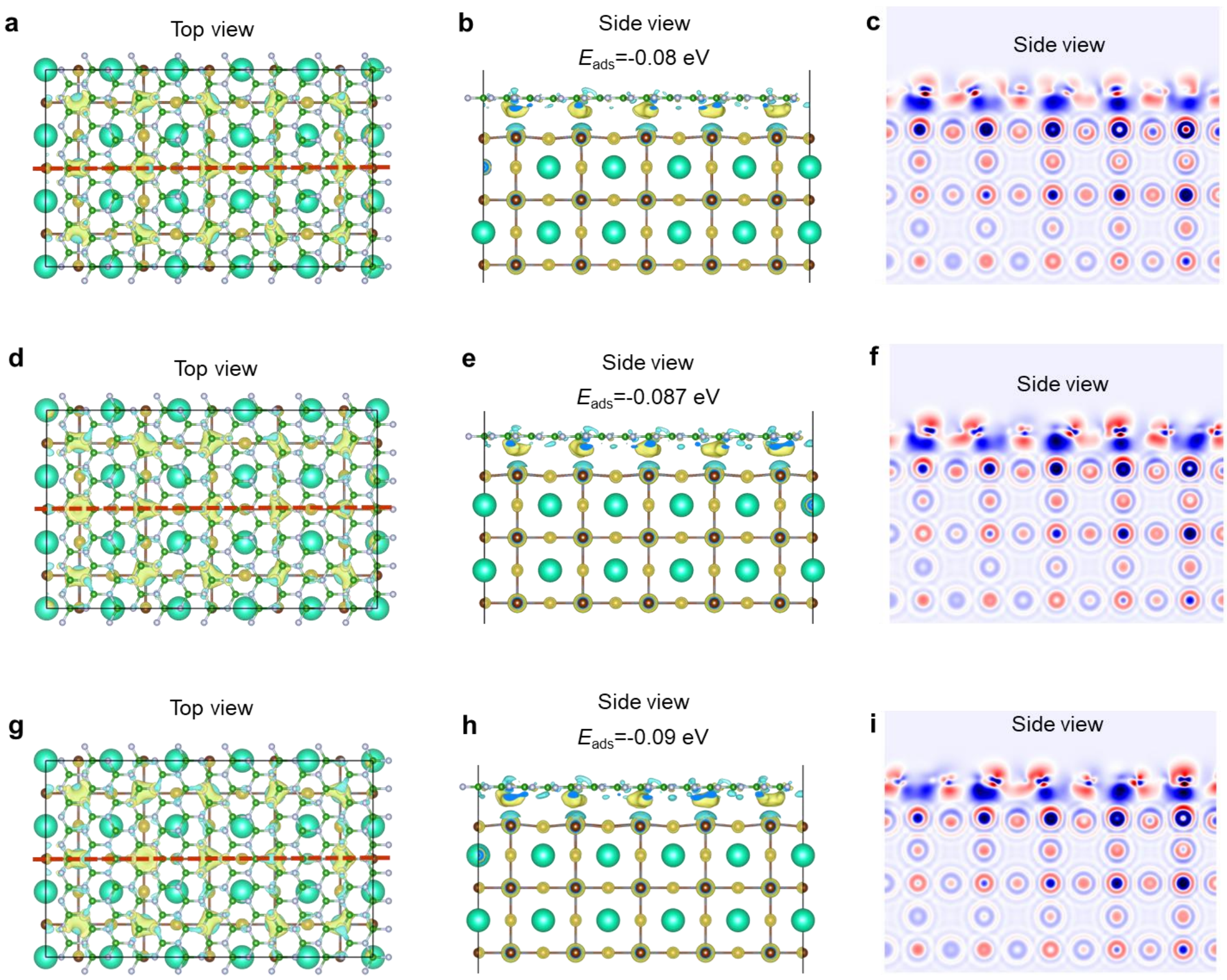

Supplementary Fig. 6 | Charge density difference analysis of differently $PbBr_2$-terminated $CsPbBr_3$. 3D and 2D slice of charge density differences of (**a-c**) $PbBr_2$-1 terminated $CsPbBr_3$; **(d-f)** $PbBr_2$-2 terminated $CsPbBr_3$; **(g-i)** $PbBr_2$-3 terminated $CsPbBr_3$.

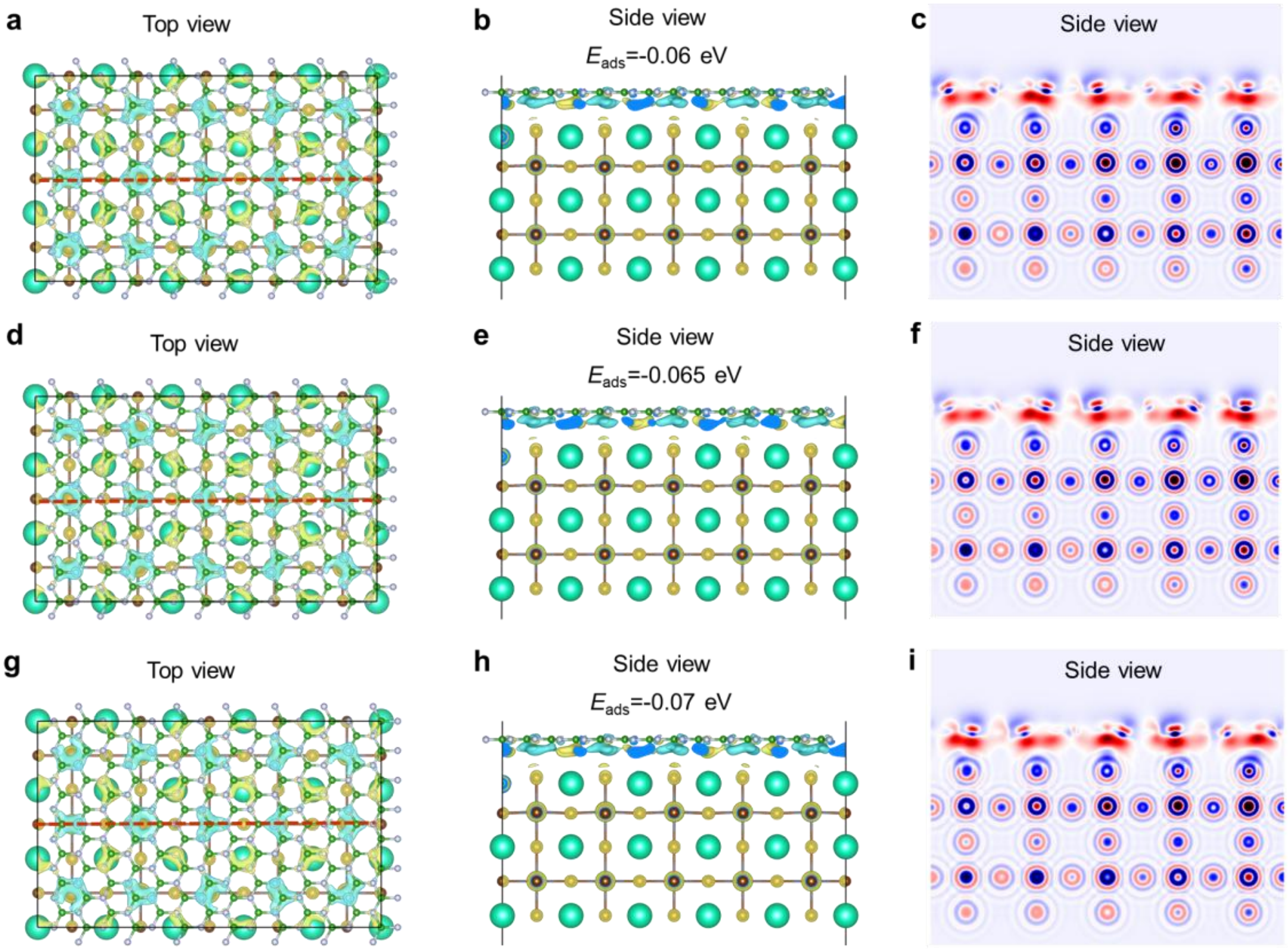

Supplementary Fig. 7 | Charge density difference analysis of differently CsBr-terminated $CsPbBr_3$. 3D and 2D slice of charge density differences of **(a-c)** CsBr-1 terminated $CsPbBr_3$; **(d-f)** CsBr-2 terminated $CsPbBr_3$; **(g-i)** CsBr-3 terminated $CsPbBr_3$.

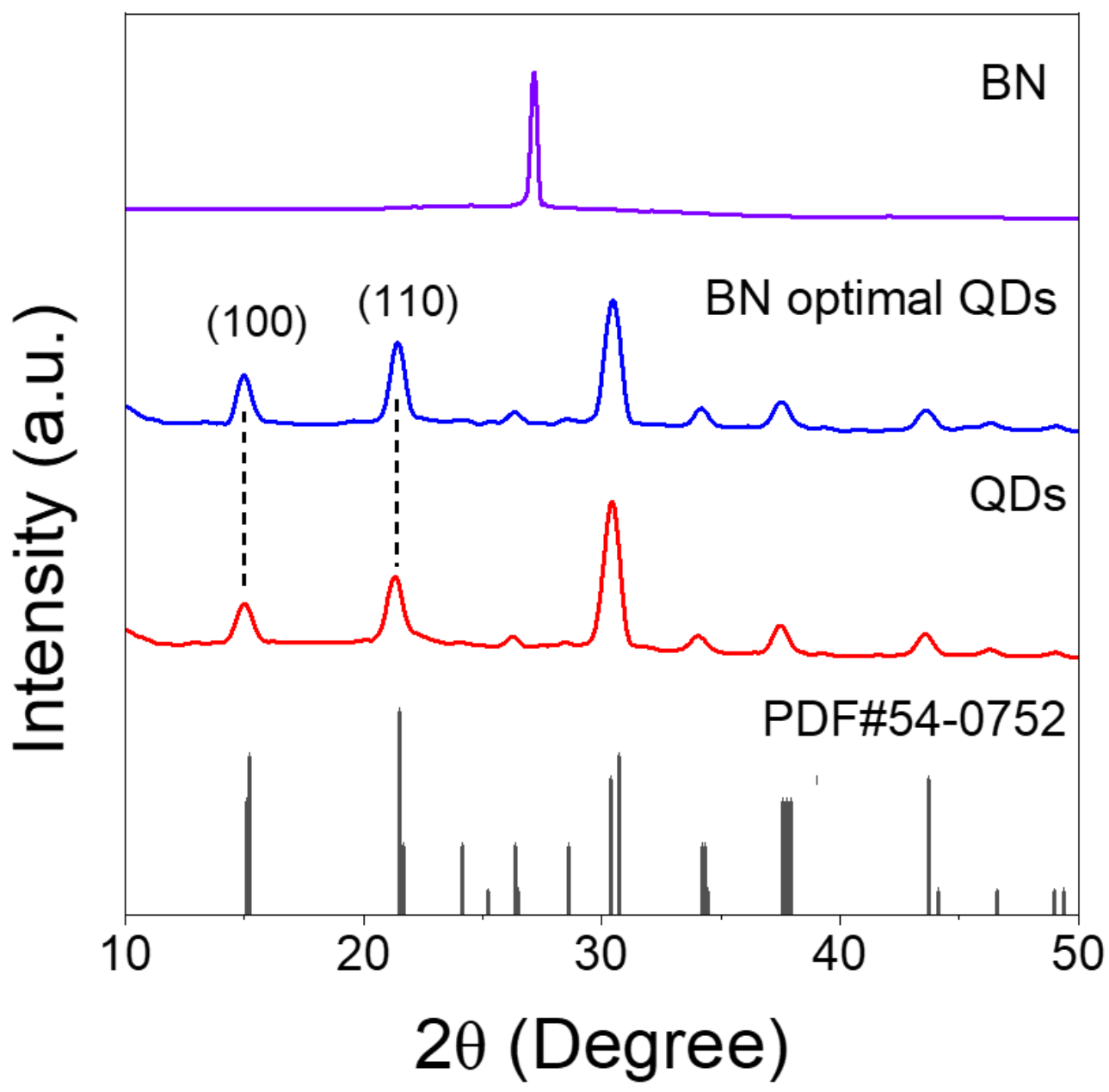

Supplementary Fig. 8 | The XRD spectra. The XRD spectra of BN-optimized QD films and BN powder.

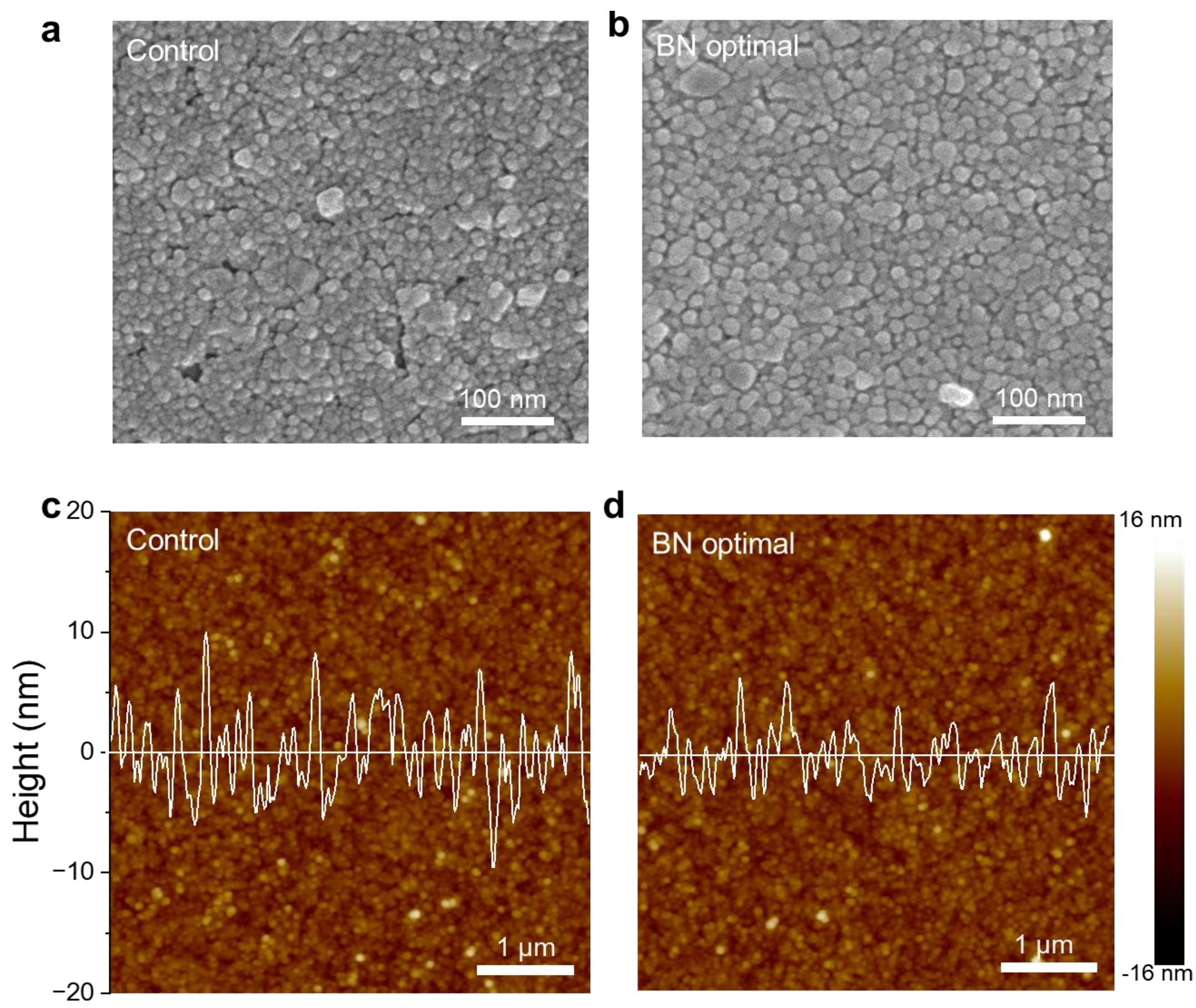

Supplementary Fig. 9 | Morphological characterization of control and BN-optimized QD films. The SEM **(a,b)** and AFM images **(c,d)** of control and BN-optimized QD films.

The BN-optimized QD films have a denser surface, and the root-mean-square roughness is reduced from 2.0 nm for the control samples to 1.5 nm.

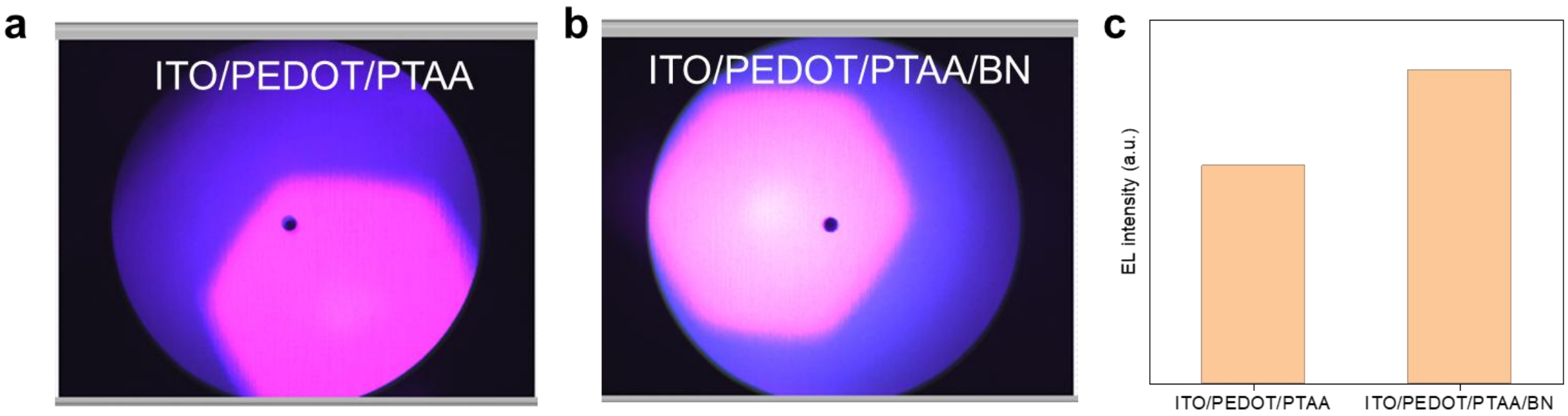

Supplementary Fig. 10 | Light extraction performance of QD devices. The light extraction intensity of the ITO/PEDOT/PTAA and ITO/PEDOT/PTAA/BN structures.

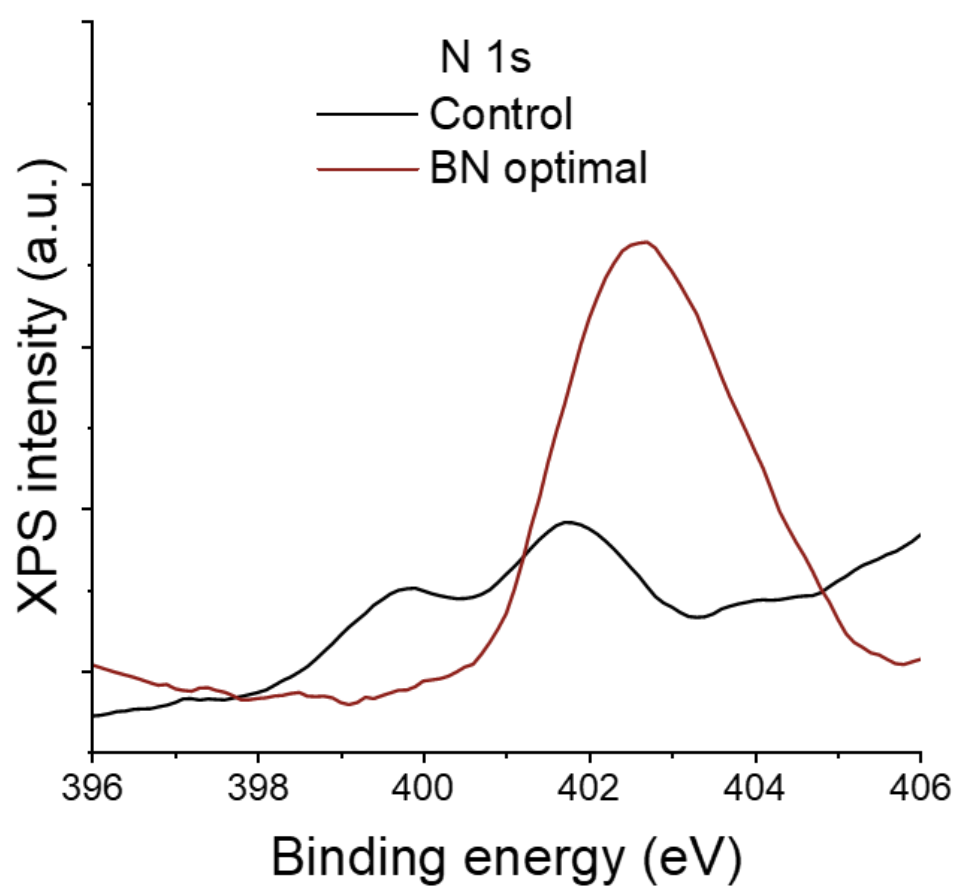

Supplementary Fig. 11 | High resolution XPS spectra of N 1s.

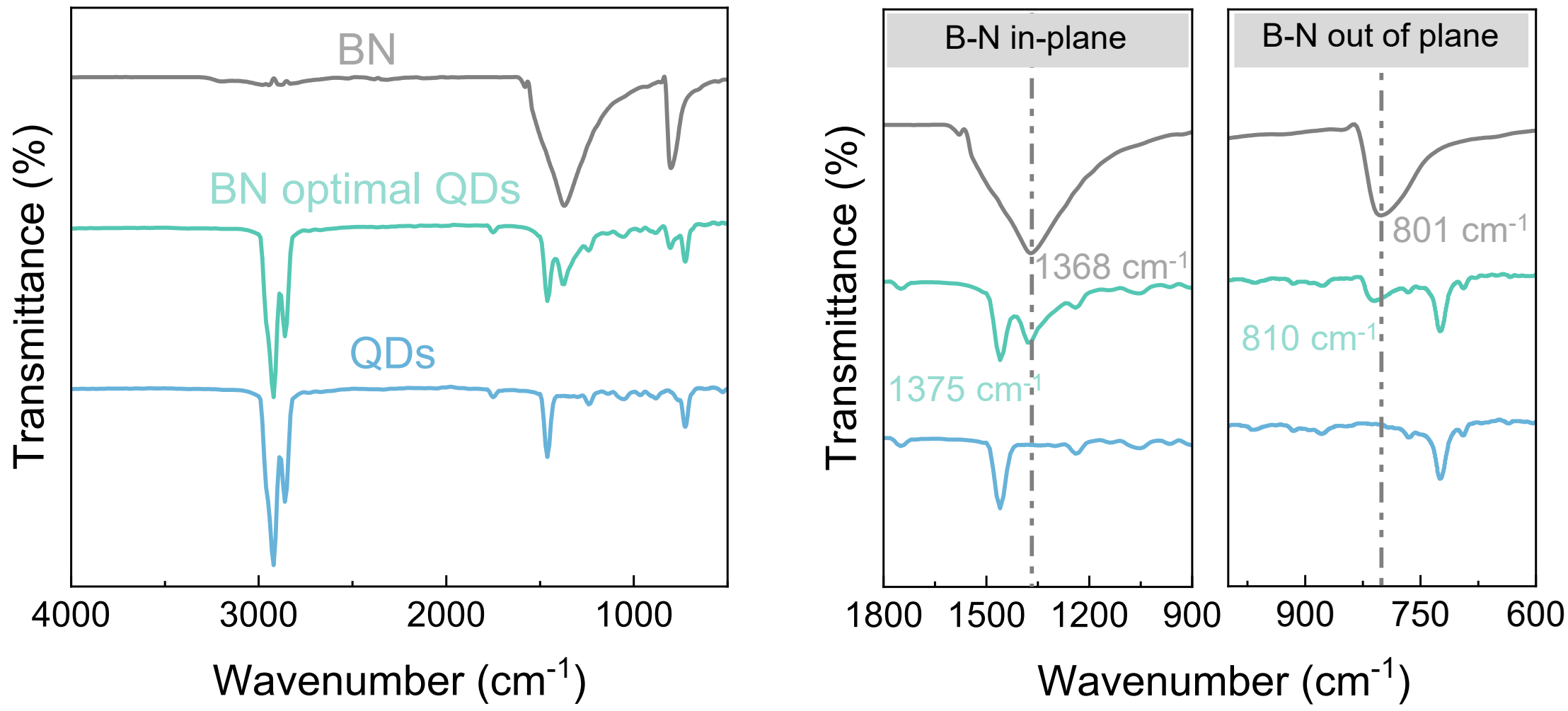

Supplementary Fig. 12 | FTIR spectroscopic characterization. FTIR spectra of QDs, BN-optimized, and BN films.

The BN-optimized QD films showed the in-plane B-N stretching mode blue-shifting to1375 cm^{-1} and an out-of-plane bending mode blue-shifting to 810 cm^{-1}, which is different that of pristine BN (1368 and 801 cm^{-1}), indicating that produces electronic interaction of perovskite and BN.

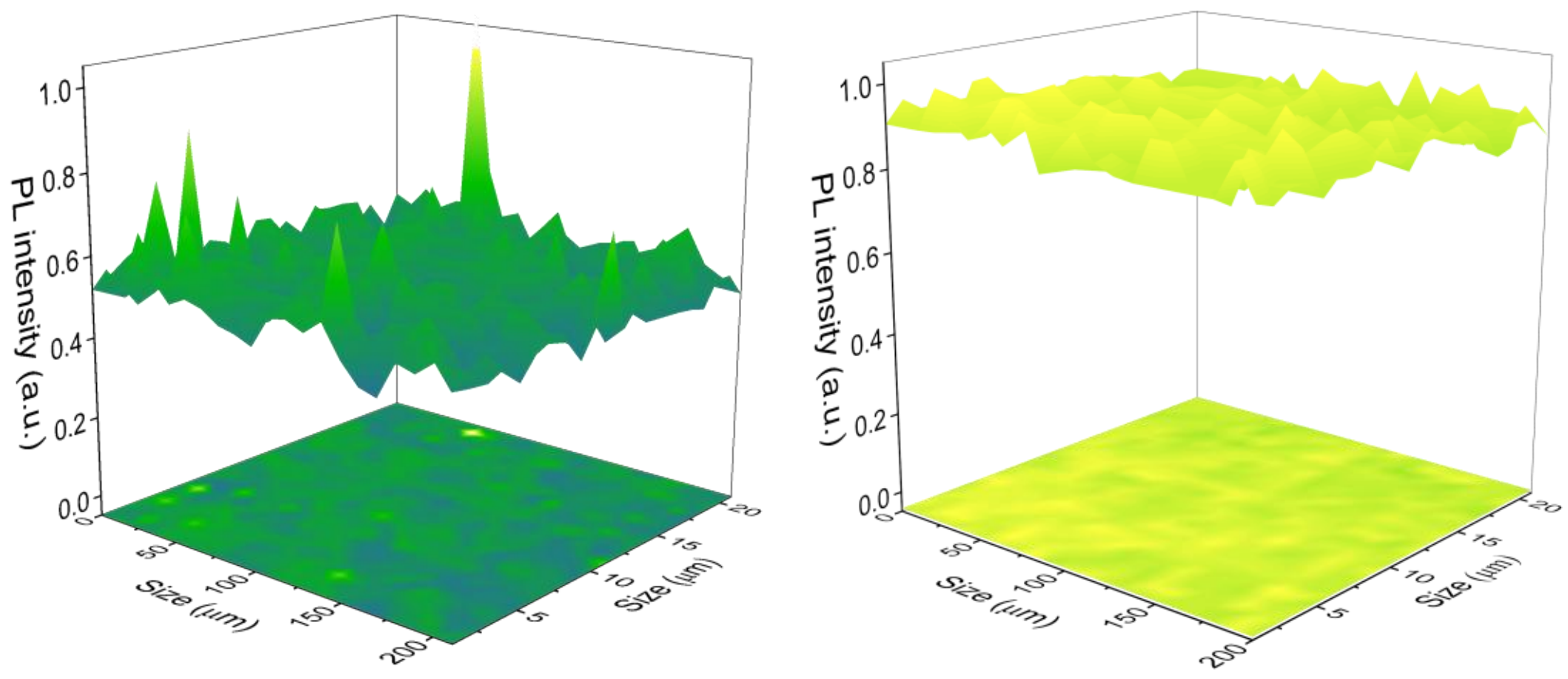

Supplementary Fig. 13 | PL mapping analysis. The PL color mapping images of control and BN-optimized QD films.

PL color mapping images show that compared to QD films, BN-optimized QD films exhibit higher and more uniform PL intensity.

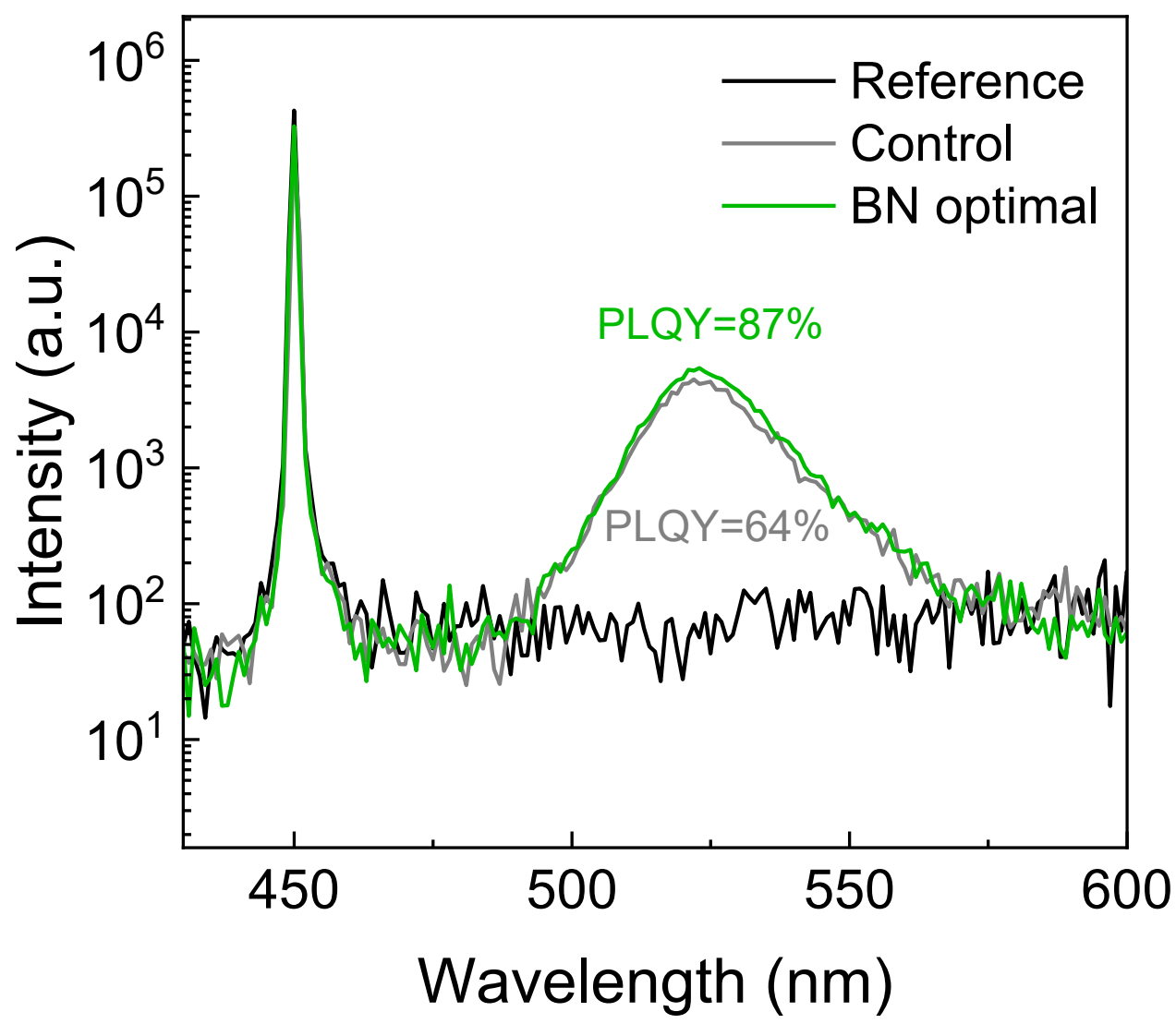

Supplementary Fig. 14 | The PLQY of control and BN-optimized samples.

Compared to the control samples (PLQY=64%), BN-optimized QD films have a higher PLQY of 87%.

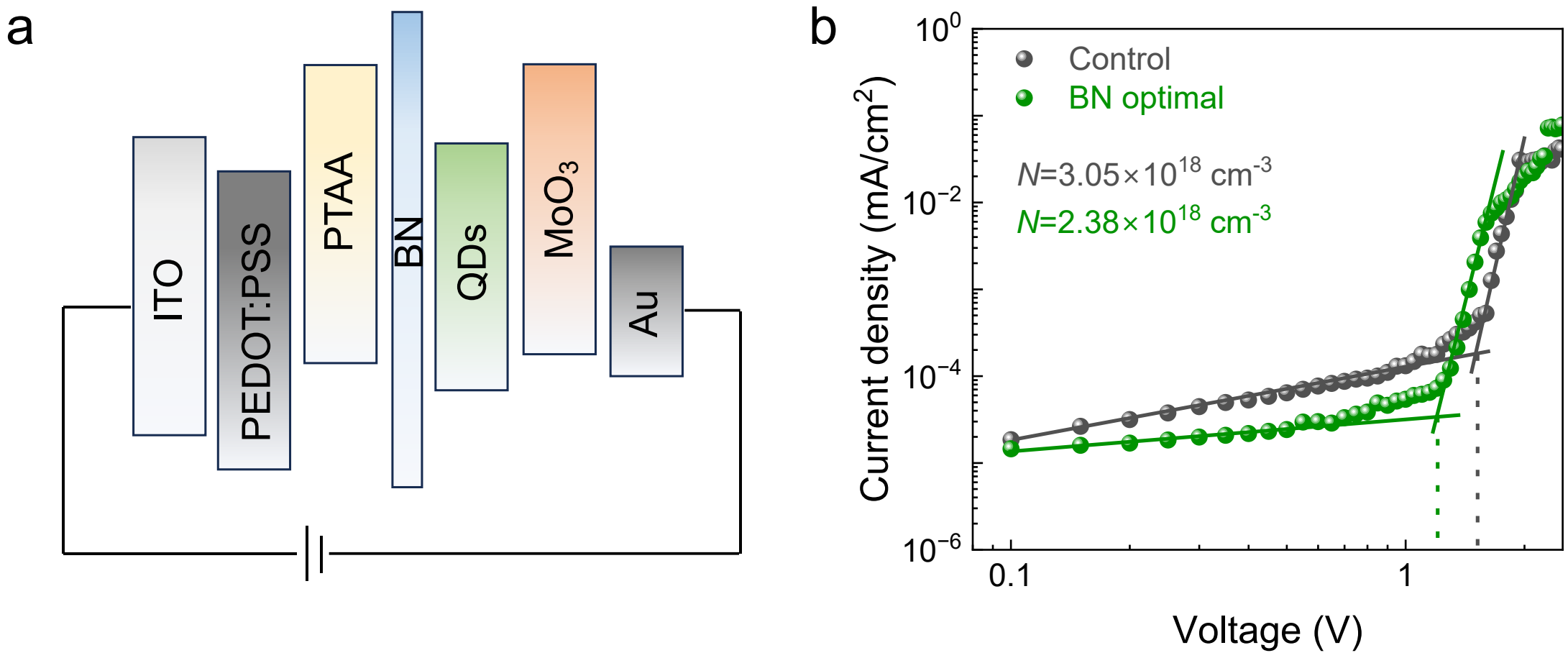

Supplementary Fig. 15 | Electrical characterization of hole-only devices. (a) Schematic diagram of "hole-only" device structure and **(b)** current density–voltage characteristics.

The defect states are quantitatively analyzed by space-charge-limited current (SCLC) via a "hole-only" device with a structure of ITO/PEDOT:PSS/PTAA/(BN)/QDs/MoO_3/Au (Supplementary Fig. 15a). The sharp rise of the current density–voltage (J–V) curve correlates with a trap-filled limit (TFL), where the defects are occupied by charge carriers (Supplementary Fig. 15b). The defect density is calculated according to the equation $N_{defects}=(2\varepsilon\varepsilon_0 V_{TFL})/(eL^2)$, where ε (22) and ε_0 (8.85×10^{14}) are the dielectric constants of the perovskite and the vacuum permittivity, respectively, L (35 nm) is the thickness of perovskite films, and e (1.60×10^{19}) is the elementary charge. The electron trap density of BN-optimized QD films is 2.38×10^{18} cm^3, smaller than that of 3.05×10^{18} cm^3 for QD samples.

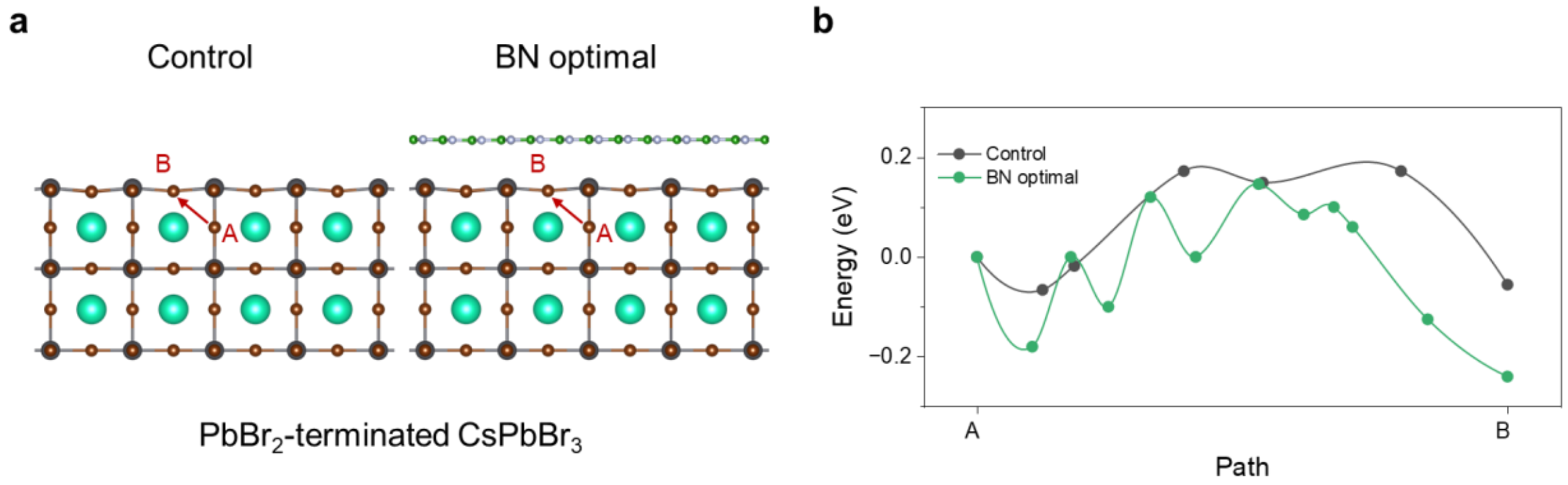

Supplementary Fig. 16 | Adsorption energy pathway and barrier analysis. The simulation path **(a)** and potential barriers **(b)** of $PbBr_2$-terminated surface $CsPbBr_3$ without/with BN adsorption.

The corresponding energy barriers of $PbBr_2$-terminated surface systems are ~0.24 eV and ~0.32 eV for without and with BN systems, respectively. This is consistent with the results of CsBr-terminated surface $CsPbBr_3$, indicating that BN plays positive role in suppressing the migration of Br^-.

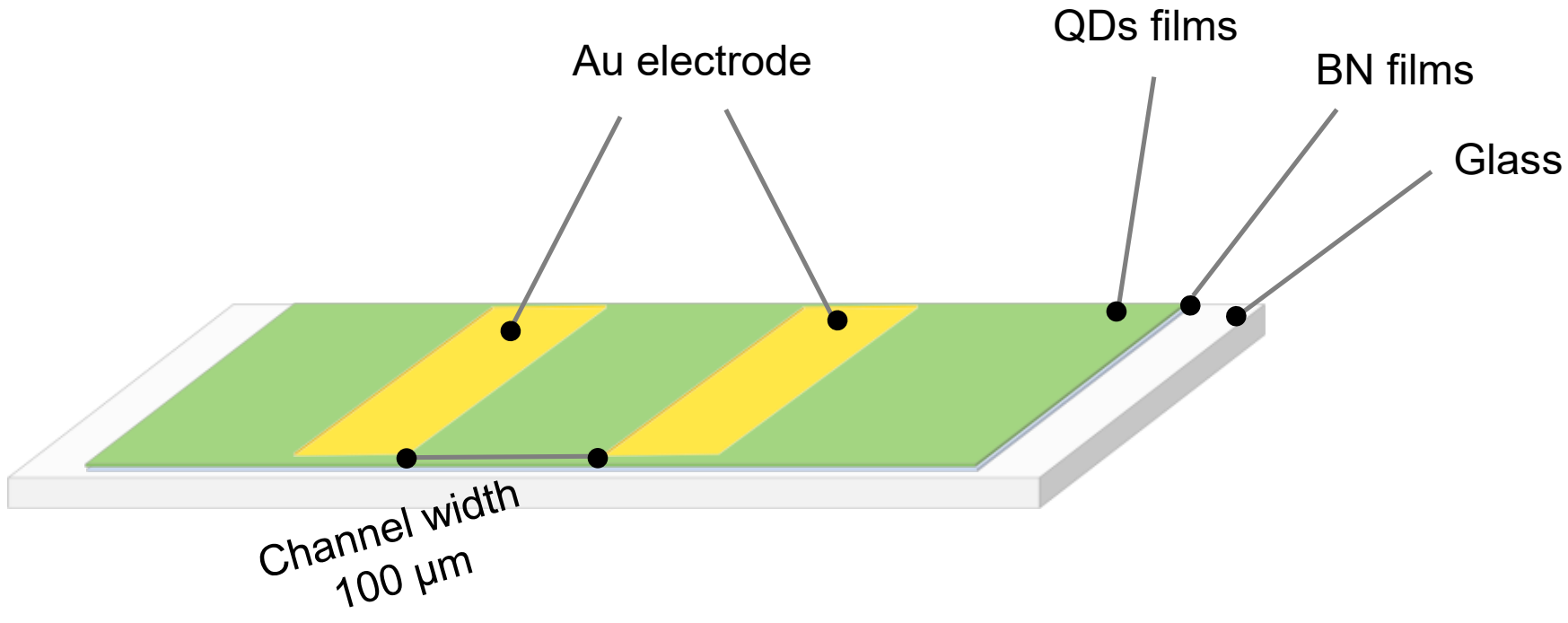

Supplementary Fig. 17 | Schematic of conductivity test setup for σ(T) characterization. Schematic diagram of device structure for temperature-dependent conductivity σ(T) testing.

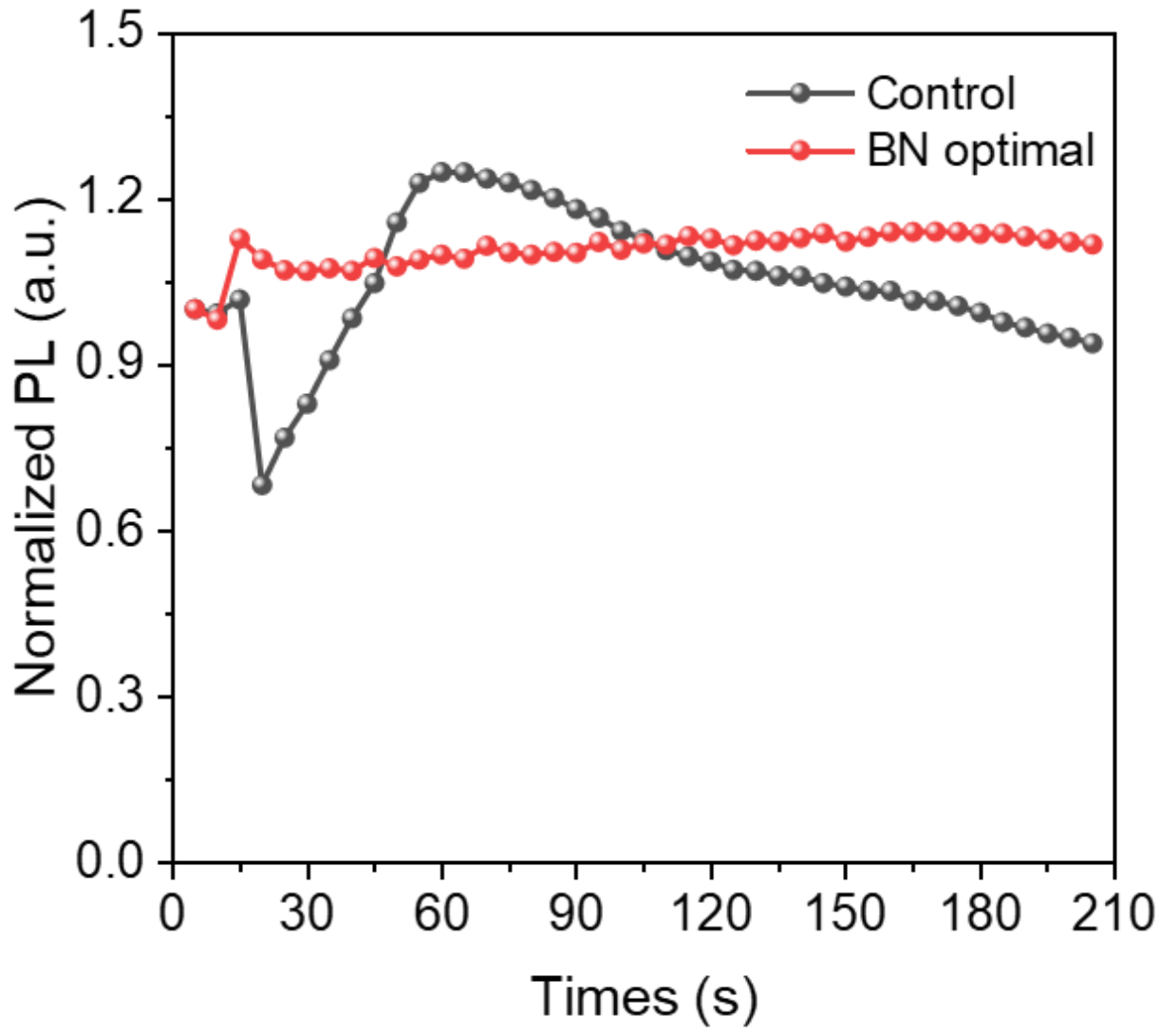

Supplementary Fig. 18 | Time-resolved PL intensity. Time-resolved PL intensity for ITO/QDs/Al and ITO/BN/QDs/Al devices under 3.0 V bias.

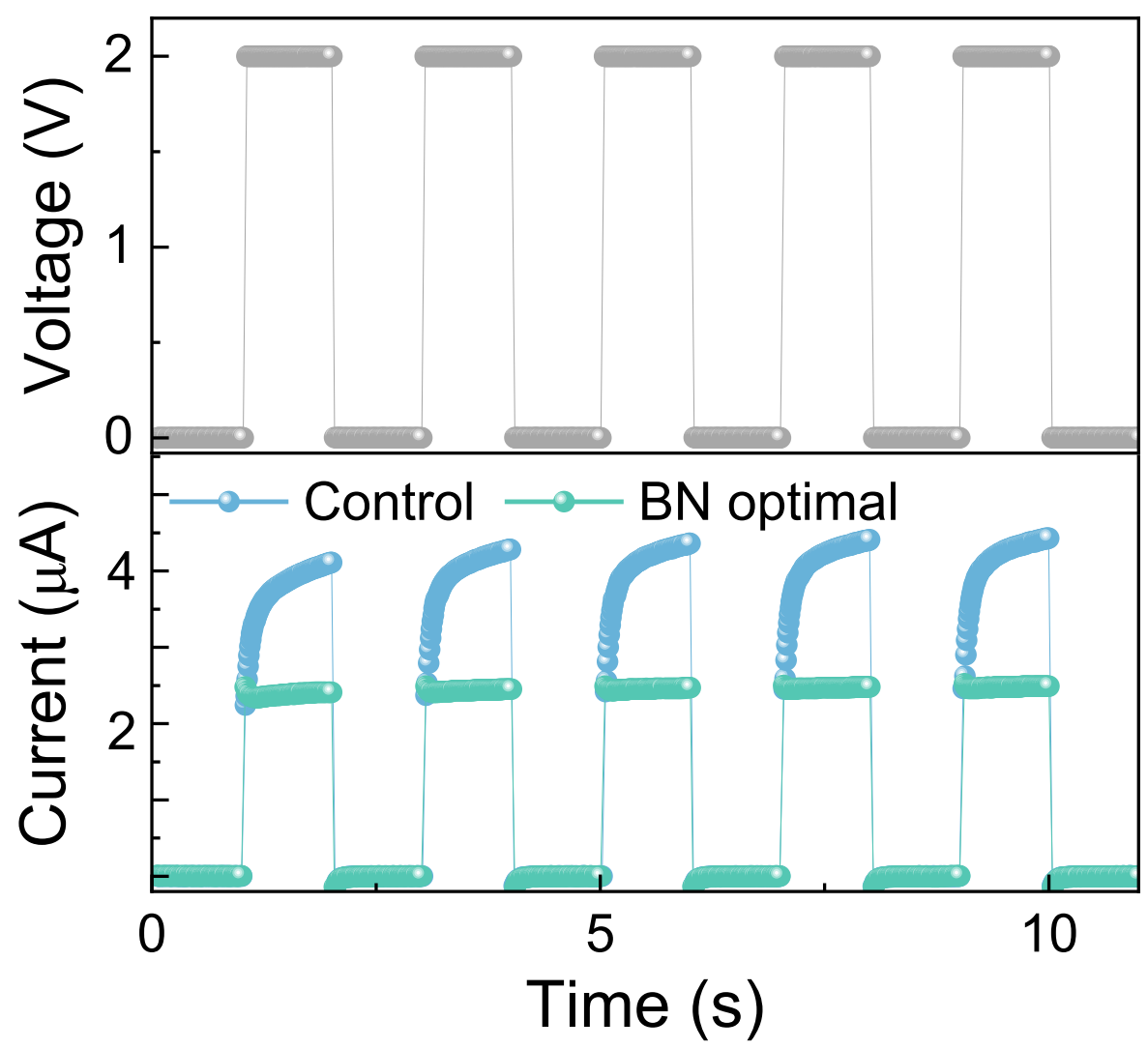

Supplementary Fig. 19 | Transient current response of QLEDs. Transient current response of QLEDs under 2.0 V voltage with a cycle of 2 s.

Before applying voltage, the current of both devices is 0 μA. However, when a voltage of 2.0 V is applied, the current of the control device continues to increase and then gradually stabilizes, indicating that ion migration within the control system is more pronounced. In contrast, the device with BN adsorption exhibits relatively stable current, further demonstrating that introducing a BN layer can effectively suppress ion migration.

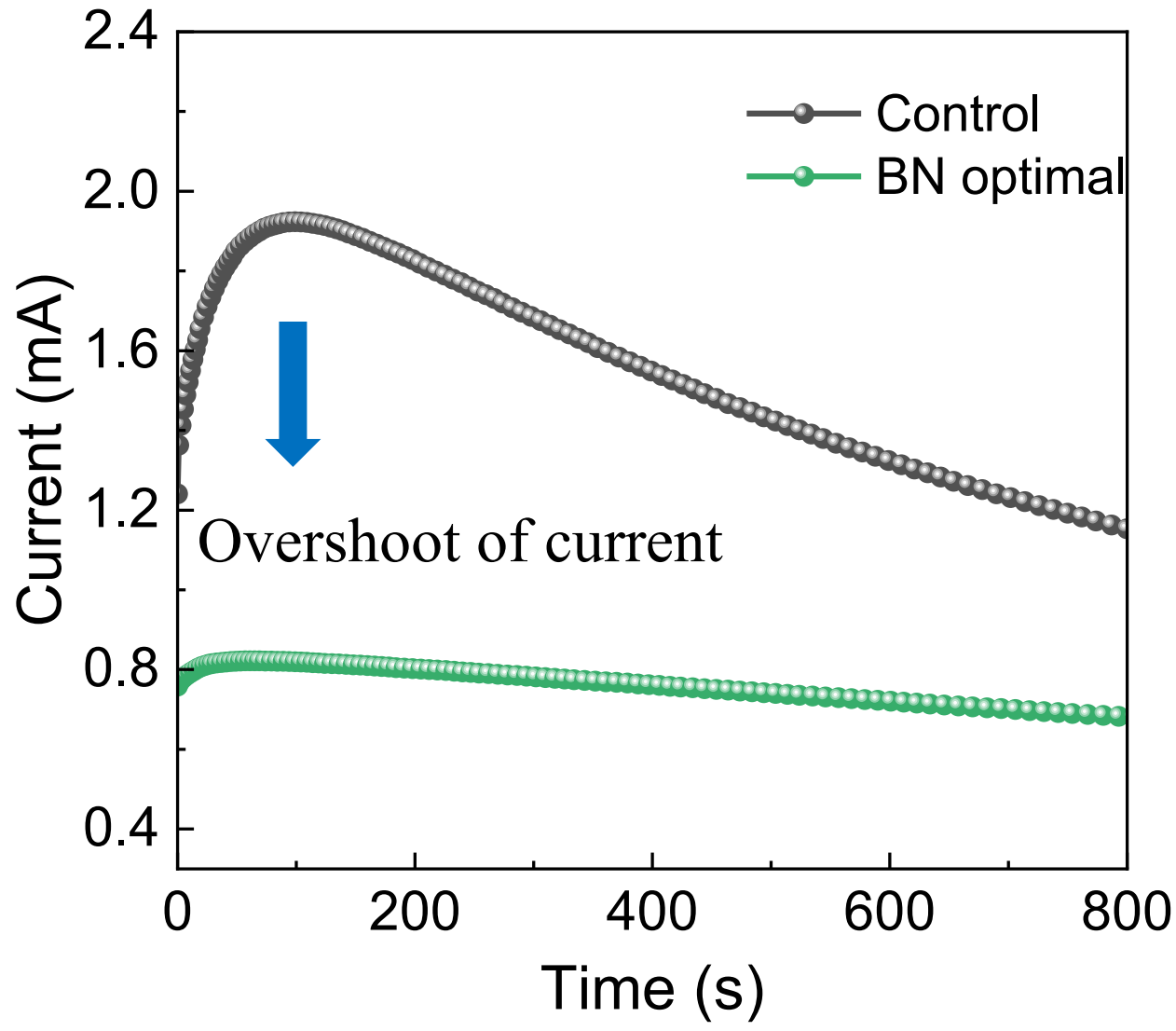

Supplementary Fig. 20 | The time-dependent change in current under constant voltage.

Under constant bias voltage of 3.0 V, the device with BN optimization maintains a relatively stable current during testing, which was due to the inhibition of ion migration by the ultrathin nano-layered BN, keeping the local electric field at the interface stable. In contrast, the control device exhibits severe current overshoot at the initial stage of the measurement. Here we attribute this phenomenon to rapid charge injection caused by the narrowing of the depletion region width or the reduction of the injection barrier due to ion migration.

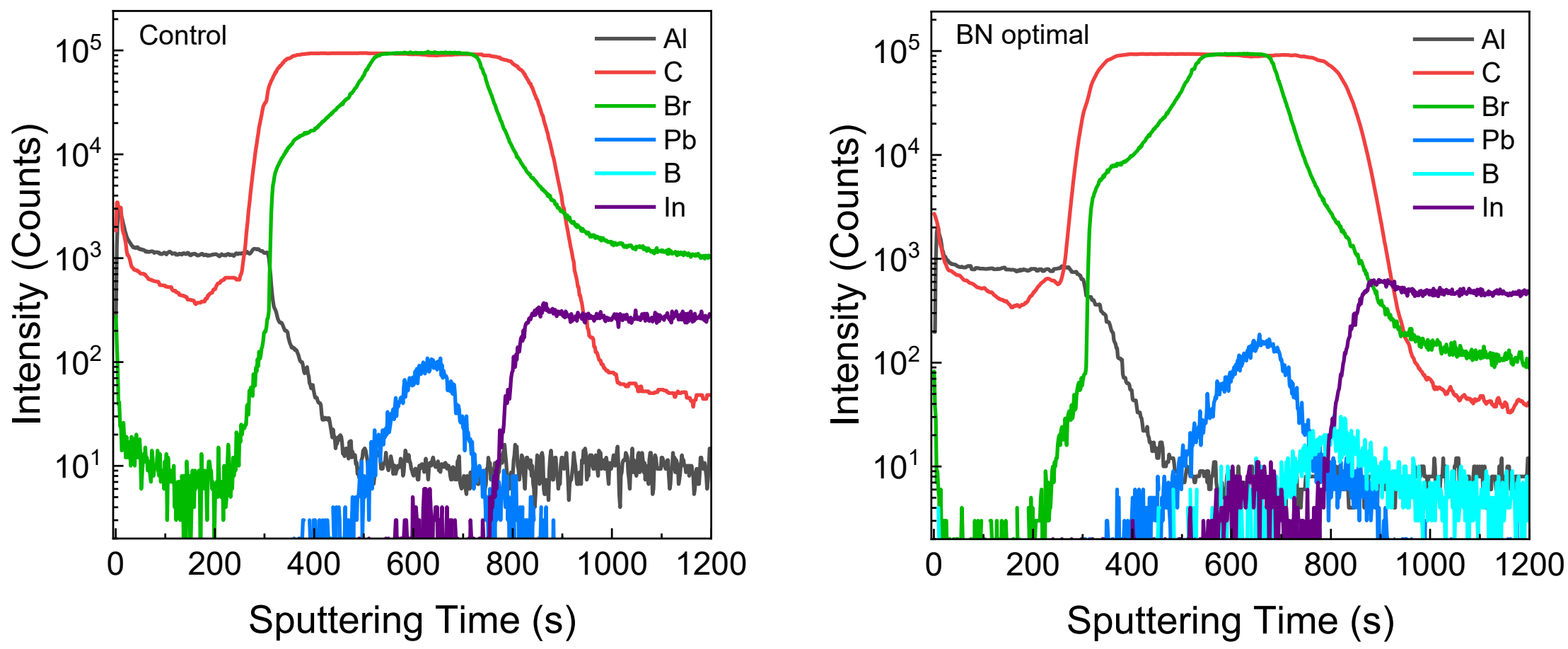

Supplementary Fig. 21 | ToF-SIMS depth profiling of QD devices. The element intensity distribution with sputtering time for ToF-SIMS depth profile of the control and BN-optimized devices.

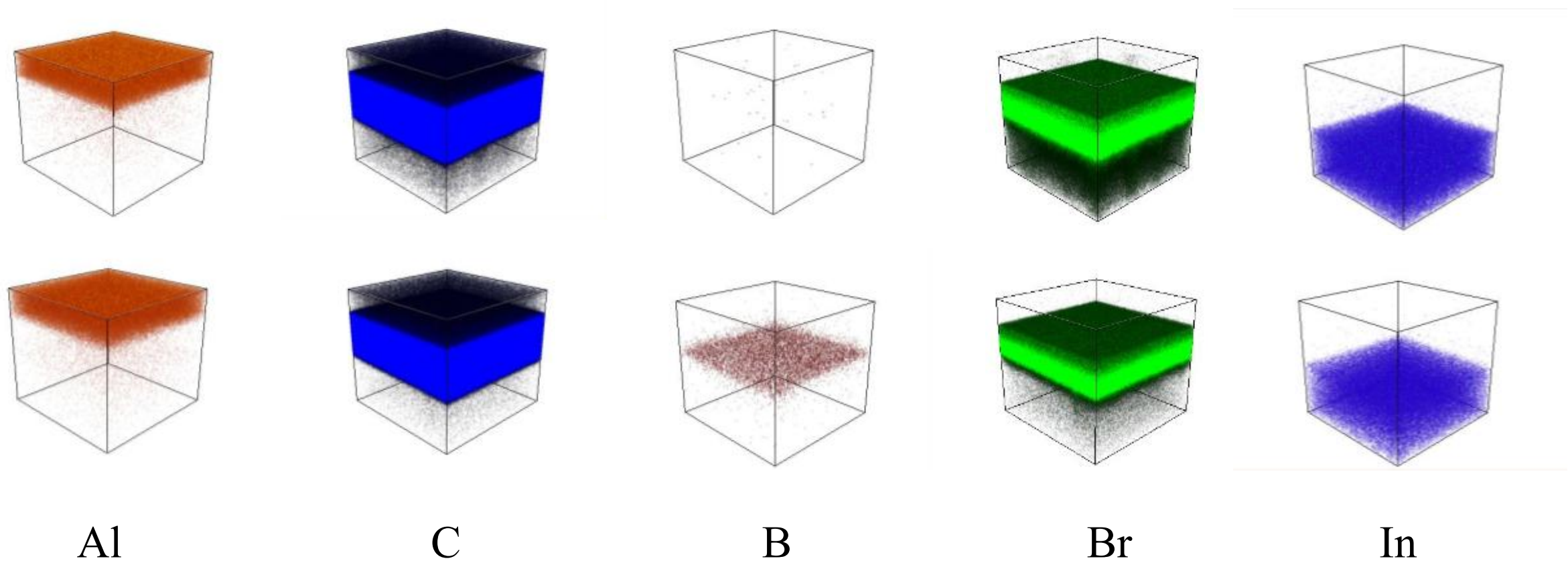

Supplementary Fig. 22 | The corresponding 3D images. The corresponding 3D images for Al, C, B, Br, and In element.

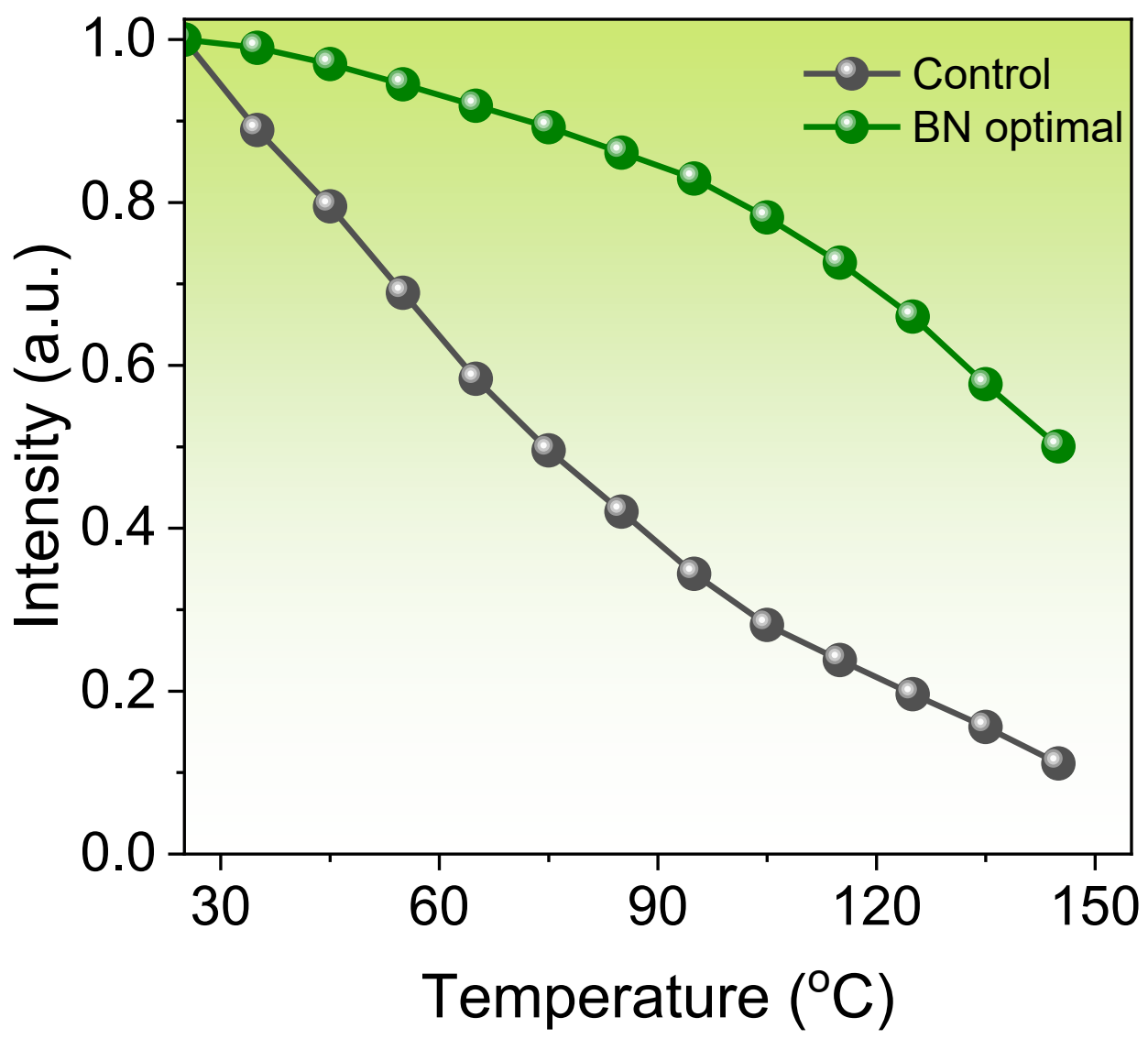

Supplementary Fig. 23 | Temperature-dependent thermal stability. The temperature-dependent stability of QD films before and after BN adsorption.

When heated to 100 °C, BN-optimized QD films could retain 78% of original PL intensity, which is higher than that of only 30% for control QD films, indicating that the BN could improve the exciton recombination stability of QDs under thermal stress.

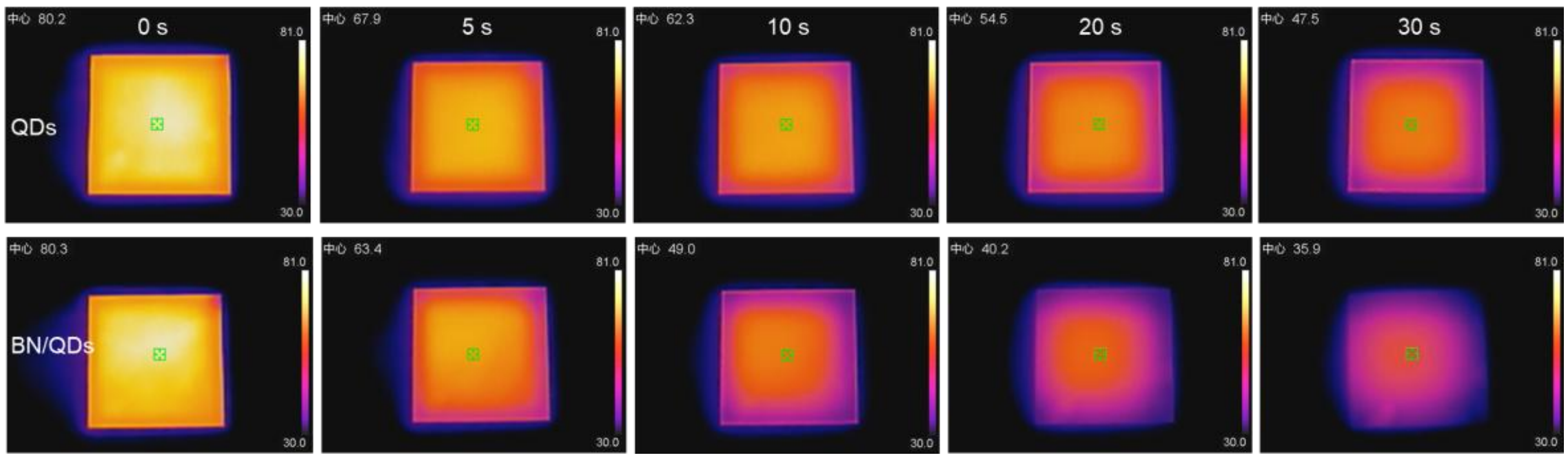

Supplementary Fig. 24 | Time-resolved infrared camera images. Infrared camera images of control and BN-optimized QD films as a function of time in air after heating to 80 °C.

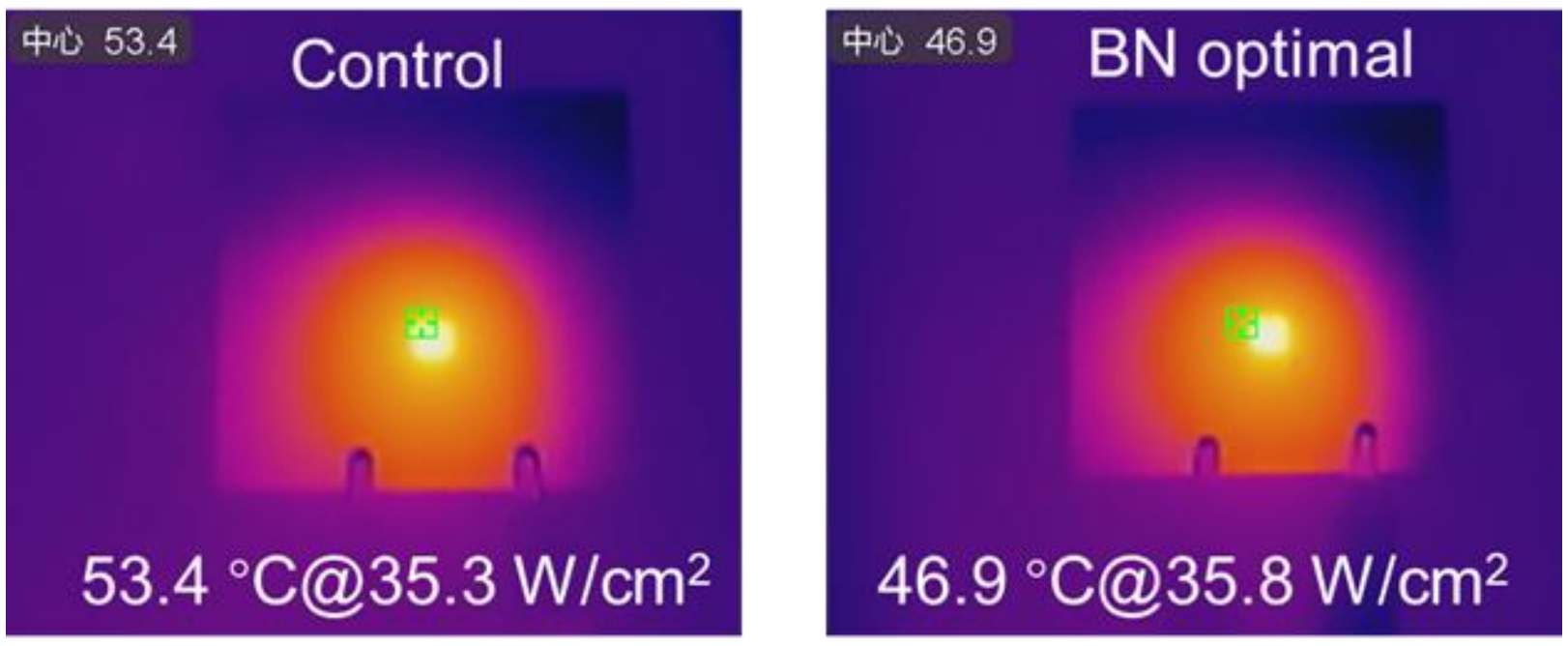

Supplementary Fig. 25 | Thermal infrared imaging of devices under operation. Infrared camera images of control and BN-optimized devices after operation at 35 W/cm^2 for 60 s.

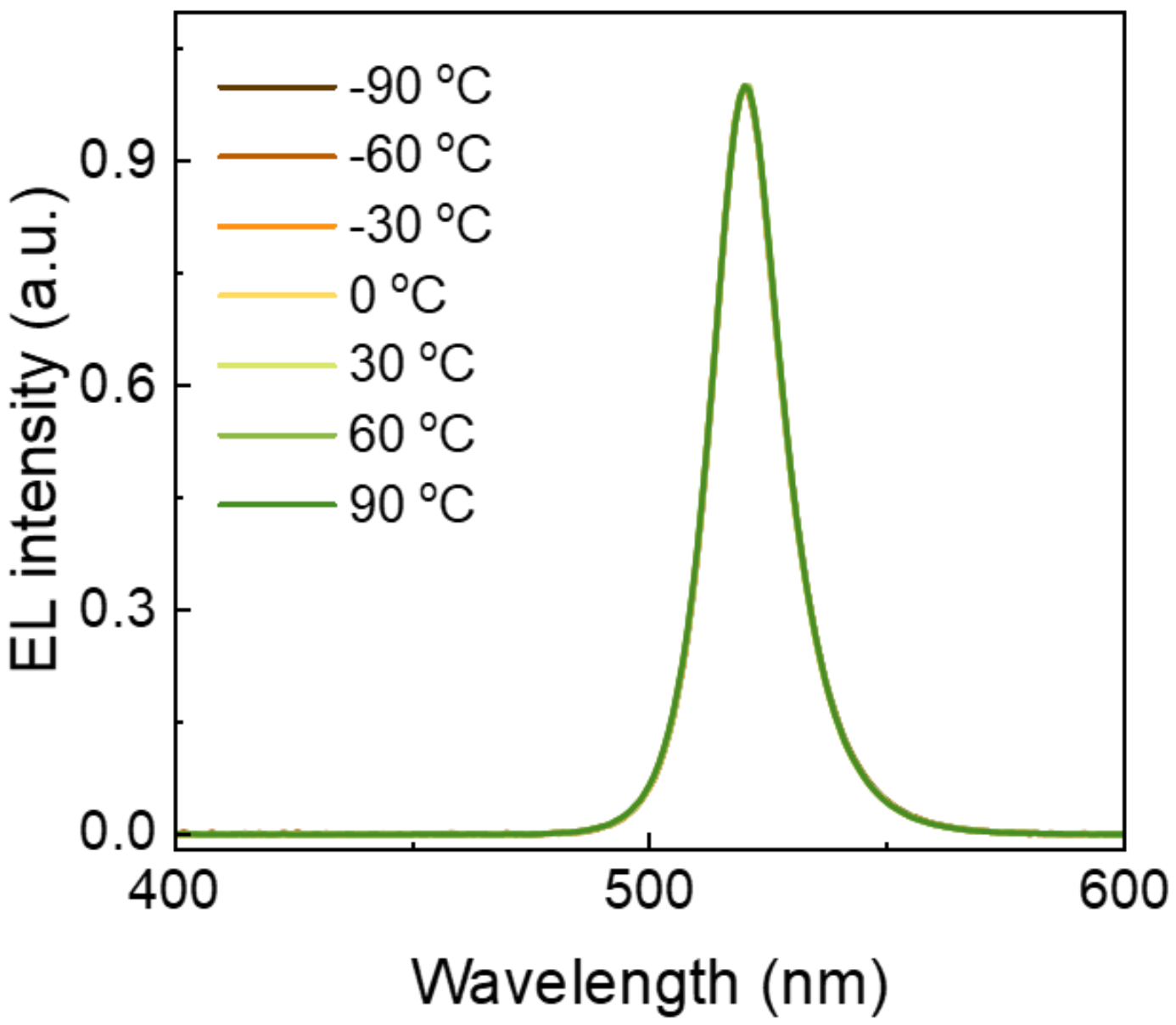

Supplementary Fig. 26 | The angle-dependent EL spectra. The angle-dependent EL spectra for BN-optimized samples.

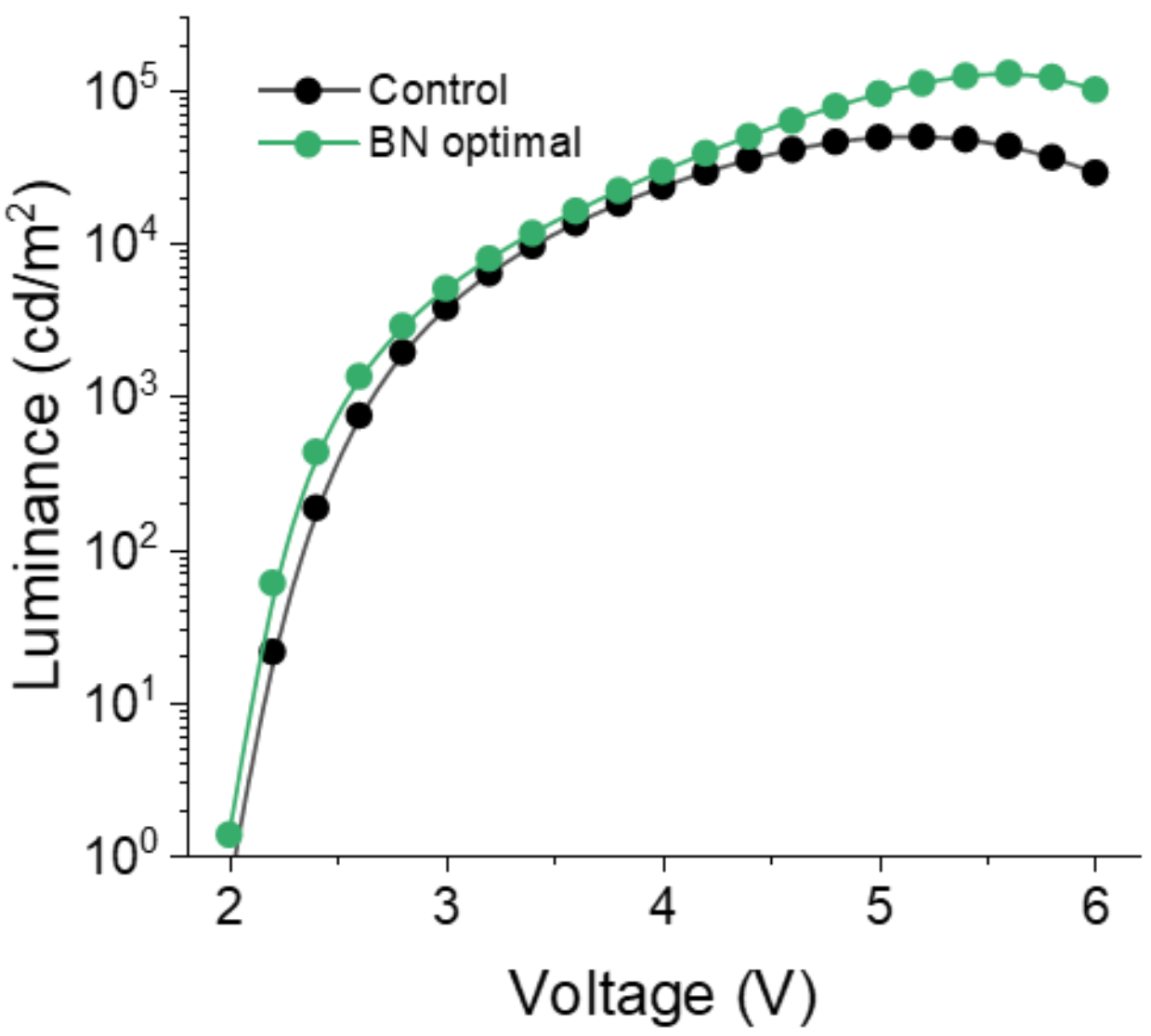

Supplementary Fig. 27 | The luminance data as a function of driving voltage.

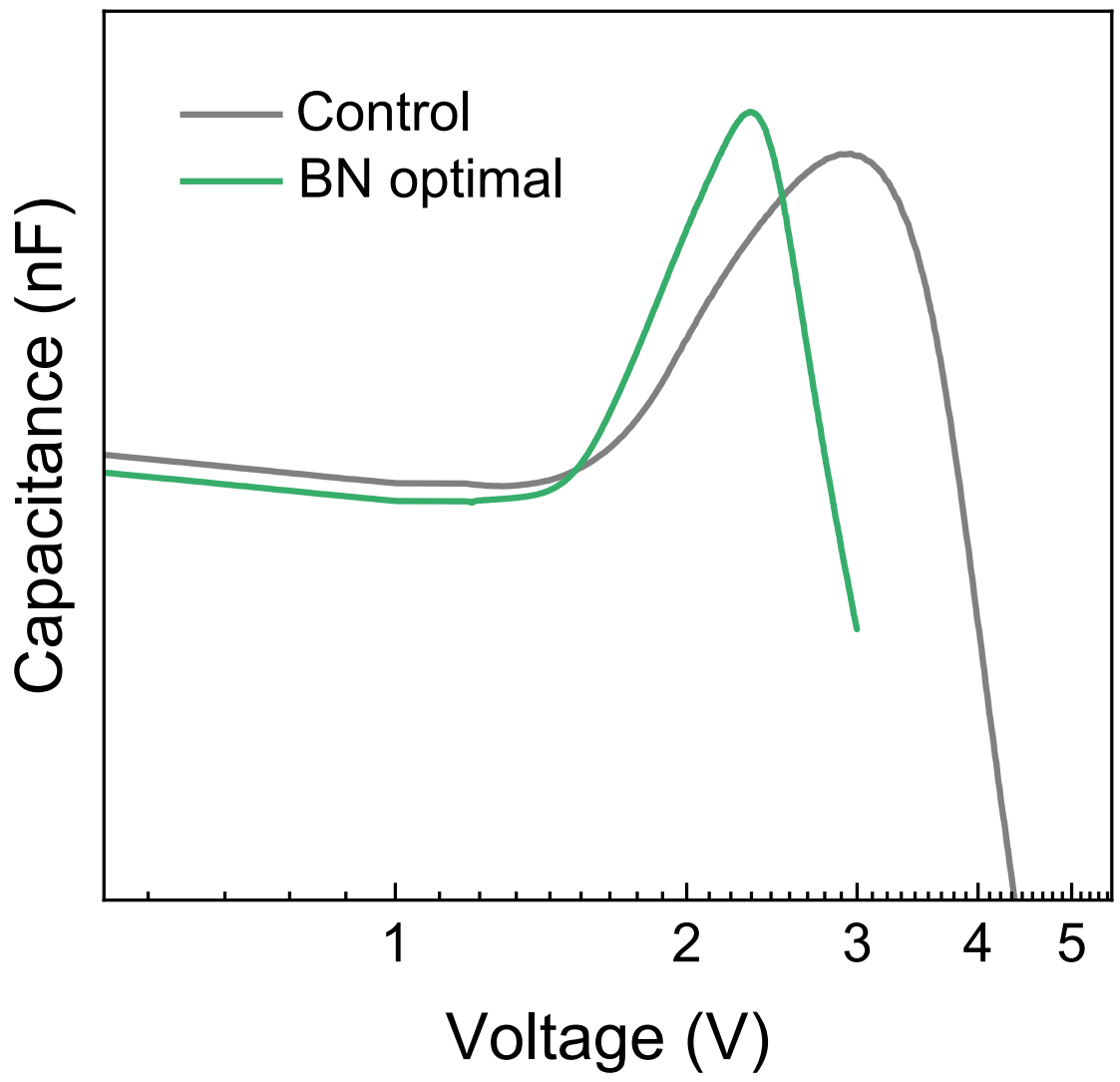

Supplementary Fig. 28 | The capacitance versus voltage (C-V). The capacitance versus voltage of control and BN-optimized devices.

The rapid carrier's injection and accumulation at the interface of carrier transporting layer (CTL) and QDs lead to the increase of capacitance, while the injection of the slow carrier and the recombination of electron-hole pairs at the higher voltage leads to a decrease in capacitance. Therefore, it can be seen from the figure that relative to the control device, the C-V curves shows that BN-optimized LEDs have more efficient electron-hole pair recombination.

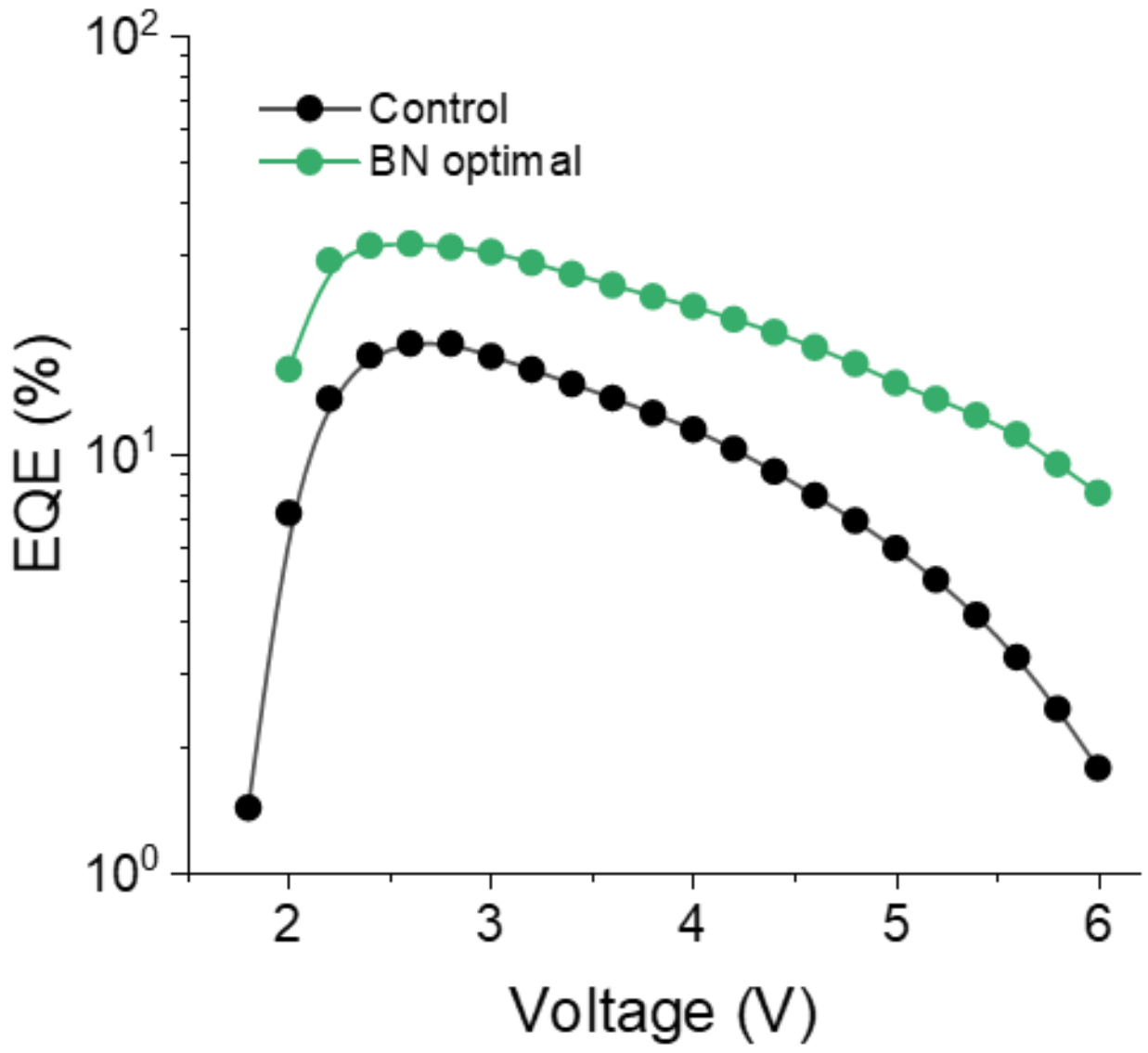

Supplementary Fig. 29 | The EQE data as a function of driving voltage.

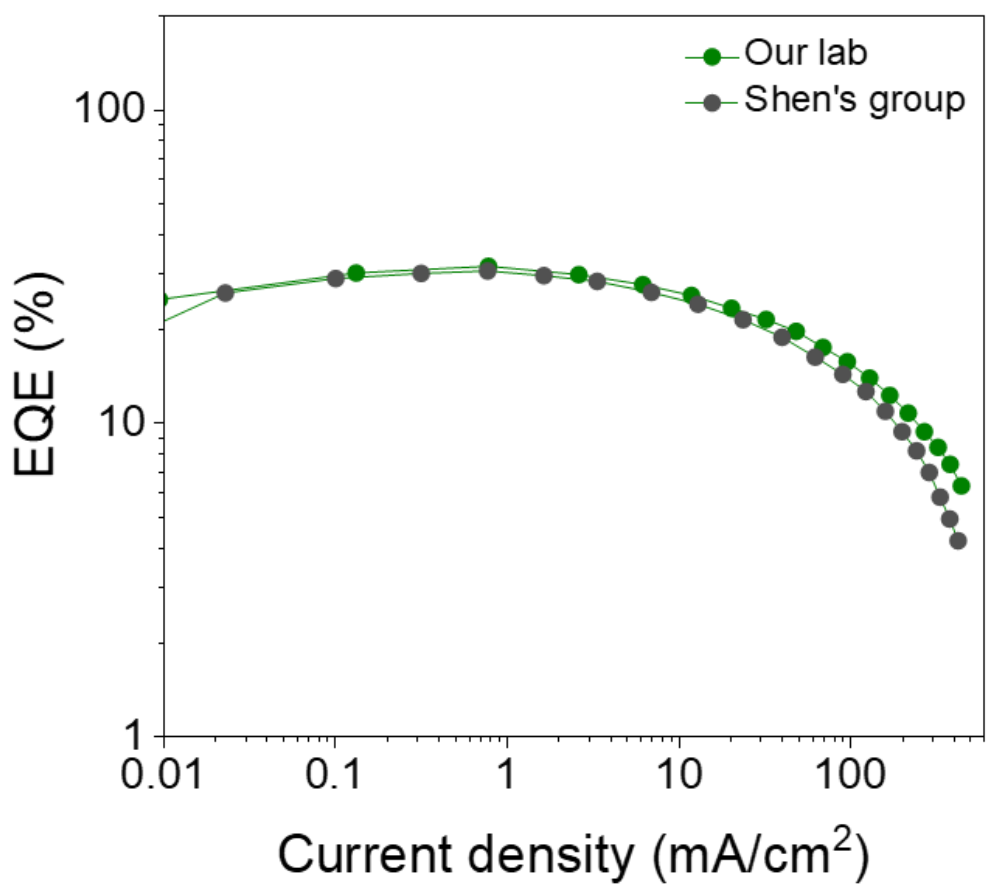

Supplementary Fig. 30 | EQE data verified by external laboratory measurements. The verified peak external quantum efficiency (EQE) of the BN-optimized device is 30.8%. This verification was conducted by the research group of Prof. Shen Huibin, Henan University, employing an Ocean Optics USB2000 spectrometer and a Keithley 2400 source meter.

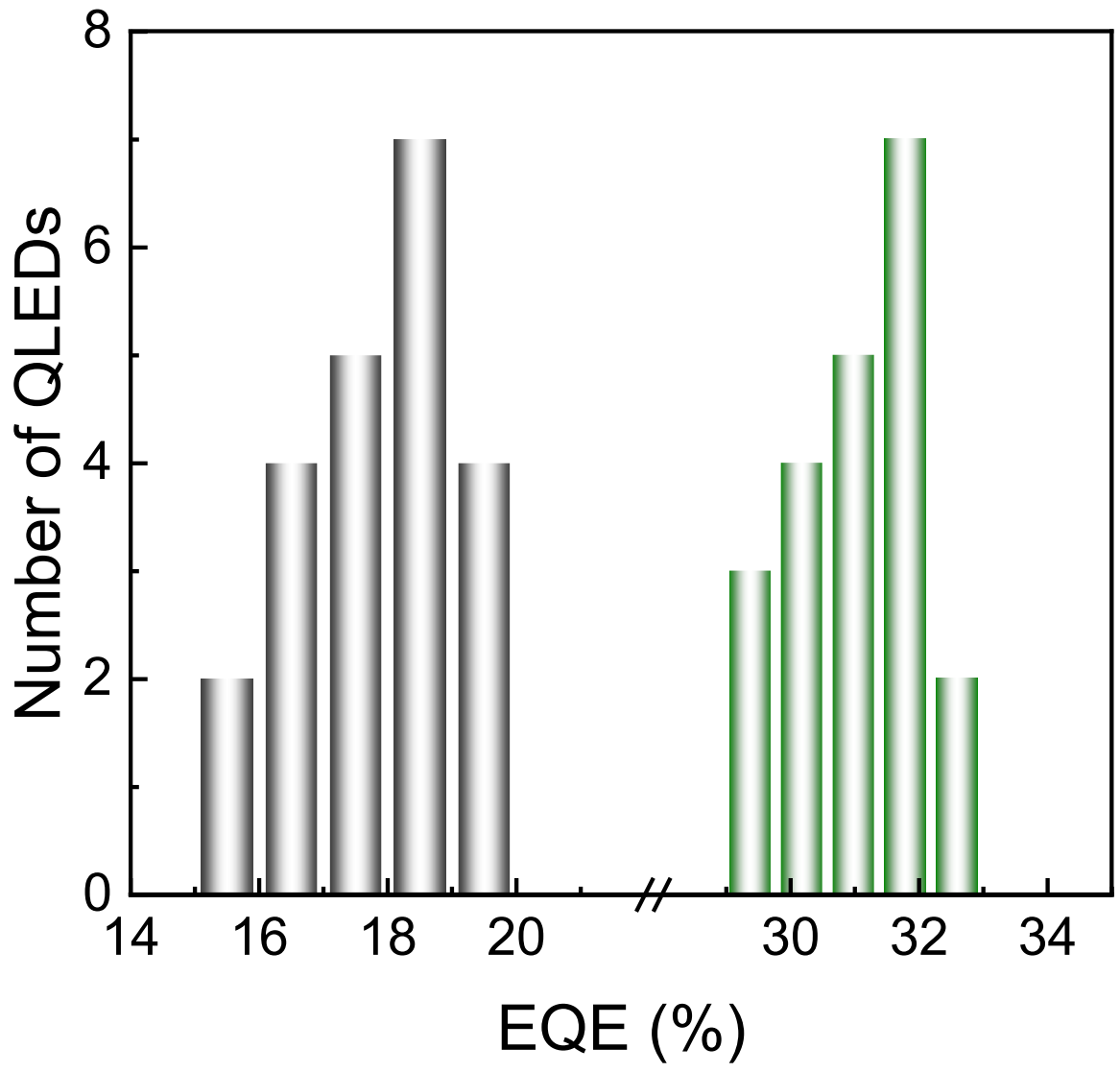

Supplementary Fig. 31 | *EQE*$_{max}$ histogram of QLEDs. *EQE*$_{max}$ histogram of control and BN-optimized QLEDs.

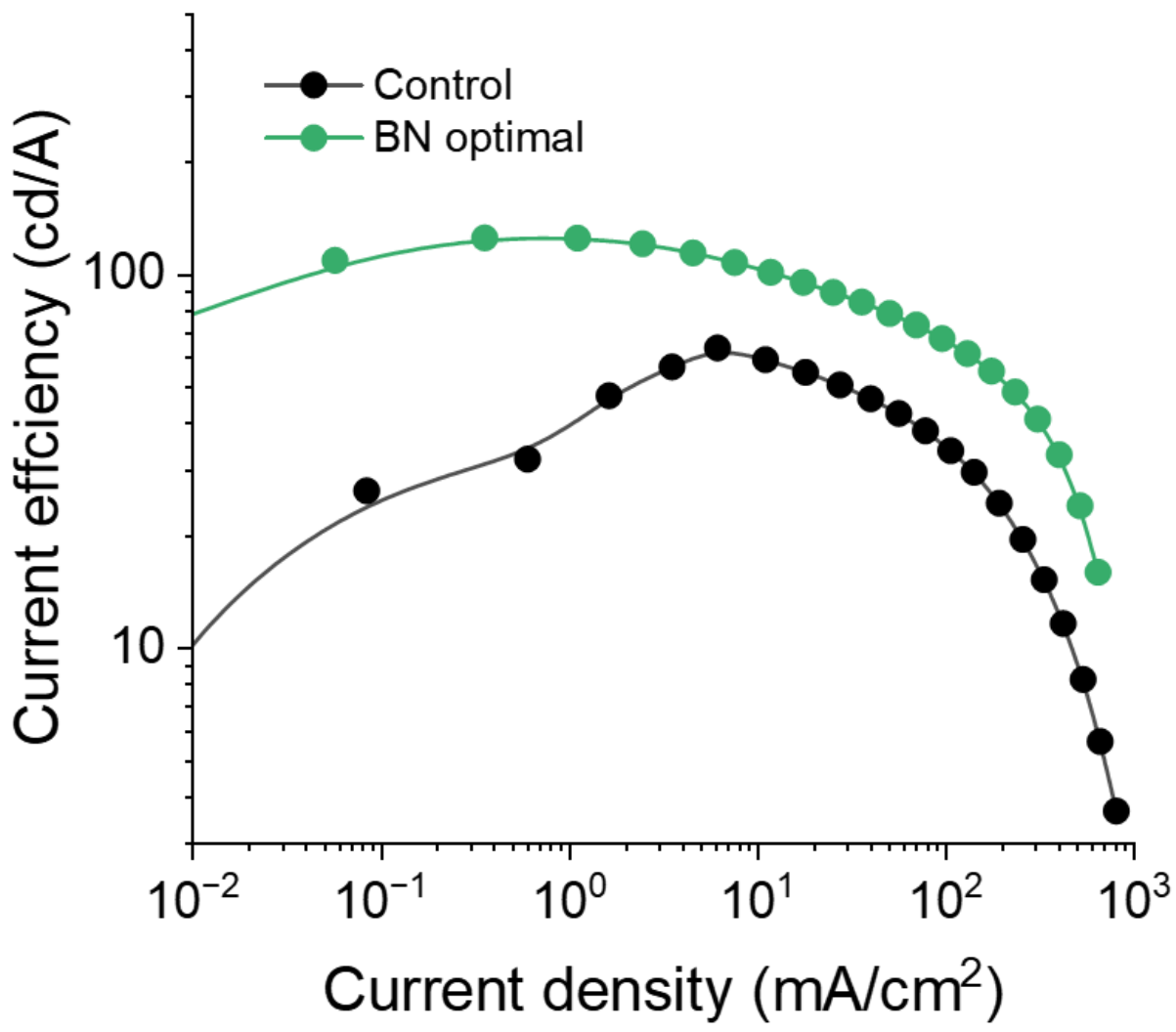

Supplementary Fig. 32 | The current efficiency of control and BN-optimized QLED.

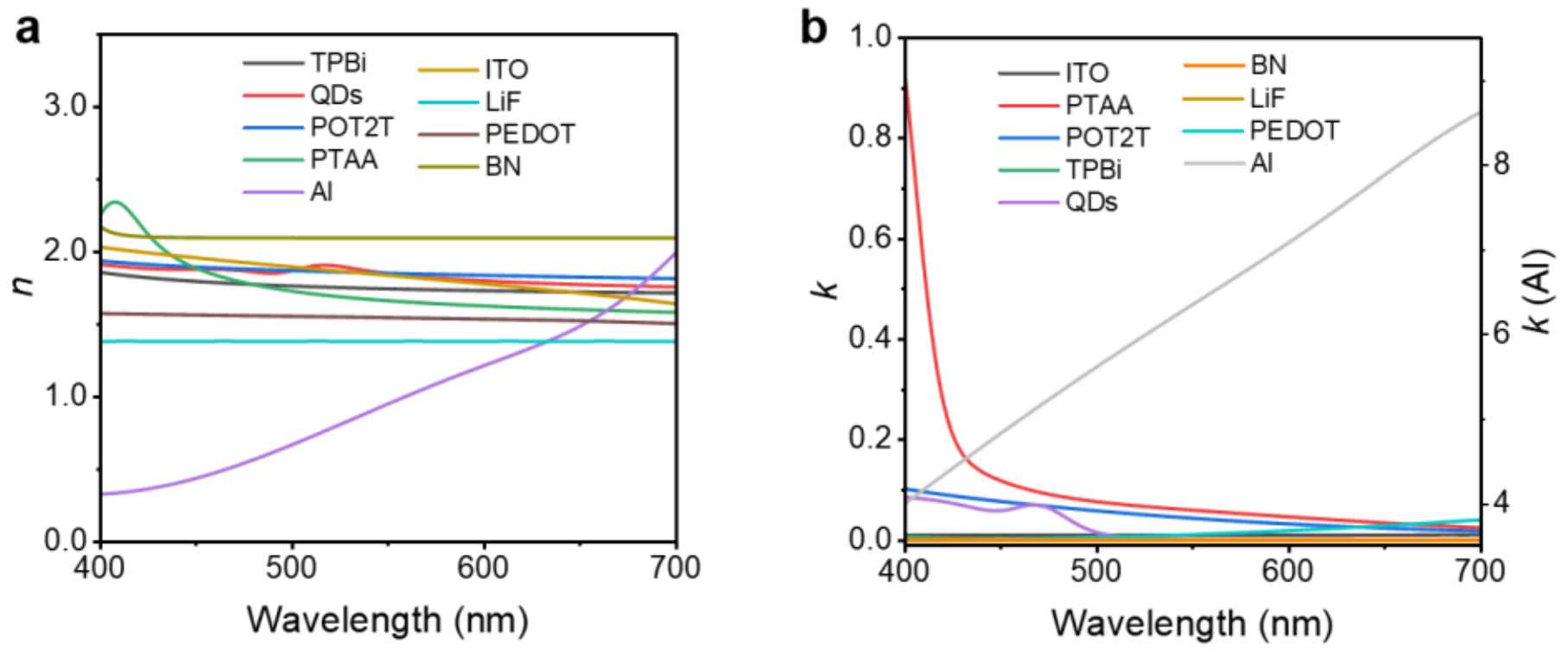

Supplementary Fig. 33 |Refractive indices (*n*) and extinction coefficients (*k*) of all layers in a PeLED. The *n* values in (a) and *k* values in (b) are all extracted by ellipsometer.

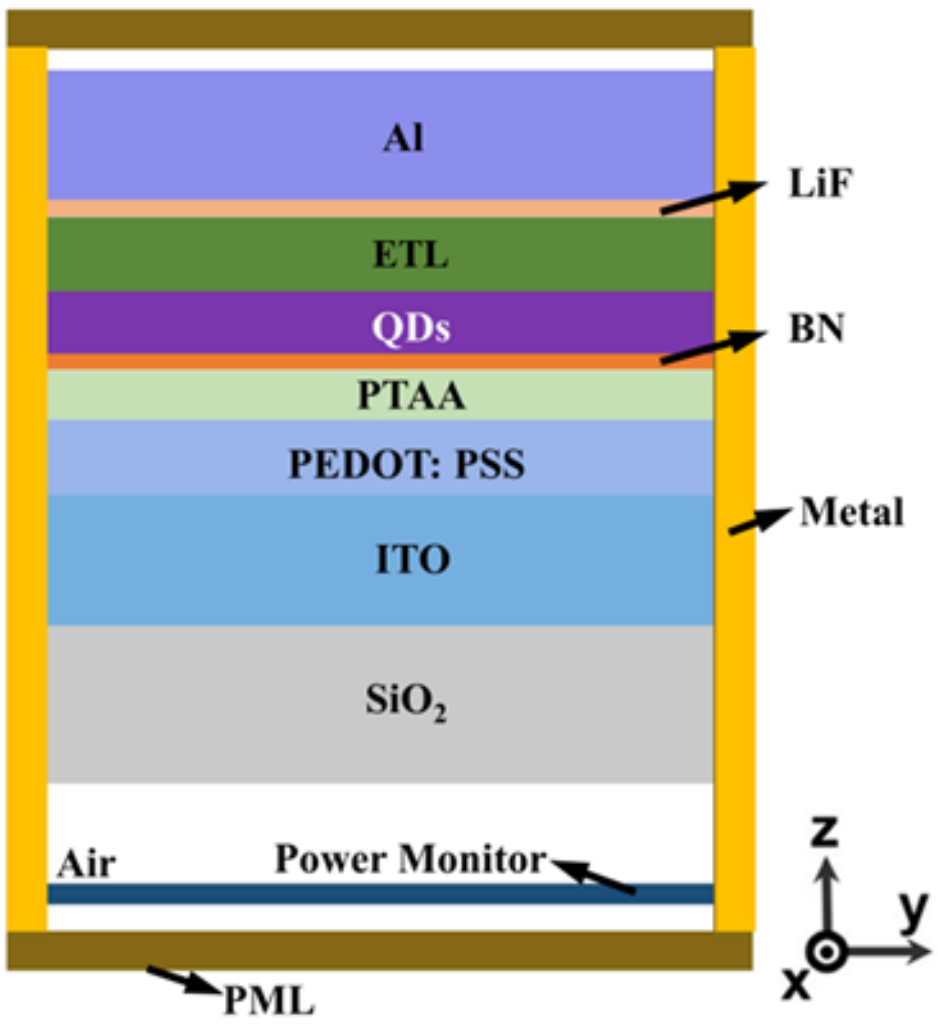

Supplementary Fig. 34 | Schematic Diagram of Simulation Model.

Model setup: The model schematic is shown in the above figure (Supplementary Fig. 32). The lateral dimension of the calculation model is set to 2×2 μm^2. This LED consists of: a 100 nm ITO layer, a 40 nm PEDOT:PSS layer, a 20 nm PTAA layer, a 1 nm BN layer, a 20 nm QDs layer, a 40 nm ETL layer, a 1 nm LiF layer, and a 100 nm Al layer. Additionally, a single-pole emitter representing electron-hole recombination is set in the center of the QDs region. The emission spectrum peak wavelength of this dipole emitter is set at 520 nm. The dipole source is polarized parallel to the X-axis direction to excite the TE mode. The absorption coefficient of the QDs layer at the 520 nm emission wavelength is set to 10000 cm^{-1}. Regarding the material refractive indices, the values of the ITO layer, PEDOT:PSS layer, PTAA layer, BN layer, QDs layer, ETL layer, and LiF layer are 1.85, 1.5098, 1.607, 2.1308, 2.02, 1.727, and 1.3921 respectively. The refractive index and absorption coefficient of the Al material are set based on the built-in Al (Palik) material parameters in the FDTD material library. The absorption coefficients of the other layers are all set to 0.

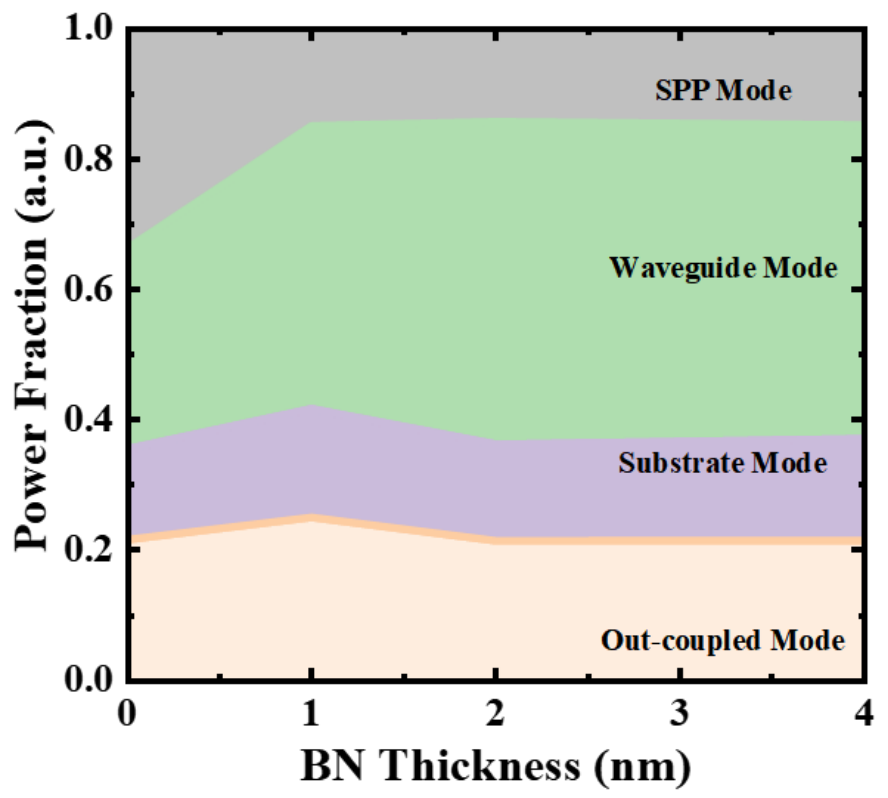

Supplementary Fig. 35 Power fractions of different optical modes in PeLEDs. In BN-optimized QLEDs with a thickness of 1nm, at a wavelength of 520 nm, the substrate mode, waveguide mode, SPP mode, and out coupled mode consume approximately 16.6%, 43.37%, 14.8%, and 25% of photons, respectively. The calculated light-outcoupling efficiency is 35%.

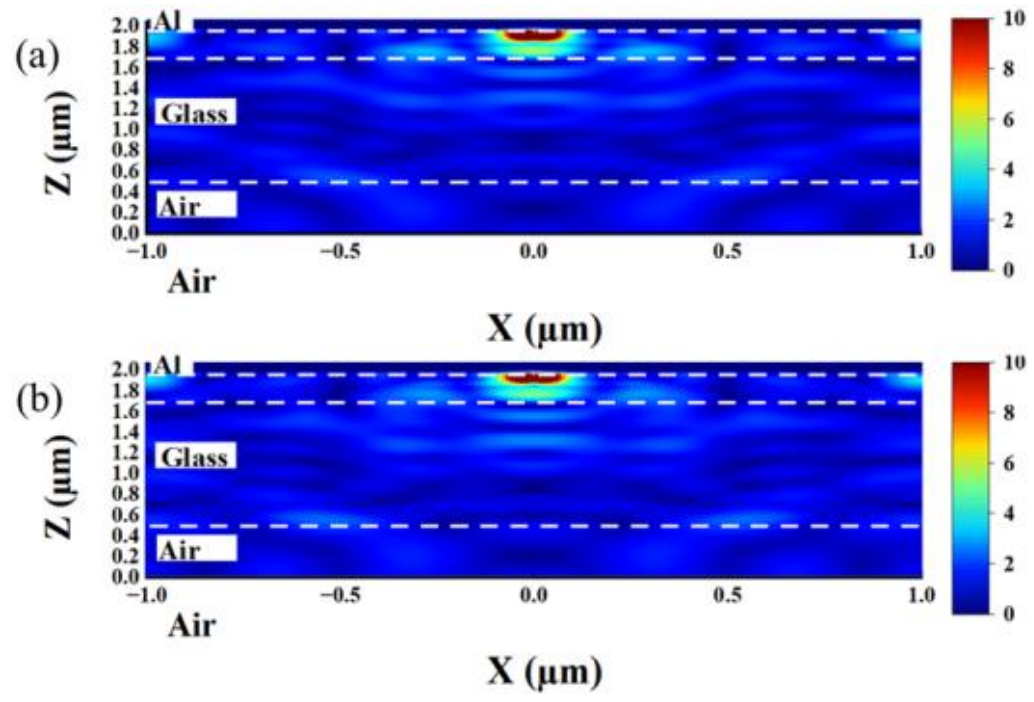

Supplementary Fig. 36 | Electric field distribution and analysis in the XZ cross-section of X-polarized light. (a) 0 nm BN layer. (b) 1 nm BN layer.

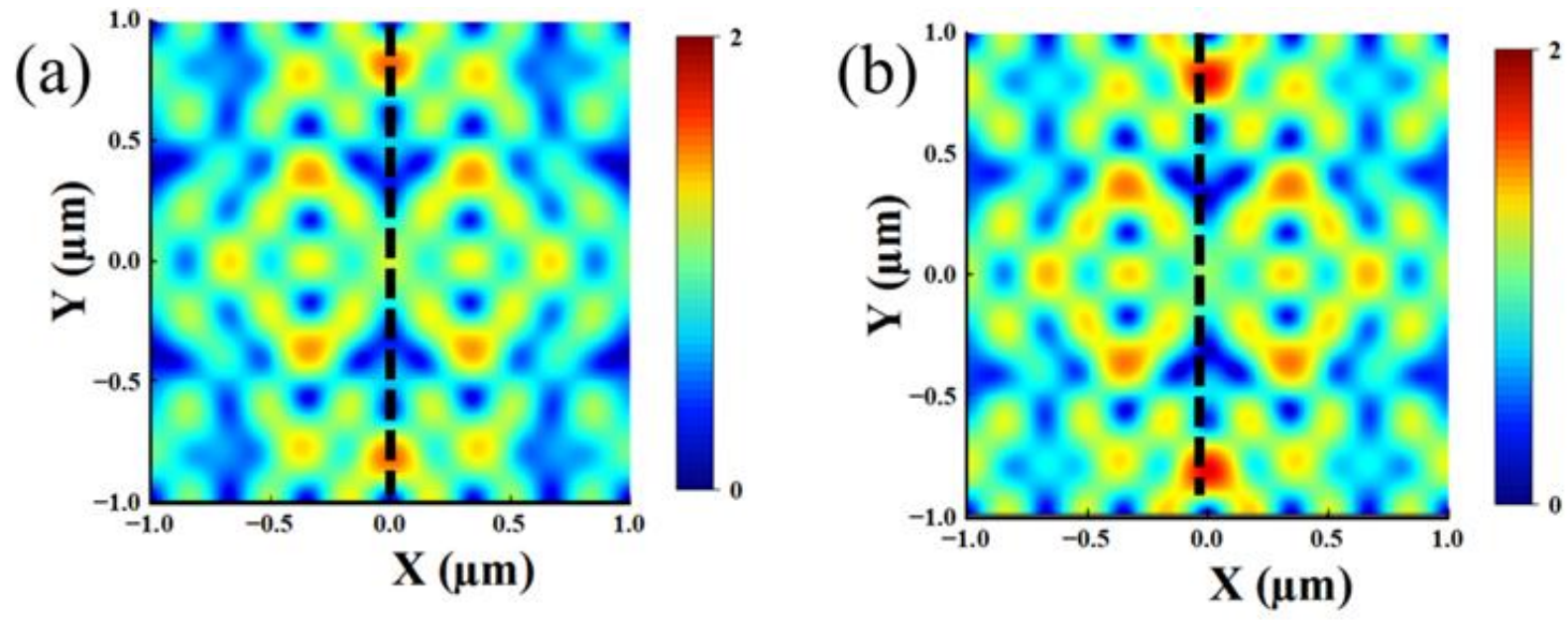

Supplementary Fig. 37 | Electric field distribution and analysis in the XY cross-section of X-polarized light. (a) 0 nm BN layer. (b) 1 nm BN layer.

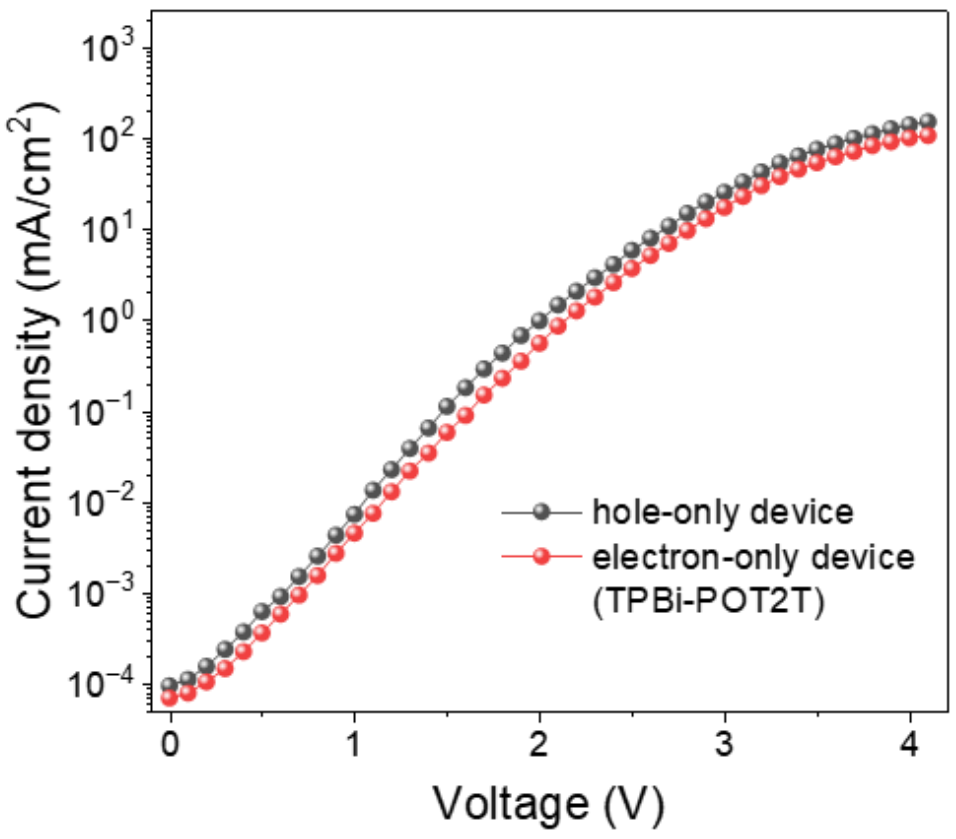

Supplementary Fig. 38 | The electron-only and hole-only devices.

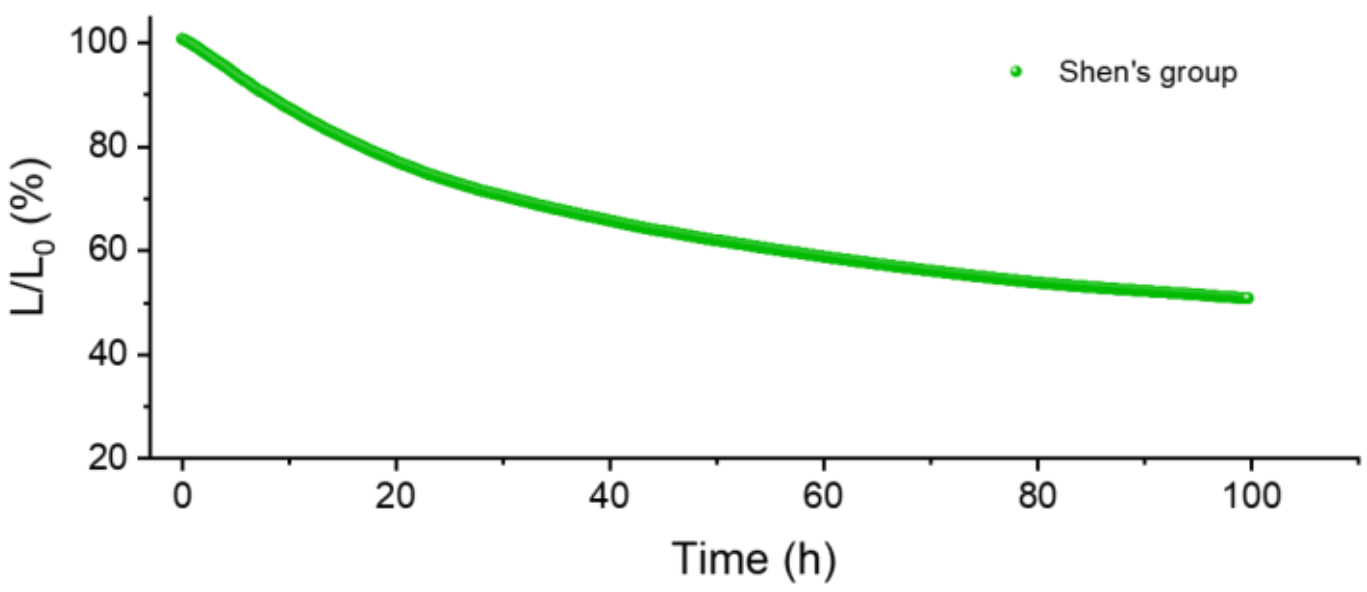

Supplementary Fig. 39 | The operational lifetime of BN-optimized QLEDs from external laboratory measurements. The stability is about 99.7 hours at L_0 of 2000 cd/m^2. This verification was conducted by the research group of Prof. Shen Huibin, Henan University, employing a QD-LED Aging Life Test System (D3000-adv).

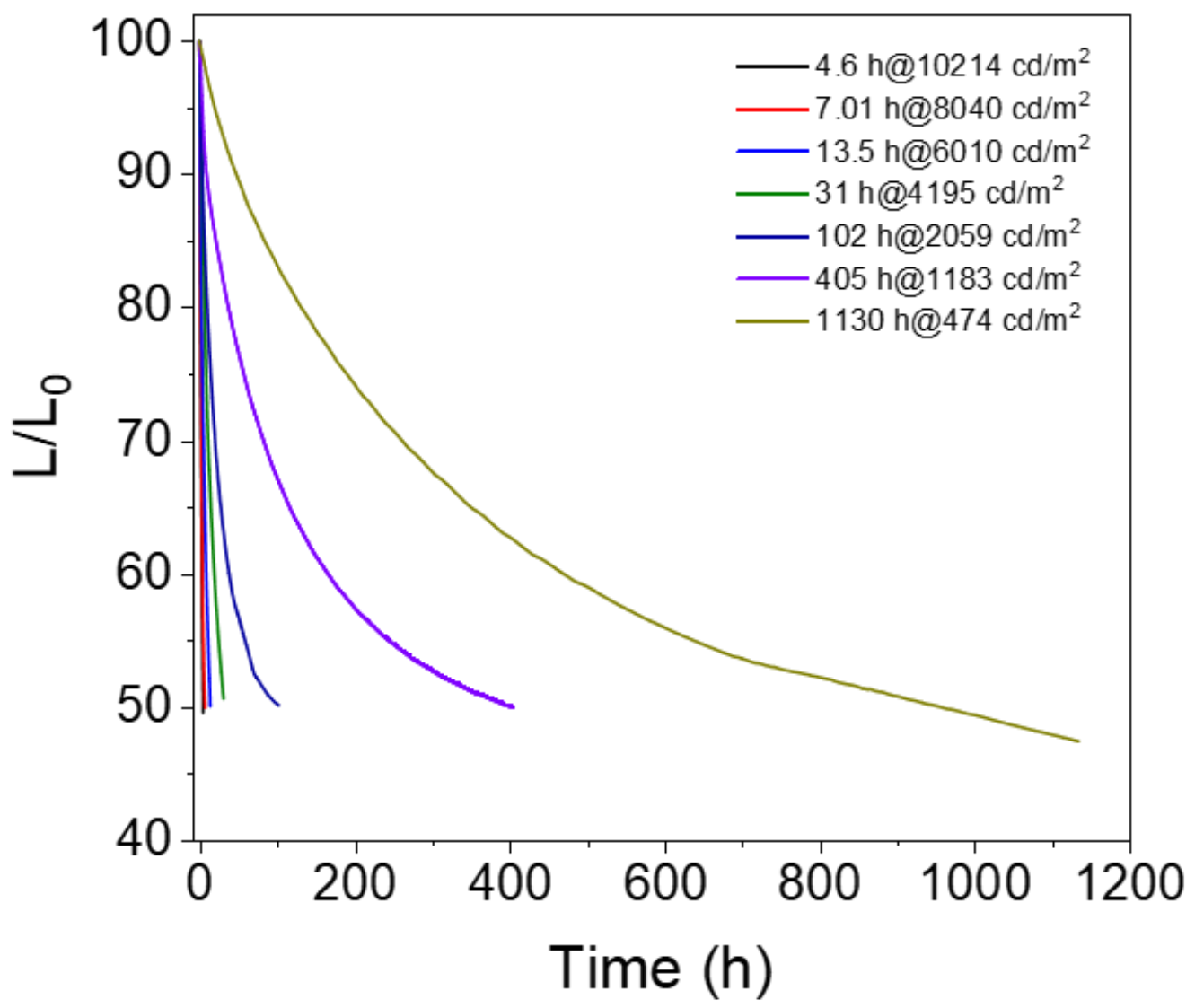

Supplementary Fig. 40 | T_{50} lifetime versus luminance for BN-modified QLEDs. The relationship between T_{50} and luminance in BN-optimized QLED accelerated lifetime experiments under different luminance.

Supplementary Table 1. Summary of PL delay time for QD and BN-optimized QD samples.

Samples	A_1	τ_1 (ns)	A_2	τ_2 (ns)	τ_{avg} (ns)
QDs	3461	4.39	571	26.48	7
BN optimal	1588	9.67	489	49.17	29

The TRPL spectra were fitted with second-order exponential delay:

$$R(t) = A_1 \exp(-t/\tau_1) + A_2 \exp(-t/\tau_2)$$

$$\tau = A_1\tau_1 + A_2\tau_2$$

τ_i and A_i are time components and weights of multiple exponential functions used for analyzing the TRPL delay curves, respectively.

Supplementary Table 2. Refractive indices (*n*) and extinction coefficients (*k*) of all layers in a PeLED.

	ITO	PEDOT:PSS	PTAA	BN	QDs	TPBi	POT2T	LiF	Al
n	1.9	1.56	1.69	2.13	1.9	1.76	1.86	1.39	0.78
k	0.007	0.01	0.05	0.01	0.058	0.0005	0.05	0	7

Supplementary Table 3. Performance parameters of BN-optimized QLED under different current densities.

Luminance (cd/m^2)	Current density (mA/cm^2)	Time (h)
10214	9.7	4.6
8040	7.32	7.01
6010	5.34	13.5
4195	3.42	31
2059	1.66	102
1183	0.81	405
474	0.41	1130

Supplementary Table 4. Comparison of high-performance green perovskite LEDs.

Device structure	EQE_{max} (%)	T_{50} (h)	Ref.
ITO/PEDOT:PSS/Perovskite NCs/TPBi/LiF/Al	26.7	~3[a] (L_0=100 cd/m^2)	*ACS Energy Lett.* 2023, **8**, 927
ITO/Buf-HIL/Perovskite NCs/ZADN/TPBi/BCP:Li/MoO_3/CBP/TAPC/ZADN/LiF/Al)	37	5596[a] (L_0=100 cd/m^2)	*Nat. Nanotechnol.* 2024, **19**, 624
ITO/$Ni_{0.9}Mg_{0.1}O_x$/PVK/PEG/2D Perovskite/TPBi/LiF/Al	30.84	1.68* (L_0=100 cd/m^2)	*Adv. Mater.* 2023, **35**, 2302283
FTO/GraHIL/3D Perovskite @BPA/ZADN/LiF/Al	28.9	31808[a] (L_0=100 cd/m^2)	*Nature* 2022, **611**, 688
ITO/NiO_x/PTAA/PVK/0D(3D) Perovskite/TPBi/LiF/Al	31	-	*Adv. Mater.* 2024, **36**, 2406706
ITO/PEDOT:PSS:PFI/RDPs/3TPYMB/LiF/Al	25.6	1.92* (L_0=7200 cd/m^2)	*Nature* 2021, **599**, 594
ITO/PEDOT:PSS/PTAA/Perovskite NCs/TPBi/PO-T2T/LiF/Al	26.2	10* (L_0=100 cd/m^2)	*Nat Electron* 2024, **7**, 487
ITO/PMMA/PTAA/ Perovskite /POT2T/LiF/Al	28.3	-	*Nat. Mater.* 2025, **24**, 778
FTO/NiOx/PVP/Perovskites/TmPPPyTz/LiF/Al	29.5	18.67* (L_0=12000 cd/m^2)	*Nat. Photon.* 2024, **18**, 363
ITO/Me-4PACz/PVK/Perovskite/ETL/LiF/Al	22	185,600[a] (L_0=100 cd/m^2)	*Nature* 2025, **643**, 96
FTO/GraHIL/Perovskite NCs/ZADN/LiF/Al	28.9	520* (L_0=1000 cd/m^2)	*Nature* 2022, **611**, 688
ITO/PEDOT:PSS/PTAA/Perovskite QDs/TPBi/LiF/Al	18.6	213[a] (L_0=100 cd/m^2)	*Adv. Funct. Mater.* 2024, **34**, 2308341
ITO/PEDOT:PSS/PTAA/Perovskite QDs/TPBi/PO-T2T/LiF/Al	21.63	180.1* (L_0=100 cd/m^2)	*Sci. Bull.* 2021, **66**, 36
ITO/PEDOT:PSS/PTAA/Perovskite QDs/3P/LiF/Al	30.2	3220[a] (L_0=100 cd/m^2)	*Angew. Chem. Int. Ed.* 2024, **63**, 202410689
ITO/PEDOT:PSS/PTAA/Perovskite QDs/TPBi/PO-T2T/LiF/Al	20.3	127* (L_0=100 cd/m^2)	*Adv. Optical Mater.* 2024, **12**, 2302253
ITO/PEDOT:PSS/Poly-TPD/Perovskite QDs/TPBi/LiF/Al	23.45	0.02* (L_0=100 cd/m^2)	*ACS Energy Lett.* 2023, **8**, 4386
ITO/PEDOT:PSS/Buf-HIL/$FA_{1-x}GA_xPbBr_3$/TPBi/LiF/Al	23.4	2.2* (L_0=100 cd/m^2)	*Nat. Photonics* 2021, **15**, 148
ITO/PEDOT:PSS/PTAA/$CsPbBr_3$ QDs/TPBi	24.7	182[a] (L_0=100 cd/m^2)	*Appl. Phys. Rev.* 2024, **11**, 031405
**ITO/PEDOT:PSS/PTAA/BN/Perovskite QDs/ETL/LiF/Al**	**32**	**25263**[a] (L_0=100 cd/m^2)	**This work**

Notes, * represents the real measured lifetime, and [a] represents the extrapolated lifetime.
