# Supplementary material for: Ultrathin nano-layered boron nitride stabilizing perovskite quantum dot-based light-emitting diodes
Source: Nat Commun. 2026 Sep 1;17:10415. doi: 10.1038/s41467-026-77166-z (PMC13631234; doi:10.1038/s41467-026-77166-z)
Supplement: Supplementary file 2 [file 41467_2026_77166_MOESM2_ESM.pdf]

nature portfolio

Peer Review File

Ultrathin nano-layered boron nitride stabilizing perovskite quantum dot-based light-emitting diodes

Corresponding Author: Professor Jizhong Song

This file contains all reviewer reports in order by version, followed by all author rebuttals in order by version.

Version 0:

Reviewer comments:

Reviewer #1

(Remarks to the Author)
Wang co-authors have presented a study on enhancing the stability of perovskite quantum dot LEDs by incorporating ultrathin nano-layered boron nitride (BN) as an ion migration blocking layer. The BN-based devices show maximum EQE of 32%, improved stability over the control devices. While the EQE claimed by the authors is amongst some of the highest efficiencies for perovskite LEDs, the device stability claims are not well supported by evidence. Several major issues need to be resolved.

1. Judged by its introduction, the motivation of the paper is rather unclear. The authors say "Perovskite quantum dots (QDs) have attracted increasing attention in the field of light-emitting diode (LEDs) due to the high photoluminescence quantum yield (PLQY), tunable emission peaks (400-700 nm), narrow full width at half maximum (FWHM, ~20 nm). Moreover, significant breakthroughs have been achieved in the external quantum efficiency (EQE) (>25%) of perovskite quantum dot light-emitting diodes (QLEDs) in recent years." In fact, perovskite LEDs in general (not limited to perovskite QD based LEDs) can easily achieve or outperform these properties. So the question is then: why do we need to use quantum dots? The authors should provide further justification.

2. In Figure 5h, the authors show the data points: about 400 h at 1000 cd/m2, >1000 h at around 500 cd/m2. The authors did not say whether these points are from data extrapolation, so we could assume that these are from their experiments. The authors should provide the actual luminance decay curves similar to Figure 5g, with current densities specified. The authors must clarify whether data points are extrapolated (in text in figures).

3. The claim that BN effectively inhibits ion migration is not fully supported by mechanistic evidence. Alternative explanations are not discussed. The authors should provide more evidence to support the ion migration inhibition claim discuss alternative explanations.

4. The heat dissipation mechanism enabled by BN is not quantified or thoroughly discussed.

5. The improvements in EQE lifetime are mainly compared to the control devices. Comparisons to other types of high-performance perovskite LEDs (not limited to $CsPbBr_3$ QD devices) are not thorough enough.

Reviewer #2

(Remarks to the Author)
In this manuscript, the authors introduced a BN interlayer into the Pe-QLEDs configuration, which demonstrated a high EQE with enhanced stability. While the study presents a valuable mechanistic insight, the overall performance enhancement, particularly in terms of device operational stability, remains inferior to that of traditional QDs based LEDs (such as CdSe based QLEDs) even perovskite film based LEDs. In addition, this work does not reveal new scientific phenomena/solve major scientific problems, its contribution to the field is limited. The absence of explanations on the underlying mechanisms also makes it hard for the reviewer to suggest its publication in high impact Nature Communications. The followings are some questions to help improve the quality of this manuscript.

1. The analysis on the interactions between BN perovskite should be clarified. The authors claimed that the BN adsorbed on

the perovskites had a strong passivation. What's difference correlation between the mentioned adsorption interaction the passivation? Do they actually refer to the same adsorption interaction?
2. Besides, when the authors claimed "This interaction serves to stabilize the perovskite structure, prevent surface defects…… Such interactions are further confirmed by the arrangement of QDs" in the manuscript, the refer of "this interaction" "Such interactions" should be more specific.
3. The authors claimed "…the chemical interaction between BN perovskites." What is the chemical interaction? Is it the abovementioned adsorption? If not, what it is what the difference relation with abovementioned adsorption?
4. How can the BN promote exciton recombination behavior? This needs to be explained in more details.
5. How can the reduced electron trap density of BN-optimized QD films can indicates the improvement of the stability perovskite QDs? Please explained in more details.
6. Since the introduction of BN can enhance the activation energy (Ea) of ion migration, how does the thickness of BN interlayer influence the PL performance of QDs the performance of LEDs? Is there an optimal thickness of the BN interlayer?
7. Although the BN can facilitate the dissipation of heat in perovskite films, the evidences for reduced Joule heating in operating QLEDs with BN are suggested to be provided?
8. Some typos should be corrected, e.g. "paterns" should be "patterns".

Reviewer #3

(Remarks to the Author)
The authors propose a novel use of ultrathin nano-layered boron nitride (BN) as an ion-migration barrier defect passivation layer in perovskite LEDs. This interface engineering significantly enhances both efficiency operational stability. They report a notably high EQE of 32% a record operational lifetime (T50) of 25,263 hours. While these results are impressive, the reliability reproducibility of the data need to be further verified . Below are detailed comments necessary clarifications:

1. Device Performance: EQE Measurement effect of BN
i. The reported EQE of 32% is exceptionally high for perovskite quantum dot LEDs without any light outcoupling enhancements, such as scattering films, or photon recycling structures. The authors should: 1) clarify whether any structural or optical enhancement was used, 2) provide optical simulations to support such high efficiency.
ii. As shown in Supplementary Figure 2b, BN exhibits a certain scattering effect in EtOH possesses a nanometer-scale fly-eye lens morphology, which is expected to enhance the outcoupling efficiency. The authors should measure the scattering effect of the BN layer using a haze meter compare the degree of scattering, device performance, EL spectrum as a function of the BN solution thickness weight percentage.
iii. The EQE should be validated using an integrating sphere setup independently verified by a well-established external laboratory to rule out measurement artifacts.
iv. Details of the EQE measurement procedure, including detector calibration measurement conditions (e.g., device orientation, encapsulation), must be clearly described.
v. The manuscript should include current efficiency (cd/A) data to correlate with EQE confirm the radiance output.
2. Device Performance: Lifetime Measurement
i. The manuscript claims that the Minolta CS-2000 was used for lifetime measurements. However, this spectroradiometer is not typically used for extended lifetime testing. Please explain in detail how this equipment was used to monitor degradation over thousands of hours.
ii. The authors must specify the test conditions, including ambient temperature, humidity, atmosphere (air or N_2), drive mode (constant current vs. constant voltage), measurement intervals.
iii. Complete lifetime curves (not just extrapolated values) from multiple samples should be included in the supplementary materials. In particular, the raw data for all points contributing to the extrapolated trend in Fig. 5h must be shown.
3. The experimental section lacks sufficient detail for reproducibility. The following information should be provided:
i. The thicknesses of all functional layers (e.g., ITO, PEDOT:PSS, PTAA, BN, QD layer, TPBi, Al), the preparation method of the BN solution, the molecular weight of PTAA, other relevant details must be explicitly stated.
ii. The deposition method for the QD layer (e.g., spin-coating parameters such as speed time), annealing conditions (temperature duration), precursor concentrations, any post-treatments (e.g., ligand exchange, solvent annealing) must be described.
4. The HOMO/LUMO (or valence/conduction band) energy levels of each layer should be clearly labeled in Fig. 1d. Additionally, UPS measurements should be conducted for QD films with without BN interlayers to verify band alignment changes due to BN passivation.
5. Crystallographic planes such as (100) (110) shown in GIWAXS (Fig. 2d) should be explicitly labeled in the figure supported by simulations or XRD data.
6. In the temperature-dependent conductivity test (Supplementary Fig. 14), the schematic shows Au deposited on top of the QD layer. If BN is located beneath the QDs, it is unclear how it serves as an effective ion migration barrier. This experimental configuration requires clarification.
7. The methodology for preparing the PeLED samples for ToF-SIMS depth profiling is missing. Specifically, it should be explained how the two different PeLED samples were electrically aged before analysis.
8. The claim that the ultrathin BN film significantly improves heat dissipation is questionable. Although BN has high intrinsic thermal conductivity, its effectiveness as a nanometer-scale film on a millimeter-thick glass substrate is likely negligible. The authors should provide thermal simulations or compare performance with a non-BN control under identical encapsulation conditions to substantiate this claim.
9. Please provide luminance EQE data as a function of driving voltage. For the BN-optimized device, data at a luminance level of 1 nit must be included to evaluate turn-on voltage.
10. The device exhibits strong sub-bandgap electroluminescence (EL) below 2.0 V, despite the perovskite QD bandgap

being approximately 2.4 eV. Such behavior is typically attributed to extremely thin or high-mobility transport layers. However, TPBi is known to have relatively low electron mobility. The authors should clarify the origin of this sub-bandgap emission. To determine whether this sub-bandgap emission originates from ultra-fast charge injection from the charge transport layers, the authors are encouraged to provide J–V curves of a control bipolar device composed only of PTAA TPBi (without the QD layer). Additionally, device performance data using various HTL ETL materials (such as PTAA with different molecular weights, Poly-TPD, PVK for HTLs, PO-T2T, TmPyPB, etc. for ETLs) should be included

Version 1:

Reviewer comments:

Reviewer #1

(Remarks to the Author)
The authors have revised the paper reasonably well by addressing the issues I raised in the last round of review. However, I believe that some of the key points are still not resolved, particularly in relation to the reliability of device lifetimes. Currently the data reporting description of experimental methods are vague, raising important concerns on the validity of the main conclusions.

1. The authors should make it very clear that the lifetime of 25263 hours at 100 cd/m2 was extrapolated in various places in the paper, including abstract, introduction, conclusion, Fig. 5h Supplementary Table 3. This is to avoid potential misleading claims.

2. According to Fig. 5g, the luminance of the sample “BN optimal” degraded more rapidly in the beginning then slowed down after about 80 hours. What happened after 80 hours? Did the device more rapidly degrade or it lasted for much longer? The degradation curve of BN devices seems to be much smoother compared to the “control”. Can the authors show the data points rather than a smooth curve?

3. In response to Reviewer #3’s comments, the authors say:

“The LED was driven at a constant current density of 1.66 mA/cm2 corresponding to an initial luminance (L0) of ~2000 cd/m2 as determined from the I-V-L characteristics. The luminance (Lt) was monitored in real time using a CS2000 spectroradiometer, while a 2602B source meter maintained the constant current output. The device lifetime (T50) was defined as the elapsed time (t) until the luminance decayed to 50% of L0 (i.e., 1000 cd/m2). All data were recorded automatically via software. It is also worth mentioning that our laboratory has two I-V-L life testing instruments, which are measurement devices designed by Hangzhou Yuanfang Instrument Co., Ltd. Suzhou Fushida Scientific Instruments Co., Ltd.. They can meet the requirements for longterm testing without affecting the overall progress of the laboratory.”

It should be noted that the authors basically mentioned two different systems for lifetime measurements. In the methods section, they only mention the CS2000 spectrometer which was alerted by reviewer #3 on whether this system is suitable for measuring long device lifetimes. Later in their response, the authors mention that they also use experimental setups designed by Hangzhou Yuanfang Instrument Co., Ltd. Suzhou Fushida Scientific Instruments Co., Ltd. However, details of these are completely missing in the paper.

So, which of the two systems were actually used for the lifetime experiments? What were the differences between the two systems (which one is more reliable for what purposes)? Please be specific so that the readers can see which figure was obtained using which setup. Such details must be provided clearly in the paper.

Reviewer #2

(Remarks to the Author)
In this manuscript, the authors introduced a BN interlayer into the perovskite QLED configuration, which resulted in a high EQE exceeding 30%. However, it should be noted that the authors previously reported an EQE as high as 30.2% using the same type of quantum dots (Angew. Chem. Int. Ed. 2024, 63, e202410689). Especially, looking carefully into the response letter revised manuscript, I found most of the conclusions to be farfetched. The EQE of over 30% claimed by the authors is also not convinced, the reviewer is wondering if such an EQE is even possible at all using their device structures. As we known, in the type LEDs with planar device structure, the light extraction efficiency (LEE) without light outcoupling structure is generally not more than 25%, while their devices have a surprisingly-high out-coupling efficiency of ~40% in the planar device structure without using any light outcoupling structures (Fig. R12). The performance including device operational lifetime (the surface ligands of colloidal perovskite quantum dots with ionic nature are not very stable) reported by these authors should be verified by an independent laboratory. I have strong concerns about the data analyses the device performance, which should be addressed before the paper can be considered for publication anywhere.

The performance lifetime enhancement are mainly attributed to defect passivation of the quantum dots suppression of ion migration, but the discussion lacks deeper mechanistic insights that could better highlight the novelty of this work. Several questions are provided below to help the authors further improve the quality of the manuscript.

The manuscript claims that the BN layer can passivate surface defects of the quantum dots. However, since BN is deposited

beneath the quantum dot layer, this explanation is not entirely convincing. A more reasonable interpretation is that BN mainly passivates interface defects, improves interfacial energy alignment, optimizes carrier transport. The authors are encouraged to clarify this point in the discussion to avoid conceptual confusion.

The authors state that the BN interlayer can suppress ion migration at the quantum dot surface. However, since the interlayer only exists at the interface between the QD layer adjacent layers, its ability to directly inhibit ion migration through the thickness of the QD layer remains unclear. Furthermore, in the schematic illustration of Figure 3g, the authors also claim that ion migration on the side of the electron transport layer is suppressed, yet no detailed mechanism or experimental evidence is provided in the manuscript. The authors are encouraged to clarify this point provide further justification or supporting data.

The BN layer is deposited beneath the quantum dot film, while the manuscript indicates that the QD layer has a thickness of 2–3 layers each QD has a size of about 8–10 nm. Under these conditions, it is questionable whether direct chemical coordination between BN the QDs can occur. Moreover, since the probing depth of XPS is typically less than 10 nm, the reported chemical shifts may not unambiguously demonstrate chemical interactions between BN the QDs, but could instead arise from other factors. The authors are encouraged to clarify this point.

In the optical simulation section, it is recommended that the authors provide additional results of the energy output at the monitor ports under different thicknesses, to better evaluate the optical behavior of the devices. From the electric field distribution plots, it can be seen that in Figure c (5 nm), the downward emission is more pronounced, while at 1 nm the electric field shows stronger emission towards both sides. It would therefore be informative to show discuss the field distribution at the optimal thickness (e.g., 4 nm). In addition, the current simulation appears to have considered ITO but not the glass substrate, which may cause discrepancies compared with the actual optical outcoupling. The authors are encouraged to address or clarify this point.

In the optical simulation section, the dipole source was set parallel to the X-axis, which implies that the quantum dot film is assumed to have 100% horizontally oriented dipoles. However, the SEM images indicate that the QD film exhibits a disordered morphology, thus this modeling assumption requires further justification. In addition, the simulated light extraction efficiency reaches as high as 42%. Achieving such a high value without incorporating any external light-extraction structures is surprising, the manuscript currently lacks a clear mechanistic explanation for this result.

The quantum dots employed in this work are reported to have a cubic morphology. However, SEM results show that the QD film exhibits irregular morphology with noticeable sintering, suggesting partial loss or incomplete coverage of surface ligands. Under these conditions, it remains unclear how the underlying BN layer can effectively influence the surface defects ion migration of the overlying QDs. The manuscript currently lacks sufficient mechanistic explanation in this regard.

Reviewer #3

(Remarks to the Author)
In the manuscript "Ultrathin nano-layered boron nitride stabilizing perovskite quantum dot-based light-emitting diodes," the authors propose ultrathin nano-layered BN as an ion-migration barrier defect-passivation layer, reporting an EQE of 32% a record T50 of 25,263 hours; while most of their responses address earlier comments, several minor revisions are still needed before publication.

1. In planar stacks without explicit outcoupling structures, the theoretical EQE limit for isotropic emitters is ~30%. Therefore, the 42.93% light-extraction efficiency obtained from FDTD is overestimated relies on a methodology that is not the community standard for planar devices. Please recalculate the outcoupling using the classical dipole–TMM approach with ellipsometry-measured n k for all layers.
2. Given the reported film PLQY of 87% assuming Lambertian emission, the practical upper-limit EQE is only about 26%, which is substantially lower than the claimed 32%. The EL intensity differences in Fig. R14 suggest that the BN layer introduces measurable scattering that could enhance light extraction. If h-BN indeed improves outcoupling via scattering, the angular profile should become super-Lambertian (with stronger forward emission), rather than remain strictly Lambertian. However, the authors still report Lambertian emission, which would mask any outcoupling enhancement due to h-BN. Consequently, the origin of the high EQE value (32%) remains unclear should not be reported without further clarification.
3. The evidence for improved heat dissipation remains inconclusive. Comparing device temperature at the same luminance is not appropriate, because the control device requires a higher voltage to reach 10,000 nits. A fair comparison of Joule heating should operate both devices at the same electrical power density (J*V)

Version 2:

Reviewer comments:

Reviewer #1

(Remarks to the Author)
The authors have revised the paper according to the comments raised in the previous round of review. The quality of the paper has been improved. However, some important issues remain.

1. The authors have tested the device EQE lifetime in another laboratory. But the measurement results (plots) are not included in the revised paper. Include the actual plots specify the name of the third-party lab in the figures.
2. The maximum efficiency measured in the third-party lab was 30.8%. But there is no mention of this result in the paper.
3. The initial L/L0 was labelled as 100 in the figures. How could this be true? Do you mean 100% or 1?

Reviewer #3

(Remarks to the Author)
The authors revised the manuscript well based on the reviewer's comments, however, there remain insufficient details regarding the FDTD simulation of light extraction efficiency. Please provide references from high-impact journals that use FDTD to calculate outcoupling efficiency in PeLED/QLED/OLED devices, explain how the present simulation methodology aligns with established practices. The authors should also provide the fractional distribution among all relevant optical loss channels, including air mode, substrate mode, waveguide mode, SPP loss, to allow proper assessment of the LEE calculation. The full n, k spectra for all layers used in the simulation must be provided. In particular, the reviewer is concerned that the absorption from ITO PEDOT:PSS is not considered, both of which have absorption at the emission wavelength. The reviewer is also concerned about the use of metal boundary conditions on the lateral walls. With the current metal sidewalls, the losses through substrate mode waveguide mode might not be accurately estimated. Additionally, the SiO2 layer is modeled as 1200 nm, which is far thinner than a real glass substrate. The authors should clarify whether this model is suitable for extracting the substrate mode loss. The reviewer still highly recommends using classical TMM simulation instead of FDTD.

Version 3:

Reviewer comments:

Reviewer #1

(Remarks to the Author)
The authors have addressed the most of the issues raised.

The only remaining suggestion I would offer is that the text label "T50 = 25263 h @ 100 cd/m2" in Fig. 5h should be removed to avoid confusion, as it is an extrapolated lifetime. Alternatively, the authors should make it very clear in the figure that this is T50 (extrapolated) rather than T50.

Reviewer #3

(Remarks to the Author)
The authors have adequately addressed the concern related to light outcoupling efficiency. I recommend that the manuscript be published in Nature Communications without further revision.

Open Access This Peer Review File is licensed under a Creative Commons Attribution 4.0 International License, which permits use, sharing, adaptation, distribution and reproduction in any medium or format, as long as you give appropriate

credit to the original author(s) and the source, provide a link to the Creative Commons license, and indicate if changes were made.
In cases where reviewers are anonymous, credit should be given to 'Anonymous Referee' and the source.
The images or other third party material in this Peer Review File are included in the article's Creative Commons license, unless indicated otherwise in a credit line to the material. If material is not included in the article's Creative Commons license and your intended use is not permitted by statutory regulation or exceeds the permitted use, you will need to obtain permission directly from the copyright holder.
To view a copy of this license, visit https://creativecommons.org/licenses/by/4.0/

Response to reviewer 1

General Comment: Wang and co-authors have presented a study on enhancing the stability of perovskite quantum dot LEDs by incorporating ultrathin nano-layered boron nitride (BN) as an ion migration blocking layer. The BN-based devices show maximum EQE of 32%, and improved stability over the control devices. While the EQE claimed by the authors is amongst some of the highest efficiencies for perovskite LEDs, the device stability claims are not well supported by evidence. Several major issues need to be resolved.

Response: We sincerely appreciate your evaluation on our manuscript. Your questions and suggestions significantly help us to clarify key technologies and enrich scientific discussions. We will carefully answer all of your questions one by one.

Question 1. Judged by its introduction, the motivation of the paper is rather unclear. The authors say "Perovskite quantum dots (QDs) have attracted increasing attention in the field of light-emitting diode (LEDs) due to the high photoluminescence quantum yield (PLQY), tunable emission peaks (400-700 nm), and narrow full width at half maximum (FWHM, ~20 nm). Moreover, significant breakthroughs have been achieved in the external quantum efficiency (EQE) (>25%) of perovskite quantum dot light-emitting diodes (QLEDs) in recent years." In fact, perovskite LEDs in general (not limited to perovskite QD based LEDs) can easily achieve or outperform these properties. So the question is then: why do we need to use quantum dots? The authors should provide further justification.

Response: Thanks for your question about why perovskite QDs were used as the research object in our manuscript. We will now provide a detailed explanation for this:

Lead halide perovskite (LHP) are semiconductor materials with a perovskite crystal structure (general formula $APbX_3$). The unique features of the LHP crystal structure endow them with properties like high absorption coefficient, long carrier lifetime, high carrier mobility, weak exciton binding energy, etc. [*Adv. Mater.* 2021, 33, 2008574; *Nature* 2025, 643, 96].

Relatively, perovskite nanostructure, **e.g., perovskite QDs possess intrinsic strong carrier confinement properties, which enables them to easily achieve high radiative recombination efficiency**: When the QDs size approaches the exciton Bohr diameter, the spatial distribution of the electron wave function is compressed, thereby triggering a significant quantum confinement effect. This confinement effect not only endows the material with size-dependent optical properties (such as tunable emission wavelengths), but also significantly enhances the radiative emission efficiency by suppressing non-radiative recombination, thereby enabling electronic and optical behaviors that traditional bulk

materials cannot achieve (*Nat. Commun.* 2024, 15, 5696). **This characteristic provides a completely new degree of freedom for the design of novel functional materials and the optimization of devices.**

Furthermore, compared with perovskite polycrystalline films, **perovskite QDs exhibit better substrate compatibility (The film-forming quality is almost unaffected by the processing atmosphere and temperature):** the QDs can be directly deposited on flexible polymers, rough surfaces, or even complex three-dimensional structures using solution-based methods such as spin-coating and inkjet printing, and usually do not require post-treatment at different temperatures and atmosphere. In contrast, perovskite films are typically formed by dissolving the ionic precursors in polar solvents, and during the film formation process, the surface characteristics of the underlying substrate, such as surface tension and atmosphere, have obviously affected the film process and quality. In device fabrication, the performance is usually less reproducible (*Chem. Rev.* 2019, 119, 3193). Therefore, in our research, we chose perovskite QDs as the subject of study. In addition, perovskite QDs and LEDs are also an important field that has received extensive attention (*Nature* 2024, 629, 586).

Change made in the main text (Page 3)

Solution-processed perovskite quantum dots (QDs) are emerging as promising materials in next-generation light-emitting diodes (LEDs) due to the strong carrier confinement, tunable emission peaks (400-700 nm), and good substrate compatibility.[1-3] Moreover, significant breakthroughs have been achieved in the external quantum efficiency (EQE) (>25%) of perovskite quantum dot light-emitting diodes (QLEDs) in recent years.[4-6]

Question 2. In Figure 5h, the authors show the data points: about 400 h at 1000 cd/m^2, and >1000 h at around 500 cd/m^2. The authors did not say whether these points are from data extrapolation, so we could assume that these are from their experiments. The authors should provide the actual luminance decay curves similar to Figure 5g, with current densities specified. The authors must clarify whether data points are extrapolated (in text and in figures).

Response: We sincerely appreciate the reviewer's careful attention to the lifetime data presentation. Below, we have supplemented the experimental data:

Firstly, the data points shown in Fig. 5h of the manuscript are all derived from experimental data. To address the concerns of the reviewers, **we have provided the L/L_0 versus time data graph for all the data points (Fig. R1) as well as the corresponding data (Table R1).**

We thank the reviewer for highlighting the need for clarity. The revisions now unambiguously distinguish between measured and extrapolated data, ensuring full reproducibility of our stability claims.

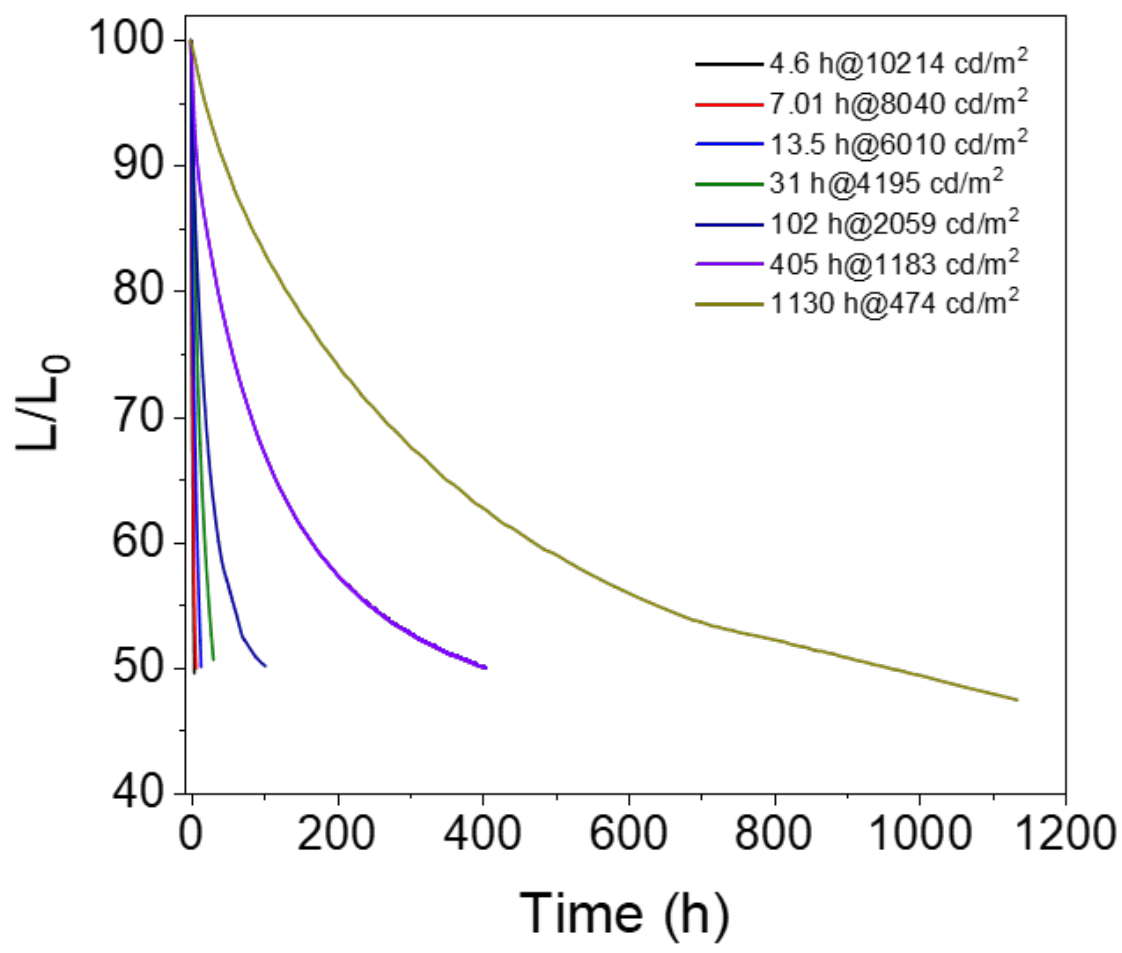

Fig. R1 The relationship between T_{50} and luminance in BN-optimized QLED accelerated lifetime experiments under different luminance.

Table.R1 Performance parameters of BN-optimized QLED under different current densities.

Luminance (cd/m^2)	Current density (mA/cm^2)	Time (h)
10214	9.7	4.6
8040	7.32	7.01
6010	5.34	13.5
4195	3.42	31
2059	1.66	102
1183	0.81	405
474	0.41	1130

Question 3. The claim that BN effectively inhibits ion migration is not fully supported by mechanistic evidence. Alternative explanations are not discussed. The authors should provide more evidence to support the ion migration inhibition claim and discuss alternative explanations.

Response: We are grateful for the valuable comments provided by the reviewers. We have supplemented the mechanism evidence through the following experiments and discussions, and analyzed other possibilities:

In addition to the DFT calculations and ToF-SIMS depth analysis discussed in the manuscript regarding the inhibition of ion migration, **to directly observe the ion migration behavior, we designed vertical ohmic devices ITO/QDs/Al and ITO/BN/QDs/Al, and applied a fixed bias to observe the PL evolution within 200 s (each device was**

encapsulated with a glass slide and ultraviolet adhesive). By applying a bias of 3.0 V, both ITO/QDs/Al and ITO/BN/QDs/Al samples showed instantaneous intensity fluctuation caused by the interference of electric field on the equilibrium carriers. As can be seen from Fig. R2, **after a longer bias for 200 s, the PL intensity of ITO/QDs/Al sample decreased rapidly. The study shows that the attenuation of PL intensity is attributed to the ion migration driven by the electric field, whereby the perovskite film accumulates a high density of defects** (*Science* 2025, 387, 186). **In contrast, the ITO/BN/QDs/Al sample maintained a stable PL intensity throughout the entire measurement period.** This result indicates that the introduction of the BN intermediate layer significantly inhibits ion migration in the perovskite QD films.

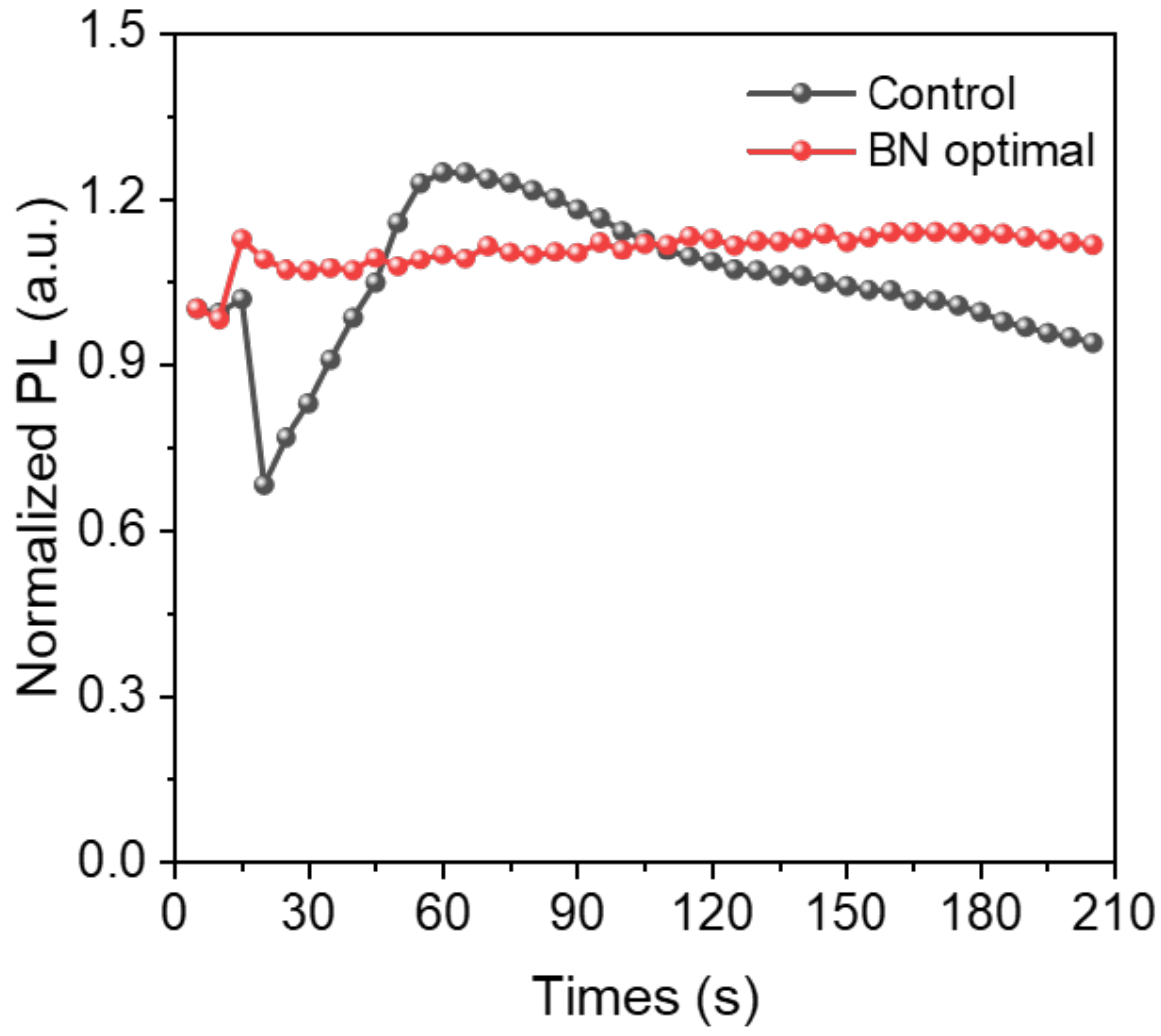

Fig. R2 Time-resolved PL intensity for ITO/QDs/Al and ITO/BN/QDs/Al devices under 3.0 V bias.

Question 4. The heat dissipation mechanism enabled by BN is not quantified or thoroughly discussed.

Response: Thank you for your valuable questions, which help us fully explain the improvement of the thermal stability performance of QLED by BN. We have supplemented quantitative experiments on the heat dissipation of BN in QDs films and QLEDs, and further explained its heat dissipation mechanism. As follows:

First, from the infrared camera images of the control and BN-optimized QD films after heating to 80 °C in air, it can be observed that **after 30 s, the control films reached 47.5 °C, whereas the BN-optimized QD films only reached 35.9 °C (Fig. R3).**

Furthermore, from the infrared camera images of control and BN-optimized devices after operation at 10000 cd/m^2 for 60 s, it can be seen that **the temperature of the control device**

reaches 67.3 °C, while the BN-optimized device is only 43.6 °C (Fig. R4).

These results indicate that the introduction of BN helps with rapid heat dissipation, which is very beneficial for reducing the ionic migration of perovskite under thermal stress and improving the stability performance of the device.

The heat dissipation mechanism of BN is also further explained as follows:

Studies have shown that **BN** contains **a thermal-excited hyperbolic phonon polariton (HPhP) modes, and HPhP can serve as an efficient heat management channel** (*Nat. Mater.* 2025, 24, 698). **The key lies in forming a unique nano-localized optical field in the infrared band, allowing HPhP to strongly near-field couple and capture the thermal radiation generated by the device operation, and using their ultra-low loss characteristics to direct and rapidly diffuse the heat energy.** This active interface heat transfer enhancement mechanism significantly reduces the local temperature peak of the hot spot during LED operation, thereby effectively improving the maximum brightness, efficiency stability, and working life of QLEDs.

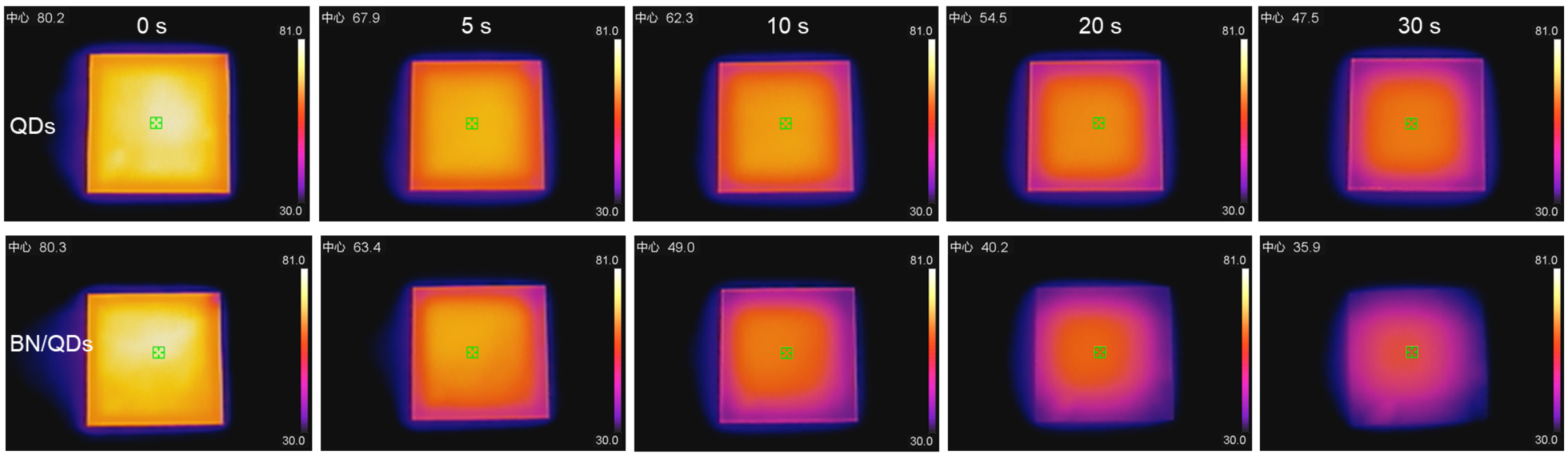

Fig. R3 Infrared camera images of control and BN-optimized QD films as a function of time in air after heating to 80 °C.

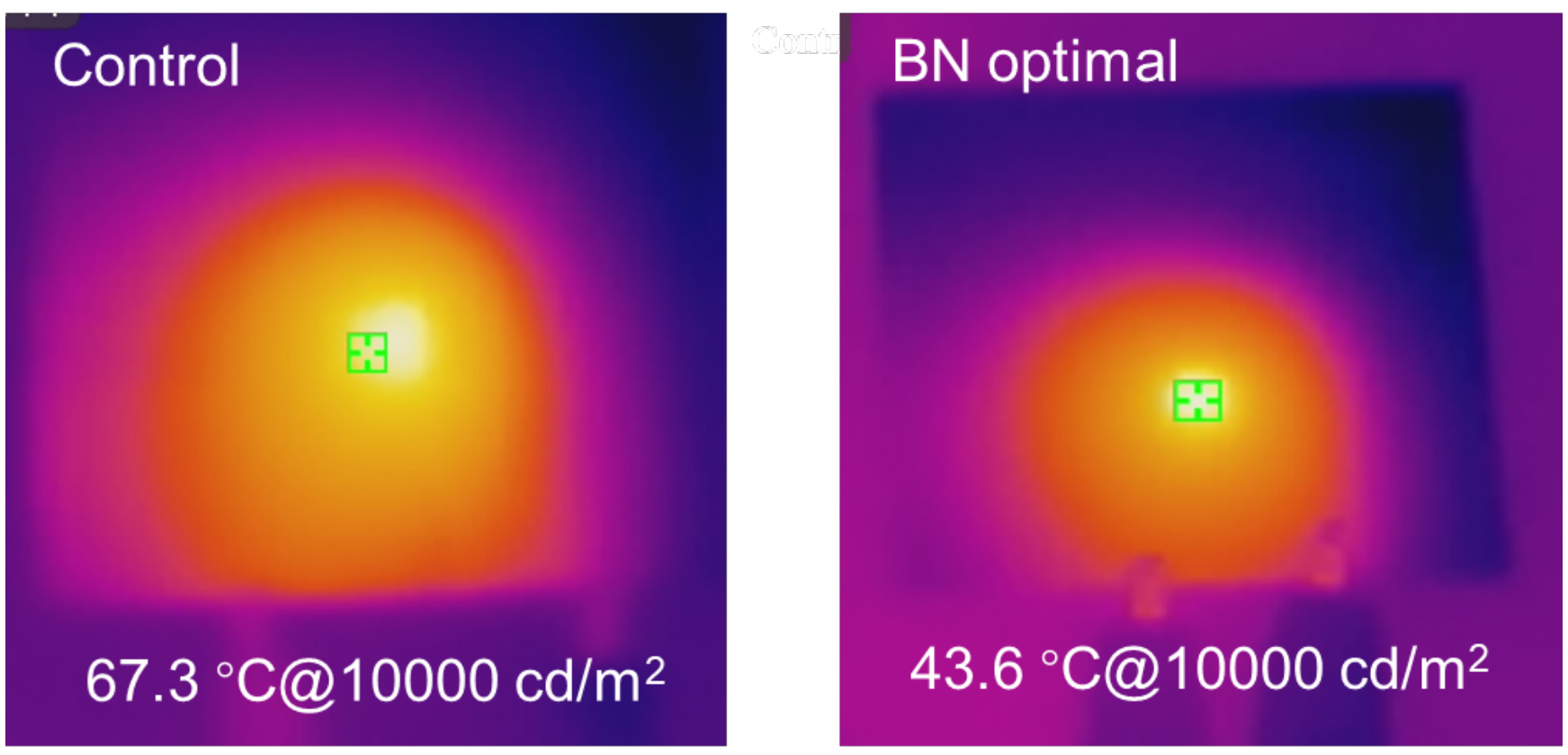

Fig. R4 Infrared camera images of control and BN-optimized devices after operation at 10000 cd/m^2 for 60 s.

Change made in the main text (Page 9)

The BN thermal-excited hyperbolic phonon polariton (HPhP) modes, serve as an efficient heat management channel, can effectively improve the heat dissipation issue during the operation of the device.

In addition, from the infrared camera images of the control and BN-optimized QD films after heating to 80 °C in air, it can be observed that after 30 s, the control films reached 47.5 °C, whereas the BN-optimized QD films only reached 35.9 °C (Fig. 4e). Furthermore, after running at 10000 cd/m^2 for 60 s, the temperature of the control device reached 67.3 °C, while the temperature of the BN-optimized device was only 43.6 °C (Supplementary Fig. 22).

Question 5. The improvements in EQE and lifetime are mainly compared to the control devices. Comparisons to other types of high-performance perovskite LEDs (not limited to $CsPbBr_3$ QD devices) are not thorough enough.

Response: We are grateful for the constructive suggestions provided by the reviewers. To better highlight the level of our work, we have now expanded the discussion and comparison with other state-of-the-art perovskite LEDs (including QDs and film-based devices) in the revised manuscript. A new table (Table R2) summarizes the key comparisons, which cover the structure of the devices and the main performance indicators (EQE, lifetime). This revision indicates that in our work, the performance of the BN-optimized QLED devices **is competitive with the best reported values in this field**.

Table R2 Comparison of high-performance green perovskite LEDs.

Device structure	EQE_{max} (%)	T_{50}	Ref.
ITO/PEDOT:PSS/Perovskite NCs/TPBi/LiF/Al	26.7	~3 h@100 cd/m^2	*ACS Energy Lett.* 2023, **8**, 927
ITO/Buf-HIL/Perovskite NCs/ZADN/TPBi/BCP:Li/MoO_3/CBP/TAPC/ZADN/LiF/Al)	37	5596 h@100 cd/m^2	*Nat. Nanotechnol.* 2024, **19**, 624
ITO/$Ni_{0.9}Mg_{0.1}O_x$/PVK/PEG/2D Perovskite/TPBi/LiF/Al	30.84	1.68 h@100 cd/m^2	*Adv. Mater.* 2023, **35**, 2302283
FTO/GraHIL/3D Perovskite @BPA/ZADN/LiF/Al	28.9	31808 h@100 cd/m^2	*Nature* 2022, **611**, 688
ITO/NiO_x/PTAA/PVK/0D(3D) Perovskite/TPBi/LiF/Al	31	-	*Adv. Mater.* 2024, **36**, 2406706
ITO/PEDOT:PSS:PFI/RDPs/3TPYMB/LiF/Al	25.6	1.92 h @ 7200 cd/m^2	*Nature* 2021, **599**, 594
ITO/PEDOT:PSS/PTAA/Perovskite NCs/TPBi/PO-T2T/LiF/Al	26.2	10 h @ 100 cd/m^2	*Nat Electron* 2024, **7**, 487
ITO/PMMA/PTAA/ Perovskite /POT2T/LiF/Al	28.3	-	*Nat. Mater.* 2025, **24**, 778
FTO/NiOx/PVP/Perovskites/TmPPPyTz/LiF/Al	29.5	18.67 h @ 12000 cd/m^2	*Nat. Photon.* 2024, **18**, 363
ITO/Me-	22	185,600 h @ 100 cd/m^2	*Nature* 2025, **643**, 96

4PACz/PVK/Perovskite/ETL/LiF/Al			
FTO/GraHIL/Perovskite NCs/ZADN/LiF/Al	28.9	520 h @ 1000 cd/m^2	*Nature* 2022, **611**, 688
ITO/PEDOT:PSS/PTAA/Perovskite QDs/TPBi/LiF/Al	18.6	213 h@100 cd/m^2	*Adv. Funct. Mater.* 2024, **34**, 2308341
ITO/PEDOT:PSS/PTAA/Perovskite QDs/TPBi/PO-T2T/LiF/Al	21.63	180.1 h@100 cd/m^2	*Sci. Bull.* 2021, **66**, 36
ITO/PEDOT:PSS/PTAA/Perovskite QDs/3P/LiF/Al	30.2	3220 h@100 cd/m^2	*Angew. Chem. Int. Ed.* 2024, **63**, 202410689
ITO/PEDOT:PSS/PTAA/Perovskite QDs/TPBi/PO-T2T/LiF/Al	20.3	127 h@100 cd/m^2	*Adv. Optical Mater.* 2024, **12**, 2302253
ITO/PEDOT:PSS/Poly-TPD/Perovskite QDs/TPBi/LiF/Al	23.45	0.02 h@100 cd/m^2	*ACS Energy Lett.* 2023, **8**, 4386
ITO/PEDOT:PSS/Buf-HIL/$FA_{1-x}GA_xPbBr_3$/TPBi/LiF/Al	23.4	2.2 h@100 cd/m^2	*Nat. Photonics* 2021, **15**, 148
ITO/PEDOT:PSS/PTAA/$CsPbBr_3$ QDs/TPBi	24.7	182 h@100 cd/m^2	*Appl. Phys. Rev.* 2024, **11**, 031405
**ITO/PEDOT:PSS/PTAA/BN/Perovskite QDs/ETL/LiF/Al**	**32**	**25263 h@100 cd/m^2**	**This work**

Response to reviewer 2

General Comment: In this manuscript, the authors introduced a BN interlayer into the Pe-QLEDs configuration, which demonstrated a high EQE with enhanced stability. While the study presents a valuable mechanistic insight, the overall performance enhancement, particularly in terms of device operational stability, remains inferior to that of traditional QDs based LEDs (such as CdSe based QLEDs) and even perovskite film based LEDs. In addition, this work does not reveal new scientific phenomena/solve major scientific problems, and its contribution to the field is limited. The absence of explanations on the underlying mechanisms also makes it hard for the reviewer to suggest its publication in high impact Nature Communications. The followings are some questions to help improve the quality of this manuscript.

Response: We sincerely appreciate your evaluation of our manuscript. Your questions and suggestions have greatly helped us improve the quality of our manuscript. We will respond to all the questions you raised carefully and one by one.

Perovskite QDs possess both synthesis advantages and optoelectronic advantages: Compared with traditional QDs (such as CdSe QDs), perovskite QDs can be rapidly prepared at room temperature, with a simple and cost-effective process, significantly reducing the complexity of synthesis and energy consumption; Compared with perovskite polycrystalline films, perovskite QDs, due to their inherent strong carrier confinement characteristics, can more easily achieve high radiative recombination efficiency, and exhibit better substrate compatibility (The film-forming quality is almost unaffected by the processing atmosphere and temperature), providing greater flexibility for device design.

As a young technology, the operational stability of perovskite QLEDs remains a big challenge limiting their practical applications. A large number of cutting-edge studies have clearly confirmed that in-depth exploration of the factors and mechanisms affecting operational stability, actively developing new materials, advanced processes, and optimizing device structures are the necessary paths to improve the performance and stability of perovskite LEDs and promote their technological maturity and wide application. The breakthrough in this research direction will not only open up a broader space for perovskite LEDs in display and lighting fields, but also very likely inject new vitality and ideas into the research in related fields such as photodetectors and solar cells. (*Nature* 2024, 629, 586; *Nature* 2025, 643, 96). Our research is a precisely powerful offensive towards this major scientific issue.

It is worth noting that in this study, our manuscript successfully reveals a new scientific

phenomenon. We **innovatively applied BN** to the interface layer, effectively passivated the defects on the surface of QDs, significantly increased the ion migration barrier on the surface of QDs, and thereby greatly inhibited the ion migration inside the LED. At the same time, **BN also reduced the accumulation of Joule heat in the LED** through efficient heat conduction, and significantly improved the operational stability of the device. **The discovery of this unique phenomenon provides new ideas and methods** for solving the operational stability problem of perovskite LEDs.

In addition, we ingeniously combined computational and experimental analysis in the research process, deeply analyzed and clarified the mechanism of BN inhibiting ion migration and improving heat conduction. Moreover, we proved through detailed device simulation that the introduction of BN layer significantly improved the external coupling efficiency of LEDs. These in-depth and systematic mechanism studies have built a solid foundation for the theoretical development of this field and provided crucial theoretical support.

Furthermore, the introduction of 2D materials to enhance the stability of perovskite films or perovskite-based devices has been receiving significant attention. For instance, using MoS_2 to improve the stability of solar cells (*Science* 2025, 387, 186). Relatively, there is almost no research on 2D materials for LED applications, and the 2D materials used in solar cells cannot be directly applied to LEDs due to different working mechanisms.

In conclusion, we believe this is an important and novel design, which would promote the development of perovskite-based LEDs.

Question 1. The analysis on the interactions between BN and perovskite should be clarified. The authors claimed that the BN adsorbed on the perovskites and had a strong passivation. What's difference and correlation between the mentioned adsorption interaction and the passivation? Do they actually refer to the same adsorption interaction?

Response: We would like to apologize for any possible confusion in the concepts and sincerely thank you for bringing this crucial issue to our attention. In this manuscript, the mentioned adsorption primarily refers to the interaction between perovskite and BN, while the passivation denotes the effect of defect reduction resulting from this interaction.

The differential charge density plot clearly shows the redistribution of charges on the perovskite surface (Fig. R5), and the characteristic shifts of the peaks of Pb 4f, Br 3d, and functional group vibration peaks in XPS/FTIR spectroscopy clearly confirm that there is a strong chemical interaction between BN and the surface atoms of perovskite, resulting in a change in their chemical environment, which is a type of chemical adsorption. The DFT

calculation results show that the binding of BN to the $PbBr_2$-terminal has a relatively high adsorption energy (with a more negative value), indicating that this binding is energetically favorable and stable, and belongs to a strong chemical adsorption.

Passivation is a functional result produced by this specific chemical adsorption: BN forms chemical bonds with the defect sites on the $PbBr_2$-terminus through its active sites. This bonding effectively neutralizes the activity of the defect state (such as filling the dangling bonds), reduces the defect density (from steady-state/transient PL, TA spectra, and hole-only devices in manuscript). **Therefore, chemical adsorption is the basis and means for passivation, while defect passivation is the direct functional effect brought about by this chemical adsorption. They do not refer to the same concept but have a clear causal relationship: effective chemical adsorption (especially at defect sites) is a necessary prerequisite for achieving defect passivation.**

We have made corrections to the inappropriate expressions in the revised version.

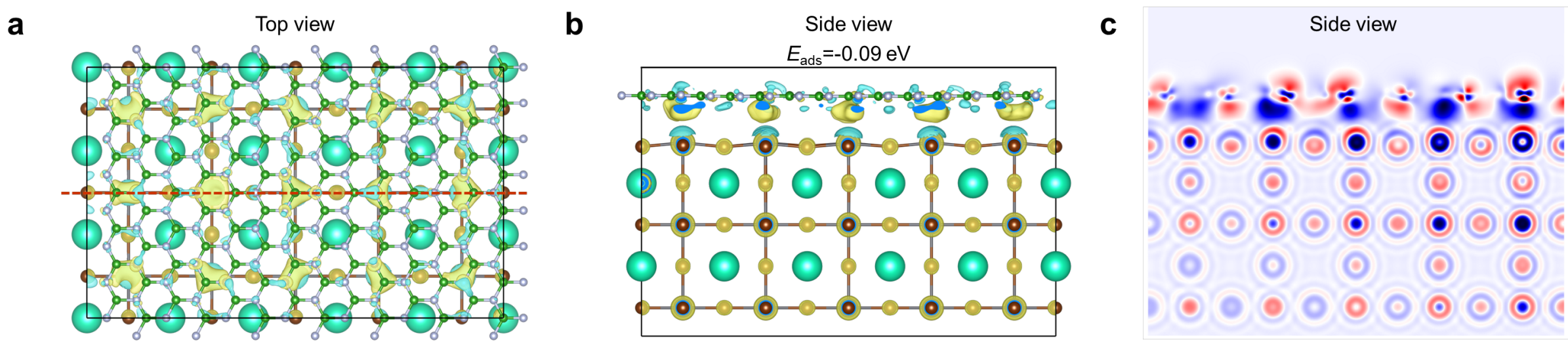

Fig. R5 3D and 2D slice of charge density differences of $PbBr_2$-terminated $CsPbBr_3$.

Question 2. Besides, when the authors claimed "This interaction serves to stabilize the perovskite structure, prevent surface defects…… Such interactions are further confirmed by the arrangement of QDs" in the manuscript, the refer of "this interaction" and "Such interactions" should be more specific.

Response: We sincerely thank the reviewers for their suggestions, which are extremely significance for us and readers. Therefore, in the manuscript, **we have explicitly indicated "this interaction" and "Such interactions" as "the bonding interactions between BN and the surface of QDs".**

Question 3. The authors claimed "…the chemical interaction between BN and perovskites." What is the chemical interaction? Is it the abovementioned adsorption? If not, what it is and what the difference and relation with abovementioned adsorption?

Response: As answered in the above question 1, **the chemical interaction refers to the chemical adsorption of BN onto the perovskite surface**. That is, BN forms chemical adsorption with the defect sites on perovskite surface through its active sites (such as N

atoms).

Furthermore, to make the expression more specific, we have modified expressions like "chemical interaction" in our manuscript to "chemical adsorption".

Question 4. How can the BN promote exciton recombination behavior? This needs to be explained in more details.

Response: Thank you for the reviewer's question. Below is our detailed explanation for them.

On the one hand, the labile surface of perovskite QDs makes them prone to forming non-radiative recombination centers during multiple purification. On the other hand, the activation energy of Br ion migration is relatively low, under the influence of external factors (light, heating, electric field, humidity), Br ions are highly likely to migrate away from their lattice positions or volatilize, thereby generating Br defects and uncoordinated Pb. These defects will capture electrons and holes, resulting in non-radiative recombination. From the answer to the above question 1, we know that **when BN is introduced, it will undergo chemical adsorption with the dangling bonds on the surface of perovskite and passivate these defects. The BN-optimized QD films shown by steady-state PL and TRPL demonstrate significantly enhanced steady-state PL intensity and significantly prolonged fluorescence lifetime, which proves that the non-radiative recombination channels caused by defects have been effectively suppressed, further promoting the excitation recombination of perovskites.**

Question 5. How can the reduced electron trap density of BN-optimized QD films can indicates the improvement of the stability perovskite QDs? Please explained in more details.

Response: Thank you for the questions raised by the reviewers. Now, we will provide detailed explanations for them.

Usually, the defects on the surface of perovskite serve as the source of ion migration. Under the influence of heat, light, and electric field, they are prone to trigger severe ion migration, resulting in a decline in the performance of perovskite materials. Meanwhile, these defects are prone to capturing electrons and holes, triggering non-radiative recombination and causing energy to dissipate in the form of heat, which generates additional heat during the operation of QDs and accelerates ion migration (*Chem*, 2024, 10, 35; *Sci. Adv.* 2025, 11, eads7054). **The introduction of the BN interlayer passivates the surface defects of the perovskite and reduces the non-radiative recombination of QDs (Fig. R6). Moreover, it increases the ion migration barrier and inhibits the ion migration at the perovskite surface (Fig. R7), thereby improving its stability to a certain extent.**

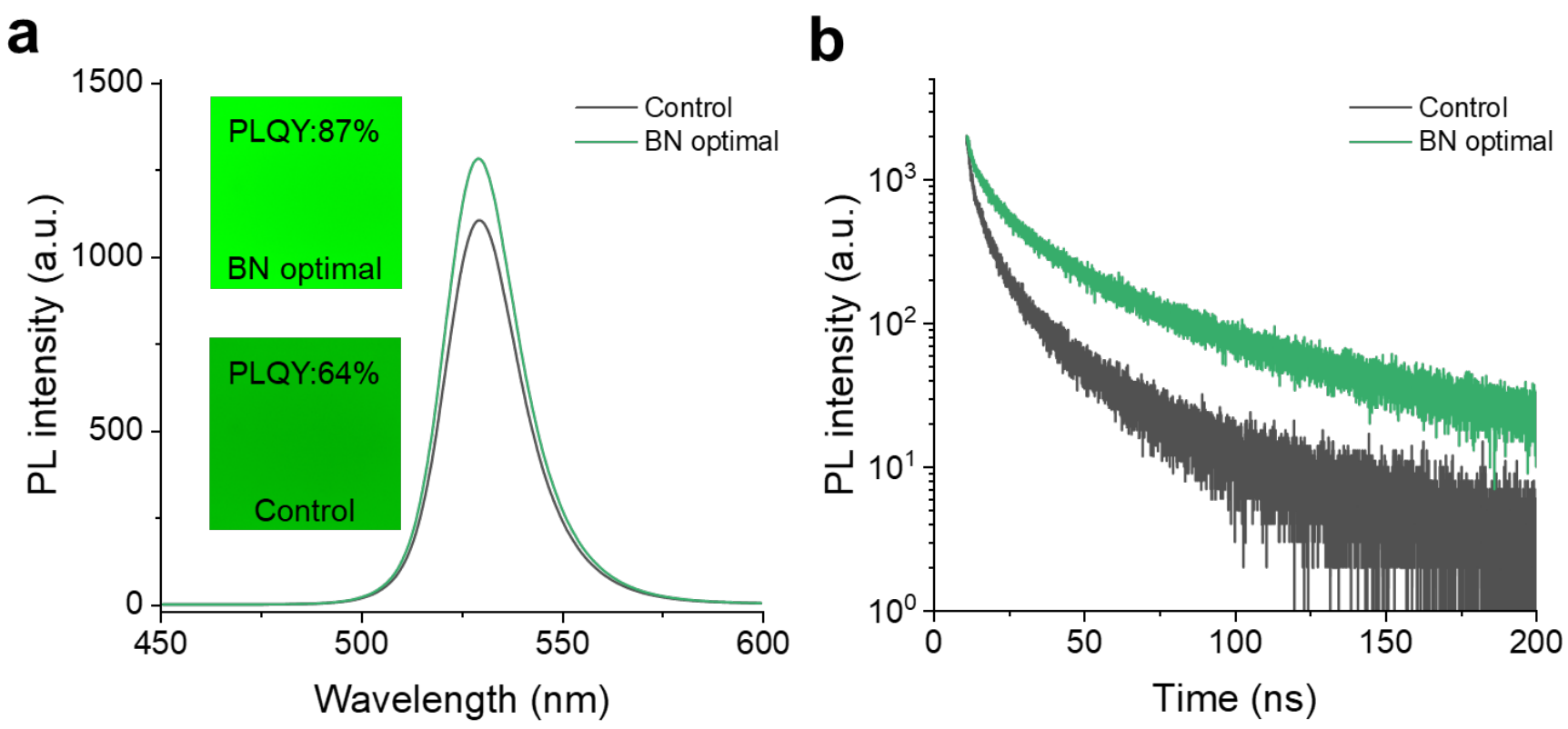

Fig. R6 (a) Steady state PL spectra, and photographs (ultraviolet lamp excitation) of control and BN-optimized QD films. (b) TRPL delay curve of control and BN-optimized QD films.

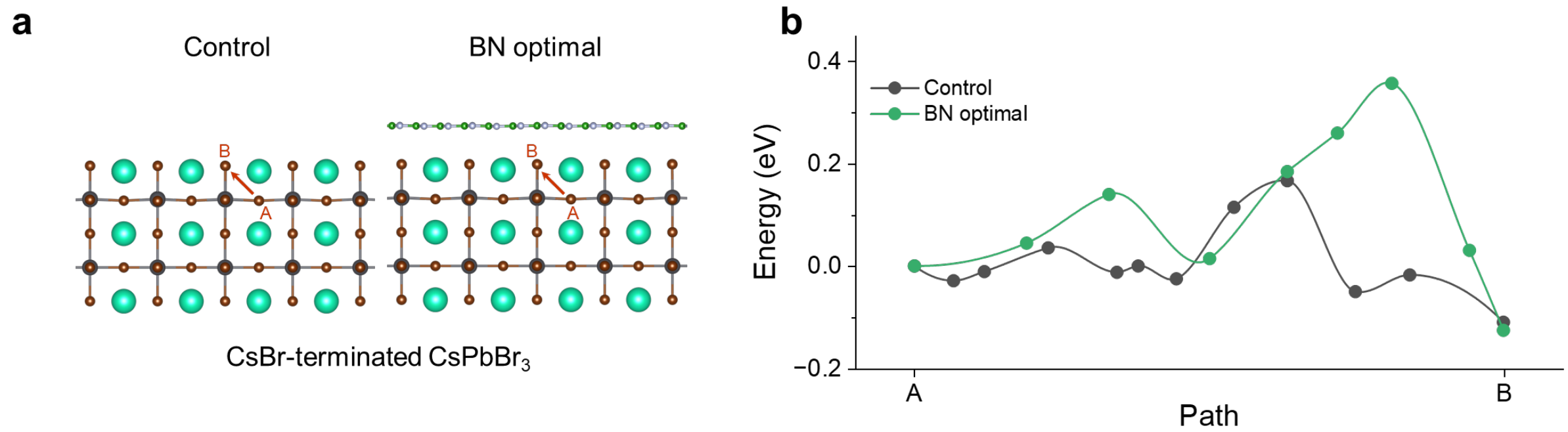

Fig. R7 The simulation path (a) and potential barriers (b) of CsBr-terminated surface $CsPbBr_3$ without/with BN adsorption.

Question 6. Since the introduction of BN can enhance the activation energy (E_a) of ion migration, how does the thickness of BN interlayer influence the PL performance of QDs and the performance of LEDs? Is there an optimal thickness of the BN interlayer?

Response: Thank you to the reviewers for raising valuable questions. In our research, BN materials possess dual characteristics. On the one hand, it can reduce defects in the material and effectively improve the recombination efficiency; on the other hand, due to the insulating property of BN, it will reduce the injection transport efficiency, thereby having a negative impact on the recombination efficiency. Given that these two effects coexist, we need to seek a balance between them.

The thickness of the BN layer plays a crucial role in this balance process. An appropriate thickness can enable BN to exert its positive effect while minimizing its negative impact. Additionally, we conducted PL performance and LED performance tests on QDs with different concentrations of BN layers. From Fig. R8 can be seen that the PL of QDs increases with the increase of BN concentration. The difference is that the EQE of QLED reaches the maximum when the BN concentration is 0.004 g/mL (Fig. R9), but the efficiency decreases

when the BN concentration is higher. This is because the chemical adsorption of BN can effectively passivate defects on the surface of QDs, suppress ion migration, and improve the radiation recombination of QDs. **However, when the concentration of BN is low, it is not sufficient to passivate defects, and when the concentration of BN is too high, it will hinder charge injection in the device, and reduce device performance. Therefore, in this manuscript, the optimal BN concentration (0.004 g/mL) is required to prepare high-performance QLED.**

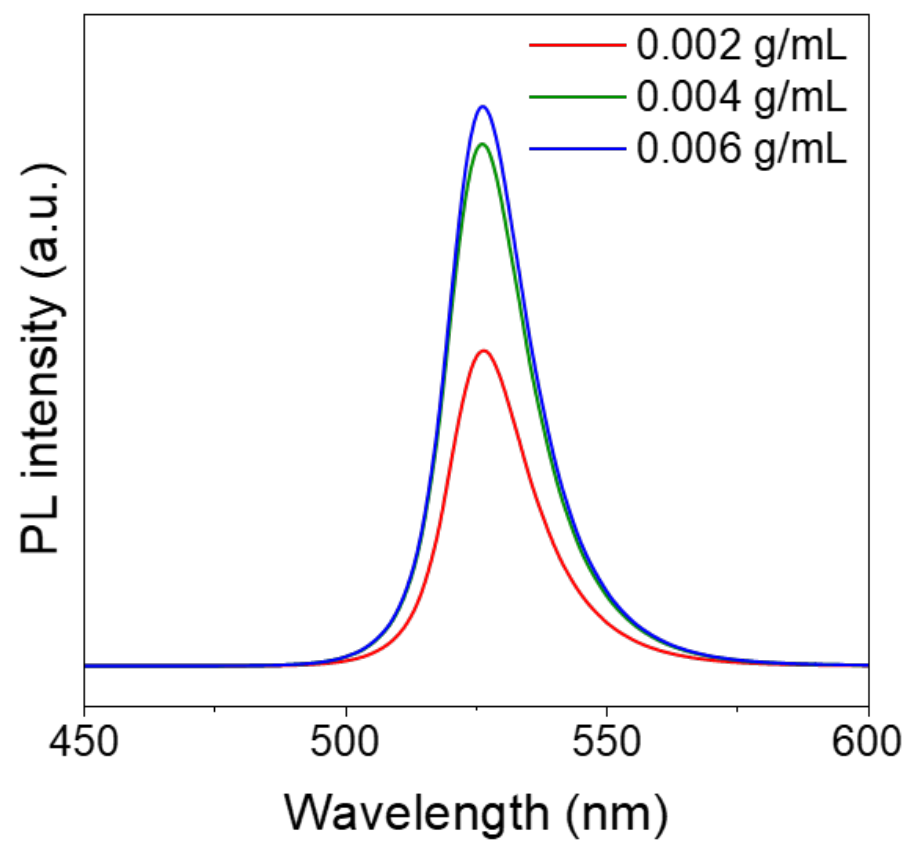

Fig. R8 PL performance of QDs with different concentrations of BN.

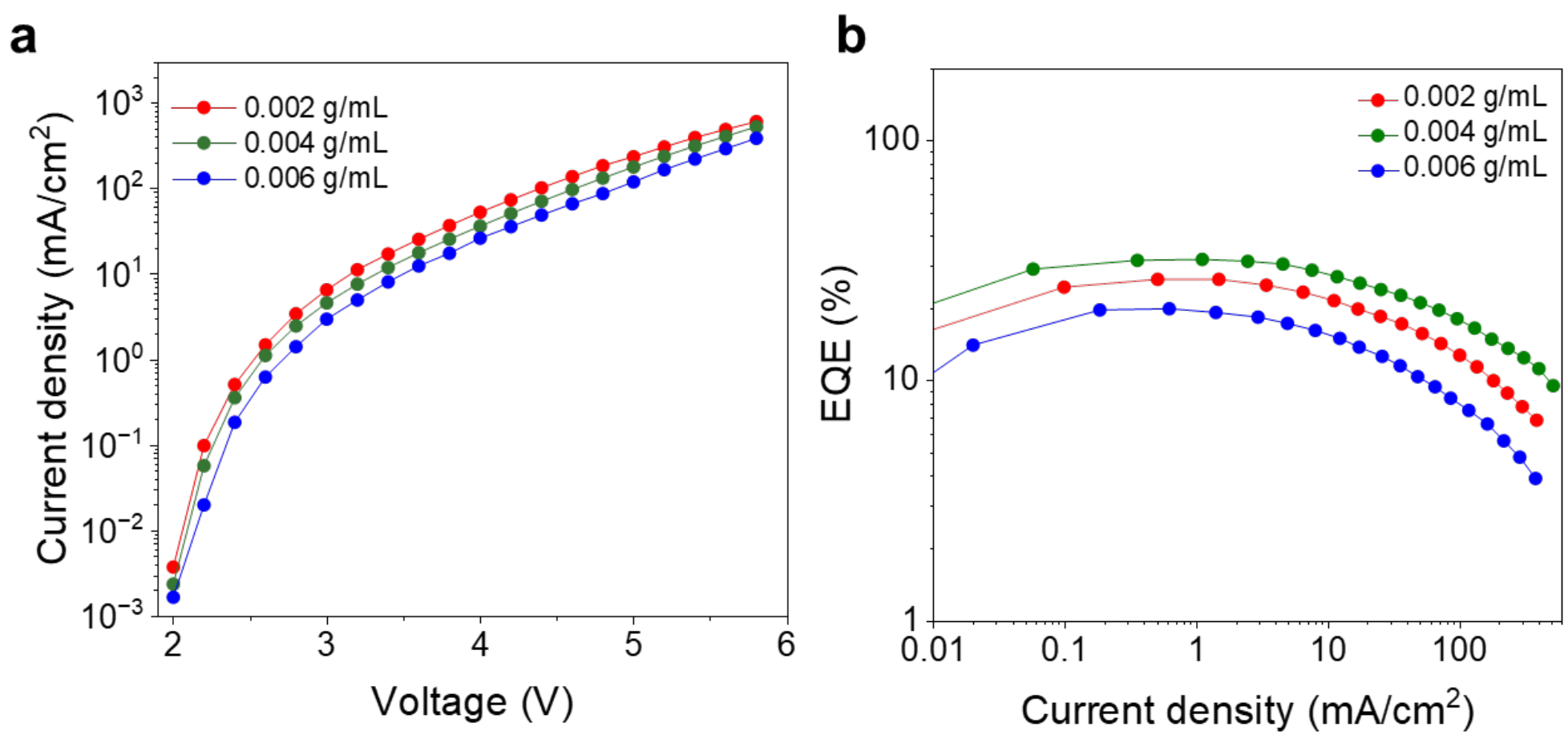

Fig. R9 Electroluminescence properties of QDs with different concentrations of BN.

Question 7. Although the BN can facilitate the dissipation of heat in perovskite films, the evidences for reduced Joule heating in operating QLEDs with BN are suggested to be provided?

Response: We would like to express our gratitude to the reviewers for their valuable suggestions. We further employed an infrared-thermal-imaging-camera to monitor the effect of BN on Joule heating in operating QLEDs. As depicted in Fig. R10, **the BN-optimized QLED exhibits a temperature of 43.6 °C after operation at 10000 cd/m^2 for 60 s, while**

the control devices experience a rapid temperature increase to 67.3 °C. This result confirms the reduced Joule heat generation and enhanced thermal dissipation properties of the BN-optimized films.

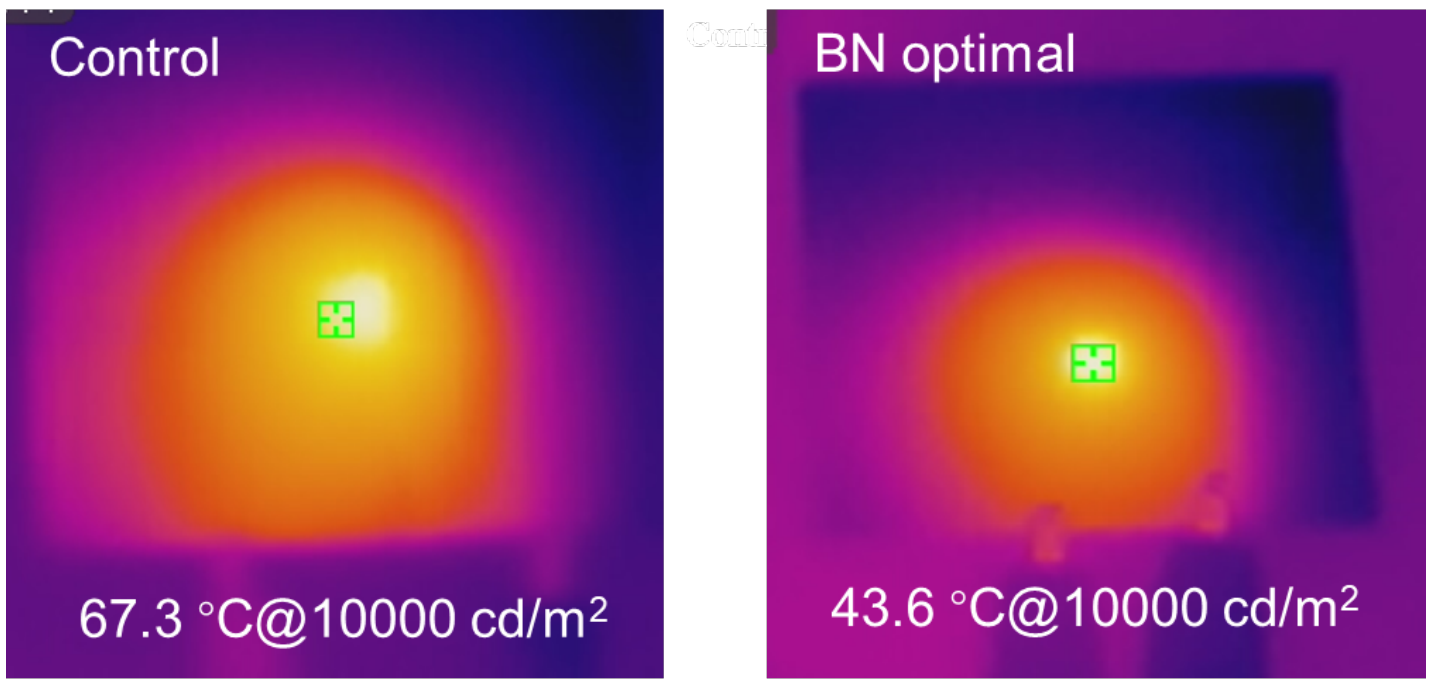

Fig. R10 Infrared thermal imaging images of control and BN optimized devices after operation at 10000 cd/m^2 for 60 s.

Change made in the main text (Page 9)

Furthermore, after running at 10000 cd/m^2 for 60 s, the temperature of the control device reached 67.3 °C, while the temperature of the BN-optimized device was only 43.6 °C (Supplementary Fig. 22).

Question 8. Some typos should be corrected, e.g. “patterns” should be “patterns”.

Response: We sincerely appreciate the reviewer's careful reading. We have corrected all noted typographical errors (e.g., “paterns” → “patterns”), and conducted a full spell-check of the manuscript using Grammarly.

Response to reviewer 3

General Comment: The authors propose a novel use of ultrathin nano-layered boron nitride (BN) as an ion-migration barrier and defect passivation layer in perovskite LEDs. This interface engineering significantly enhances both efficiency and operational stability. They report a notably high EQE of 32% and a record operational lifetime (T_{50}) of 25,263 hours. While these results are impressive, the reliability and reproducibility of the data need to be further verified. Below are detailed comments and necessary clarifications:

1. Device Performance: EQE Measurement and effect of BN

Question i. The reported EQE of 32% is exceptionally high for perovskite quantum dot LEDs without any light outcoupling enhancements, such as scattering films, or photon recycling structures. The authors should: 1) clarify whether any structural or optical enhancement was used, and 2) provide optical simulations to support such high efficiency.

Response: Thank you for the important questions raised by the reviewers. The concern you raised regarding whether the high external quantum efficiency (EQE = 32%) is dependent on optical coupling enhancement is very reasonable, especially in the absence of any additional optical structures. This value is indeed significantly higher than what is typically reported. We fully understand and appreciate your cautious attitude towards this. Now, we will specifically clarify the following in response to your two points:

Firstly, **we hereby explicitly state that all the perovskite QLED devices reported in this manuscript have not used any external or integrated optical coupling enhancement structures.** The core reason for achieving high efficiency lies in introducing a BN interlayer, which possesses the following characteristics 1. to passivate the defects on the QD surface, 2. to significantly reduce ion migration in the QLED, and 3. to improve the thermal stability performance of the QLED during operation, thereby significantly enhancing the efficiency and stability of the QLED.

Secondly, we fully agree with your viewpoint that optical simulation is crucial for understanding and confirming the light extraction performance of the device. **In response to your valuable suggestions, we have supplemented detailed optical simulation analysis, as follows:**

Model setup: The model schematic is shown in the Fig.R11. The lateral dimension of the calculation model is set to 2×2 μm^2. This LED consists of: a 100 nm ITO layer, a 40 nm PEDOT:PSS layer, a 20 nm PTAA layer, a 1 nm BN layer, a 30 nm QDs layer, a 40 nm ETL layer, a 1 nm LiF layer, and a 100 nm Al layer. Additionally, a single-pole emitter representing

electron-hole recombination is set in the center of the QDs region. The emission spectrum peak wavelength of this dipole emitter is set at 520 nm. The dipole source is polarized parallel to the X-axis direction to excite the TE mode. The absorption coefficient of the QDs layer at the 520 nm emission wavelength is set to 500 cm^{-1}. Regarding the material refractive indices, the values of the ITO layer, PEDOT:PSS layer, PTAA layer, BN layer, QDs layer, ETL layer, and LiF layer are 1.85, 1.5098, 1.607, 2.1308, 2.02, 1.727, and 1.3921 respectively. The refractive index and absorption coefficient of the Al material are set based on the built-in Al (Palik) material parameters in the FDTD material library. The absorption coefficients of the other layers are all set to 0.

Set the reflection rate of the four side boundaries to 100%, thereby inferring that the limited side dimensions are effectively infinite. The conditions for the top and bottom boundaries are set to perfectly matched layer (PML), which can completely absorb electromagnetic energy. In our model, a non-uniform grid is used for simulation, with the minimum grid size set to 0.95 nm, providing good accuracy for the calculation of LEE. The power monitor is placed 500 nm away from the ITO layer to collect the power transmission passing through the monitor. LEE is derived by calculating the ratio of the total extracted power collected by the power monitor to the total power emitted by the dipole.

The influence of BN layer thickness on the light extraction efficiency (LEE) and electric field distribution is shown in Figs. R12, R13. As can be seen from the figures, when the BN layer thickness is 1 nm, the light extraction efficiency (**42.92%)** and electric field strength of the device reach the optimal values, and the light field distribution is consistent with the results of the light extraction efficiency.

The PLQY of the BN-optimized QD films measured in the manuscript is 89%. Although PLQY is measured under light excitation and EQE is measured under electric excitation, in an ideal situation (assuming the electric injection efficiency $\eta_{inj} \approx 100\%$ and the electroluminescence process can fully utilize all excitons), the maximum theoretical EQE (EQE_{theory}) of the device can be estimated by the formula: **$EQE_{theory} \approx PLQY * LEE * \eta_{inj} \approx PLQY * LEE$, that is, $EQE_{theory} \approx 87\% * 42.92\% \approx 37.3\%$.** However, the EQE of the BN-optimized QLED in the manuscript is 32%, which is slightly lower than the theoretical calculation value. Therefore, these results indicate that it is completely reasonable for the QLED efficiency to reach 32% after the introduction of BN.

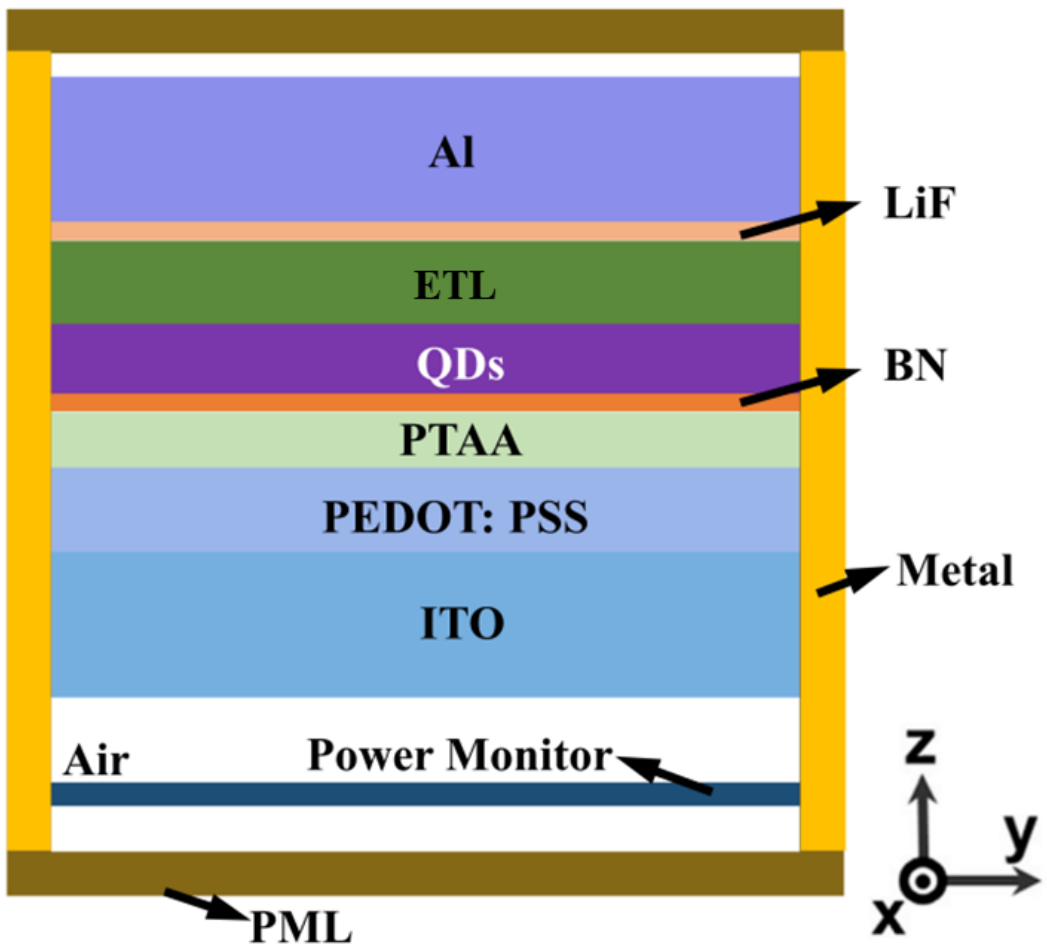

Fig. R11 Schematic Diagram of Simulation Model.

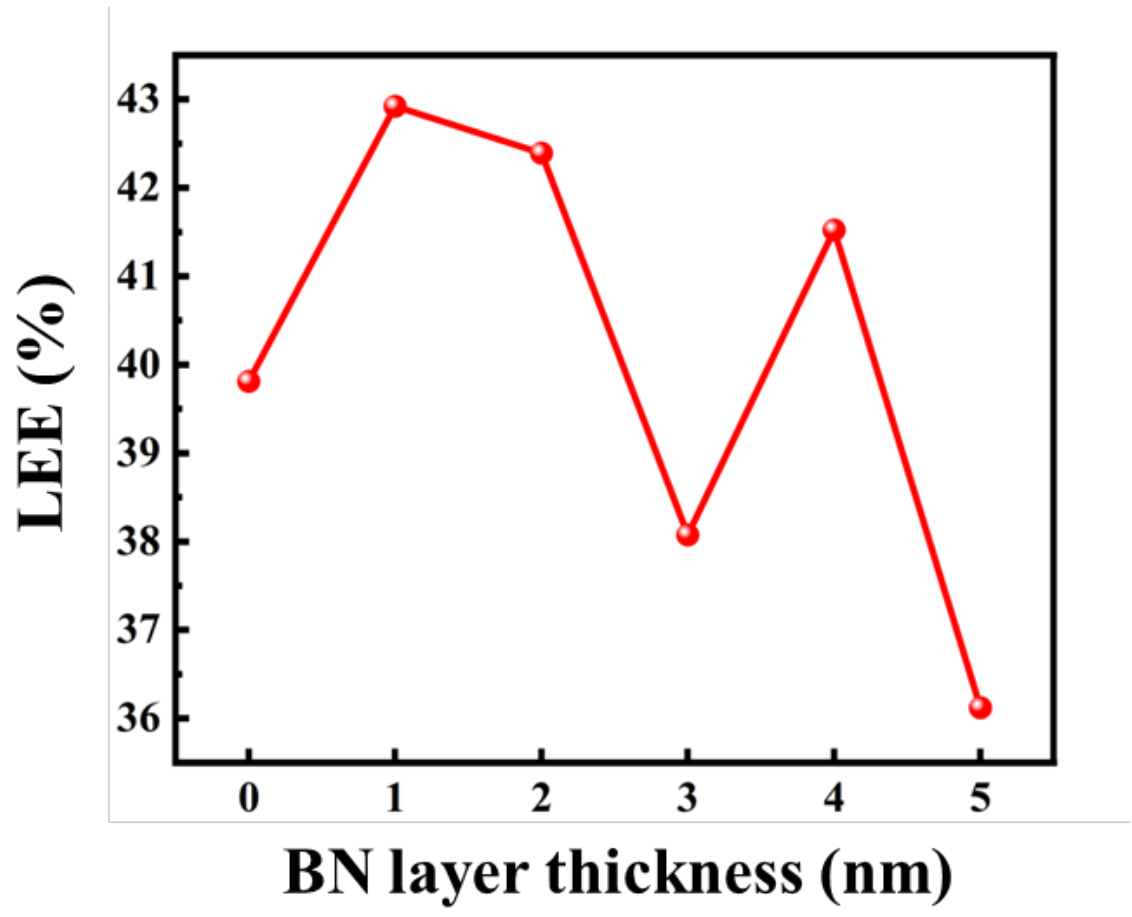

Fig. R12 The influence of BN layer thickness on LEE.

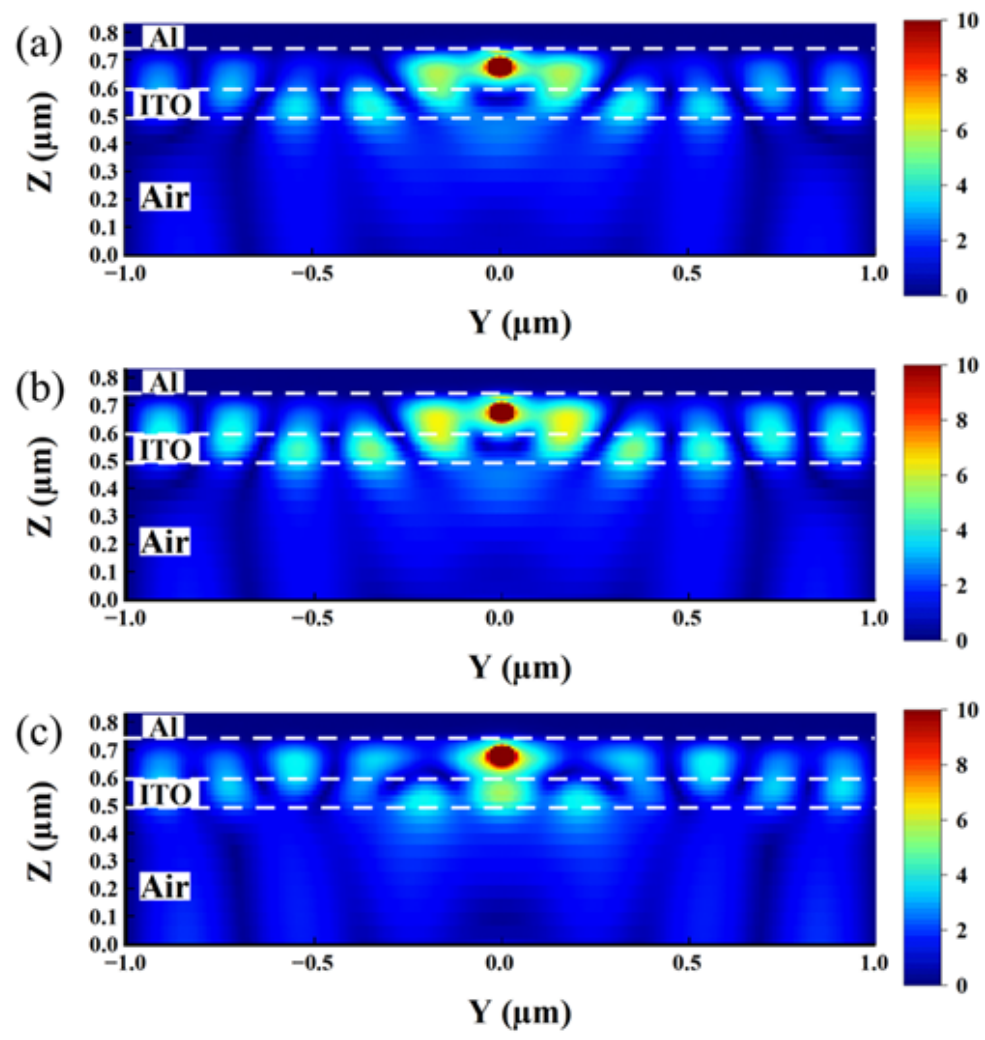

Fig. R13 Electric field distribution in the YZ plane of TE polarized light. (a) 0 nm BN layer. (b) 1 nm BN layer. (c) 5 nm BN layer.

Question ii. As shown in Supplementary Figure 2b, BN exhibits a certain scattering effect in EtOH and possesses a nanometer-scale fly-eye lens morphology, which is expected to enhance the outcoupling efficiency. The authors should measure the scattering effect of the BN layer using a haze meter and compare the degree of scattering, device performance, and EL spectrum as a function of the BN solution thickness and weight percentage.

Response: We sincerely appreciate the reviewer for providing such valuable comments. Regrettably, the test lower limit of commercial haze meters currently available on the market is generally greater than 50 nm, while the thickness of the BN layer introduced in this study is approximately 1 nm. Due to this limitation, we have not yet found a third-party institution that can accurately test the haze of this thickness BN film, so we are temporarily unable to measure the scattering effect of the BN layer using a haze meter.

In order to evaluate the light scattering effect of the BN layer, we carefully designed the following comparative experiment: the experiment was divided into the control group (ITO/PEDOT/PTAA) and the experimental group (ITO/PEDOT/PTAA/BN). Afterwards, a 365 nm ultraviolet light source was used to irradiate the sample vertically from the front, and a spectrometer and an integrated software camera were used to take a transparent photo at the ITO electrode on the back. The experimental results are shown in Fig. R14. **After introducing the BN layer, the collected light intensity significantly increased. This phenomenon indicates that the scattering effect of BN in this system helps with light collection, which also provides evidence for the high light extraction rate of BN-optimized QLED in problem one's optical simulation.** Although we were unable to directly measure the haze value, the above-based device-level light extraction comparison experiments provided strong evidence for the light management role of BN from an indirect perspective. We plan to prioritize the development of a nanoscale haze testing scheme in our subsequent work.

In addition, we also supplemented the study on the impact of introducing different concentrations of BN on the performance of QLEDs. **As shown in Fig. R15, when BN was introduced at a concentration of 0.004 g/mL, the device performance reached the optimal level.** Further analysis revealed that **when the BN concentration is too high, it would hinder charge injection in the device; while when the concentration is too low, it cannot fully passivate the surface defects of QDs, which is not conducive to the inhibition of ion migration.** Thus, it can be seen that both too high or too low BN concentration will have adverse effects on the device performance. This result is consistent with the optical simulation results of BN thickness in Question 1.

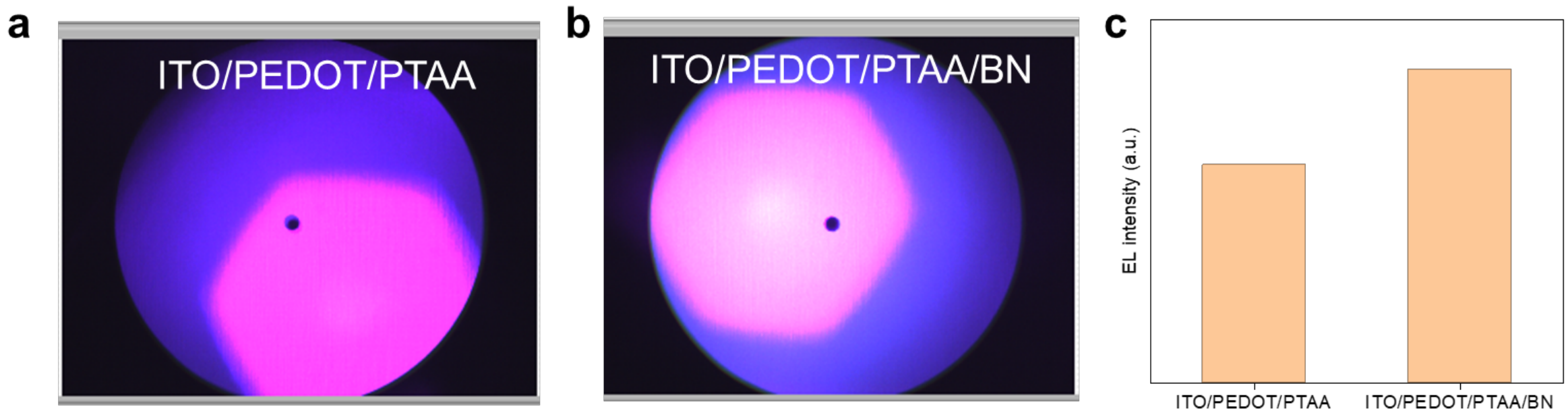

Fig. R14 The light extraction intensity of the ITO/PEDOT/PTAA and ITO/PEDOT/PTAA/BN structures.

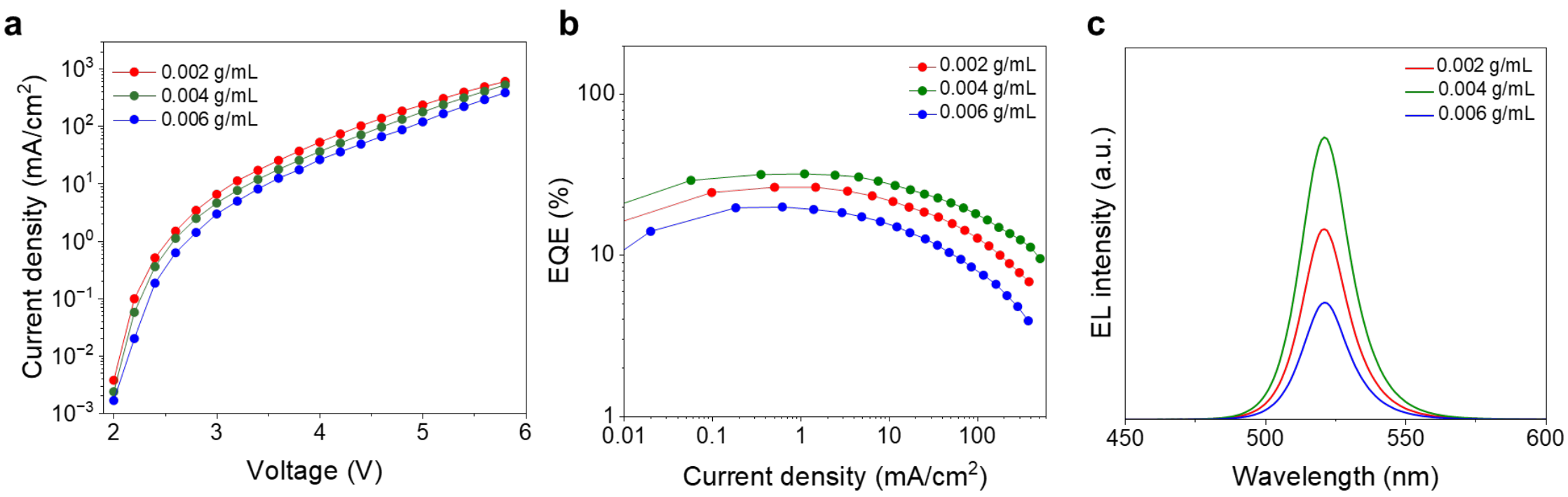

Fig. R15 Electroluminescence properties of QDs with different concentrations of BN.

Question iii. The EQE should be validated using an integrating sphere setup and independently verified by a well-established external laboratory to rule out measurement artifacts.

Response: Thank you for your reminder. To ensure the credibility of the data, **we first verified it in our group's integrated sphere testing system (Fig. R16)**. The testing system consists of a fiber integration sphere, a Keithley 2400 source meter, and an LA-5000N spectrometer (Suzhou Fusida Scientific Instrument Co., Ltd designed the measurement equipment). The screenshot of the test data is shown in Fig. R17. **The maximum peak EQE of the BN-optimized QLED is 31.2%.**

In addition, **we also compared the test data in our laboratory with the device test results in Shen Huibin's research group of Henan University. As shown in Fig. R18, the measured EQE values are 31.5% (our laboratory) and 30.8% (other laboratories).** These findings indicate that the performance data we obtained are consistent across different laboratories with different equipment settings.

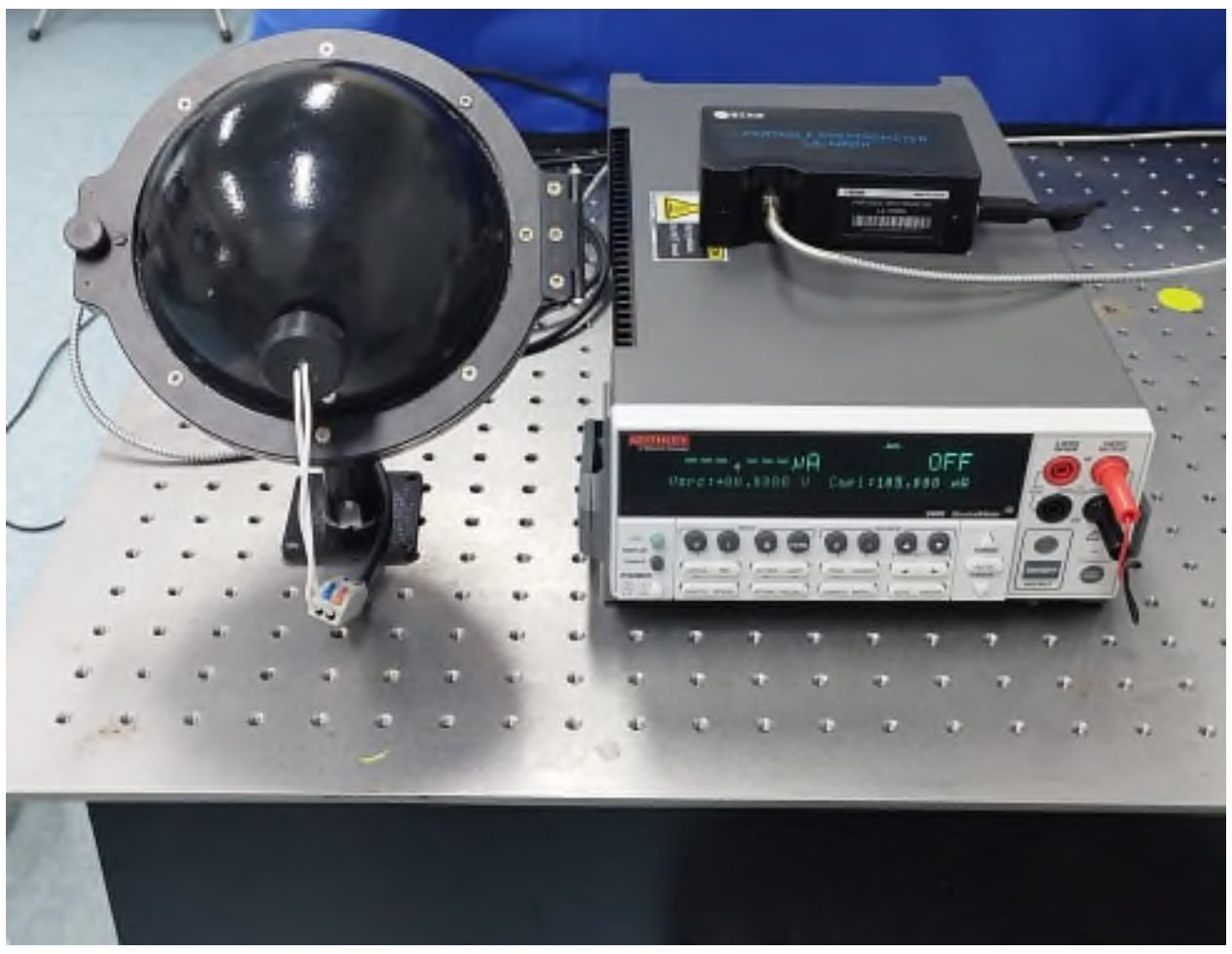

Fig. R16 Physical diagram of integrating sphere**.**

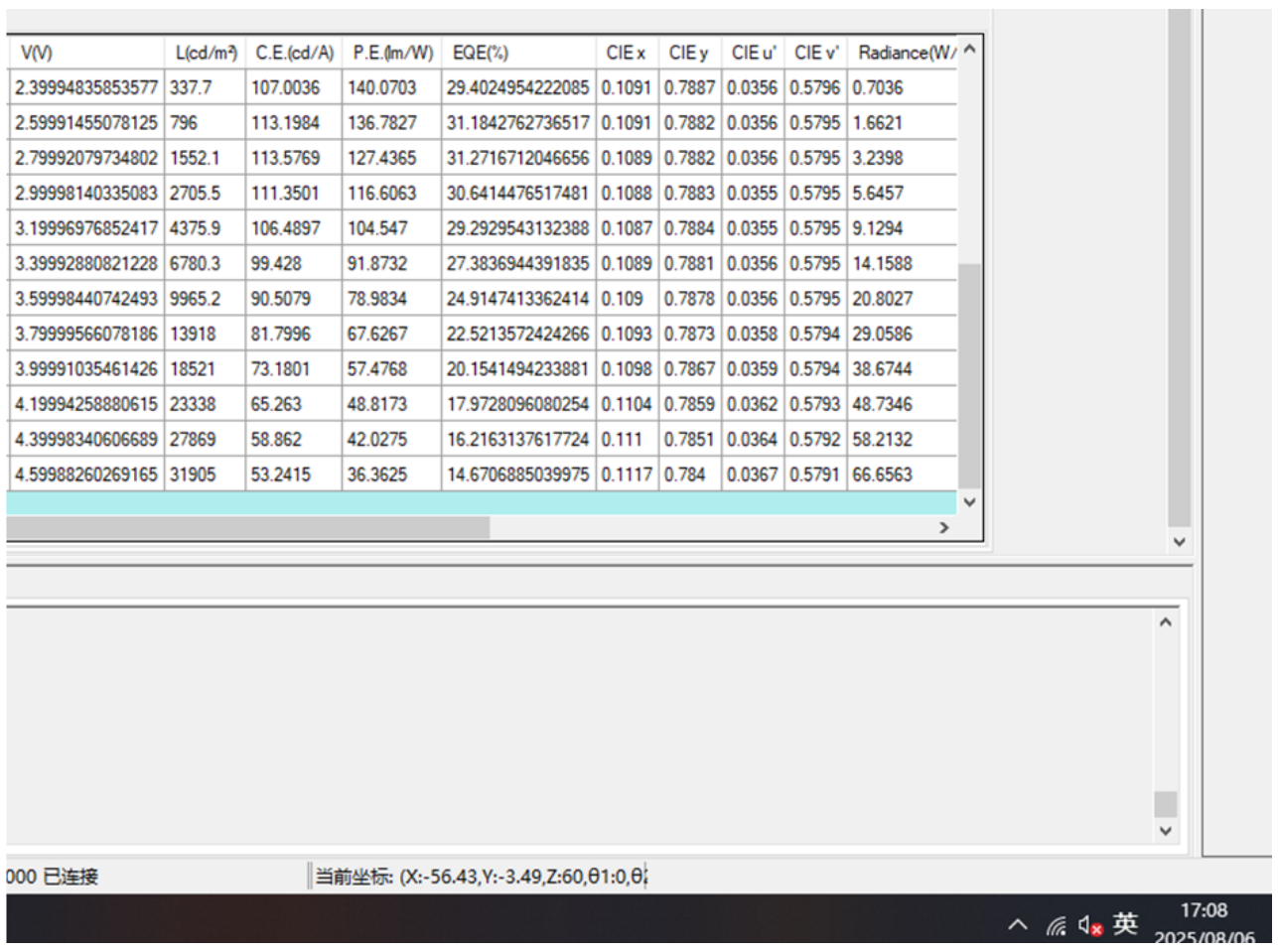

V(V)	L(cd/m²)	C.E.(cd/A)	P.E.(lm/W)	EQE(%)	CIE x	CIE y	CIE u'	CIE v'	Radiance(W/
2.39994835853577	337.7	107.0036	140.0703	29.4024954222085	0.1091	0.7887	0.0356	0.5796	0.7036
2.59991455078125	796	113.1984	136.7827	31.1842762736517	0.1091	0.7882	0.0356	0.5795	1.6621
2.79992079734802	1552.1	113.5769	127.4365	31.2716712046656	0.1089	0.7882	0.0356	0.5795	3.2398
2.99998140335083	2705.5	111.3501	116.6063	30.6414476517481	0.1088	0.7883	0.0355	0.5795	5.6457
3.19996976852417	4375.9	106.4897	104.547	29.2929543132388	0.1087	0.7884	0.0355	0.5795	9.1294
3.39992880821228	6780.3	99.428	91.8732	27.3836944391835	0.1089	0.7881	0.0356	0.5795	14.1588
3.59998440742493	9965.2	90.5079	78.9834	24.9147413362414	0.109	0.7878	0.0356	0.5795	20.8027
3.79999566078186	13918	81.7996	67.6267	22.5213572424266	0.1093	0.7873	0.0358	0.5794	29.0586
3.99991035461426	18521	73.1801	57.4768	20.1541494233881	0.1098	0.7867	0.0359	0.5794	38.6744
4.19994258880615	23338	65.263	48.8173	17.9728096080254	0.1104	0.7859	0.0362	0.5793	48.7346
4.39998340606689	27869	58.862	42.0275	16.2163137617724	0.111	0.7851	0.0364	0.5792	58.2132
4.59988260269165	31905	53.2415	36.3625	14.6706885039975	0.1117	0.784	0.0367	0.5791	66.6563

Fig. R17 Screenshot of the integral sphere test performance of the BN-optimized QLED.

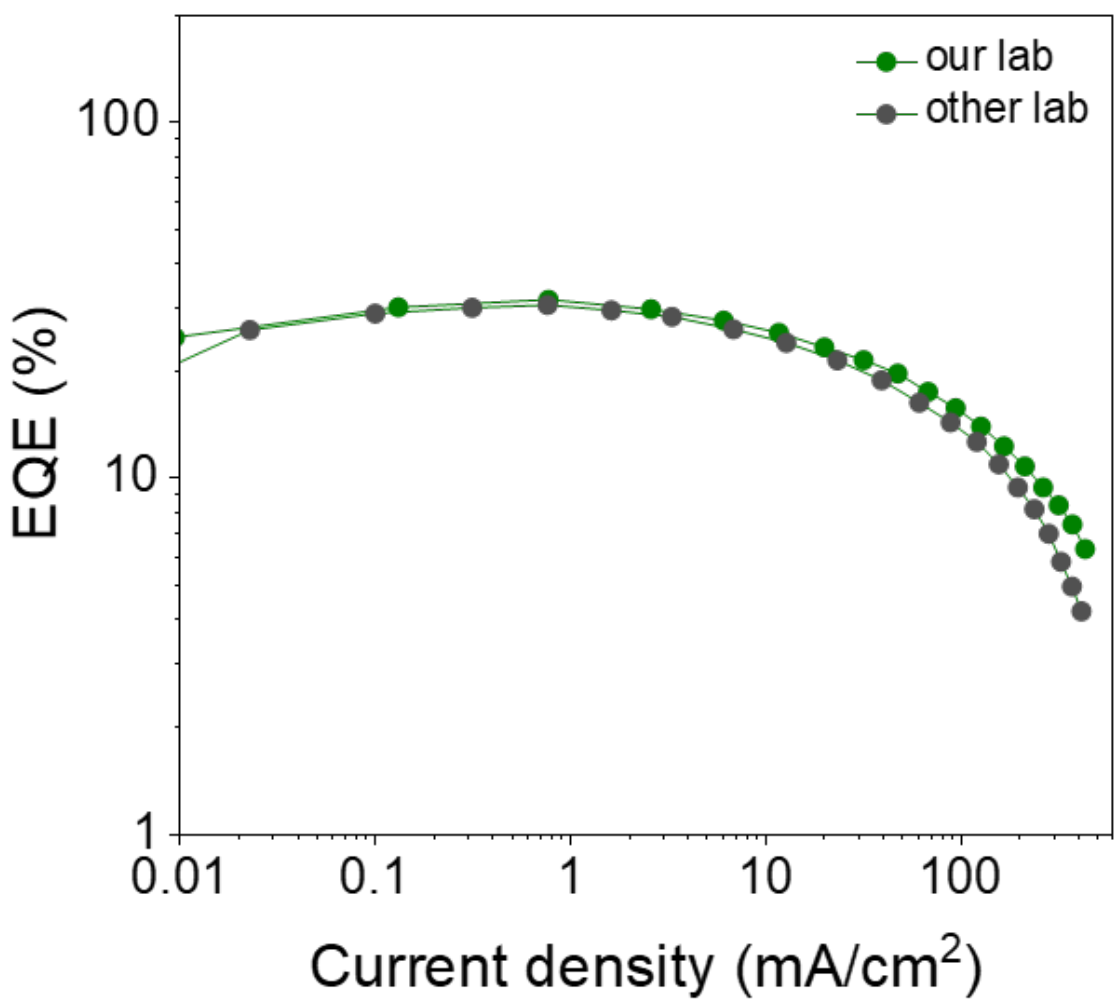

Fig. R18 EQE data with measurements from other labs.

Question iv. Details of the EQE measurement procedure, including detector calibration and measurement conditions (e.g., device orientation, encapsulation), must be clearly described.

Response: Thank you for the valuable suggestions from the reviewer. We have supplemented the details of the EQE measurement procedure as follows:

First, using a UV curing packaging instrument, and package the already deposited QLED device with a glass cover plate in N_2 glove box. The specific process was as follows: a UV curing adhesive was pre-applied between the glass cover plate and the QLED device substrate, then placed in the packaging instrument (Fig. R19), and UV irradiation with a duration of 15 s was applied to rapidly cross-link and solidify the UV curing adhesive, achieving a firm and airtight bond between the glass cover plate and the QLED device substrate.

Secondly, the encapsulated QLED device was transferred to a photovoltaic performance testing platform integrated with the core components of the Keithley 2602B system source meter (Fig. R20a), CS-2000 spectral radiometric luminance meter (Fig. R20b), and analysis software package. Then, at room temperature, a linear scanning voltage was applied to the encapsulated QLED device (scan range: 0 V to 6.0 V, scan step: 0.20 V). The Keithley 2602B source meter was responsible for applying the current and real-time monitoring the voltage response of the device. Meanwhile, the CS-2000 simultaneously measured the light radiation luminance (*L*) and the electroluminescence (EL) spectrum generated by the device under the corresponding voltage drive, and finally, the key photovoltaic performance data set of the QLED device such as *EQE*, *I-V-L*, EL, etc. was obtained through the analysis software package.

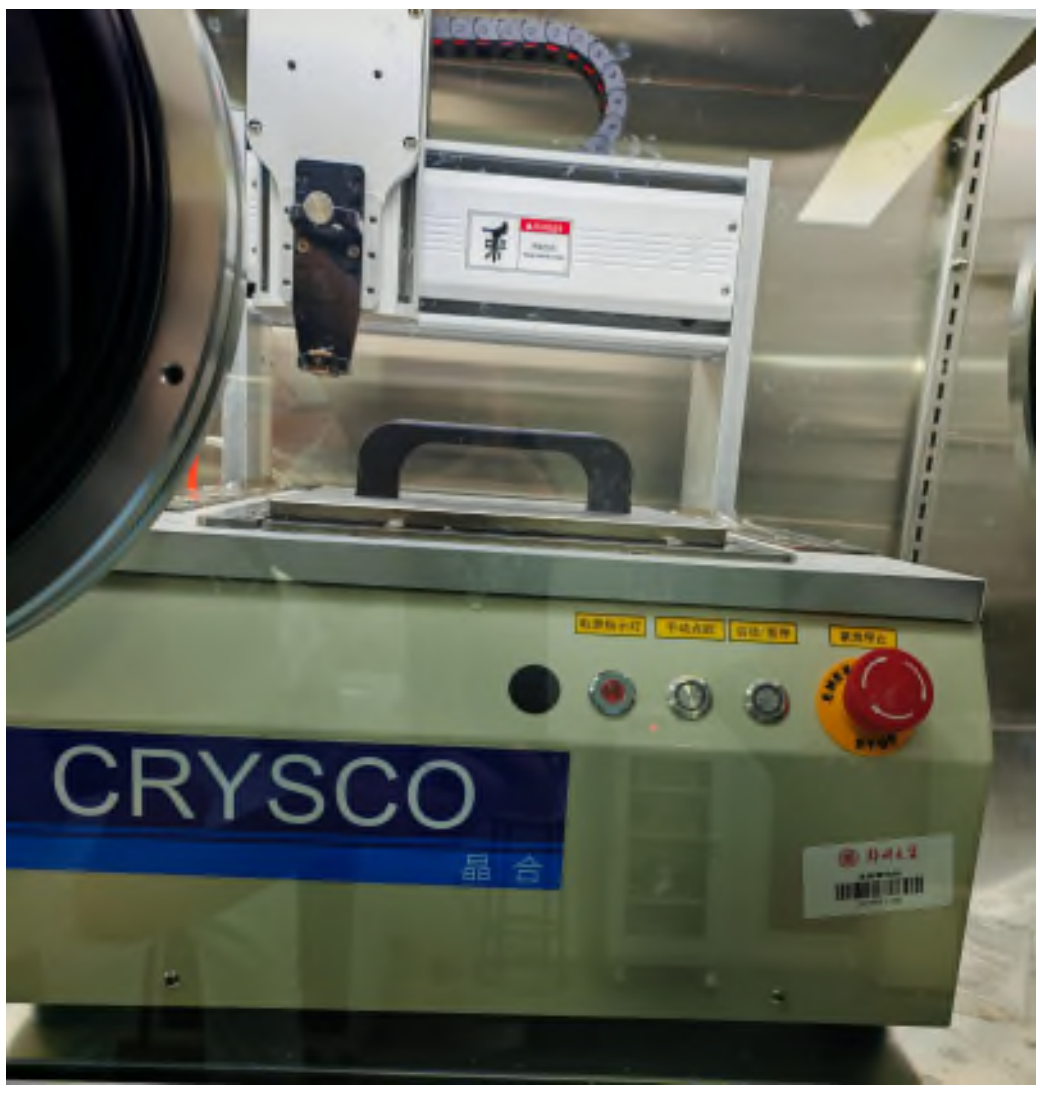

Fig. R19 UV Ultraviolet Packaging Instrument.

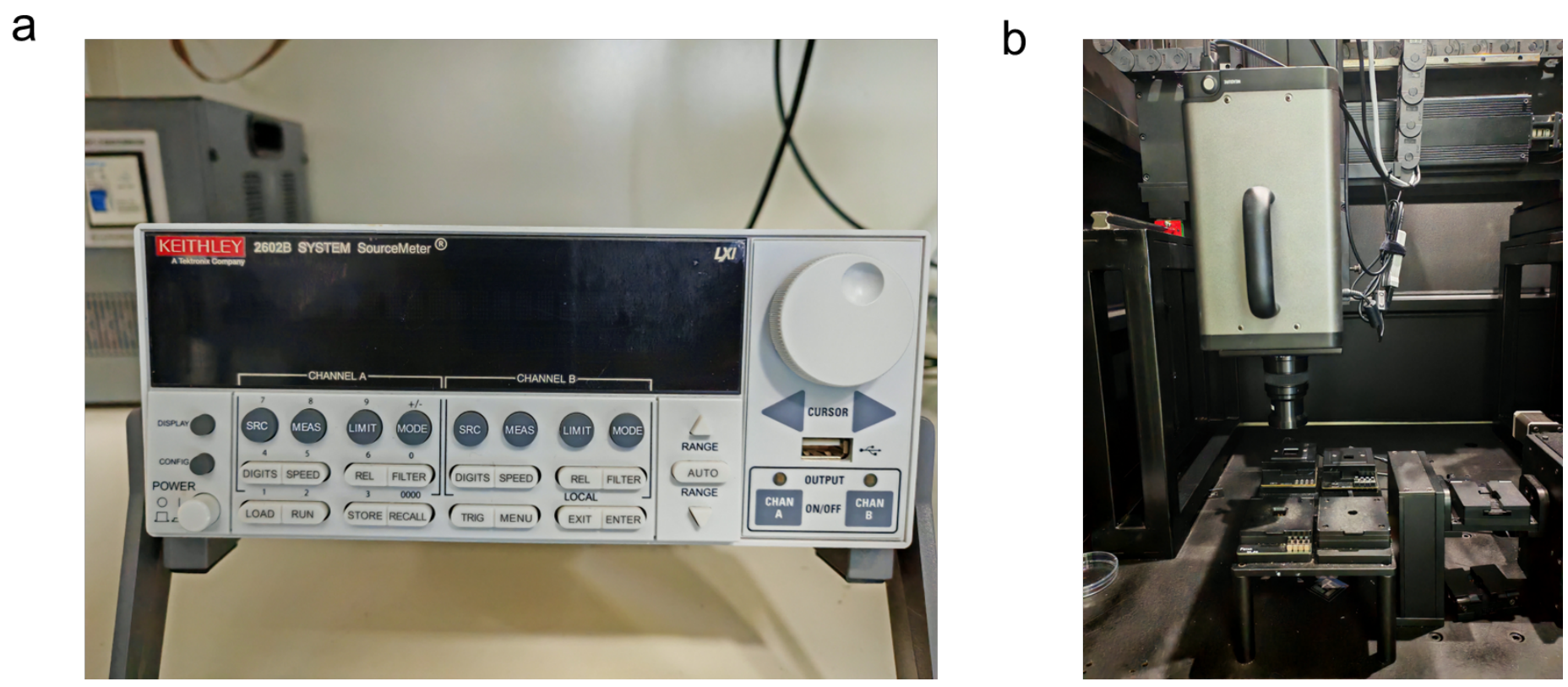

Fig. R20 The image of 2602B source meter (a) and CS2000 spectroradiometer (b).

Change made in the main text (Page 13)

Device encapsulation

First, using a UV curing packaging instrument, and package the already deposited QLED device with a glass cover plate in N_2 glove box. The specific process was as follows: a UV curing adhesive was pre-applied between the glass cover plate and the QLED device substrate, then placed in the packaging instrument, and UV irradiation with a duration of 15 s was applied to rapidly cross-link and solidify the UV curing adhesive, achieving a firm and airtight bond between the glass cover plate and the QLED device substrate.

Question v. The manuscript should include current efficiency (cd/A) data to correlate with EQE and confirm the radiance output.

Response: We appreciate the reviewer for highlighting the importance of current efficiency data to correlate with EQE and radiance output. Fig. R21 shows the detailed current efficiency (cd/A) versus current density characteristics (mA/cm^2) of the control and BN optimized device. **The figure clearly shows that the BN optimized device achieved a peak current efficiency of 125 cd/A at a current density of 1.10 mA/cm^2.**

It is worth noting that the shape and peak position of the current efficiency curve closely mirror the EQE profile presented in Fig. 5f (manuscript). This strong correlation directly confirms that the trend of quantum efficiency is consistent with the measured optical power output per electrical input.

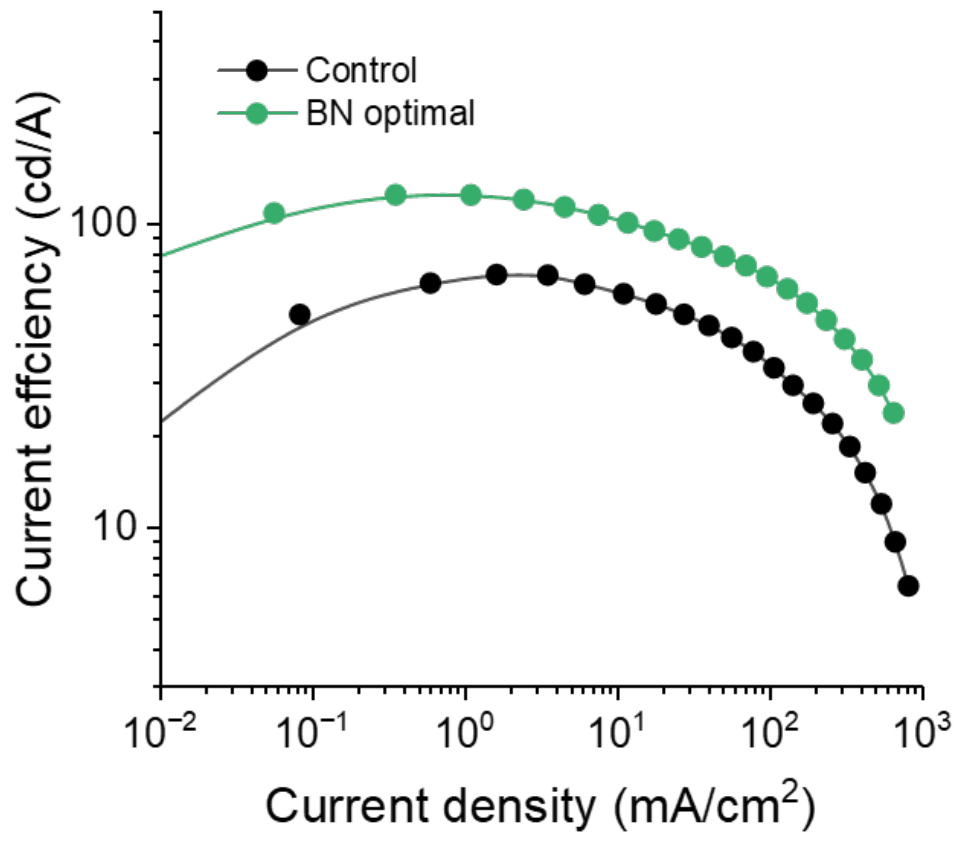

Fig. R21 The current efficiency of control and BN-optimized QLED.

Change made in the main text (Page 10)

After introducing BN, the QLEDs exhibited a significantly high maximum EQE of 32%, which is 1.7-fold higher than that of the control device (18.5%) (Fig. 5f and Supplementary Fig. 27) and had good reproducibility (Supplementary Fig. 28). Correspondingly, the BN-optimized device reaches a peak current efficiency of 125 cd/A, much higher than the control device with a peak current efficiency of 68 cd/A (Supplementary Fig. 29).

2. Device Performance: Lifetime Measurement

Question i. The manuscript claims that the Minolta CS-2000 was used for lifetime measurements. However, this spectroradiometer is not typically used for extended lifetime testing. Please explain in detail how this equipment was used to monitor degradation over thousands of hours.

Response: We sincerely appreciate the reviewer's questions. **In our manuscript, the CS-2000 spectroradiometer serves as the luminance acquisition device during the lifetime testing process.**

The key equipment for the lifetime testing of the devices in this manuscript includes a 2602B source meter (providing stable and accurate current and voltage for testing LEDs), a CS2000 spectroradiometer (utilized for high-precision measurement of the LED's luminance, and integrated software (responsible for controlling the current or voltage output of the 2602B source meter and the sampling interval of the CS2000 spectroradiometer. Additionally, it is used to collect and calculate photoelectric characteristics such as *I-V-L* during the testing process). The interface of the lifetime testing software is shown in Fig. R22.

The details of the lifetime test for the BN-optimized device at an initial luminance of

2000 cd/m^2 are as follows:

The LED was driven at a constant current density of 1.66 mA/cm^2, corresponding to an initial luminance (L_0) of ~2000 cd/m^2, as determined from the *I-V-L* characteristics. The luminance (L_t) was monitored in real time using a CS2000 spectroradiometer, while a 2602B source meter maintained the constant current output. The device lifetime (T_{50}) was defined as the elapsed time (t) until the luminance decayed to 50% of L_0 (i.e., 1000 cd/m^2). All data were recorded automatically via software.

It is also worth mentioning that our laboratory has two *I-V-L* and life testing instruments, which are measurement devices designed by Hangzhou Yuanfang Instrument Co., Ltd. and Suzhou Fushida Scientific Instruments Co., Ltd.. They can meet the requirements for long-term testing without affecting the overall progress of the laboratory.

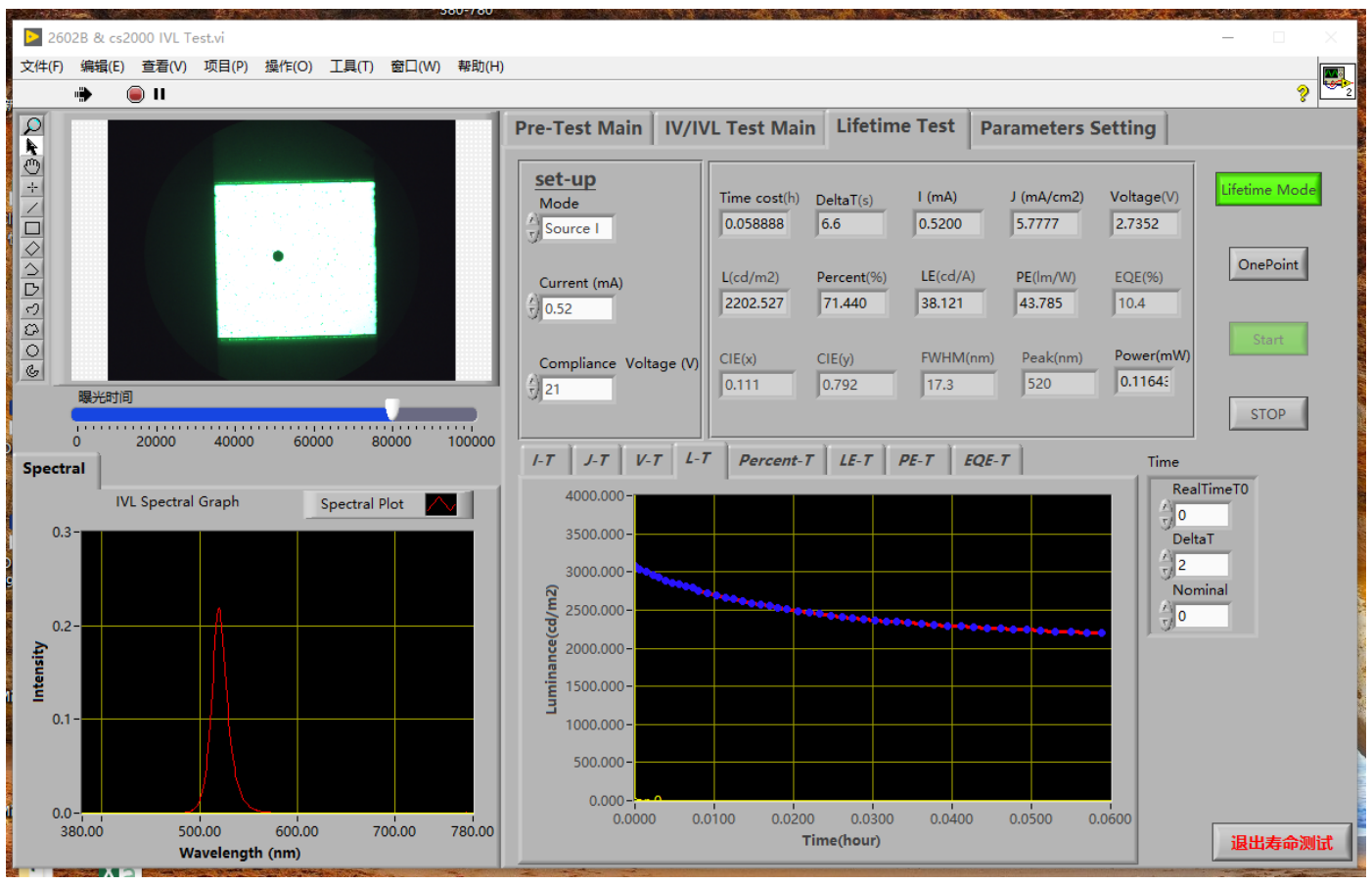

Fig. R22 The interface of the lifetime testing software.

Change made in the main text (Page 14)

Details of the lifetime test are as follows: Firstly, the initial luminance (designated as L_0) is measured using the CS2000. Subsequently, through the software, the 2602B source meter is configured to output a constant current. Simultaneously, the CS2000 is set to automatically measure the device's luminance (L_t). Concurrently, the curve of time (t) versus the corresponding luminance (L_t) is continuously recorded. When the real-time measured luminance L_t reaches approximately L_0×50%, the total elapsed test time (t) is recorded as the T_{50} lifetime of the LED. The test conditions are in an atmosphere with a temperature of 25 °C and a humidity of 25%.

Question ii. The authors must specify the test conditions, including ambient temperature, humidity, atmosphere (air or N_2), drive mode (constant current vs. constant voltage), and measurement intervals.

Response: Thanks to the reviewers' suggestions, it was our negligence not to provide detailed information on the testing conditions for lifetime in the manuscript.

In our manuscript, the lifetime of the devices was measured in an atmospheric environment with a temperature of 25 °C and humidity of 25%. Through software settings, the 2602B source meter output a constant current, while the CS2000 photometer automatically collected the brightness of the device. The test steps are as described in Question i.

Question iii. Complete lifetime curves (not just extrapolated values) from multiple samples should be included in the supplementary materials. In particular, the raw data for all points contributing to the extrapolated trend in Fig. 5h must be shown.

Response: We sincerely appreciate the reviewer's careful attention to the lifetime data presentation. We have provided the L/L_0 versus time data graph (Fig. R23) and the corresponding data (Table R3) for all data points in Fig. 5h (manuscript).

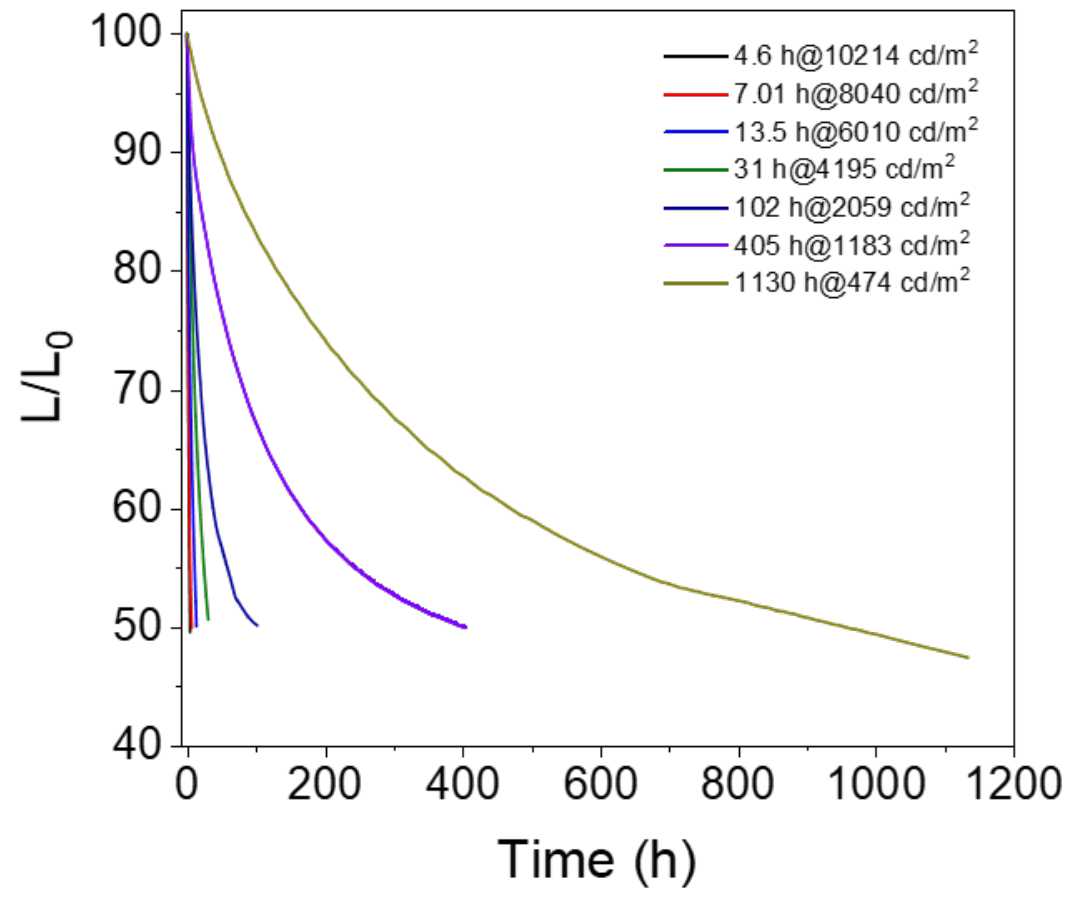

Fig. R23 The relationship between T_{50} and luminance in BN-optimized QLED accelerated lifetime experiments under different luminance.

Table.R3 Performance parameters of BN-optimized QLED under different current densities

Luminance (cd/m^2)	Current density (mA/cm^2)	Time (h)
10214	9.7	4.6
8040	7.32	7.01
6010	5.34	13.5
4195	3.42	31
2059	1.66	102
1183	0.81	405
474	0.41	1130

3. The experimental section lacks sufficient detail for reproducibility. The following information should be provided:

Question i. The thicknesses of all functional layers (e.g., ITO, PEDOT:PSS, PTAA, BN, QD layer, TPBi, Al), the preparation method of the BN solution, the molecular weight of PTAA, and other relevant details must be explicitly stated.

Response: Thank you for the valuable comments from the reviewers. We have measured the thickness of each functional layer using the cross-sectional TEM images of the LED device (Fig. R24). The results are as follows: the thicknesses of PEDOT:PSS, PTAA, BN, QD, ETL (TPBi/POT2T), LiF, and Al are approximately 30 nm, 20 nm, 1 nm, 35 nm, 40 nm, 1 nm, and 100 nm, respectively.

The details of the preparation of the PTAA solution are as follows: PTAA with a relative molecular weight of 15000-20000 is dissolved in chlorobenzene (heated at 40 °C and placed on a magnetic stirrer at 800 rpm for 8 hours to ensure complete dissolution and uniform mixing), and then the solution is filtered through an organic filter with a pore size of 0.25 μm before use.

The preparation method of BN solution is as follows: 0.1 g of BN nanosheets with an average size of ~20 nm is dispersed in 10 mL of anhydrous ethanol, then the mixture is subjected to ultrasonic treatment in an ultrasonic instrument for 2 hours to ensure complete dispersion. After ultrasonic treatment, the dispersion is centrifuged at 4000 rpm for 2 min in a centrifuge and the supernatant is carefully collected as the solution for preparing BN films.

All the relevant content has been incorporated into the manuscript.

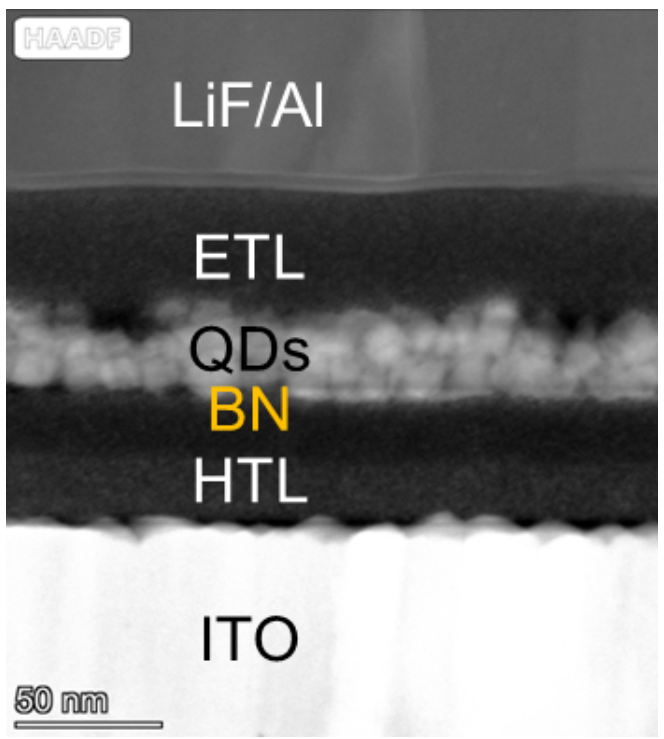

Fig. R24 The cross-sectional electron microscope image of the BN-optimized QLED.

Question ii. The deposition method for the QD layer (e.g., spin-coating parameters such as speed and time), annealing conditions (temperature and duration), precursor concentrations, and any post-treatments (e.g., ligand exchange, solvent annealing) must be described.

Response: Thank you for the reviewer's suggestion, the deposition method of QDs layer in

this work is as follows:

Using the QDs synthesis and purification process described in the method section, prepare a standard QDs solution (solvent: n-hexane). In a glovebox with N_2 atmosphere, spin coat at 3000 rpm (acceleration of 1500 rpm/s) for 45 s to form a film, followed by annealing on a hot plate at 80 °C for 5 minutes to remove the residual solvents, and form a QDs layer. The relevant content has been presented in the revised manuscript as follows:

Change made in the main text (Page 13)

In an N_2 atmosphere glove box, the PTAA, BN ethanol solution and QD layers were deposited layer by layer at a spin speed of 3000 rpm (acceleration 1500 rpm/s) for 45 s. Among them, the PTAA layer was annealed on a hot plate at 120 °C for 20 min to remove the residual solvents, while the BN layer and QDs layer were annealed on a hot plate at 80 °C for 5 min to remove the residual solvents.

Question 4. The HOMO/LUMO (or valence/conduction band) energy levels of each layer should be clearly labeled in Fig. 1d. Additionally, UPS measurements should be conducted for QD films with and without BN interlayers to verify band alignment changes due to BN passivation.

Response: Thank you to the reviewers for this constructive suggestion. We have made the following improvements to the energy level characterization section:

Firstly, in the revised Fig. 1d, we clearly marked the HOMO/LUMO energy level values of all functional layers (including the BN intermediate layer), as shown in Fig. R25.

Additionally, we supplemented the UPS spectra of QD and BN-optimized QD films, as well as the band gap of QDs, as shown in Fig. R26. From the figures, it can be seen that the HOMO energy level of the BN-optimized QD films increased from -5.76 eV in the QD films to -5.47 eV, indicating that the introduction of BN reduces the potential barrier of holes and helps the injection of holes. Furthermore, we also supplemented the testing method of UPS and the calculation method of HOMO/LUMO energy levels, as follows:

The UPS spectra were measured with a monochromatic He I light source (21.2 eV) and a Thermo Scientific ESCALAB 250 XI analyzer. A sample bias of -5 eV was applied to observe the secondary electron cutoff (SEC). The work function (Φ) can be determined by the difference between the photon energy and the binding energy of the secondary cutoff edge.

The energy level diagram of the device is calculated from the ultraviolet photoelectron

spectroscopy (UPS) data and according to the following formula:

$$V_{HOMO}=21.22-(E_{cutoff}-E_{onset})$$

where the incident photon capacity was 21.22 eV, E_{cutoff} was the secondary electron cutoff, and E_{onset} was the valence-band region.

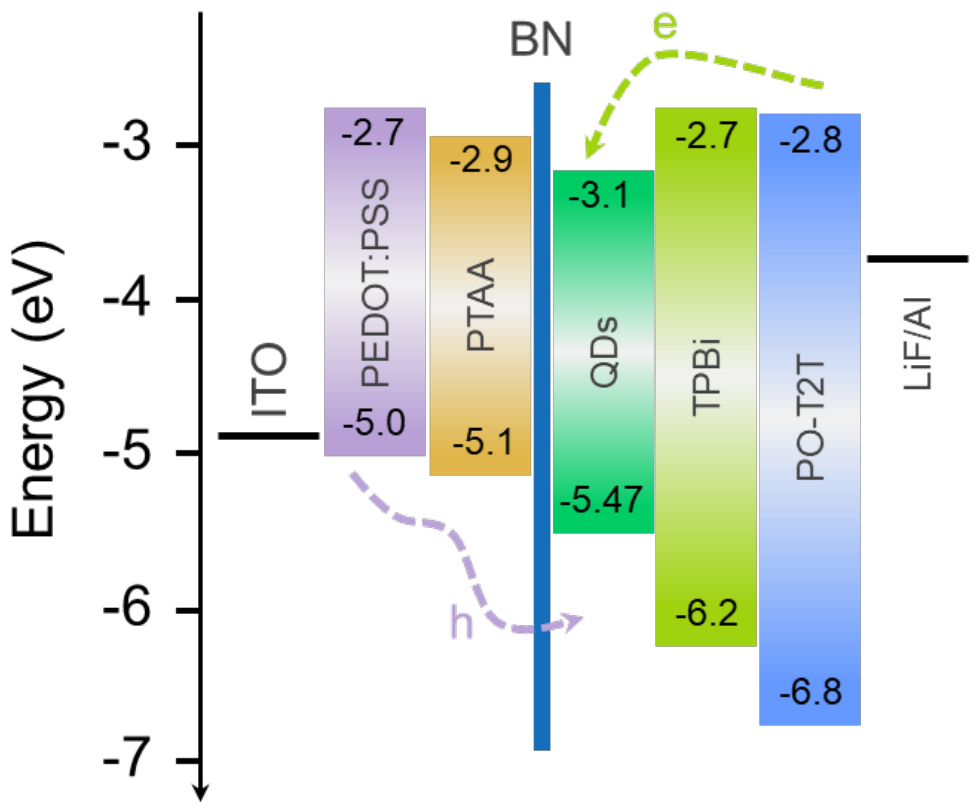

Fig. R25 The energy level diagram of the fabricated QLEDs device.

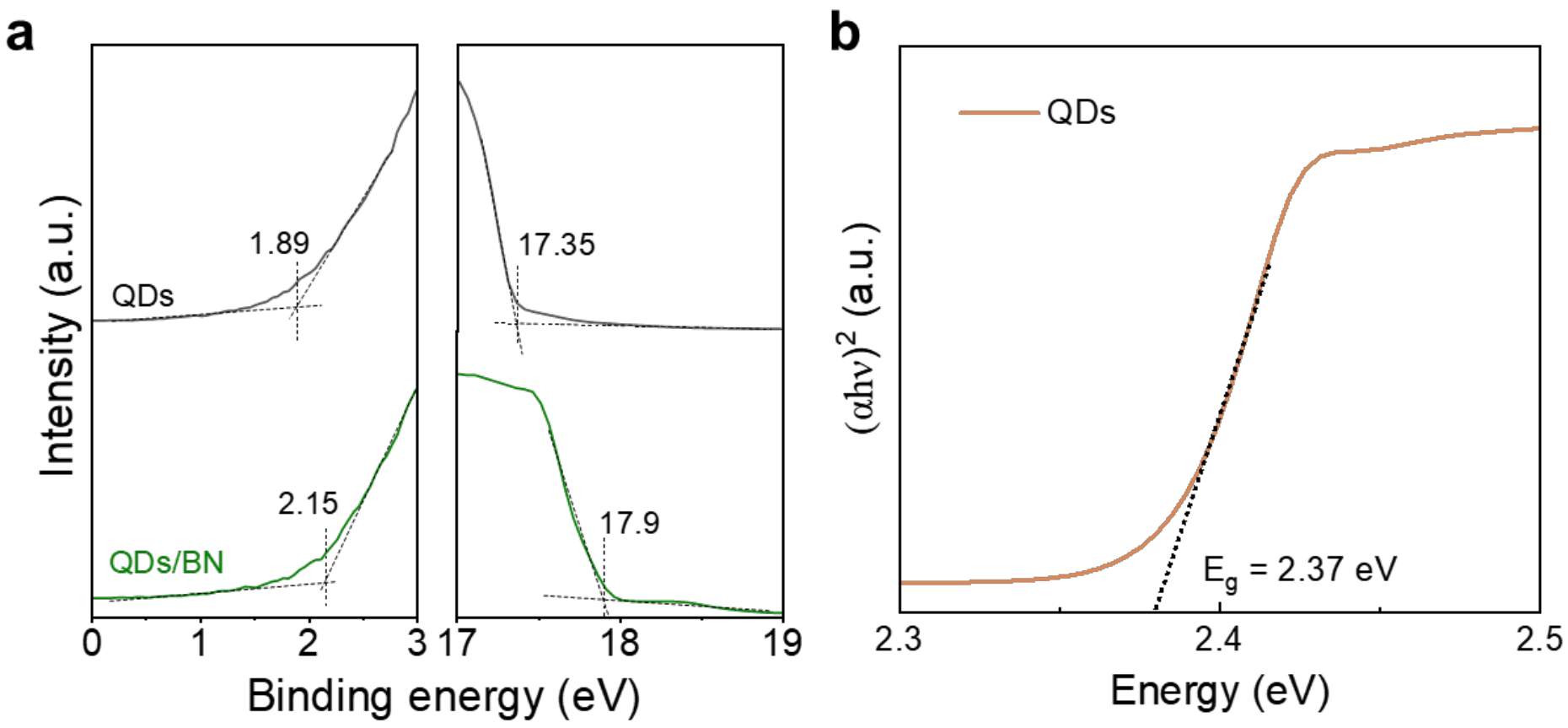

Fig. R26 (a) High-binding-energy secondary electron cutoff and valence-band edge regions of QDs and QDs/BN film measured by UPS, (b) Bandgap values of QD films from absorption spectra.

Question 5. Crystallographic planes such as (100) and (110) shown in GIWAXS (Fig. 2d) should be explicitly labeled in the figure and supported by simulations or XRD data.

Response: We appreciate the reviewer's insightful suggestion. As requested, we have clearly marked the (100) and (110) crystallographic planes in GIWAXS (Fig. 2d in manuscript), and provided XRD with the marked (100) and (110) crystallographic planes as support, as shown in the following figure:

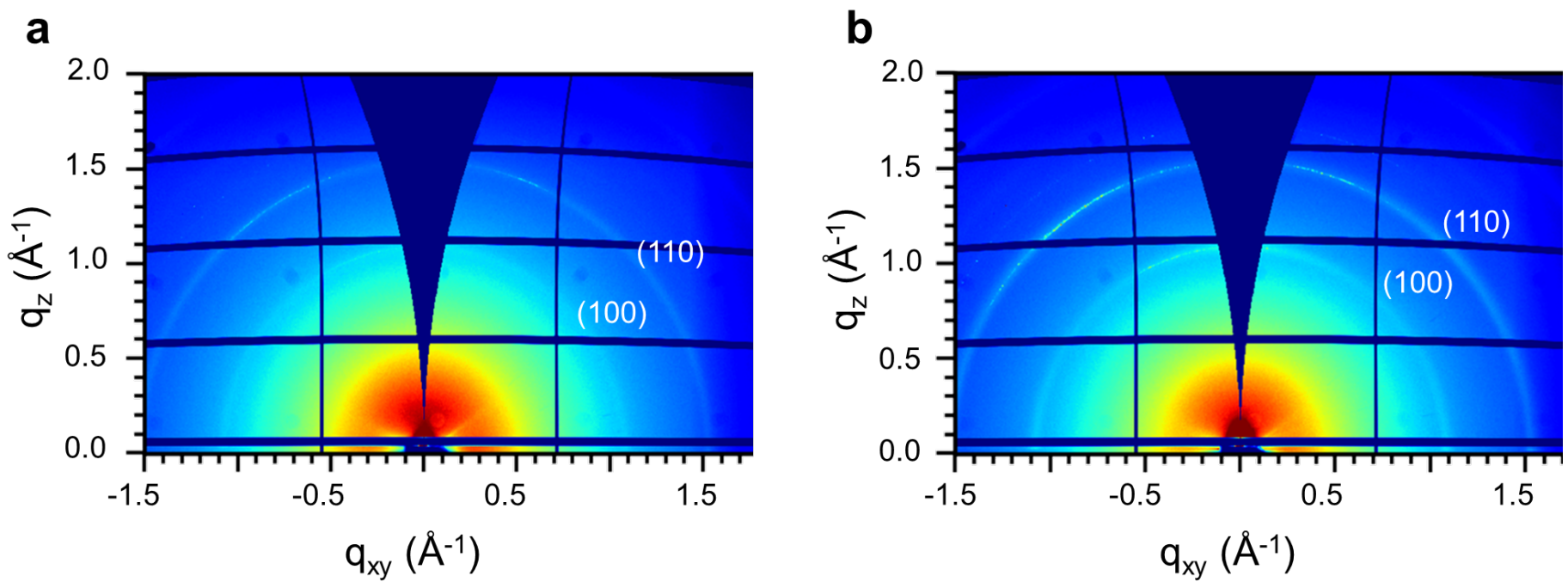

Fig.R27 GIWAXS 2D patterns of the control (a) and BN optimized QD films (b).

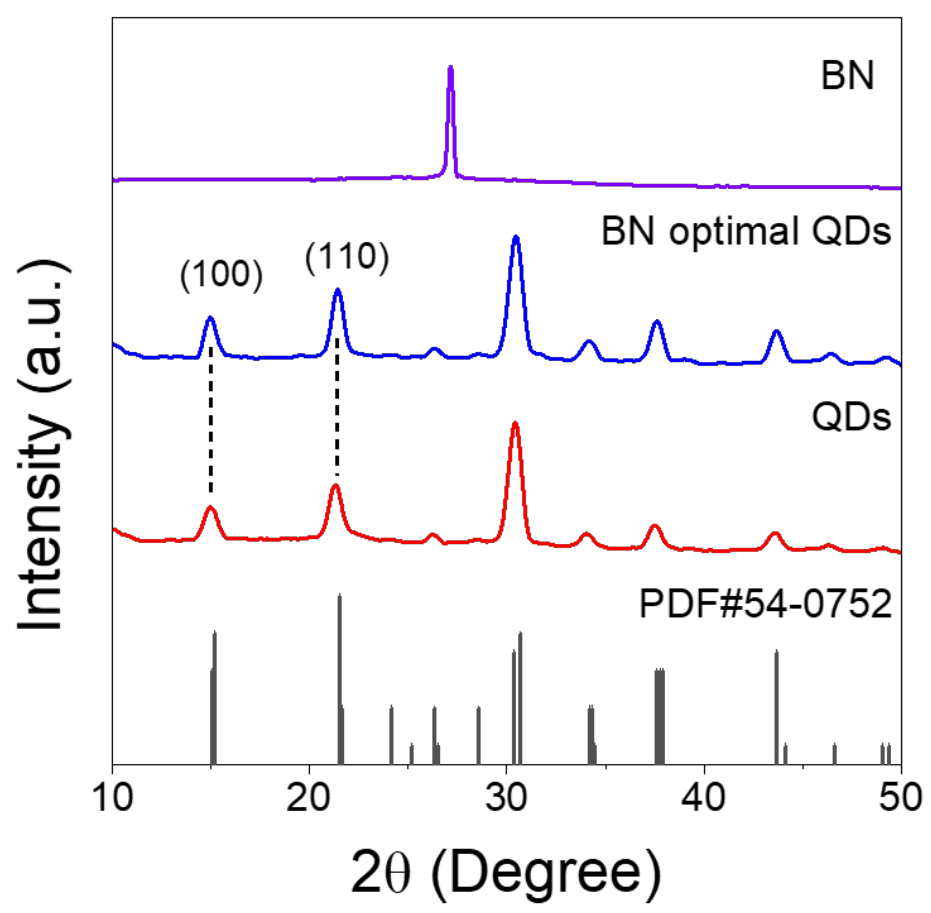

Fig.R28 The XRD spectra of QDs, BN-optimized QDs and BN films.

Question 6. In the temperature-dependent conductivity test (Supplementary Fig. 14), the schematic shows Au deposited on top of the QD layer. If BN is located beneath the QDs, it is unclear how it serves as an effective ion migration barrier. This experimental configuration requires clarification.

Response: Thanks to the reviewers' suggestions, here we need to clarify that when using this device for temperature-related conductivity tests, the main mechanism is to suppress the ion migration on the surface of the perovskite by the targeting effect of BN on the perovskite.

The DFT path simulation and ToF-SIMS depth profile results in the manuscript indicate that the introduction of the BN interlayer increases the migration barrier of Br^-, hindering the migration of Br^-. At the same time, XPS spectra, FTIR spectra (Fig. 2f and Supplementary Fig. 10), and DFT calculations show that BN undergoes chemical adsorption on the surface of perovskite QDs. Moreover, PL spectra, PL lifetime, TA spectra (Fig. 2i-j), and the defect density related to the hole-only device (Supplementary Fig. 13) indicate that the defect state of the optimized QDs by BN has been reduced. These results indicate that a targeting effect between BN and perovskite could passivate the defects of the perovskite and inhibit ion

migration.

Question 7. The methodology for preparing the PeLED samples for ToF-SIMS depth profiling is missing. Specifically, it should be explained how the two different PeLED samples were electrically aged before analysis.

Response: Thank you for the reviewer's suggestion, it was our oversight. In this work, before the ToF-SIMS deep analysis of QLEDs, the prepared QLEDs with or without BN interlayers need to be powered on at a luminance of 2000 for 40 min for electrical aging.

Change made in the main text (Page 14)

A ToF-SIMS (ION-ToF ToF-SIMS V) was used to depth-profile the perovskite QLED devices. Before the ToF-SIMS deep analysis of QLEDs, the prepared QLEDs with or without BN interlayers need to be powered on at a brightness of 2000 for 40 min for electrical aging.

Question 8. The claim that the ultrathin BN film significantly improves heat dissipation is questionable. Although BN has high intrinsic thermal conductivity, its effectiveness as a nanometer-scale film on a millimeter-thick glass substrate is likely negligible. The authors should provide thermal simulations or compare performance with a non-BN control under identical encapsulation conditions to substantiate this claim.

Response: Thank you for the comments and suggestions from the reviewers. We fully understand your concerns regarding the statement "ultrathin BN film significantly improves heat dissipation". Although the BN film is only at the nanometer-scale on the millimeter-thick glass substrate, in actual research, the unique layered structure of BN enables it to play a significant role in improving the heat dissipation of QLED even when it is extremely thin.

BN is a typical layered material. The layers are bonded together by relatively weak van der Waals forces, while the atoms within each layer are closely connected through covalent bonds. When QLED devices are operating, Joule heating generated by defects, non-radiative recombination processes, etc. will form heat accumulation points locally. Due to the strong interaction of the covalent bonds within the BN layers, it has excellent thermal conductivity, and the heat from these accumulation points can quickly disperse along the layers of the BN sheet.

In addition, in the actual BN film system, there is a possibility of overlap between the BN sheets. This overlap will form a continuous sheet network structure, further expanding the path of heat conduction. Heat can not only be conducted within a single BN sheet, but also be transmitted throughout the network through the overlapping areas between the sheets,

providing more channels and pathways for heat dissipation, which is conducive to more efficient conduction and dissipation of heat throughout the system, effectively preventing heat accumulation and significantly improving the heat dissipation performance of QLED.

Furthermore, we further employed an infrared-thermal-imaging-camera to monitor the effect of BN on Joule heating in operating QLEDs. As depicted in Fig. R29, the BN-optimized QLED exhibits a temperature of 43.6 °C after operation at 10000 cd/m^2 for 60 s, while the control devices experience a rapid temperature increase to 67.3 °C. This result confirms the reduced Joule heat generation and enhanced thermal dissipation properties of the BN-optimized films.

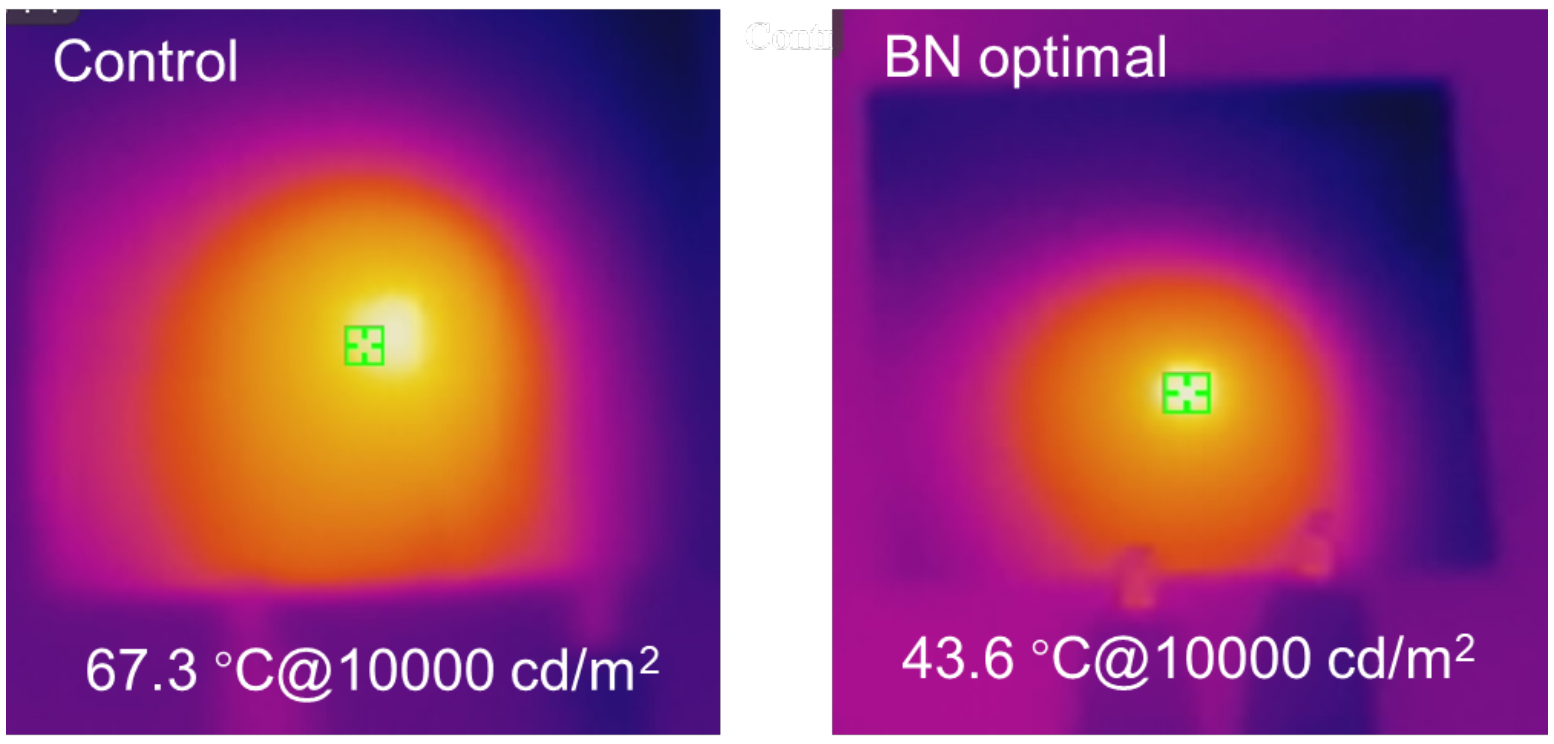

Fig. R29 Infrared thermal imaging images of control and BN optimized devices after operation at 10000 cd/m^2 for 60 s.

Question 9. Please provide luminance and EQE data as a function of driving voltage. For the BN-optimized device, data at a luminance level of 1 nit must be included to evaluate turn-on voltage.

Response: Thanks to the reviewers' suggestions, we have added the luminance and EQE data as a function of driving voltage. As shown in Fig. R30a, when BN is introduced, the corresponding turn-on voltage of the device decreases from 2.04 V to 1.96 V when the luminance reaches 1 nit.

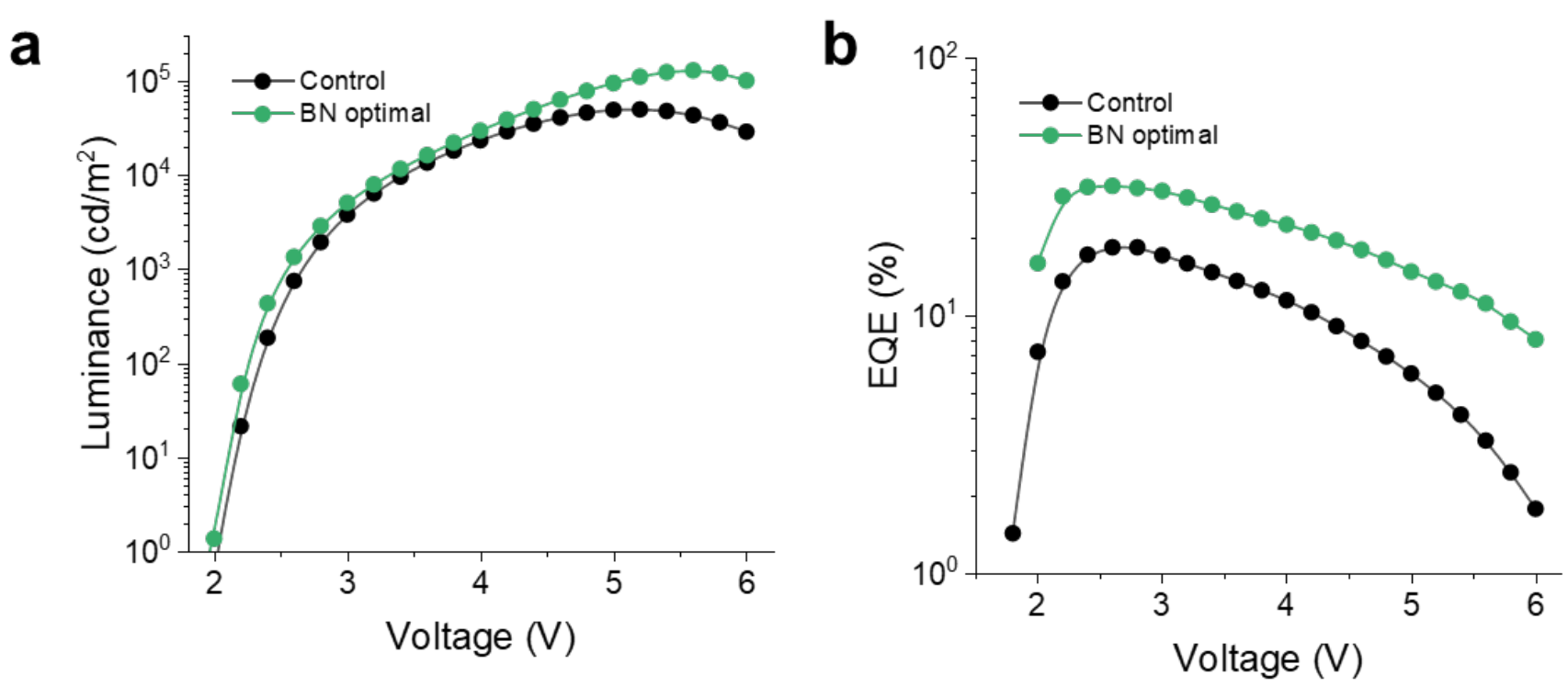

Fig. R30 The luminance (a) and EQE (b) data as a function of driving voltage.

Question 10. The device exhibits strong sub-bandgap electroluminescence (EL) below 2.0 V, despite the perovskite QD bandgap being approximately 2.4 eV. Such behavior is typically attributed to extremely thin or high-mobility transport layers. However, TPBi is known to have relatively low electron mobility. The authors should clarify the origin of this sub-bandgap emission. To determine whether this sub-bandgap emission originates from ultra-fast charge injection from the charge transport layers, the authors are encouraged to provide J–V curves of a control bipolar device composed only of PTAA and TPBi (without the QD layer). Additionally, device performance data using various HTL and ETL materials (such as PTAA with different molecular weights, Poly-TPD, PVK for HTLs, and PO-T2T, TmPyPB, etc. for ETLs) should be included.

Response: We would like to thank the reviewers for their meticulous comments.

First of all, we sincerely correct the description errors in the manuscript: the actual device adopts a TPBi/PO-T2T dual-layer electron transport structure, rather than a single TPBi layer. This correction is crucial for understanding the sub-bandgap electroluminescence mechanism. As the authors stated, sub-bandgap electroluminescence is usually attributed to extremely thin or high-mobility transport layers. Relatively, the electron mobility of TPBi is low (~10^{-5} $cm^2/V{\cdot}s$), making it impossible to achieve this sub-bandgap electroluminescence. While the electron mobility of PO-T2T (1.7 x 10^{-3} $cm^2/V{\cdot}s$) is higher than that of TPBi, the TPBi/PO-T2T dual-layer electron transport layer could improve the efficiency of injecting electrons into the QD layer, thereby achieving sub-bandgap emission. Meanwhile, as we previously reported (*Science Bulletin* 2021, 66, 36), only high-mobility PO-T2T can also achieve sub-bandgap electroluminescence, and the turn on voltage is slight lower than that of TPBi/PO-T2T dual-layer electron transport layer.

To determine whether this sub-bandgap emission is due to the ultrafast charge injection in the charge transport layer, we provided the *J-V* curves of a control bipolar device consisting only of PTAA and TPBi, TPBi/PO-T2T (without the QD layer) (Fig. R31). According to the results of R31, the introduction of PO-T2T can significantly improve the carrier injection in the device, causing rapid carrier injection to occur near 1.5 V (sub-bandgap) in the device. This also suggests that the introduction of high-mobility PO-T2T in the charge transport layer is curial for the sub-bandgap luminescence in our device.

In addition, we also provided the device performance data using various HTL and ETL materials. Specifically, we compared the device performance of different molecular weight PTAA and poly-TPD used as HTLs. As shown in Fig. R32, compared to PTAA with a relative molecular weight of 5W (EQE_{max} = 26.4%), when the molecular weight is 2W, it has a slightly higher current density, and the EQE_{max} = 31.3%. While, the poly-TPD is used as HTL, it has a slightly lower current density, and the EQE_{max} = 19.3%. Moreover, compared with

poly-TPD (2.1 V), the turn-on voltage of PTAA (1.96 V) as the HTL is lower. The results indicate that PTAA, with a higher mobility, is more suitable as the HTL in this work.

In addition to testing different HTL, we also investigated the device performance using pure TmPyPB (1.0×10^{-3} $cm^2/V\cdot s$) and PO-T2T as the ETL, as shown in Fig. R33. The turn-on voltages of the TmPyPB, and PO-T2T devices are 1.94 V, and 1.91 V, respectively. However, their EQE_{max} is relatively low (18.7% for TmPyPB and 22% for PO-T2T). These results indicate that the combination of PTAA and TPBi/PO-T2T can achieve a better balance of carrier injection, thereby enabling more efficient recombination.

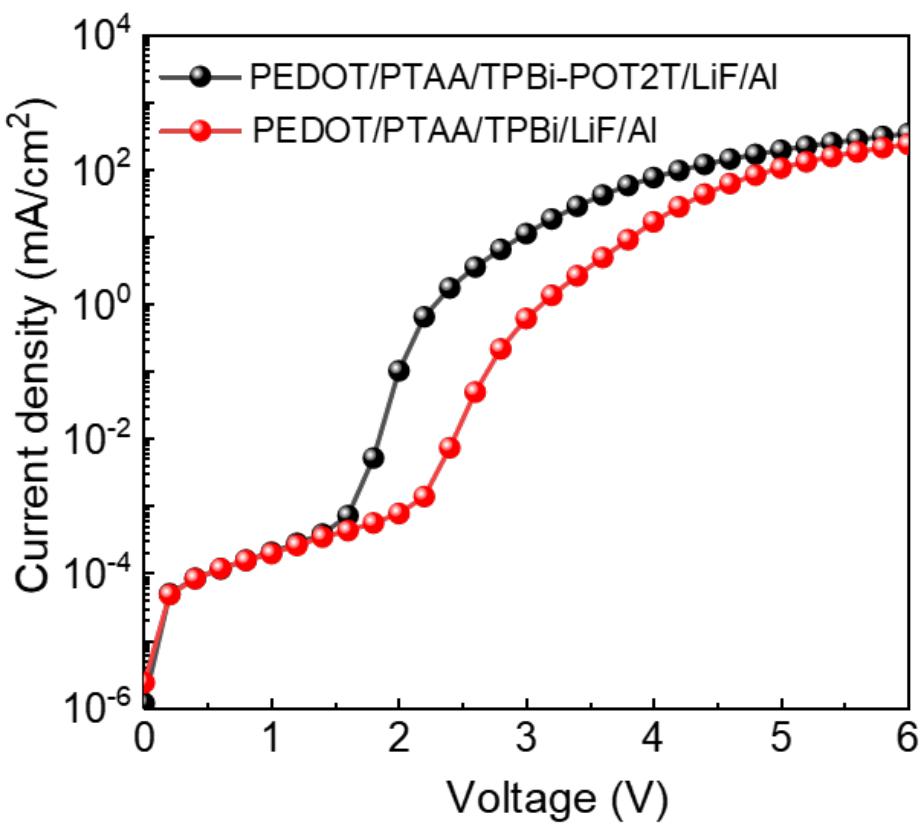

Fig. R31 The *J-V* curve of PEDOT/PTAA/TPBi-POT2T/LiF/Al and PEDOT/PTAA/TPBi/LiF/Al device.

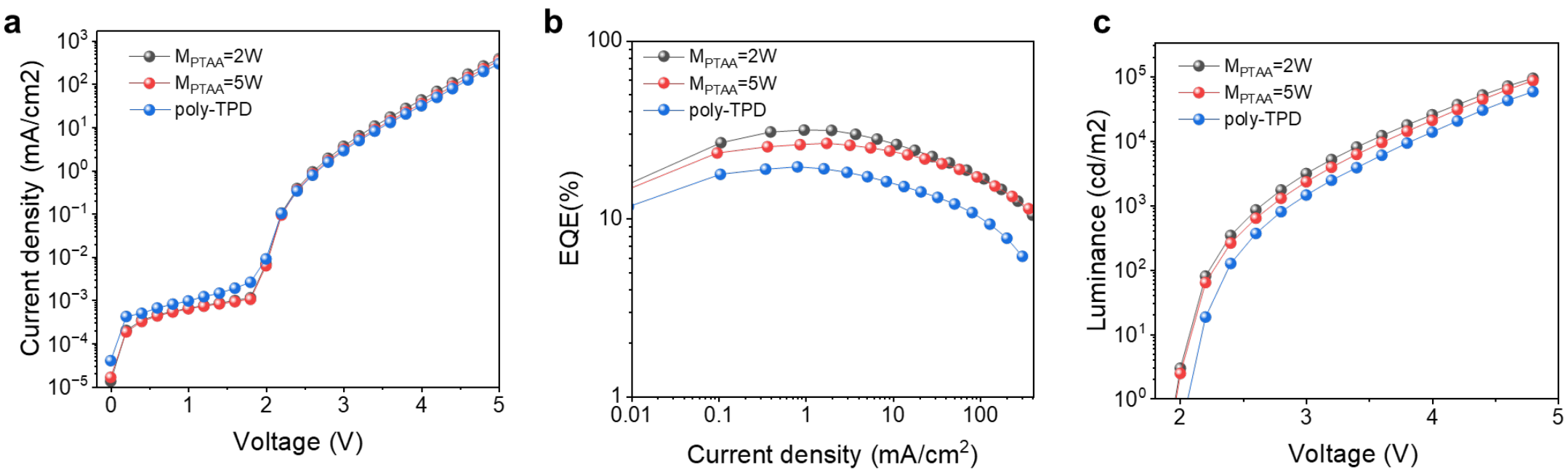

Fig. R32 Performance comparison of QLEDs with different HTLs.

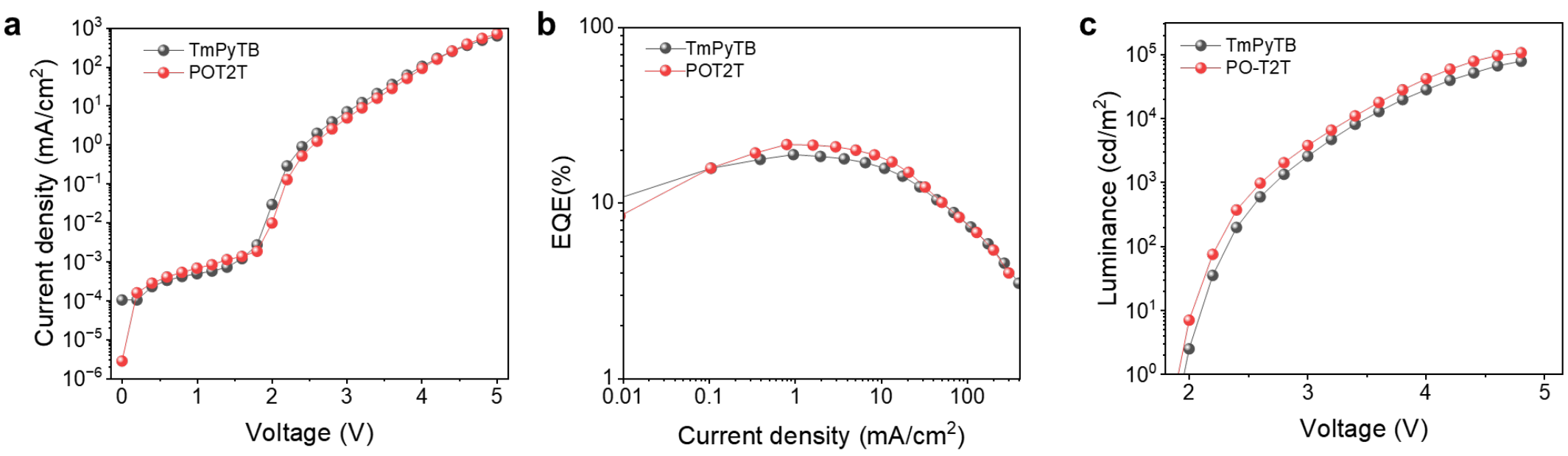

Fig. R33 Performance comparison of QLEDs with different ETLs.

Response to reviewer 1

General Comment: The authors have revised the paper reasonably well by addressing the issues I raised in the last round of review. However, I believe that some of the key points are still not resolved, particularly in relation to the reliability of device lifetimes. Currently the data reporting and description of experimental methods are vague, raising important concerns on the validity of the main conclusions.

Response: We sincerely appreciate the reviewer's insightful comments regarding the reliability of device lifetime data. We fully agree that clarifying the experimental methodology is critical to strengthening the validity of our conclusions. Therefore, we have added more detailed information about the QLED preparation and performance testing in the revised version.

Change made in the main text (Page 13)

Device construction

The patterned ITO was treated with deionized water, isopropyl alcohol and acetone by ultrasonic treatment for 15 mins, and then treated with ultraviolet-ozone for 15 mins before use. PEDOT:PSS were filtered by 0.25 μm filter, and then spin-coated onto the ITO coated glass substrate at 3000 rpm for 45 s and then heat at 120 °C for 15 mins. Subsequently, it was transferred to an N_2 atmosphere glovebox. The PTAA solution (5 mg/mL) was spin-coated onto the PEDOT:PSS with a spin speed of 3000 rpm (with an acceleration of 1500 rpm/s) for 45 s. The substrates were then heated at 120 °C for 20 mins to remove the residual solvents. After the films had cooled to room temperature, the BN ethanol solution (0.2 mg/mL, 0.5 mg/mL, 1 mg/mL, 2 mg/mL) was spin-coated onto the PTAA with a spin speed of 3000 rpm (with an acceleration of 1500 rpm/s) for 45 s. The substrates were then heated at 80 °C for 5 mins. After the films had cooled to room temperature, the QDs solution (0.1 mol/L) was spin-coated onto the BN layer with a spin speed of 3000 rpm (with an acceleration of 1500 rpm/s) for 45 s. The substrates were then heated at 80 °C for 5 mins. After annealing, the films were moved to a thermal evaporation system under a vacuum of ~5×10^{-4} Pa to sequentially deposit TPBi (5 nm)/PO-T2T (35 nm) and LiF/Al electrodes (1 nm/100 nm). The light-emitting area of the device was 9 mm^2 as defined by the overlapping area of ITO and Al electrodes.

Change made in the main text (Page 14)

Device performance test

The performance of the encapsulated QLEDs were conducted using the testing system provided by Suzhou Fushida Scientific Instrument Co., Ltd. (Model 2602B source meter and CS-2000 spectrophotometer). Firstly, the luminance-current-voltage (*L*-*J*-*V*) curve was measured by sweeping the voltage from 0 to 6 V with a step of 0.2 V to determine the initial luminance (L_0) and the corresponding current density. Then, with this current as the constant driving condition, the luminance decay (L_t) was monitored in real time using the CS-2000. The test was stopped when L_t dropped to 50% of L_0, and the total time (T_{50}) was recorded as the lifetime. The test conditions are in an atmosphere with a temperature of 25 °C and a humidity of 25%. The data was automatically collected and analyzed by the accompanying software. All QLEDs were tested after being encapsulated in N_2.

Question 1. The authors should make it very clear that the lifetime of 25263 hours at 100 cd/m^2 was extrapolated in various places in the paper, including abstract, introduction, conclusion, Fig. 5h and Supplementary Table 3. This is to avoid potential misleading claims.

Response: We sincerely appreciate the reviewer's valuable comment regarding the clarity of the lifetime extrapolation method. We fully agree that explicitly stating the extrapolated nature of the 25,263 hours lifetime at 100 cd/m^2 is essential to ensure transparency and avoid potential misinterpretation. The modifications to the abstract, introduction, conclusion, Figure 5h, and Supplementary Table 3 are as follows:

Change made in the main text (Page 2)

BN-optimized QLEDs achieved an average EQE of 30.05% and extrapolated operational lifetime T_{50} of 25,263 hours at an initial luminance (L_0) of 100 cd/m^2

Change made in the main text (Page 5)

the champion BN-optimized QLEDs achieved an average EQE of 30.05% and extrapolated operational lifetime T_{50} (defined as the time for the luminance to decrease to half of the initial luminance) of 25,263 hours at an initial luminance (L_0) of 100 cd/m^2

Change made in the main text (Page 11)

it can be determined that the extrapolated lifetime of BN-optimized QLEDs at 100 cd/m^2 is 25,263 hours

Supplementary Table 3. Comparison of high-performance green perovskite LEDs.

Device structure	EQE_{max} (%)	T_{50} (h)	Ref.
ITO/PEDOT:PSS/Perovskite NCs/TPBi/LiF/Al	26.7	~3[a] (L_0=100 cd/m^2)	*ACS Energy Lett*. 2023, **8**, 927
ITO/Buf-HIL/Perovskite NCs/ZADN/TPBi/BCP:Li/MoO_3/CBP/TAPC/ZADN/LiF/Al)	37	5596[a] (L_0=100 cd/m^2)	*Nat. Nanotechnol.* 2024, **19**, 624
ITO/$Ni_{0.9}Mg_{0.1}O_x$/PVK/PEG/2D Perovskite/TPBi/LiF/Al	30.84	1.68[*] (L_0=100 cd/m^2)	*Adv. Mater.* 2023, **35**, 2302283
FTO/GraHIL/3D Perovskite @BPA/ZADN/LiF/Al	28.9	31808[a] (L_0=100 cd/m^2)	*Nature* 2022, **611**, 688
ITO/NiO_x/PTAA/PVK/0D(3D) Perovskite/TPBi/LiF/Al	31	-	*Adv. Mater.* 2024, **36**, 2406706
ITO/PEDOT:PSS:PFI/RDPs/3TPYMB/LiF/Al	25.6	1.92[*] (L_0=7200 cd/m^2)	*Nature* 2021, **599**, 594
ITO/PEDOT:PSS/PTAA/Perovskite NCs/TPBi/PO-T2T/LiF/Al	26.2	10[*] (L_0=100 cd/m^2)	*Nat Electron* 2024, **7**, 487
ITO/PMMA/PTAA/Perovskite/POT2T/LiF/Al	28.3	-	*Nat. Mater.* 2025, **24**, 778
FTO/NiOx/PVP/Perovskites/TmPPPyTz/LiF/Al	29.5	18.67[*] (L_0=12000 cd/m^2)	*Nat. Photon.* 2024, **18**, 363
ITO/Me-4PACz/PVK/Perovskite/ETL/LiF/Al	22	185,600[a] (L_0=100 cd/m^2)	*Nature* 2025, **643**, 96
FTO/GraHIL/Perovskite NCs/ZADN/LiF/Al	28.9	520[*] (L_0=1000 cd/m^2)	*Nature* 2022, **611**, 688
ITO/PEDOT:PSS/PTAA/Perovskite QDs/TPBi/LiF/Al	18.6	213[a] (L_0=100 cd/m^2)	*Adv. Funct. Mater.* 2024, **34**, 2308341
ITO/PEDOT:PSS/PTAA/Perovskite QDs/TPBi/PO-T2T/LiF/Al	21.63	180.1[*] (L_0=100 cd/m^2)	*Sci. Bull.* 2021, **66**, 36
ITO/PEDOT:PSS/PTAA/Perovskite QDs/3P/LiF/Al	30.2	3220[a] (L_0=100 cd/m^2)	*Angew. Chem. Int. Ed.* 2024, **63**, 202410689
ITO/PEDOT:PSS/PTAA/Perovskite QDs/TPBi/PO-T2T/LiF/Al	20.3	127[*] (L_0=100 cd/m^2)	*Adv. Optical Mater.* 2024, **12**, 2302253
ITO/PEDOT:PSS/Poly-TPD/Perovskite QDs/TPBi/LiF/Al	23.45	0.02[*] (L_0=100 cd/m^2)	*ACS Energy Lett.* 2023, **8**, 4386
ITO/PEDOT:PSS/Buf-HIL/$FA_{1-x}GA_xPbBr_3$/TPBi/LiF/Al	23.4	2.2[*] (L_0=100 cd/m^2)	*Nat. Photonics* 2021, **15**, 148
ITO/PEDOT:PSS/PTAA/$CsPbBr_3$ QDs/TPBi	24.7	182[a] (L_0=100 cd/m^2)	*Appl. Phys. Rev.* 2024, **11**, 031405
**ITO/PEDOT:PSS/PTAA/BN/Perovskite QDs/ETL/LiF/Al**	**32**	**25263[a] (L_0=100 cd/m^2)**	**This work**

Notes, [*] represents the real measured lifetime, and [a] represents the extrapolated lifetime.

Question 2. According to Fig. 5g, the luminance of the sample "BN optimal" degraded more rapidly in the beginning and then slowed down after about 80 hours. What happened after 80 hours? Did the device more rapidly degrade or it lasted for much longer? The degradation curve of BN devices seems to be much smoother compared to the "control". Can the authors show the data points rather than a smooth curve?

Response: Thank you for your profound and constructive comments on the content of Fig. 5g. These comments are crucial for us to present the research results more comprehensively and accurately, and for readers to deeply understand the degradation behavior of the samples. Here is a detailed reply to the points you have raised.

Regarding the reasons for "*the luminance of the sample "BN optimal" degraded more rapidly in the beginning and then slowed down after about 80 hours*"

As shown in Fig. 5g, the luminance of the BN optimized QLED indeed exhibits a two-stage characteristic of "rapid decay → slow decay", with the transition occurring around 80 hours. We speculate that this is associated with the time required for the dynamic equilibrium of a small number of defects and the equilibrium of ion migration under an applied electric field: at the initial stage of external voltage application, the defects and ion migration in the device lead to a rapid drop in luminance; as time elapses, the surface defects gradually reach a dynamic equilibrium, and the ion migration process under the applied electric field also tends to be stable. The kinetics of equilibrium establishment for these two processes collectively govern the stage-wise transition of the luminance decay rate in the device.

After 80 hours, when the surface defects gradually reach dynamic equilibrium and the ion migration process under an external electric field tends to stabilize, the device lifetime also shows a slow and stable decrease (Fig. R1). Furthermore, to ensure the reliability of the data, **we entrusted the Shen Huibin's group from Henan University to verify it**. Choosing to conduct cross-validation in this research group is mainly because the Shen's group has been deeply engaged in the field of QD for nearly 20 years, achieving significant results and having a considerable influence (such as *Nature* 2024, 635, 854; *Nat. Photon.* 2024, 18, 186; *Nat Commun* 2025, 16, 10143, etc.). The related verification results are highly persuasive. At the same time, our perovskite emitter is in a nanocrystal form, which is highly in line with the research system of Shen's group, ensuring the rationality of the verification. As shown in Fig. R2, at an initial luminance of 2000 cd/m^2, the verified lifetime T_{50} of this device is 99.7 hours, and after 80 hours, the lifetime also shows a slow and stable decline, which is consistent with our test trend.

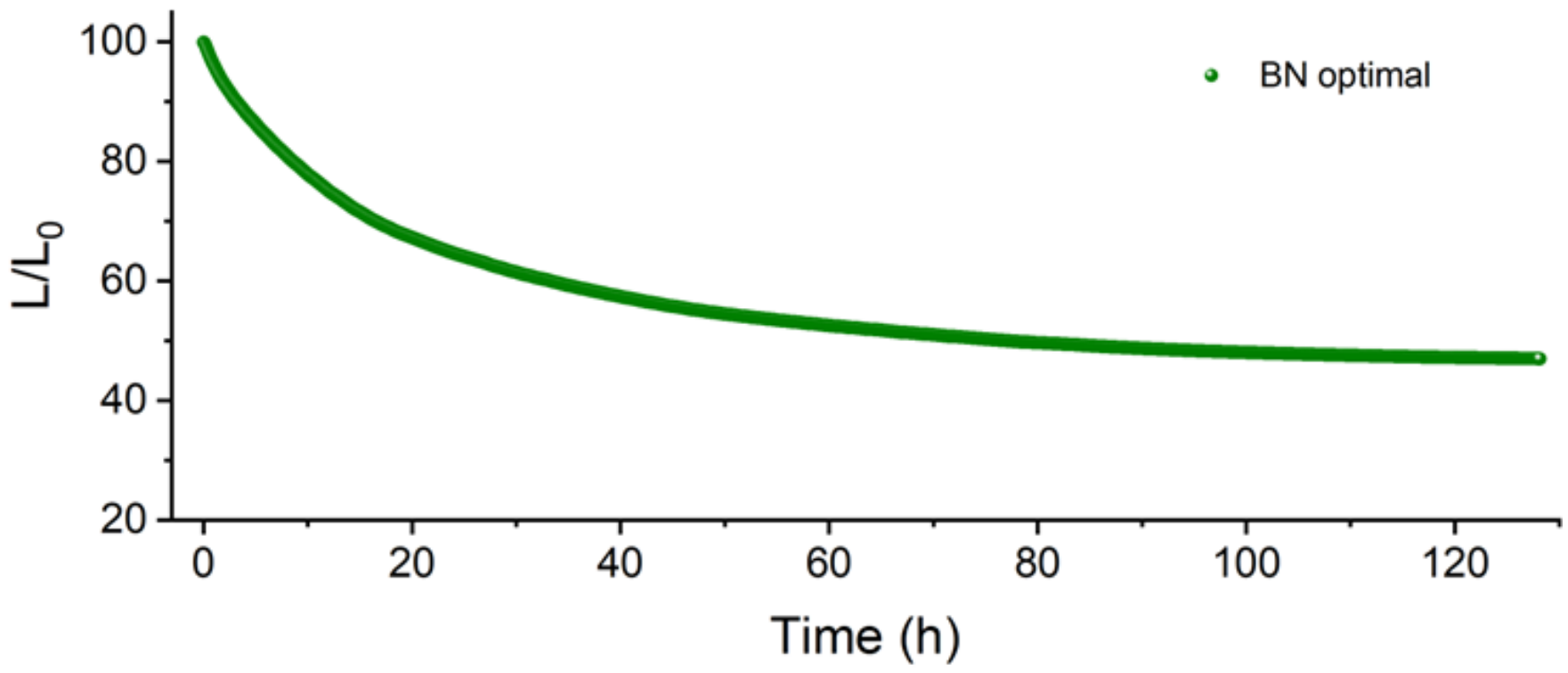

Fig. R1 The operation lifetime of BN-optimized QLEDs under an initial luminance of 2000 cd/m^2.

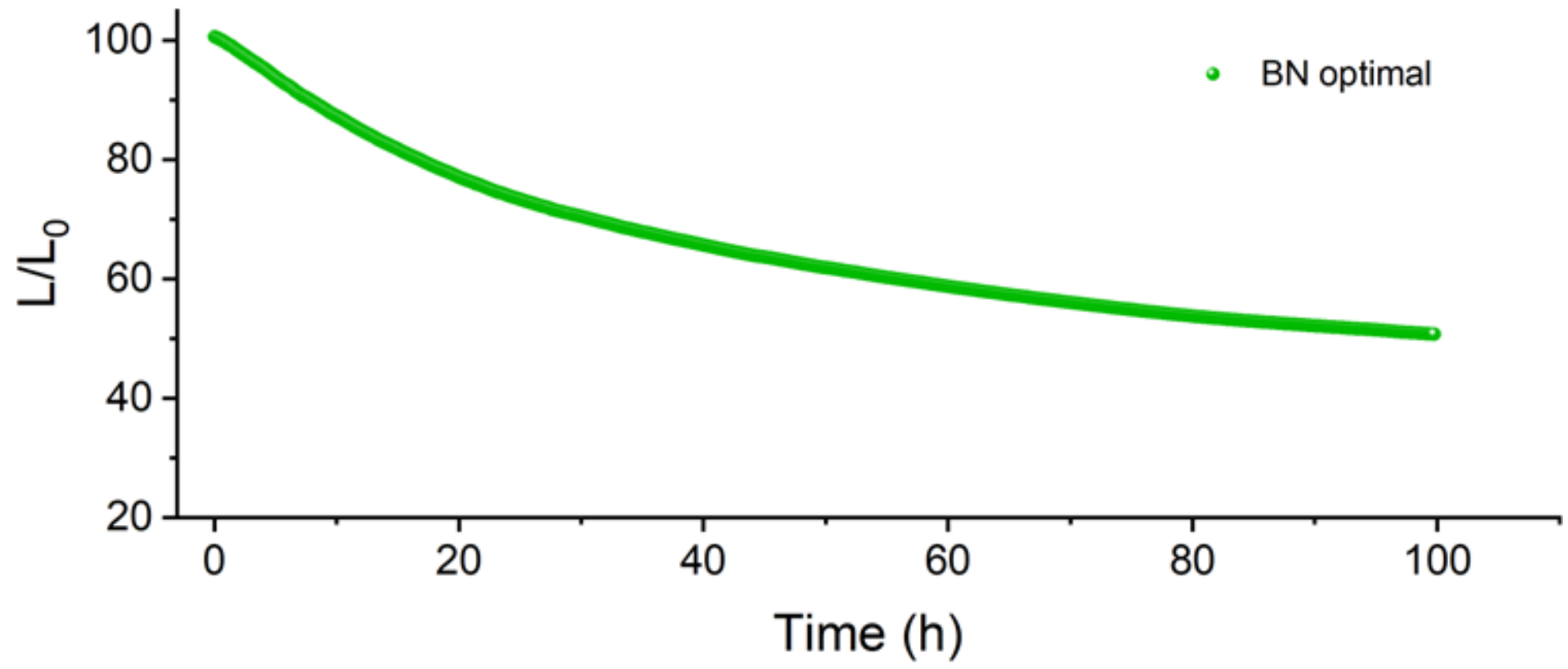

Fig. R2 The operation lifetime of BN-optimized QLEDs under an initial luminance of 2000 cd/m^2 from other labs.

You mentioned that you would like us to show the data points instead of the smoothed curve. We fully understand the significance of this suggestion. Presenting the original data points enables readers to directly observe the experimental data and assess the variability and reliability of the results. Therefore, we have supplemented the figure with the original data points, as shown in Fig. R3.

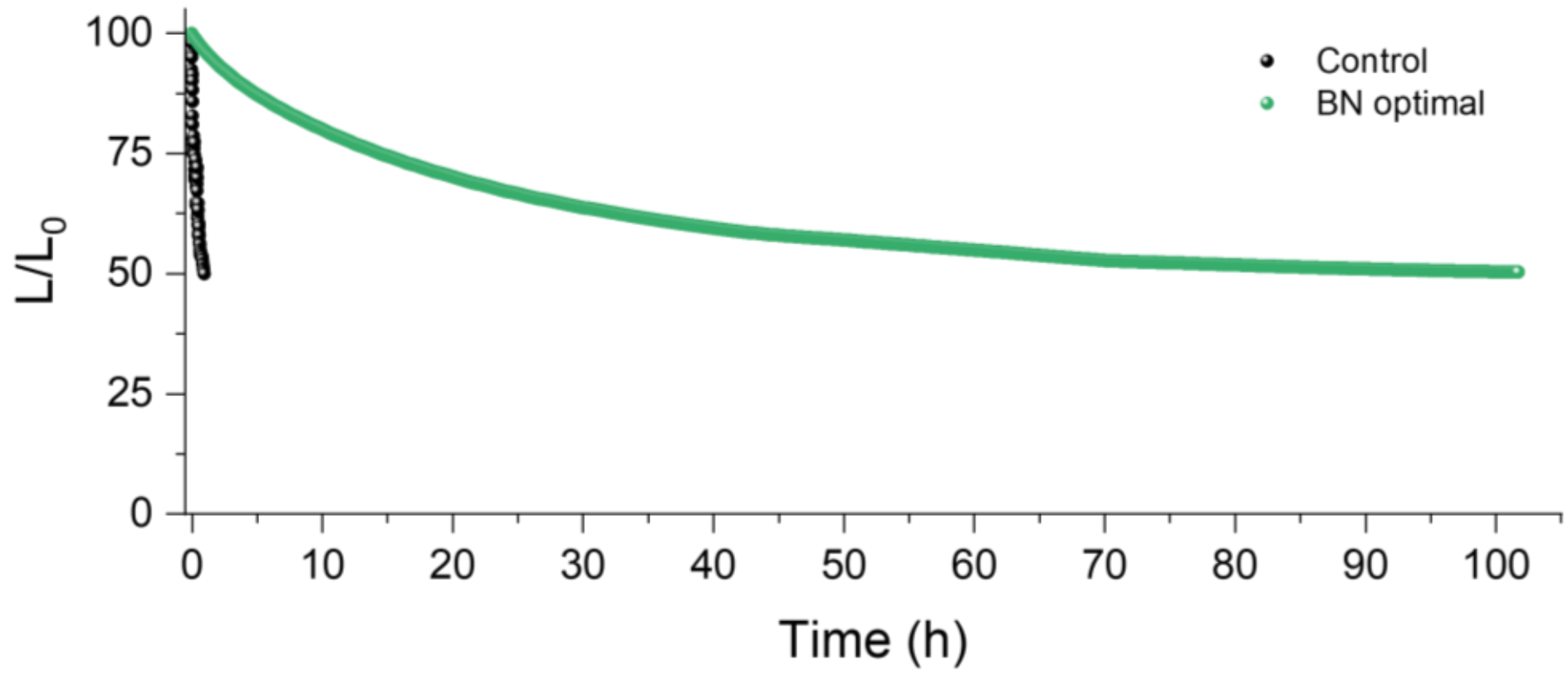

Fig. R3 The operation lifetime of QLEDs based on control and BN-optimized QLEDs under an initial luminance of 2000 cd/m^2.

Question 3. In response to Reviewer #3's comments, the authors say:

"The LED was driven at a constant current density of 1.66 mA/cm^2, corresponding to an initial luminance (L_0) of ~2000 cd/m^2, as determined from the *I-V-L* characteristics. The luminance (L_t) was monitored in real time using a CS2000 spectroradiometer, while a 2602B source meter maintained the constant current output. The device lifetime (T_{50}) was defined as the elapsed time (t) until the luminance decayed to 50% of L_0 (i.e., 1000 cd/m^2). All data were recorded automatically via software. It is also worth mentioning that our laboratory has two *I-V-L* and life testing instruments, which are measurement devices designed by Hangzhou Yuanfang Instrument Co., Ltd. and Suzhou Fushida Scientific Instruments Co., Ltd.. They can meet the requirements for longterm testing without affecting the overall progress of the laboratory."

It should be noted that the authors basically mentioned two different systems for lifetime measurements. In the methods section, they only mention the CS2000 spectrometer which was alerted by reviewer #3 on whether this system is suitable for measuring long device lifetimes. Later in their response, the authors mention that they also use experimental setups designed by Hangzhou Yuanfang Instrument Co., Ltd. and Suzhou Fushida Scientific Instruments Co., Ltd. However, details of these are completely missing in the paper.

So, which of the two systems were actually used for the lifetime experiments? What were the differences between the two systems (which one is more reliable for what purposes)? Please be specific so that the readers can see which figure was obtained using which setup. Such details must be provided clearly in the paper.

Response: Firstly, we sincerely apologize for any misunderstandings caused by our previous response and for the omission of equipment information in the paper.

In our previous response, we provided the specific operation procedures for the lifetime test and mentioned that "our laboratory has two *I-V-L* and lifetime testing instruments, which are measurement devices designed by Hangzhou Yuanfang Instrument Co., Ltd. and Suzhou Fushida Scientific Instruments Co., Ltd.. They can meet the requirements for long term testing without affecting the overall progress of the laboratory", **indeed our research group has two testing devices that can test stability, namely the CS2000 photoelectric performance testing system and the multi-channel LED lifetime-specific testing equipment.** Generally speaking, the CS2000 photoelectric performance testing system with comprehensive data dimensions can simultaneously monitor the evolution patterns of key parameters such as luminance, EQE, and spectrum over time. However, due to the hardware limitations of the single-substrate testing position, **CS2000 system is mainly** used to test the performance of the devices and obtain multi-dimensional performance data of blue and red perovskite QLEDs. Occasionally, **the CS2000 system is also** used to test the stability verification of the core experimental group of green perovskite QLEDs to obtain multi-dimensional performance attenuation data. The multi-channel LED lifetime-specific testing equipment is equipped with 16 independent testing channels and can simultaneously complete parallel lifetime tests for 16 groups of devices, significantly improving experimental efficiency. Its testing principle is to convert the device's light emission signal into an electrical signal through a silicon photodiode, and record the luminance attenuation curve in real time. It is a commonly used equipment for characterizing the stability of perovskite LEDs (*Nat. Photon.* 2022, 16, 637). Meanwhile, we performed multiple comparative analyses on the consistency of the two test devices, and no significant discrepancy was observed in the stability results (Fig. R4). The luminance decay curves derived from the two devices exhibited a high degree of overlap, confirming the reliability of our device stability.

The specific testing instruments and testing details have been supplemented and revised in the main text.

Change made in the main text (Page 14)

The performance of the encapsulated QLEDs were conducted using the testing system provided by Suzhou Fushida Scientific Instrument Co., Ltd. (**Model 2602B source meter and CS-2000 spectrophotometer**).

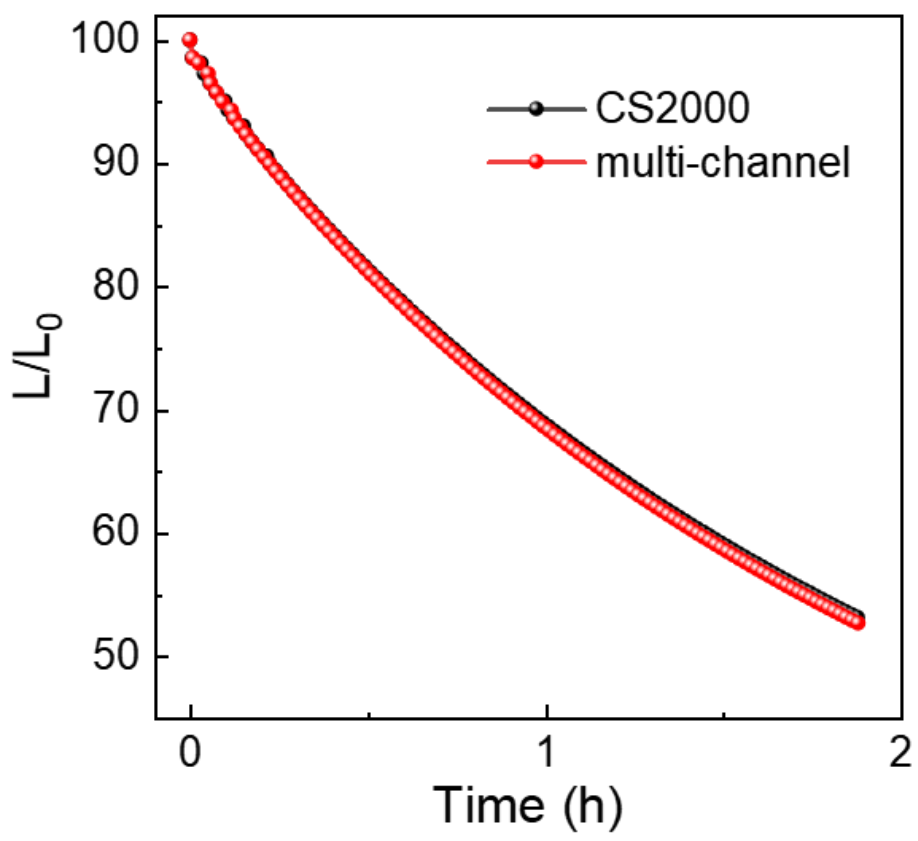

Fig. R4 Comparison of device lifetime under 10,000 cd/m^2 initial luminance in the CS2000 optoelectronic performance testing system and multi-channel LED lifetime-specific testing equipment.

Response to reviewer 2

General Comment: In this manuscript, the authors introduced a BN interlayer into the perovskite QLED configuration, which resulted in a high EQE exceeding 30%. However, it should be noted that the authors previously reported an EQE as high as 30.2% using the same type of quantum dots (Angew. Chem. Int. Ed. 2024, 63, e202410689). Especially, looking carefully into the response letter and revised manuscript, I found most of the conclusions to be farfetched. The EQE of over 30% claimed by the authors is also not convinced, and the reviewer is wondering if such an EQE is even possible at all using their device structures. As we known, in the type LEDs with planar device structure, the light extraction efficiency (LEE) without light outcoupling structure is generally not more than 25%, while their devices have a surprisingly-high out-coupling efficiency of ~40% in the planar device structure without using any light outcoupling structures (Fig. R12). The performance including device operational lifetime (the surface ligands of colloidal perovskite quantum dots with ionic nature are not very stable) reported by these authors should be verified by an independent laboratory. I have strong concerns about the data analyses and the device performance, which should be addressed before the paper can be considered for publication anywhere.

Response: Thank you very much for your valuable comments on this research. These comments are of great significance for improving our work. Regarding the issues you pointed out, we have provided explanations and responses as follows:

(1) Firstly, we would like to thank you for pointing out the correlation between this study and the EQE data we previously reported in "Angew. Chem. Int. Ed." (2024, 63, e202410689). In fact, it is not the case that these QDs can achieve high efficiency in any device structure. Generally speaking, the EQE of LEDs is closely related the PL properties (**PLQY, e.g., φ**), charge balance factor (γ) and out-coupling factor (χ), **which can be semi-quantitatively analyzed using the equation** $\boldsymbol{\eta_{EQE} = \chi \cdot \varphi \cdot \gamma}$ (*Light Sci. Appl.* 2021, 10, 61, *Adv. Sci.* 2023, 10, 2303192). Since the first perovskite QLED is reported, our group has been deeply engaged in the study of perovskite QDs and QLEDs for over a decade. The as-prepared QDs are all aimed at achieving high-performance LEDs. Currently, the PLQY of the QDs used in the devices has exceeded 90%, and even approached 100%. Thus, the φ would not have a significant impact on the EQE in our devices, and **the realization of high-efficiency EQE is highly dependent on the regulation of the device structure (e.g.,** carrier transport balance, γ**)**.

Indeed, the QDs employed in this study are basically consistent with the QDs reported in *Angew*. At this point, the φ would not have a significant impact on the EQE in our devices, and **the realization of high-efficiency EQE is highly dependent on the regulation of the device structure (e.g., carrier transport balance, γ). The core innovation of the *Angew* is**

the introduction of 3P organic molecules as ETL, which can not only passivate the interface defects between QDs and the ETL, but also have high electron mobility, which can form an appropriate mobility correlation with the hole transport material of PTAA, enable the carrier transport balance, and ultimately achieving 30% EQE.

In our BN MS, although the same QDs were used, when 3P was employed as the ETL, the optimal peak EQE was approximately 25% (Fig. R5). This was due to the introduction of ultra-thin BN nanosheets in this study, which somewhat weakened the hole injection ability of the QLED; thus, due to the excessive electron injection and transport capacity, it led to carrier imbalance and degradation of device performance. Therefore, **we selected TPBi-POT2T with a relatively lower mobility as the ETL, optimized the carrier transport balance** (Fig. R6), and finally made the device EQE reach 30%. **In summary, although the QDs are almost the same, the realization of high-efficiency devices heavily relies on the structural design, which can ensure the balance of carrier transport (large** γ**).**

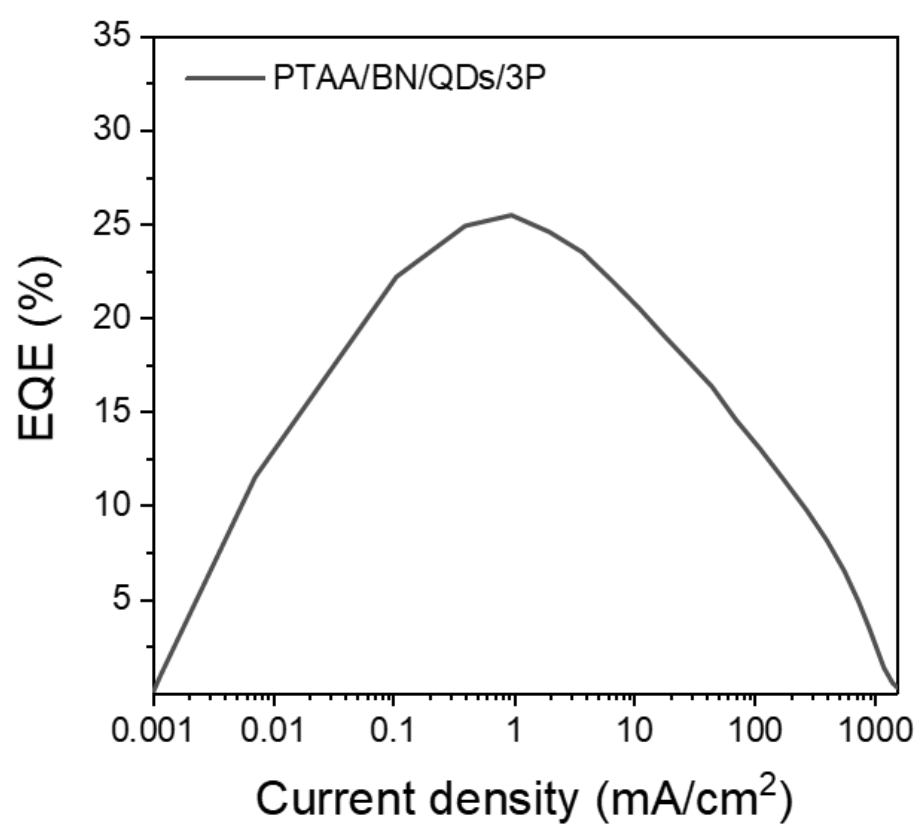

Fig. R5 The EQE-current density curve of QLED with 3P as the electronic transport layer.

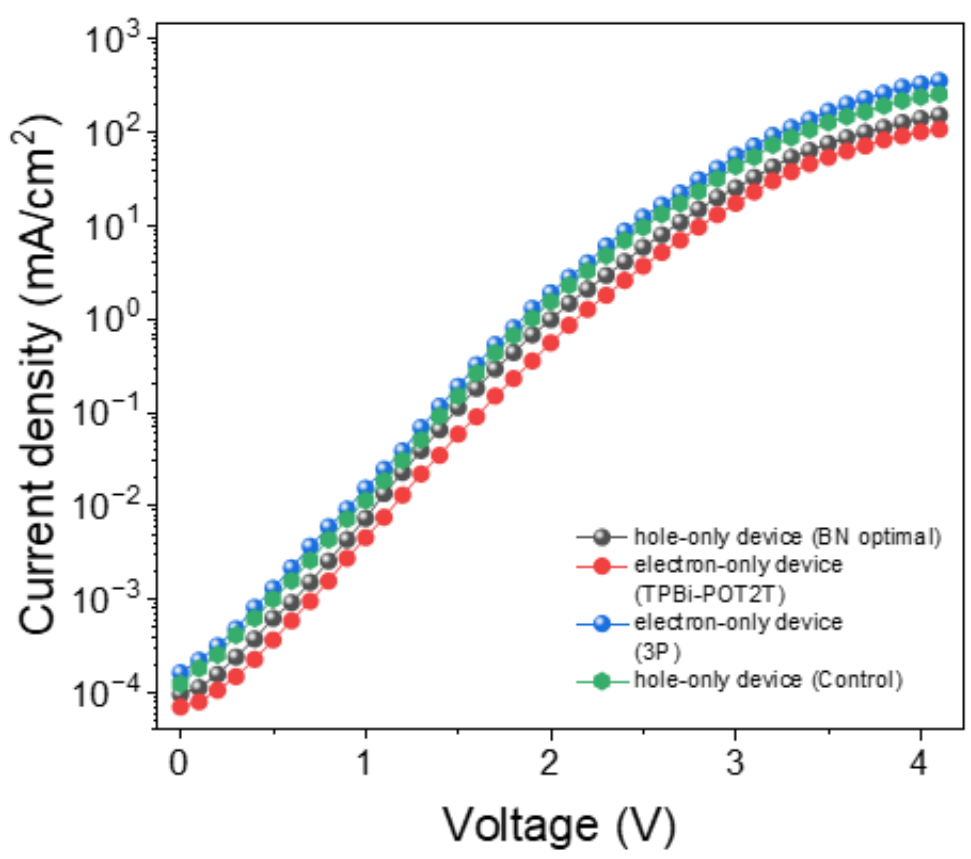

Fig. R6 The electron-only and hole-only devices.

<u>(2) Explanation on the LEE of planar structure devices</u>

The viewpoint you raised, “the light extraction efficiency (LEE) without light outcoupling structure is generally not more than 25%....” is completely consistent with the traditional understanding. To avoid the problem of overestimation of efficiency that might occur when conducting one-dimensional simulations only in the X direction, we entrusted **Tianjin SIMUCAL Technology Co., Ltd.** to optimize the calculation logic, supplemented the **three-dimensional optical simulation** and provide corresponding **simulation reports**, **(Fig. R7)**.

Device Simulation Report

一. Model configuration

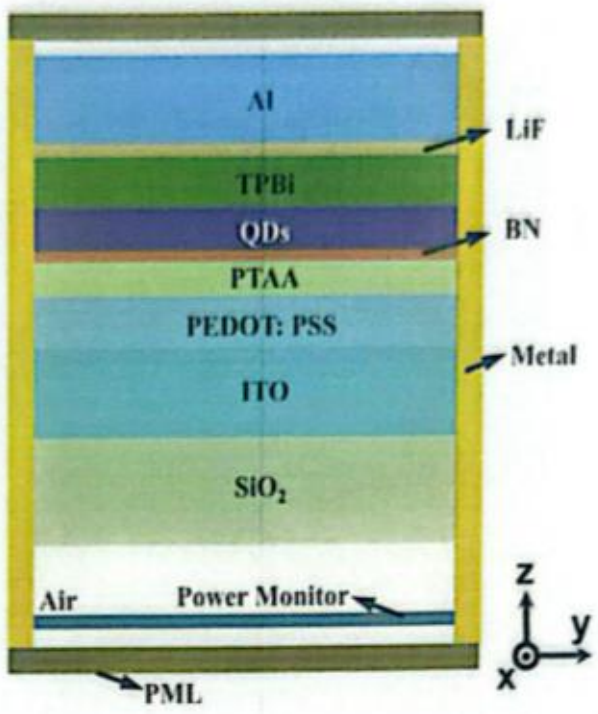

Fig. 1 Schematic diagram of the simulation model

The model schematic is shown in the above figure. The lateral dimensions of the computational model are set to 2×2 μm². The LED consists of the following layers: 1200 nm SiO_2, 100 nm ITO, 40 nm PEDOT:PSS, 20 nm PTAA, 1 nm BN, 30 nm QDs, 40 nm TPBi-POT2T, 1 nm LiF, and 100 nm Al. The absorption coefficient of the QDs layer is set to 10000 cm^{-1} at an emission wavelength of 520 nm. In terms of material refractive indices, the values for the ITO, PEDOT:PSS, PTAA, BN, QDs, TPBi-POT2T, and LiF layers are 1.85, 1.5098, 1.607, 2.1308, 2.02, 1.727, and 1.3921, respectively. The refractive indices and absorption coefficients of SiO_2 and Al are based on the built-in material parameters of SiO_2 (Glass)–(Palik) and Al (Palik) from the FDTD material library. The absorption coefficients of all other layers are set to 0.

When the BN layer is 0 nm, the average light extraction efficiency is 24.1%. With photon recycling, the average light extraction efficiency becomes 25.7%. When the BN layer is 1 nm, the average light extraction efficiency is 30.8%. With photon recycling, the average light extraction efficiency reaches 35.7%.

Company:天津赛米卡尔科技有限公司（Tianjin SIMUCAL Technology Co., Ltd)

Time:18/3/2026

Fig. R7 The simulation results from Tianjin SIMUCAL Technology Co., Ltd..

In the simulations, the polarization direction of the dipole source includes the X, Y, and Z dimensions. Randomly oriented dipole excitations are used to describe the lateral electric field distribution near the surface, the average LEE reaches 35.7% which is consistent with our experimental results. The high LEE is due to two aspects. The first point is that **the enhanced optical confinement in thinner perovskite QD films promotes horizontal dipole emission, thereby increasing the proportion of horizontally oriented transitions. A higher ratio of such transitions consequently leads to a greater EQE in PeLEDs** (*Nat. Nanotechnol*. 2023, 18, 981). The second one is that **the Stokes shift of perovskite materials is relatively small, and a photon recycling effect will occur within the device** - this effect enables the waveguide mode and the bound photons in the substrate to regain the extraction opportunity through the reabsorption-re-emission process, significantly improving the light extraction rate of the device (*Nat Commun*, 2020, 11, 611; *Nat. Nanotechnol*. 2023, 18, 981).

(3) Regarding the EQE of our devices. In the early days of the LED field, such as OLED and QLED devices, the theoretical upper limit of EQE was generally believed to be around 25%. Similarly, the theoretical maximum efficiency of perovskite-based LEDs initially was also estimated to be 25%. However, since the first room-temperature perovskite film-based LEDs and QLEDs are reported (*Nat. Nanotechnol.* 2014, 9, 687; *Adv. Mater.* 2015, 27, 7162), the EQE of these devices has achieved significant breakthroughs (EQE exceeding 30%). Although the internal mechanism of the ultra-high EQE of current perovskite devices is not yet fully clear, an increasing number of theoretical simulations have shown that the theoretical upper limit of the EQE can be increased to about 40% (*Adv. Mater.* 2023, 35, 2302283*; Opt. Lett.* 2020, 45, 4786). **The exceptional EQE of planar perovskite-based LED may be** primarily attributed to the photon recycling (PR) based on a small Stokes shift (e.g., large absorption/emission overlap), which supports the cycling of re-absorption and re-emission. As Greenham et al presented, "While an outcoupling efficiency of 100% is theoretically possible with PR", which indicated that the EQE of planar perovskite-based LED still has enormous room for improvement (*Nat. Commun.* 2020, 11, 611).

Guided by the previous reports, we conducted FDTD simulations for our device. The simulation results indicated that the introduction of BN had a relatively certain impact on the LEE of the device (30.8% for QLED with BN, 24.1% for control QLED). When we further considered the PR effect, the LEE of the QLED optimized with BN could reach 35.7%. Thus, our QLED achieving 30% EQE is rational according to the theoretical calculation results.

In real situations, our device design is expected to achieve high-performance QLEDs. Firstly, compared to pristine QDs, the BN optimal QDs present enhanced PL properties (**a**

high PLQY, e.g., φ) owing to reducing the defect density of QDs (Fig. 2g, h). Secondly, the BN layer slightly reduces the hole injection capacity (Fig. 5d). Thus, we choose TPBi-POT2T with a relatively lower mobility as the electron transport material to achieve the balance carrier transport, thereby improve the charge balance factor carrier utilization rate (*γ*). Furthermore, in perovskite QLEDs, an appropriate PR effect can enhance the light extraction efficiency (**a high χ**). According to the equation $\boldsymbol{\eta_{EQE} = \chi \cdot \varphi \cdot \gamma}$ (*Light Sci. Appl.* 2021, 10, 61, *Adv. Sci.* 2023, 10, 2303192), our QLEDs should have a high EQE. In contrast, the control QLED would have a lower device efficiency due to QDs with a relatively lower **φ**, and a small ***γ*** (this is because that PTAA had a higher hole mobility, resulting in a significant imbalance in carrier transport, which is crucial for device performance).

In terms of the testing principle of the device, our testing equipment adopts a combination scheme of CS2000 spectrophotometer and 2602B source meter. The CS2000 spectrophotometer is used to detect light, while the 2602B source meter provides stable and precise current-voltage drive for the device. Such equipment is also used for testing perovskite-based LEDs (*Nat. Photonics* 2021, 15, 238; *Nature* 2022, 611, 688). Therefore, it is reasonable to measure an EQE of over 30% using our equipment.

Meanwhile, regarding the reliability of our EQE, **we also conducted verification and comparison experiments in Shen Huaibin's group**. **The maximum EQE was 30.8%, which further confirmed the reliability of our data (Fig. R8).** Choosing to conduct cross-validation in Shen's group was mainly due to the fact that Shen's research group has been deeply engaged in the QD field for nearly 20 years, achieving significant results and having a considerable influence (such as *Nature* 2024, 635, 854; *Nat. Photon.* 2024, 18, 186; *Nat Commun* 2025, 16, 10143, etc.). The related verification results are highly persuasive. At the same time, our perovskite emitter is in a nanocrystal form, which is highly in line with the research system of Shen's group, ensuring the rationality of the verification. Indeed, the efficiency reliability of current perovskite LEDs still needs to be further strengthened and confirmed. Unlike solar cells (IEC 61215 standard) and photodetectors (standardized protocols in *Nat. Photon.* 2025, 19, 1178), the perovskite-based LED field still lacks unified testing guidelines, leading to inconsistent reporting of core metrics. We believe that the testing of perovskite LEDs will become more standardized in the near future.

Furthermore, a recent ref. (*Nature* 2026, 651, 83) about perovskite nanocrystal LEDs has reported an EQE of 29.6%, which also indicates that perovskite nanocrystal as emitters could

obtain high performance LEDs.

In summary, we believe that our device has achieved a champion peak EQE of 32%, which is reasonable and credible. Considering that the random maximum point is difficult to rigorously characterize the experimental results, to ensure the reliability of the data, **only in the curve description of Fig. 5f, "the champion peak EQE of 32%" was adopted, and for all other descriptions in the MS, we uniformly employed "the average peak EQE of 30.05%".**

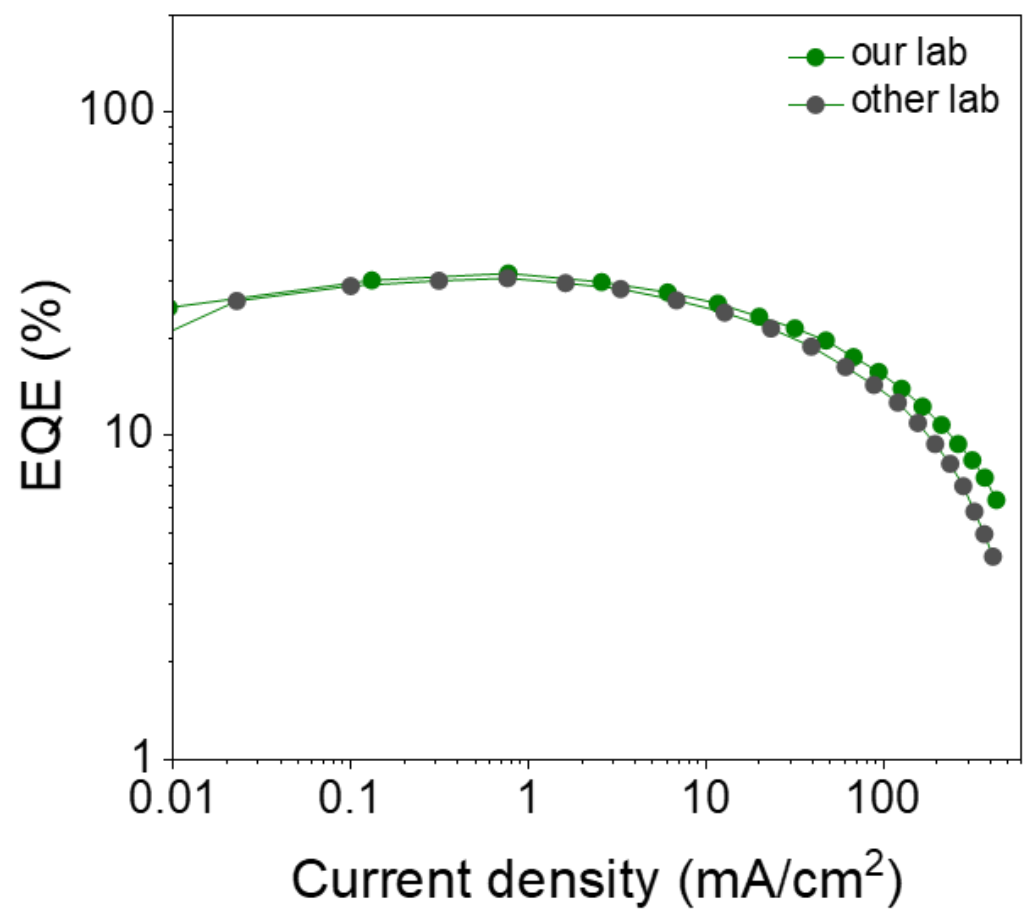

Fig. R8 EQE data with measurements from other labs.

<u>(4) Regarding the stability of our devices.</u> **The stability of perovskite LEDs is typically constrained by various factors, including halide ion migration, imbalance in carrier transport, and the accumulation of Joule heat during the device operation**. Therefore, developing strategies that can simultaneously optimize these key issues is significantly crucial for enhancing the stability of the devices. To address this challenge, **our study innovatively introduces an ultrathin BN functional layer beneath the QD layer to enhance stability**.

The BN functional layer can enhance device stability through the following synergistic mechanisms. **First, BN can effectively inhibit the migration and diffusion of Br^- at the physical level** (Fig. 3f-h as evidence) compared to control sample; Second, **BN can also passivate QDs** (Fig. 2f-j as evidence) and reduce the defect states, which mitigates ion migration and consequently enhances device stability. Moreover, the BN optimal QLED has the optimized charge transport balance (Supplementary Fig. 35 as evidence), which reduced interface exciton quenching, and played a positive role in improving the stability of the device. In addition, **BN itself has excellent heat dissipation properties, which can effectively alleviate the Joule heat accumulation effect during device operation** (Fig. 4 as evidence),

and improving stability. In our device design, benefiting from the aforementioned multiple advantages brought by BN, the BN-optimized QLED exhibits excellent stability compared to the control device.

In addition, we also conducted stability data cross-laboratory verification with the Shen's group under an initial luminance of 2000 cd/m^2. The deviation of the tested device lifetimes from both groups was less than 5% (Fig. R9), further verifying the accuracy and credibility of the device stability data.

In summary, in terms of device stability, **BN has multiple functions in device design,** including passivating interface defects, inhibiting ion migration, optimizing carrier injection balance, and enhancing heat dissipation, significantly improving the operational stability of the device. The experimental results show that the BN optimized QLED achieved a lifetime of 25263 hours, which is 100 times higher than the control device. Thus, the excellent stability exhibited by BN optimized QLED has sufficient rationality and credibility.

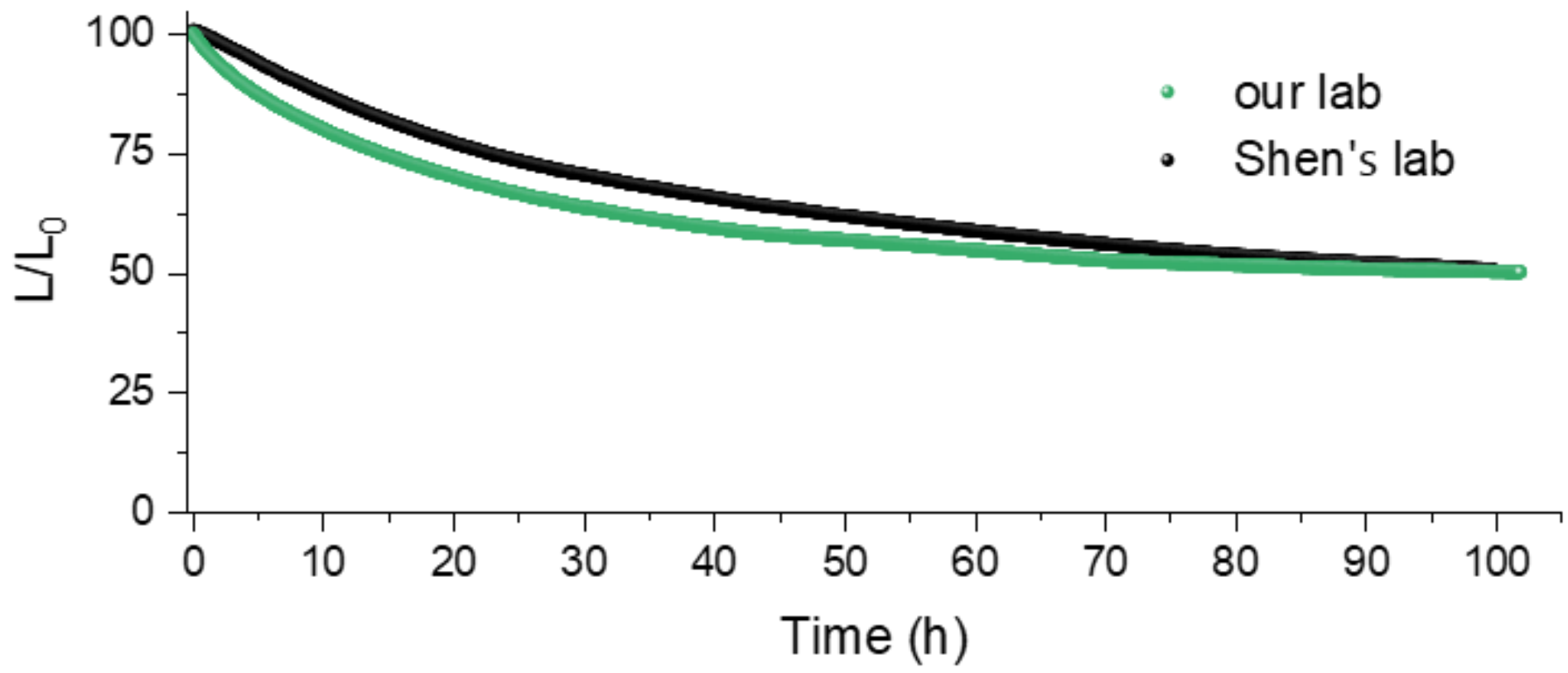

Fig. R9 The operation lifetime of BN-optimized QLEDs under an initial luminance of 2000 cd/m^2 from our and Shen's lab.

(5) Regarding the issue of "the surface ligands of colloidal perovskite quantum dots with ionic properties are not very stable", we would like to clarify that **in earlier studies, the ionic ligands on the surface of perovskite QDs** (such as salted amine, iodinated ammonium, etc.) **were in a dynamic equilibrium state**, which led to QD aggregation and flocculation, significantly affecting the optical and electrical properties. **Subsequently, some strongly bonded ligands** were used to improve the stability of QDs, including phosphoric acid (*Nature* 2022, 611, 688), sulfonic acid (*ACS Appl. Mater. Interfaces* 2025, 17, 6639), thiocyanate (*Light Sci Appl* 2025, 14, 35), etc.. These ligands bind tightly to the surface active sites of QDs through strong coordination or ionic bonding, greatly enhancing the stability of QDs. At present, the ligand used for perovskite QDs has become well-established, which can balance

high PLQY, excellent colloidal stability, and PL and EL stability.

Once again, we thank the reviewers for their constructive suggestions. We have supplemented relevant experimental details and literature support according to the above explanations, further improving the manuscript, and hope to receive your approval.

Question 1. The manuscript claims that the BN layer can passivate surface defects of the quantum dots. However, since BN is deposited beneath the quantum dot layer, this explanation is not entirely convincing. A more reasonable interpretation is that BN mainly passivates interface defects, improves interfacial energy alignment, and optimizes carrier transport. The authors are encouraged to clarify this point in the discussion to avoid conceptual confusion.

Response: Thank you for your valuable suggestions. We fully agree with your core viewpoint that the BN layer is deposited beneath the QDs layer. However, the role of nanomaterials is very significant.

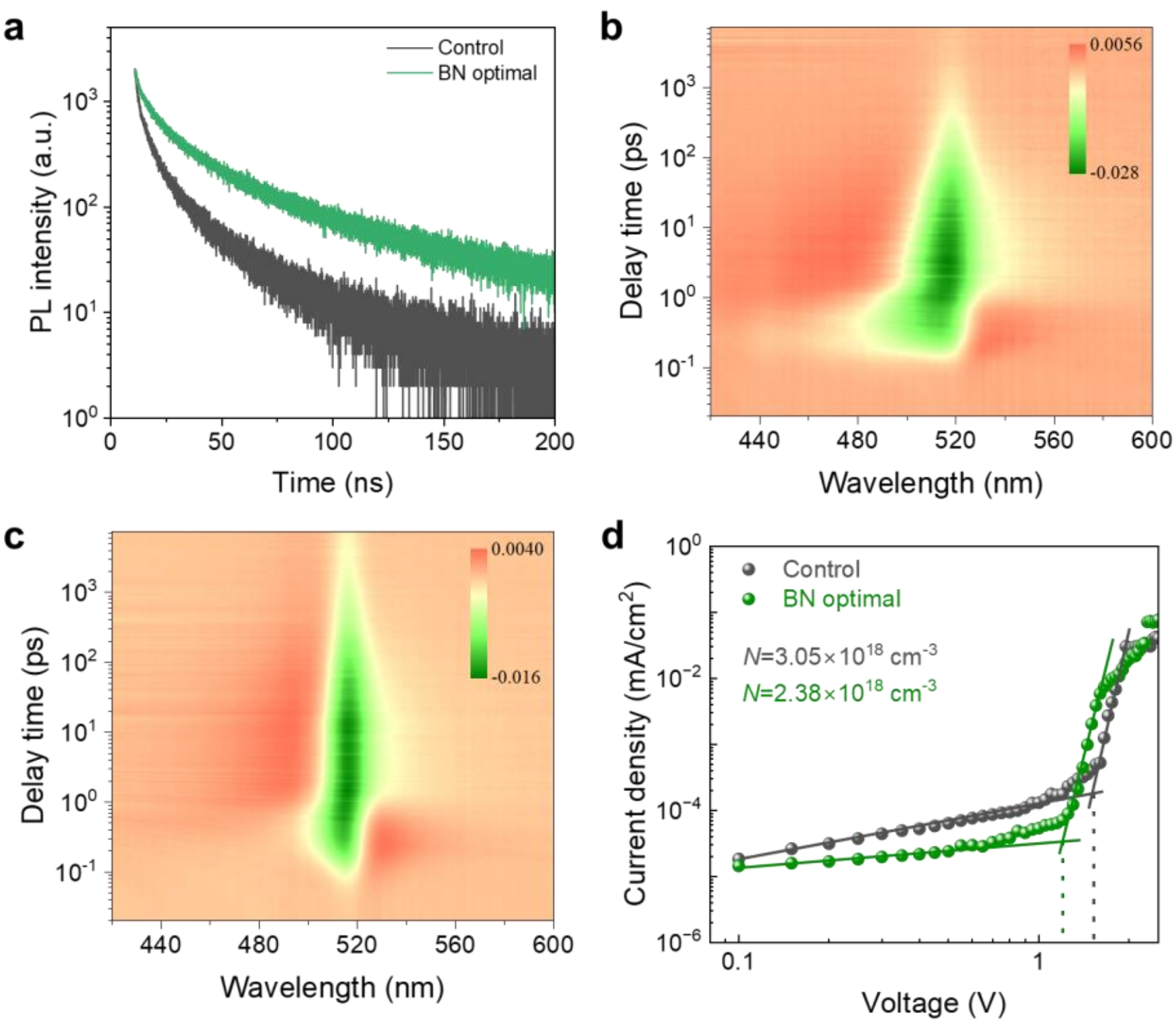

Fig. R10 (a) TRPL delay curve of control and BN-optimized QD films. Pseudo-color TA spectra and pump-detection delay time for control (b) and BN-optimized (c) QD films. (d) current density–voltage characteristics.

First, as shown in PL results (Fig. 2g), it can be evident seen that the introduction of BN significantly enhances the luminescence performance of the QD films. The defect-related characterization and the defect density calculation (Fig. R10) further confirm the effective

suppression of defects; combined with DFT theoretical calculations and XPS etching test results (Fig. R11), it is clearly verified that the BN layer has the effect of passivating interface defects. Meanwhile, the recent reports in the literature also indicates that BN could enhance the PL and reduce the nonradiative recombination (*Small* 2023, 19, 2207092; *Nano Energy* 2026, 148, 111696).

Second, BN deposited beneath the QDs acts as a buried interfacial layer, which could passivate interface defects. Although there are relatively few reports on BN layer passivation of interface defects in optoelectronic devices, studies on similar inorganic materials such as Si_3N_4, BP nanoflakes, and $Ti_3C_2T_x$ in perovskite solar cells have confirmed that these materials have excellent interface defect passivation capabilities (*Nat. Photon.* 2026, 20, 280; *Surfaces and Interfaces* 2023, 43, 103531; *Adv. Funct. Mater.* 2023, 33, 2300700), which can provide additional support for this work.

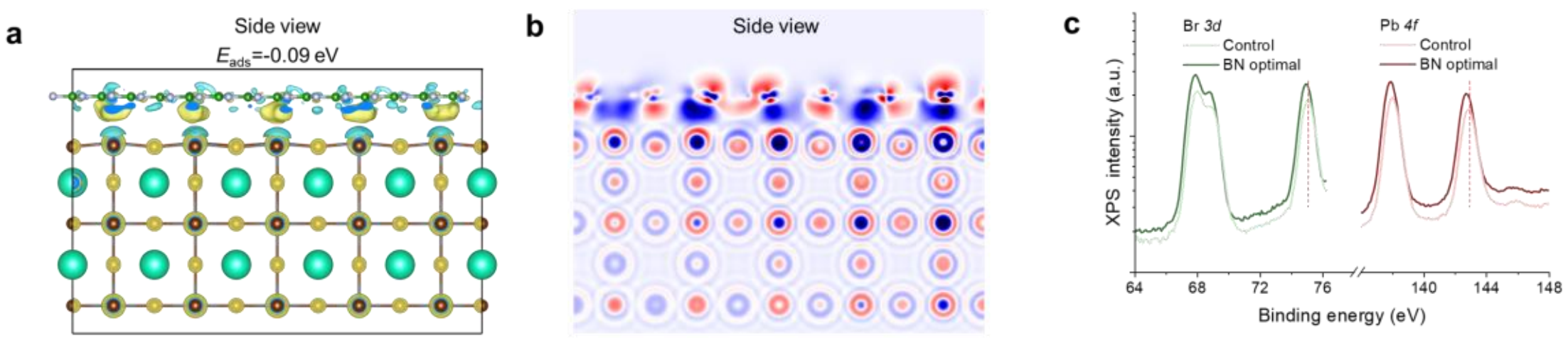

Fig. R11 (a-b) 3D and 2D slice of charge density differences of $PbBr_2$-3 terminated $CsPbBr_3$. (c) High resolution XPS spectra of Pb 4*f* and Br 3*d* for control and BN-optimized QD films.

Regarding the regulatory effect of BN layer on the **arrangement of interface energy levels**, we have performed UPS measurements, and the results are shown in Fig. R12. After introducing BN, the HOMO energy level of QD films decreased by only 0.04 eV, indicating that BN exerts no significant modulation on the energy level characteristics of QD films. This conclusion is consistent with the reports (*Adv. Mater.* 2015, 27, 7800). These results imply that the introduced BN does not significantly change the hole transport and electron-hole pair recombination. In addition, the KPFM test results showed that the introduction of BN did not induce an obvious shift in the surface potential of the QD films, but slightly improved the uniformity of the surface potential (Fig. R13). The improved uniformity of surface potential suggests that the introduction of BN is beneficial for the uniform distribution and synchronous transport of electrons and holes in the QD layer, and promoting exciton recombination to a certain extent (*Energy Environ. Sci.*, 2025,18, 319; *J. Mater. Chem. A*, 2025,13, 29583).

Regarding the influence of BN layer incorporation on the **carrier transport behavior of the device**, we found that it slightly weakened the hole-injection capability of the QLED (Fig.

R6). Accordingly, in this work, we selected TPBi-POT2T, which has better compatibility with hole injection, as the electron transport layer, effectively achieving the balance of carrier injection and transport (Supplementary Fig. 35).

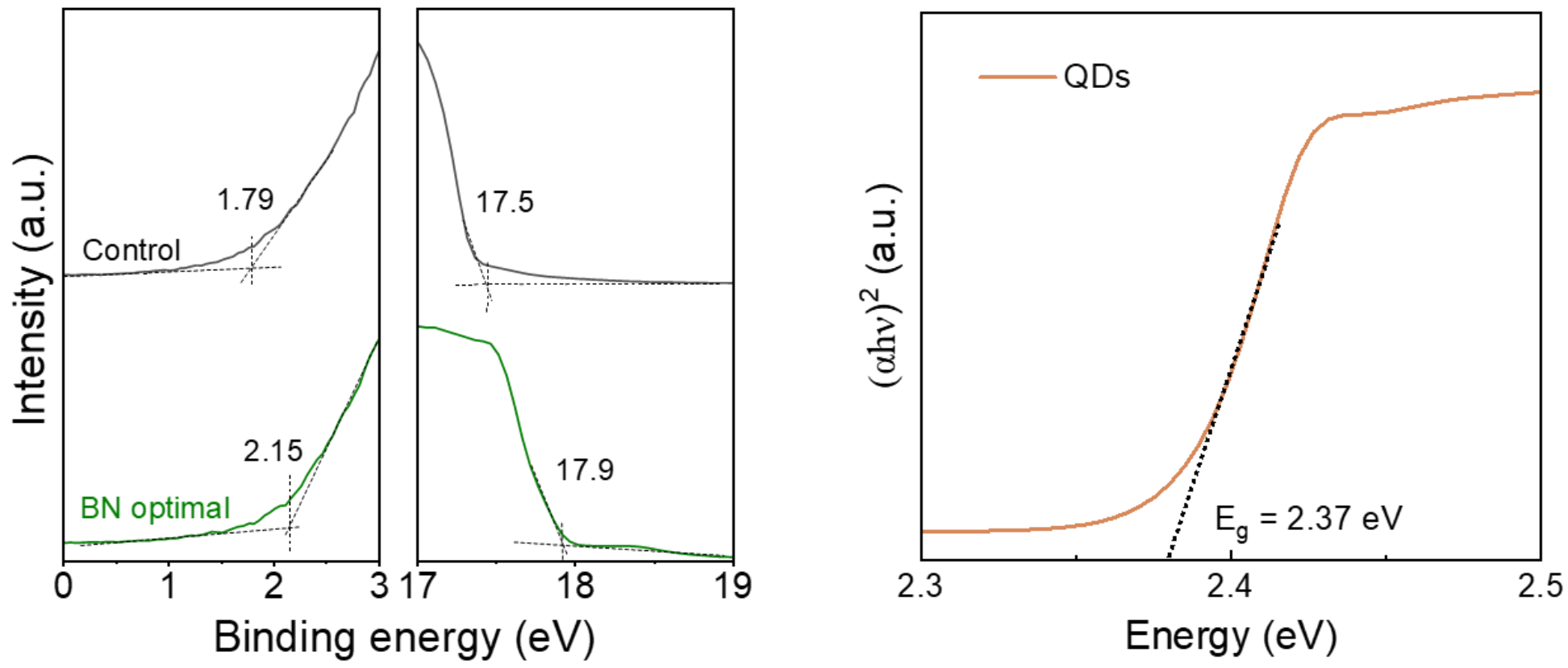

Fig. R12 (a) High-binding-energy secondary electron cutoff and valence-band edge regions of QDs and QDs/BN film measured by UPS; (b) Bandgap values of QD films from absorption spectra.

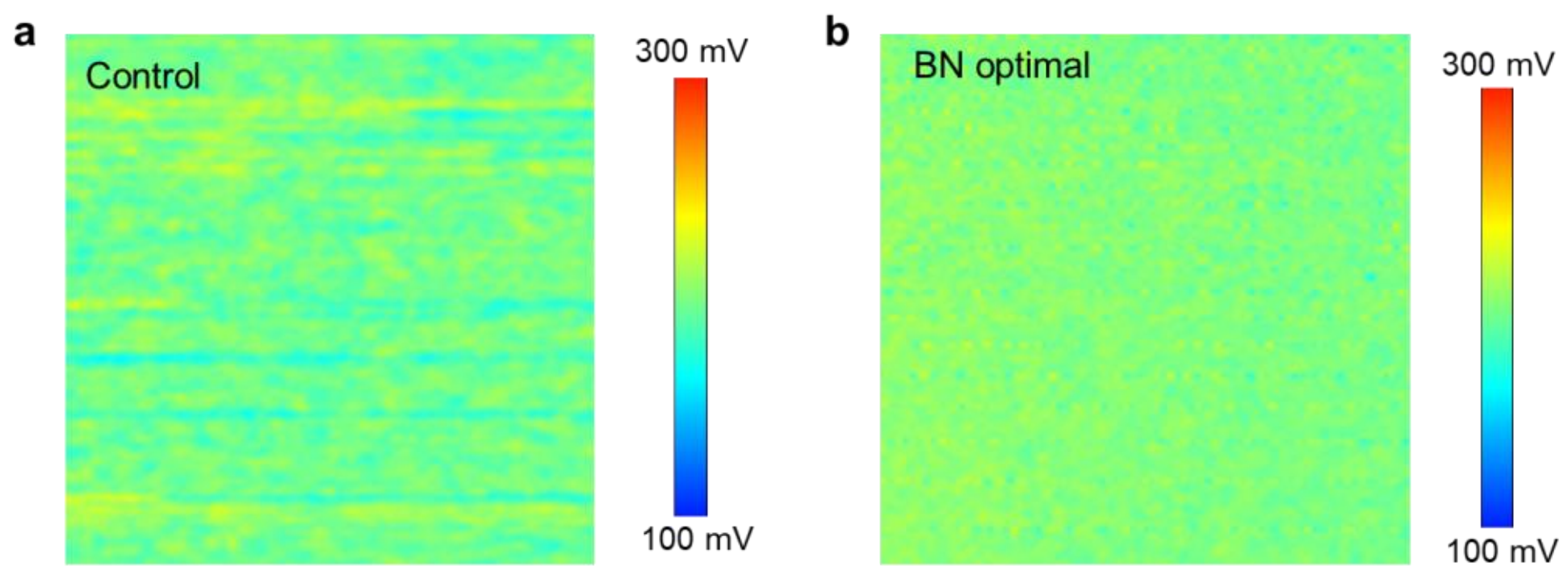

Fig. R13 KPFM images for the control (a) and (b) BN optimal QD films.

Question 2. The authors state that the BN interlayer can suppress ion migration at the quantum dot surface. However, since the interlayer only exists at the interface between the QD layer and adjacent layers, its ability to directly inhibit ion migration through the thickness of the QD layer remains unclear. Furthermore, in the schematic illustration of Figure 3g, the authors also claim that ion migration on the side of the electron transport layer is suppressed, yet no detailed mechanism or experimental evidence is provided in the manuscript. The authors are encouraged to clarify this point and provide further justification or supporting data.

Response: Thank you for your comments. Regarding your point that “the BN interlayer can suppress ion migration at the quantum dot surface”, we have carefully checked our MS and found that there is no relevant description in the current MS. This misunderstanding may have arisen from our previous oversight, for which we apologize. As you said, in our MS, BN indeed exists at the interface between the QD layer and adjacent layers, acting as a buried

interface layer.

Regarding the regulatory effect of the BN interlayer on ions migration in the QD layer, we believe BN suppresses the ion migration through the synergistic effect of interface defect passivation and the enhancement of the ion migration barrier. First, the BN can improve the luminescence performance of QD films (Fig. 2g). Combined with the calculation of defect density (Supplementary Fig. 15), it is further verified that the introduction of BN significantly reduces the interface defect density. The effective elimination of interface defects directly cut off the key pathways for the migration of ions from the QDs interior to the interface and across the layers, reducing the driving force for the directional migration of ions within the QDs at the source. Meanwhile, relevant BN-based studies have demonstrated that ions tend to migrate preferentially along defect sites such as interfaces and grain boundaries. These high-energy defects provide low-barrier migration channels for ions and are also the core nucleation sites for the migration and aggregation of ions within the QDs (*Nano Lett.* 2024, 24, 16443; *Adv. Mater.* 37, 2505214; *Nat. Photon.* 2023, 17, 401). In addition, our calculations reveal that the migration potential barrier of Br^- and the activation energy for ion migration both increase after the introduction of BN (Fig. R14), indicating that the interface modification of BN can regulate interfacial charges, indirectly enhancing the migration resistance of charged ions within the QDs, achieving the inhibition of ion migration. In summary, the inhibition of ion migration by the BN interlayer is a dual effect of "interface passivation blocking migration channels + elevated potential barriers suppressing overall ion motion". Furthermore, previous studies have also confirmed that BN plays a crucial role in preventing ion migration (*Chem. Eng. J.* 2024, 480, 147945; *Small* 2023, 19, 2207092).

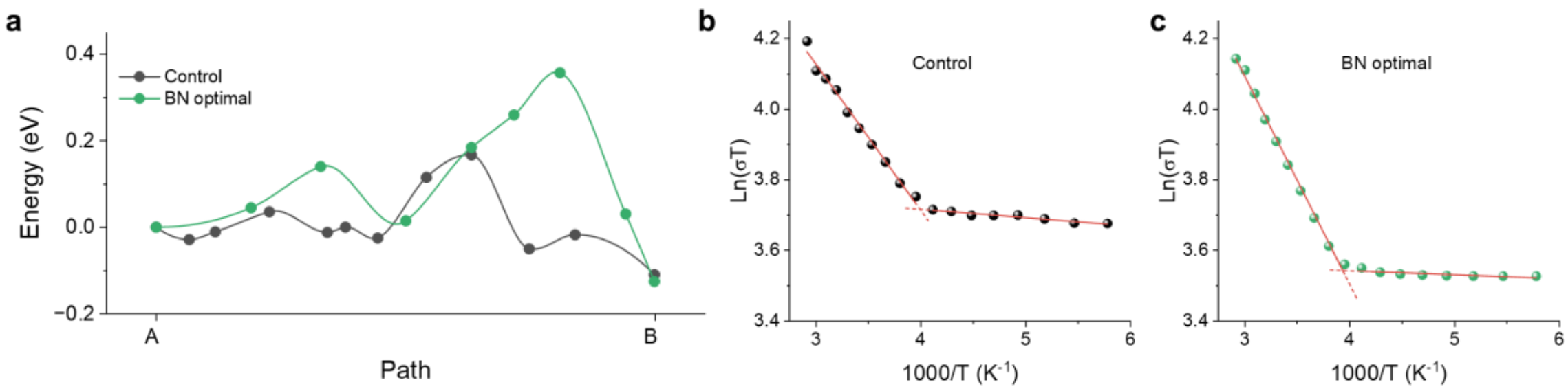

Fig. R14 (a) The potential barriers of CsBr-terminated surface $CsPbBr_3$ without/with BN adsorption. Temperature-dependence conductivity σ(T) for control (b) and BN-optimized devices (c).

Regarding the inhibition of ion migration on the ETL side illustrated in the schematic of Fig. 3g, it is proposed based on the aforementioned dual effects of BN intercalation, and combined with the ToF-SIMS depth-profiling results. As is currently known, halide ions

generally migrate toward the anode, thereby degrading the performance of the device (*Energy Environ. Sci.*, 2025,18, 8744). Based on the ToF-SIMS depth-profiling results (Fig. 3g), we can observe that in the control QLED device, a considerable amount of Br^- migrates toward the anode, whereas only a small fraction migrates toward the cathode. In general, halide ions exhibit nearly symmetric migration or diffusion toward both electrodes in devices without bias aging. However, after the device is subjected to forward-bias aging (2000 cd/m^2, 40 min), halide ions undergo significant migration toward the anode, leading to an increased Br^- concentration in the HTL and a decreased Br^- concentration in the ETL. For BN optimal QLED, the passivation effect of BN as a buried interfacial layer can reduce the defect state density and suppress the ion migration pathways. Consequently, even after forward-bias aging, the Br^- concentration on the ETL side remains relatively low. On the HTL side, the Br^- content is also reduced owing to the fewer ion migration channels; in addition, the blocking effect of BN further hinders Br^- migration toward the HTL. Therefore, in our schematic, combined with the ToF-SIMS depth-profiling results, the ETL still contains a certain amount of Br^- in the control QLED, while the Br^- concentration in the HTL is relatively higher. In contrast, for the BN-optimized QLED, the ETL contains fewer Br^- ions, and the Br^- content in the HTL is also lower. Thus, we believe that our current schematic diagram is reasonable.

Question 3. The BN layer is deposited beneath the quantum dot film, while the manuscript indicates that the QD layer has a thickness of 2–3 layers and each QD has a size of about 8–10 nm. Under these conditions, it is questionable whether direct chemical coordination between BN and the QDs can occur. Moreover, since the probing depth of XPS is typically less than 10 nm, the reported chemical shifts may not unambiguously demonstrate chemical interactions between BN and the QDs, but could instead arise from other factors. The authors are encouraged to clarify this point.

Response: Thank you for the valuable questions and rigorous suggestions. Regarding your inquiries about the chemical interaction between BN and QDs, as well as the XPS detection depth, we hereby provide a detailed clarification:

Firstly, regarding the limitations of the XPS detection depth, we have fully considered for this issue during the experimental design stage. In our previous tests, BN was spin-coated onto QDs and studied their interactions via XPS. However, this configuration differs from the actual device structure where BN is located beneath the QDs, so we repeated the measurements and specifically employed depth-resolved etched XPS characterization (Fig. R15). It should be emphasized that: the XPS data supporting the conclusion that “BN optimal” all correspond to the test results at an etching depth of 25 nm. Meanwhile, we have revised the XPS characterization section and supplemented detailed experimental procedures. The updated XPS results are consistent with our previous findings, that is, compared with the QD

films, the characteristic peaks of Pb 4f and Br 3d in BN-optimized QD films shifted to lower binding energies, whereas the N 1s peak shifts to higher binding energies (Fig. 2f and Supplementary Fig. 11), indicating that there is a direct chemical coordination between BN and QDs.

Meanwhile, **our DFT calculations further verified** that the adsorption energies of the BN-perovskite composite system are all negative, indicating that the adsorption process is spontaneous and the interfacial binding is stable, ruling out the possibility of weak physical adsorption; Furthermore, **differential charge density analysis** revealed charge density redistribution in the adsorbed system, also confirming the existence of strong interactions beyond van der Waals forces. These results provide a solid theoretical foundation for the formation of bonding interactions (Fig. 2a-c, and Supplementary Fig. 6).

These results of the theoretical calculations and experimental characterization mutually corroborate and are highly consistent, clearly demonstrating the direct chemical coordination between BN and perovskite interface.

In addition, we revise the Fig. 2f description as "**f**, High resolution XPS spectra of Pb 4f and Br 3d for control and 25 nm etching of BN-optimized QD films.".

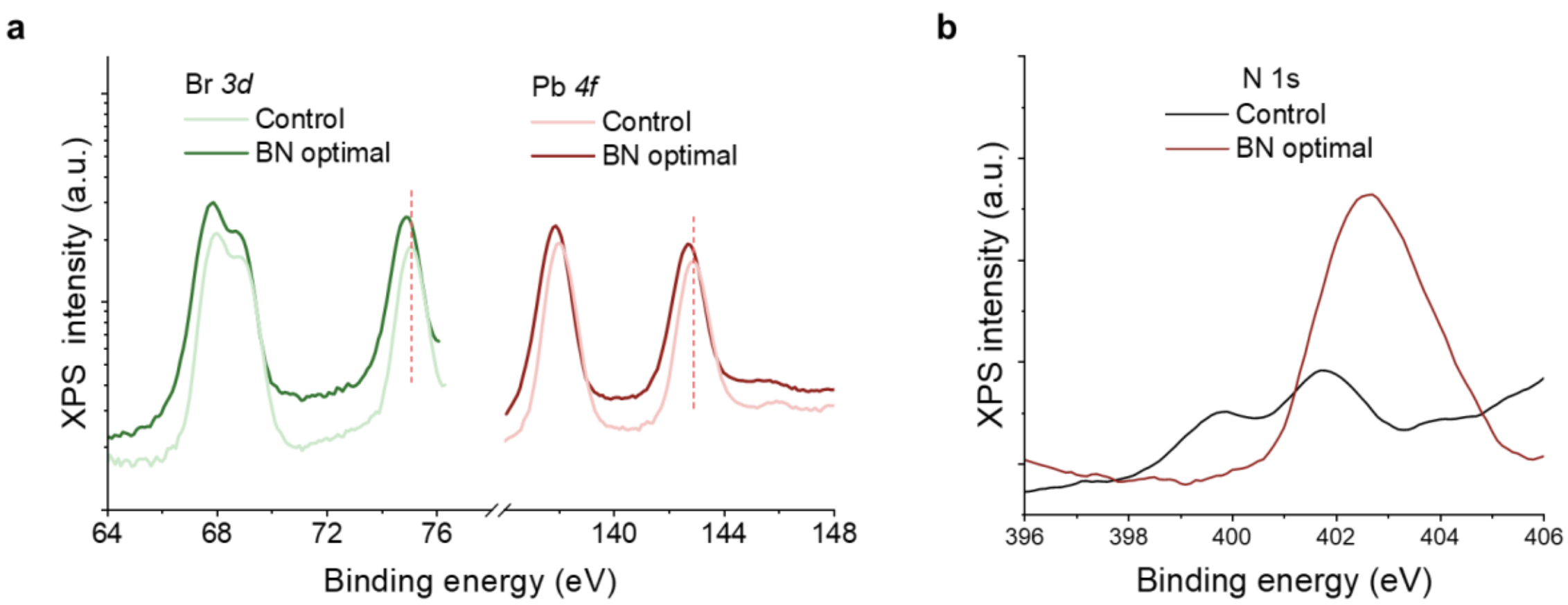

Fig. R15 High resolution XPS spectra of Pb 4f, Br 3d (a) and N 1s (b).

Question 4. In the optical simulation section, it is recommended that the authors provide additional results of the energy output at the monitor ports under different thicknesses, to better evaluate the optical behavior of the devices. From the electric field distribution plots, it can be seen that in Figure c (5 nm), the downward emission is more pronounced, while at 1 nm the electric field shows stronger emission towards both sides. It would therefore be informative to show and discuss the field distribution at the optimal thickness (e.g., 4 nm). In addition, the current simulation appears to have considered ITO but not the glass substrate,

which may cause discrepancies compared with the actual optical outcoupling. The authors are encouraged to address or clarify this point.

Response: Thank you for your valuable suggestions, which help us improve the completeness and accuracy of the optical simulation analysis.

Firstly, regarding the glass substrate that was omitted from the initial simulation, we have incorporated it into the computational model and rerun all relevant simulations to eliminate the differences between the simulation and the actual optical output coupling conditions of the device (Fig. R16).

Secondly, to satisfy the requirements for systematic evaluation of device optical performance at varying BN layer thicknesses, we have supplemented additional simulation data and corresponding in-depth analysis: **we have added electric field distribution characteristic maps of X-polarized light on the XZ and XY planes for BN layer thicknesses of 0, 1, 4, and 5 nm** (Figs. R17-R18), and systematically compared the light field radiation behaviors under each thicknesses. The results demonstrate that the device with a 1 nm BN layer exhibits enhanced electric field radiation intensity in both directions, accompanied by uniform light field propagation and the highest energy coupling efficiency. This configuration achieves the optimal balance of directional emission performance and output efficiency, and clearly verifying that 1 nm represents the optimal BN layer thickness for regulating the optical performance of the device.

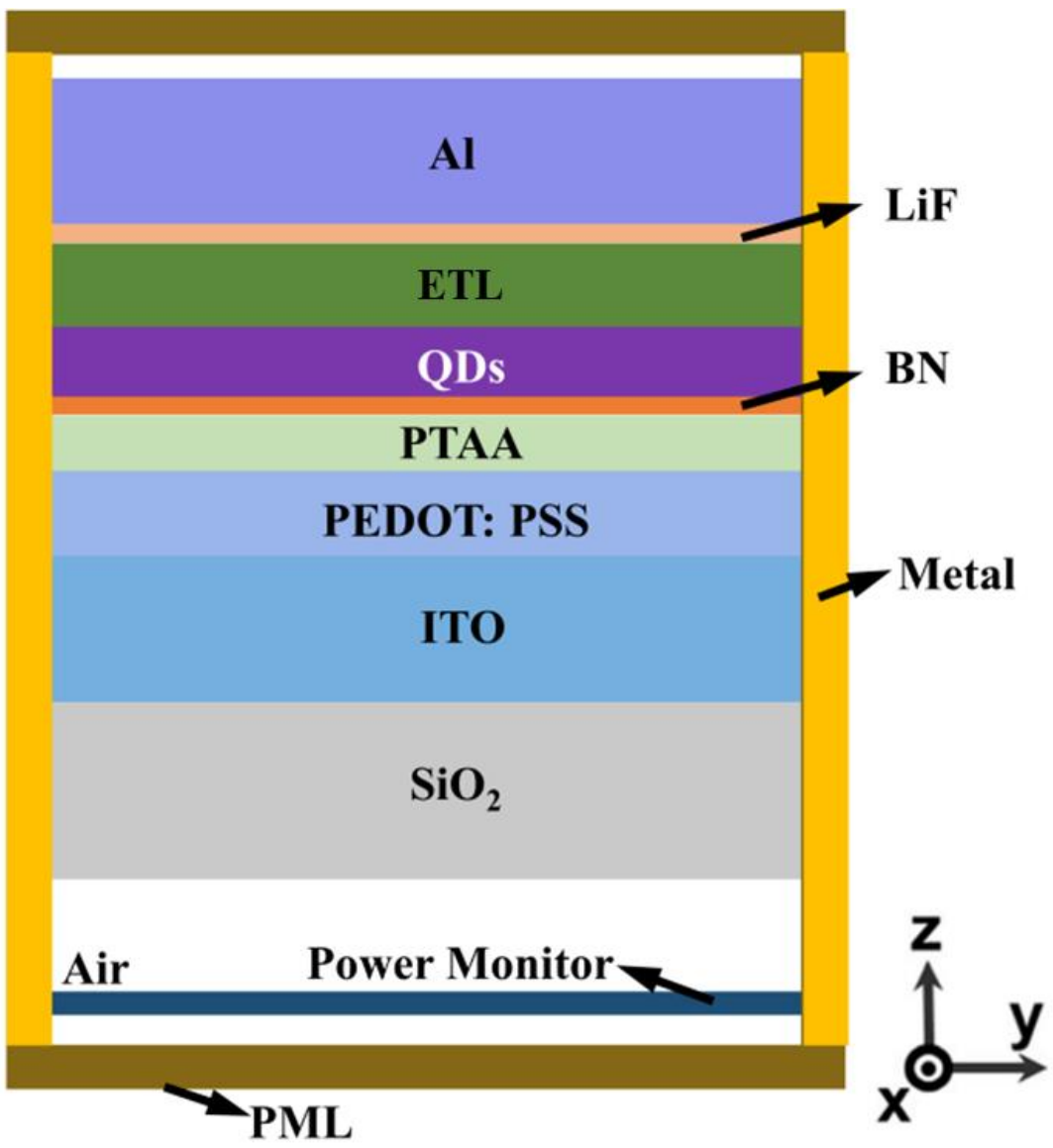

Fig. R16 Schematic diagram of the simulation model.

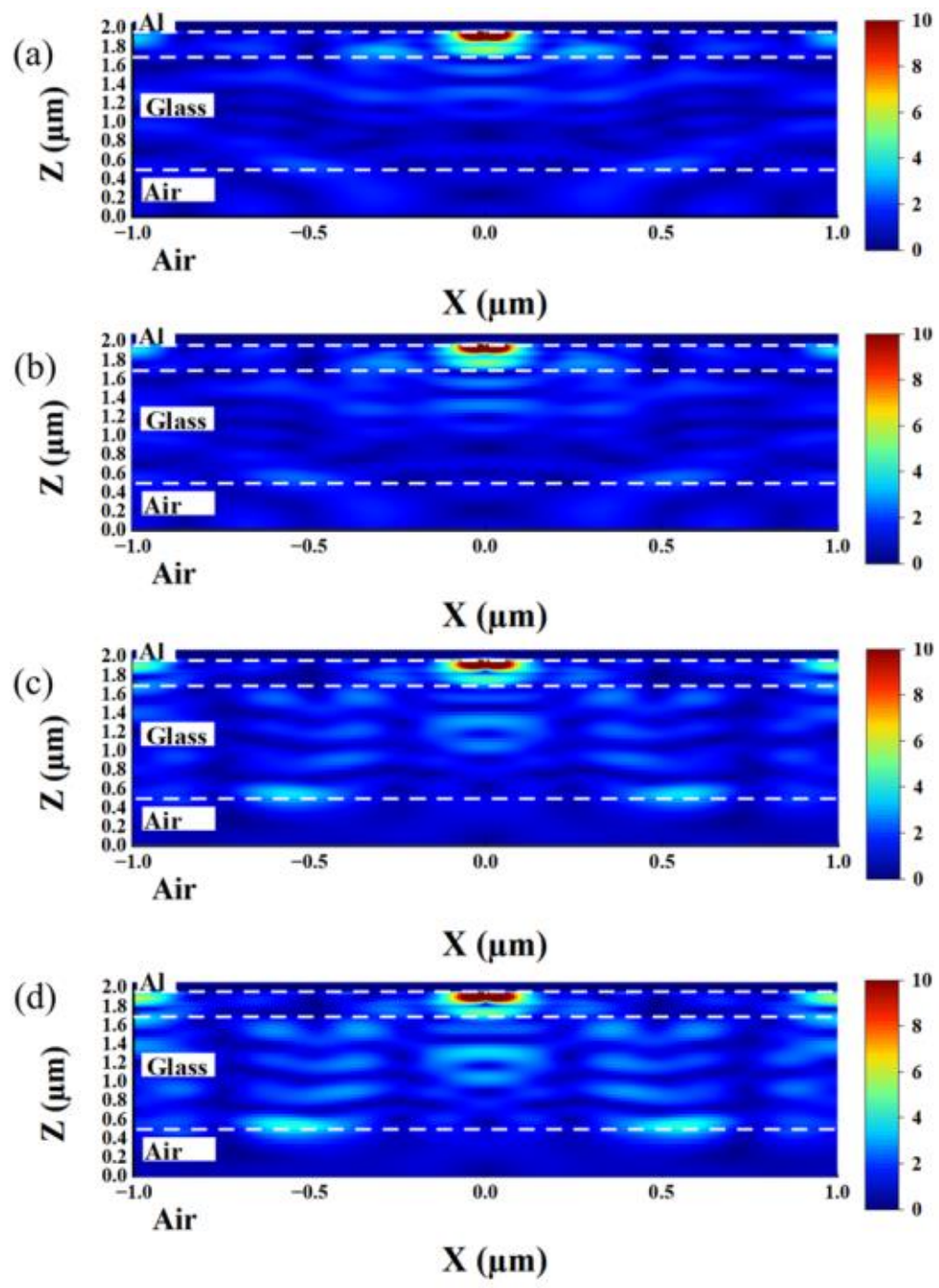

Fig. R17 Electric field distribution and analysis in the XZ cross-section of X-polarized light. (a) 0 nm BN layer. (b) 1 nm BN layer. (c) 4 nm BN layer. (d) 5 nm BN layer.

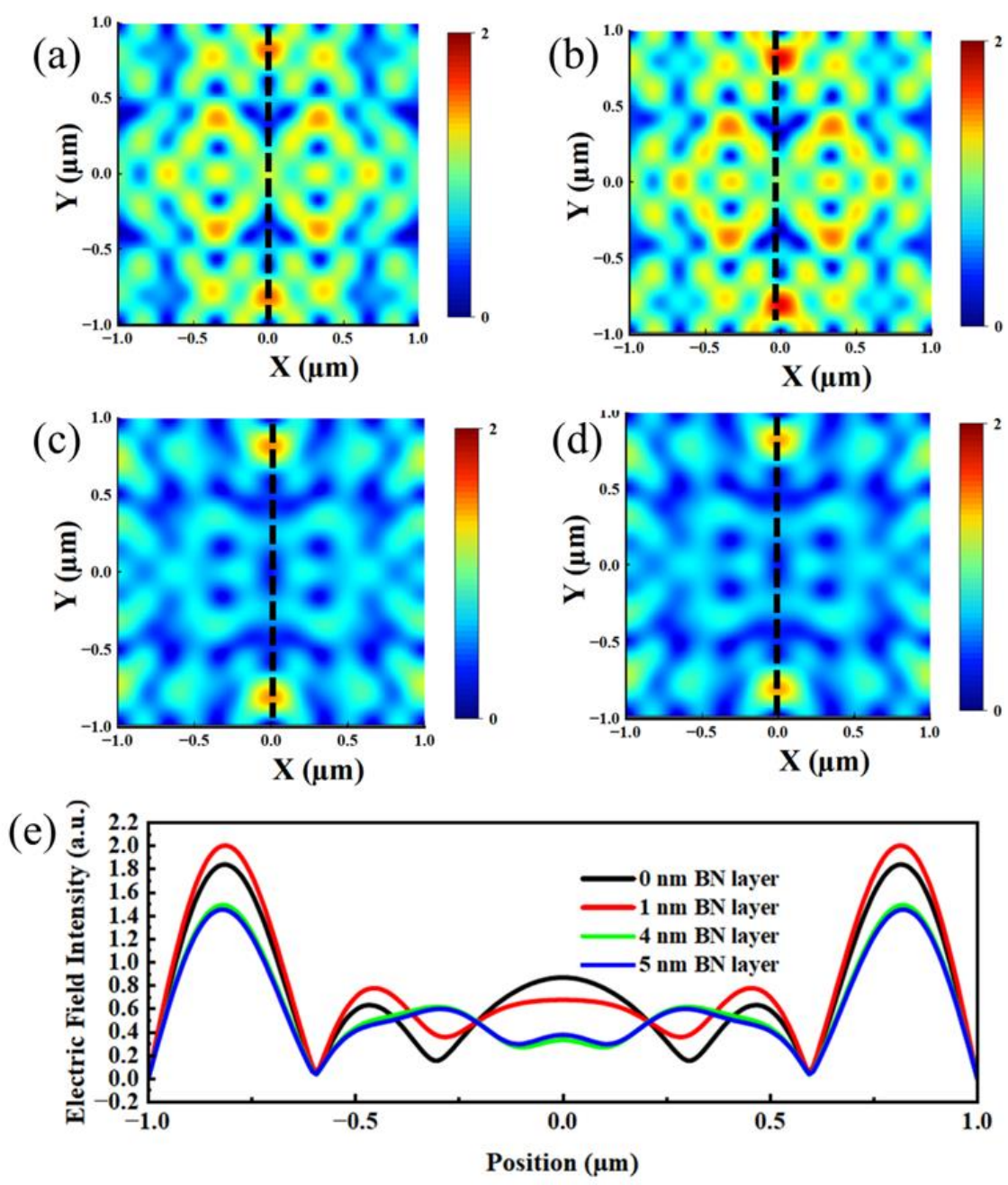

Fig. R18 Electric field distribution and analysis in the XY cross-section of X-polarized light. (a) 0 nm BN layer. (b) 1 nm BN layer. (c) 4 nm BN layer. (d) 5 nm BN layer. (e) Electric field intensity distribution at X = 0 nm.

Question 5. In the optical simulation section, the dipole source was set parallel to the X-axis, which implies that the quantum dot film is assumed to have 100% horizontally oriented

dipoles. However, the SEM images indicate that the QD film exhibits a disordered morphology, and thus this modeling assumption requires further justification. In addition, the simulated light extraction efficiency reaches as high as 42%. Achieving such a high value without incorporating any external light-extraction structures is surprising, and the manuscript currently lacks a clear mechanistic explanation for this result.

Response: Thank you for your valuable suggestions. Firstly, as stated in the response to the **General Comment**, our initial simulation only adopted dipole sources aligned along the X-axis, yielding a high LEE of 42.39%. This simulation is limited by its one-dimensional configuration, which could potentially result in an overestimation of the simulated performance. To avoid the deviation caused by one-dimensional limitations, we entrusted **Tianjin SIMUCAL Technology Co., Ltd.** to optimize the calculation logic, supplemented the **three-dimensional optical simulation** and provided corresponding formal **simulation reports (Fig. R7).** In the simulation, the polarization direction of the dipole source covers the X, Y, and Z dimensions, which is far more consistent with the actual dipole distribution characteristics under the disordered morphology of the QD films. The average LEE obtained from the simulation is 30.8%. In this work, the EQE of the BN-optimized QLED device exceeds 30%, which is higher than the theoretical calculation value obtained from the afore mentioned three-dimensional simulation. To reasonably explain this experimental result, we have verified via comprehensive literature survey that the photon recycling effect plays a key role in enhancing the external light coupling efficiency of perovskite LED devices. Therefore, in the subsequent simulation, we further incorporated the photon recycling effect into the calculation model, and the corrected LEE of device reached 35.7%. This simulated result is in excellent agreement with the experimental data obtained in this work and consistent with the relevant conclusions reported in the literature (*Adv. Mater.* 2025, 37, e06413; *Adv. Mater.* 2024, 36, 2406706).

Second, the high LEE can be attributed to two main aspects. The first point is that **the enhanced optical confinement in thinner perovskite QD films promotes horizontal dipole emission**, which increases the proportion of horizontally oriented transitions. A higher ratio of such transitions in turn leads to a greater EQE in PeLEDs. (*Nat. Nanotechnol*. 2023, 18, 981). The second one is that **the Stokes shift of perovskite materials is relatively small, enabling a photon-recycling effect within the device.** This effect allows waveguide-mode and substrate-trapped photons to regain extraction opportunities through the reabsorption-re-emission process, significantly improving the device's light-outcoupling efficiency (*Nat. Commun*. 2020, 11, 611; *Nat. Nanotechnol*. 2023, 18, 981).

Question 6. The quantum dots employed in this work are reported to have a cubic morphology. However, SEM results show that the QD film exhibits irregular morphology

with noticeable sintering, suggesting partial loss or incomplete coverage of surface ligands. Under these conditions, it remains unclear how the underlying BN layer can effectively influence the surface defects and ion migration of the overlying QDs. The manuscript currently lacks sufficient mechanistic explanation in this regard.

Response: Thank you for your valuable comments. As you pointed out, the QDs employed in this work have a regular cubic morphology, while the QD films in the SEM images display irregular agglomeration and sintering-like features, which suggests partial loss or incomplete coverage of the surface ligands of the QDs. **It should be noted that TEM and SEM have essential differences in imaging principles, signal sources, sample preparation methods, and resolution** (*Appl. Mater. Today.* 2025, 45, 102829; *Chinese Phys. B* 2024, 33 096803): TEM operates in the transmission mode, where electrons penetrate the ultrathin sample to form a two-dimensional projection, which clearly reveals the morphology, size, and structure of individual nanoparticles; In contrast, SEM employs a surface reflection/scanning mode, with signals originating only from the sample surface layer. Given the relatively large interaction volume of the electron beam, signal overlap among small-sized particles readily occurs, making it easier to observe the overall agglomerated morphology rather than the contour of individual particles. Meanwhile, TEM sample preparation requires ultrathin and well-dispersed deposition on a carbon support film, which favors the visualization of the intrinsic morphology of individual nanoparticles. By comparison, SEM samples are prepared as directly deposited films, in which nanoparticles tend to agglomeration due to high surface energy, and the observed features reflect the surface accumulation state of the film. Accordingly, TEM is more suitable for characterizing the intrinsic morphology of QDs, whereas SEM reflects the surface morphology of the entire film. **In the present work, SEM images are primarily employed to demonstrate that the QD films exhibit a more uniform and flat surface morphology after the introduction of BN.**

Regarding the mechanism by which the BN layer modulates surface defects and ion migration in the underlying QDs, the core effects can be summarized as "**interface passivation that blocks migration pathways + elevated potential barriers that suppress global ion motion**" (as addressed in Responses 1 and 2). Firstly, the introduction of BN substantially reduces the density of interface defects, and effective elimination of interface defects directly cut off the dominant pathways for ions migration from the QDs interior to the interface and across adjacent layers, reducing the driving force for the directional ions migration transport within the QDs at the source. Meanwhile, after the introduction of BN, the migration potential barrier of Br^- increased and the overall ion migration activation energy increased, indicating that BN interface modification can regulate interfacial charge

distributions, indirectly enhancing the migration resistance of charged ions within the QDs. In this way, BN inhibits ion migration throughout the entire QD layer, rather than acting only locally at the QDs/HTL interface. Moreover, BN reduces the Joule heating in QLED devices, and thermal effects are known to strongly accelerate ion migration. In QLEDs, the suppression of non-radiative recombination and Joule heating not only mitigates ion migration at the BN/QD interface but also exerts a global regulatory effect on defects and ion migration across the entire QD layer.

Therefore, both the direct interface blocking effect and the indirect whole-layer regulatory effect of BN contribute to the modulation of surface defects and ion migration in the QD layer.

Response to reviewer 3

General Comment: In the manuscript "Ultrathin nano-layered boron nitride stabilizing perovskite quantum dot-based light-emitting diodes," the authors propose ultrathin nano-layered BN as an ion-migration barrier and defect-passivation layer, reporting an EQE of 32% and a record T_{50} of 25,263 hours; while most of their responses address earlier comments, several minor revisions are still needed before publication.

Response: Thank you very much for your overall evaluation of our manuscript "Ultra-thin Nanosheet Boron Nitride-Stabilized Perovskite Quantum Dots-Based Light Emitting Diode". We are very grateful for your recognition of our core work, as well as your constructive feedback on the minor revisions. We will address all questions one by one and fully reflect the relevant revisions in the revised version.

Question 1. In planar stacks without explicit outcoupling structures, the theoretical EQE limit for isotropic emitters is ~30%. Therefore, the 42.93% light-extraction efficiency obtained from FDTD is overestimated and relies on a methodology that is not the community standard for planar devices. Please recalculate the outcoupling using the classical dipole–TMM approach with ellipsometry-measured n and k for all layers.

Response: Thank you for your meticulous questioning of the simulation method for light extraction efficiency and the theoretical limit of EQE in this study. We have verified and supplemented the opinions one by one, and the specific response is as follows:

1) Regarding the overestimation of the FDTD simulation results and the universality of the method

Firstly, we agree with your point that "the 42.93% light extraction efficiency might be overestimated". The original simulation employed only a single polarization dipole source along the X-direction, which cannot fully represent the actual emission characteristics of the isotropic emitter. we have entrusted **Tianjin SIMUCAL Technology Co., Ltd.** to optimize the calculation logic and perform **supplementary independent simulations, in which the dipole polarization directions were set along the X, Y, and Z axes of the 3D structure (Fig.R19-21)**. **The corresponding simulation reports are also provided as supporting documents.** In the simulation, we first systematically investigated the influence of the BN layer thickness on the LEE. The results revealed that both the LEE and the internal electric field intensity reached their optimal values at a BN layer thickness of 1 nm. Specifically, the LEE was determined to be 30.8% at 1 nm BN, higher than that of the device without BN (24.1%). This result indicates that the introduction of the BN layer is beneficial for enhancing the LEE. Subsequently, considering the EQE exceeds 30%, which is higher than the initially

calculated theoretical limit. To rationally account for this discrepancy, we conducted an extensive literature review and identified that the **photon recycling effect** plays a crucial role in boosting the LEE of perovskite LEDs. Therefore, we further considered the photon cycling effect in the device simulation. The results showed that the LEE of the device, when considering photon cycling, reached 35.7%, which is in good agreement with our experimental observations and previously reported literature values (*Adv. Mater.* 2025, 37, e06413*; Adv. Mater.* 2024, 36, 2406706).

The high LEE can be attributed to two main aspects. The first point is that **the enhanced optical confinement in thinner perovskite QD films promotes horizontal dipole emission**, which increases the proportion of horizontally oriented transitions. A higher ratio of such transitions in turn leads to a greater EQE in PeLEDs. (*Nat. Nanotechnol*. 2023, 18, 981). The second one is that **the Stokes shift of perovskite materials is relatively small, enabling a photon-recycling effect within the device.** This effect allows waveguide-mode and substrate-trapped photons to regain extraction opportunities through the reabsorption-re-emission process, significantly improving the device's light-outcoupling efficiency (*Nat Commun*, 2020, 11, 611; *Nat. Nanotechnol*. 2023, 18, 981).

Secondly, regarding the application of the "classical dipole-TMM method", we have actively contacted the cooperating institutions with the simulation capabilities of this software, but have not reached a cooperation agreement yet. It should be emphasized that the original choice of FDTD simulation was based on its wide industrial recognition and established authority in the calculation of optical field distributions. The simulation parameters were set according to the high-level research published in the field (such as *Adv. Mater*. 2024, 36, 2406706; *Joule*, 2021, 5, 456; *Nat Commun*. 2019, 10, 727), fully guarantees the scientific rigor and reliability of the adopted methodology.

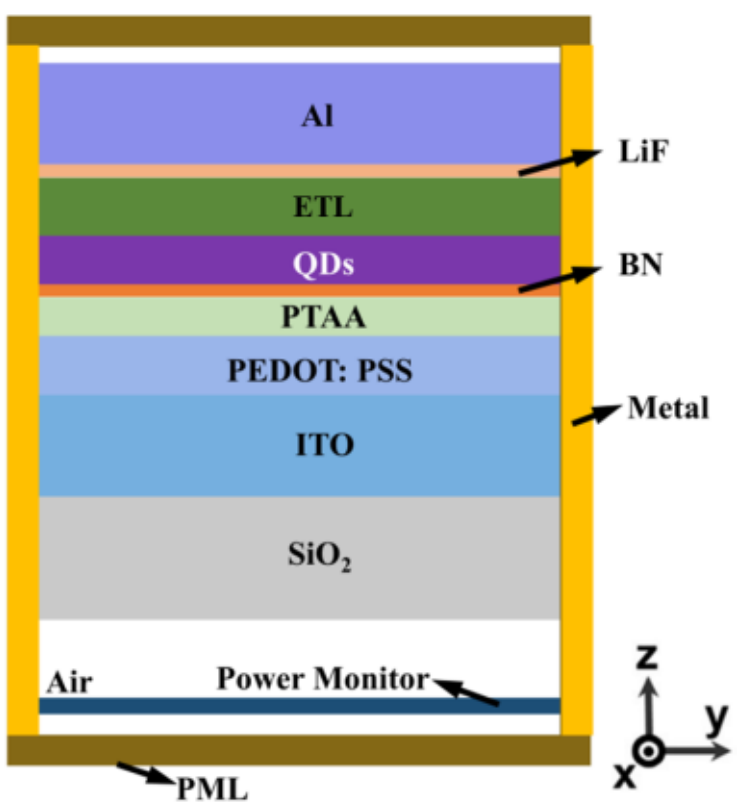

Fig. R19 Schematic diagram of the simulation model.

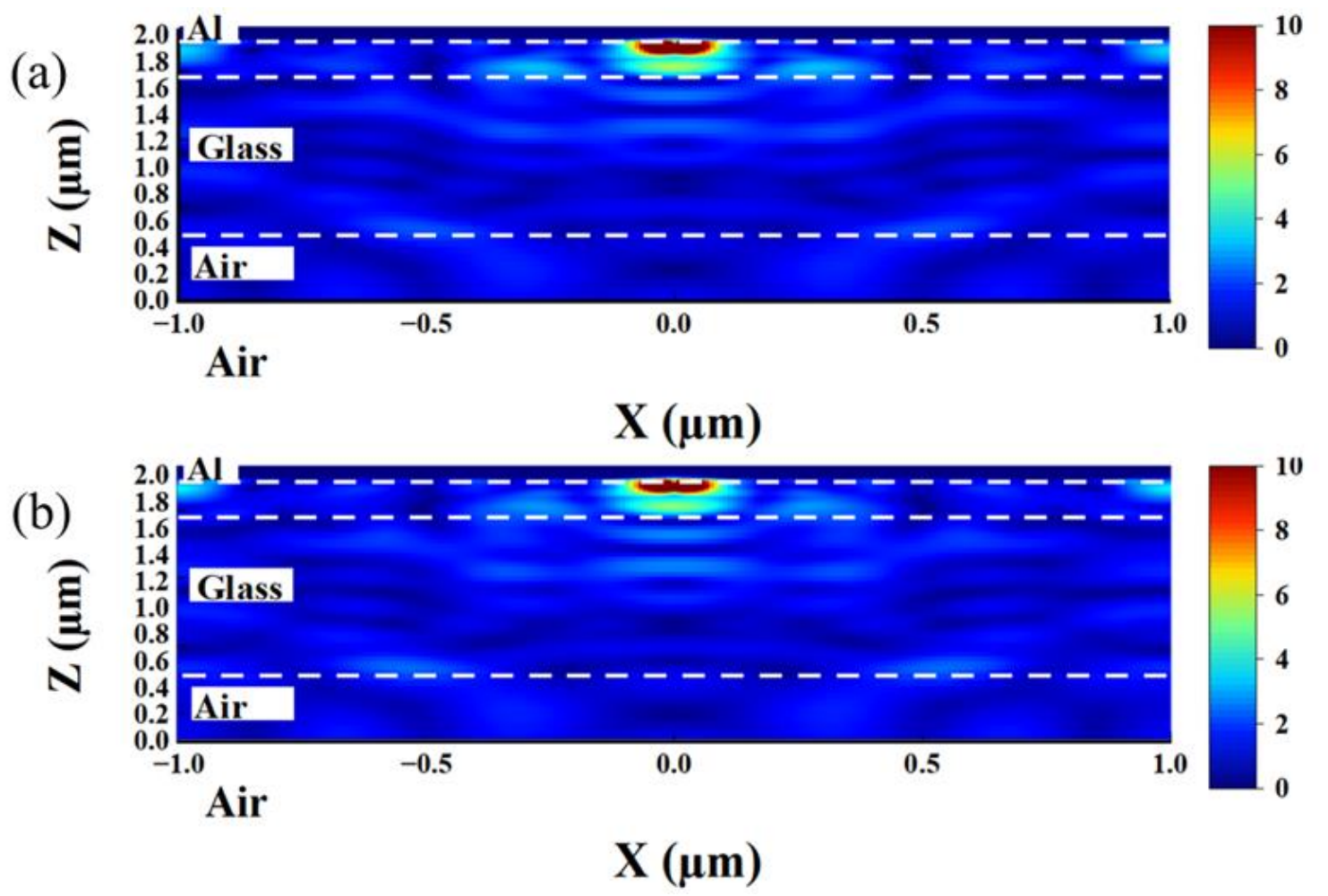

Fig. R20 Electric field distribution and analysis in the XZ cross-section of X-polarized light. (a) 0 nm BN layer. (b) 1 nm BN layer.

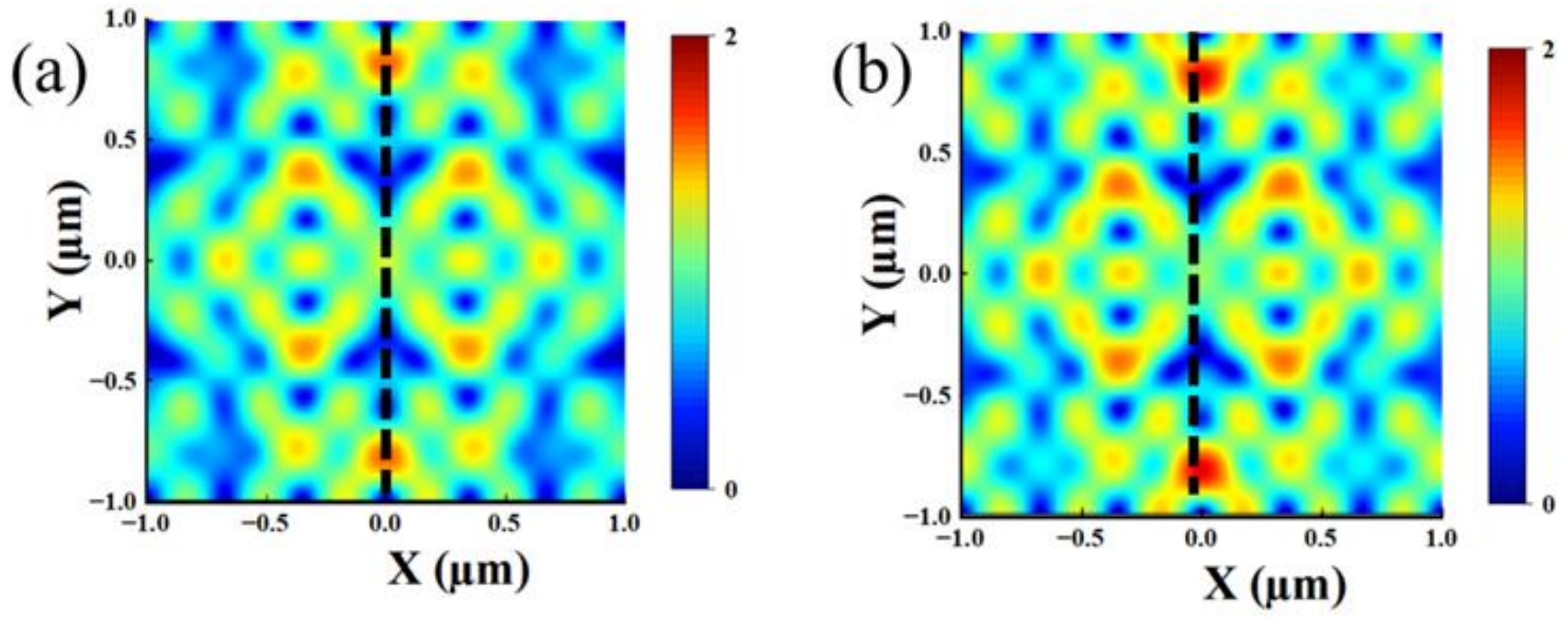

Fig. R21 Electric field distribution and analysis in the XY cross-section of X-polarized light. (a) 0 nm BN layer. (b) 1 nm BN layer.

2) Regarding the breakthrough of the EQE limit of 30% in the planar stacking structure

Regarding the viewpoint that “the theoretical EQE limit of isotropic emitters without explicit external coupling structure is approximately 30%”. As far as we know, EQE is the core parameter for evaluating the electro-optical conversion performance of LEDs. In the early development of LED technologies, such as OLED and QLED devices, the theoretical upper bound of EQE was generally considered to be around 25%. Similarly, the theoretical maximum efficiency of perovskite-based LEDs was initially estimated to be 25% as well. Nevertheless, following the first reports of room-temperature perovskite film LEDs and QLEDs (*Nat. Nanotechnol.* 2014, 9, 687; *Adv. Mater.* 2015, 27, 7162), the EQE of these devices has achieved significant breakthroughs, with values exceeding 30%. Although the intrinsic mechanism underlying the ultra-high EQE of current perovskite devices remains not fully understood, a growing number of theoretical simulations have demonstrated that the

theoretical upper limit of the EQE can be raised to approximately 40% (*Adv. Mater.* 2023, 35, 2302283*; Opt. Lett.* 2020, 45, 4786). **The exceptional EQE performance of planar perovskite-based LED may be primarily ascribed to the photon recycling (PR) enabled by a small Stokes shift (i.e., large spectral overlap between absorption and emission), which facilitates repeated cycles of re-absorption and re-emission.** In addition, Greenham et al. presented that "While an outcoupling efficiency of 100% is theoretically possible with PR", suggesting that the EQE of planar perovskite-based LED still possesses tremendous room for further improvement (*Nat. Commun.* 2020, 11, 611).

Guided by the previous reports, we conducted FDTD simulations on our device. The simulation results revealed that the introduction of BN exerted a relatively minor influence on the LEE of the device (30.8% for the QLED with BN, 24.1% for the control QLED). The exceptionally high theoretical EQE of the QLED can be ascribed to the thin QD emissive layer, which favors high EQE, together with the PR effect (*Nat. Nanotechnol* 2023, 18, 981). When we further considered the PR effect, the LEE of the BN-optimized QLED reached 35.7%, which is in excellent agreement with a previous report (*Nature* 2026, 649, 53). Therefore, the achievement of 30% EQE in our QLED is rational and well-supported by the theoretical calculations and reported results.

In real situations, our device design is expected to achieve high-performance QLEDs. Firstly, compared to pristine QDs, the BN optimal QDs present an enhanced PL properties (**a high PLQY, e.g., φ**) owing to reducing the defect density of QDs (Fig. 2g, h). Secondly, the BN layer slightly reduces the hole injection capacity (Fig. 5d). Thus, we choose TPBi-POT2T with a relatively lower mobility as the electron transport material to achieve the balance carrier transport, thereby improve the charge balance factor carrier utilization rate (γ). Furthermore, in perovskite QLEDs, an appropriate PR effect can enhance the light extraction efficiency (**a high χ**). According to the equation $\boldsymbol{\eta_{EQE} = \chi \cdot \varphi \cdot \gamma}$, our QLEDs should have a high EQE, where χ represents the light extraction efficiency, φ represents the radiative recombination efficiency, and γ represents the charge balance factor (*Light Sci. Appl.* 2021, 10, 61, *Adv. Sci.* 2023, 10, 2303192). In contrast, the control QLED would have a lower device efficiency due to QDs with a relatively lower $\boldsymbol{\varphi}$, and a small $\boldsymbol{\gamma}$ (this is because that PTAA had a higher hole mobility, resulting in a significant imbalance in carrier transport, which is crucial for device performance).

Considering that the random maximum point is difficult to rigorously characterize the

experimental results, to ensure the reliability of the data, **only in the curve description of Fig. 5f, "the champion peak EQE of 32%" was adopted, and for all other descriptions in the MS, we uniformly employed "the average peak EQE of 30.05%".**

Question 2. Given the reported film PLQY of 87% and assuming Lambertian emission, the practical upper-limit EQE is only about 26%, which is substantially lower than the claimed 32%. The EL intensity differences in Fig. R14 suggest that the BN layer introduces measurable scattering that could enhance light extraction. If h-BN indeed improves outcoupling via scattering, the angular profile should become super-Lambertian (with stronger forward emission), rather than remain strictly Lambertian. However, the authors still report Lambertian emission, which would mask any outcoupling enhancement due to h-BN. Consequently, the origin of the high EQE value (32%) remains unclear and should not be reported without further clarification.

Response: Thank you very much for your reminder and insightful thoughts. To investigate whether BN can enhance the LEE of the device, we systematically studied the influence of BN layer thickness on LEE. The simulation results showed that when the BN layer thickness was 1 nm, both the LEE and the electric field intensity of the device reached the optimal values. The LEE of the device with a 1-nm-thick BN layer increased to 30.8%, which was higher than that of the device without BN (24.1%). This result confirms that the introduction of BN effectively improves the LEE of the device. Further, **BN enhances LEE by optimizing the total optical cavity thickness and facilitating light outcoupling. Accordingly, the introduction of BN did not significantly alter the Lambertian emission characteristics of our QLED**.

To elucidate the high EQE achieved in our study, we extensively reviewed the relevant literature and found that the photon cycling effect plays a crucial role in enhancing the LEE of perovskite LED devices. Therefore, we further incorporated the photon recycling effect into our device simulations. The results revealed that the LEE of the device reached 35.7% when the photon recycling effect was considered.

Furthermore, based on our GIWAXS measurements, the introduction of BN is found to promote the orientation arrangement of QDs (Fig. R22). Consequently, the perovskite films composed of these small-sized QDs exhibit a stronger preference for horizontal dipole emission, leading to an increased fraction of horizontal transition dipole moments (TDMs) (*Nat. Nanotechnol.* 2023, 18, 981; *Adv. Sci.* 2021, 8, 2003559).

From the above, it is reasonable for us to achieve a high EQE result of 32%.

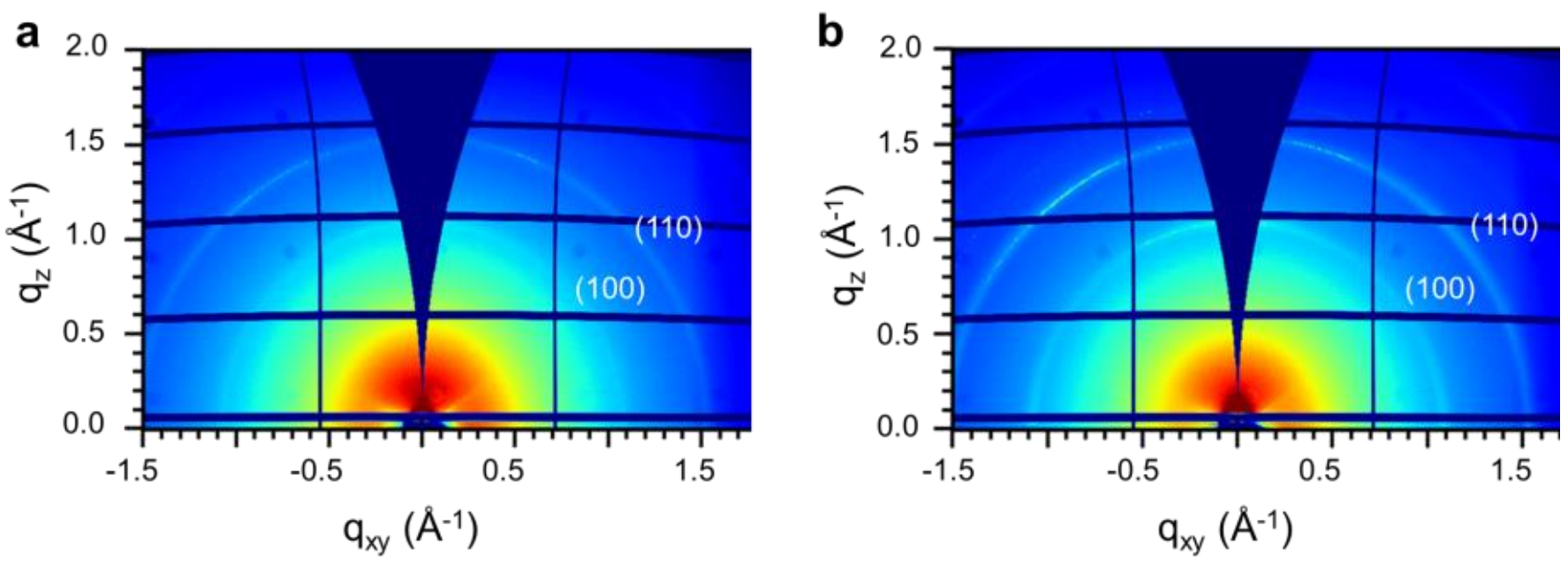

Fig. R22 GIWAXS 2D patterns of the control (a) and BN-optimized QD films (b).

Question 3. The evidence for improved heat dissipation remains inconclusive. Comparing device temperature at the same luminance is not appropriate, because the control device requires a higher voltage to reach 10,000 nits. A fair comparison of Joule heating should operate both devices at the same electrical power density (J*V).

Response: We are extremely grateful for your valuable suggestions. In our previous response to your comments, we used the method of comparing the device temperatures at the same luminance to prove the heat dissipation effect of BN. After your correction, we realized that this method might lead to inaccurate conclusions. You proposed that two devices should operate under the same electrical power density for a fair comparison of Joule heating. This suggestion is very professional and reasonable.

Therefore, we conducted a new experiment. Under the same electric power density conditions, the control device was set at 3.2V, and the electric power density was 35.3 W/cm^2 at this time; the BN optimized device was set at 4.0V, and the electric power density was 35.8 W/cm^2. After allowing both devices to operate for 60 s separately, the temperatures of the QLEDs before and after introducing BN were tested. The test results are shown in Fig. R23.

The test data clearly show that even when the two devices operate **under the same electrical power density, the temperature of the BN-optimized QLED (46.9 °C) is still lower than that of the control device (53.4 °C).** This result further confirms that the BN has the characteristics of reducing joule heat generation and enhancing heat dissipation performance.

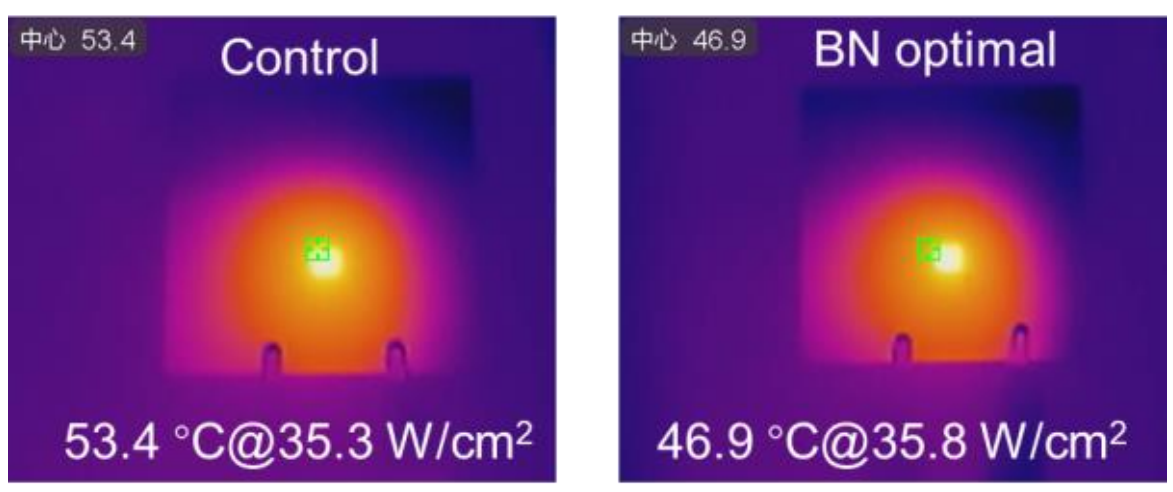

Fig. R23 Infrared thermal imaging images of control and BN optimized devices after operation at ~35 W/cm^2 for 60 s.

Response to reviewer 1

General Comment: The authors have revised the paper according to the comments raised in the previous round of review. The quality of the paper has been improved. However, some important issues remain.

Response: We sincerely appreciate for your positive evaluation on our modification, and your questions and suggestions significantly help us further improve this work and polish the content rationally. We will fully address all the remaining issues and revise the manuscript strictly in accordance with your requirements one by one.

Question 1. The authors have tested the device EQE and lifetime in another laboratory. But the measurement results (plots) are not included in the revised paper. Include the actual plots and specify the name of the third-party lab in the figures.

Response: We fully agree with your opinion and have added the device EQE and lifetime data in the revised version. The EQE-current density curve and the operation lifetime under an initial luminance of 2000 cd/m^2 of the BN optimized QLED were measured by a third-party laboratory, and the specific details of the modifications are as follows:

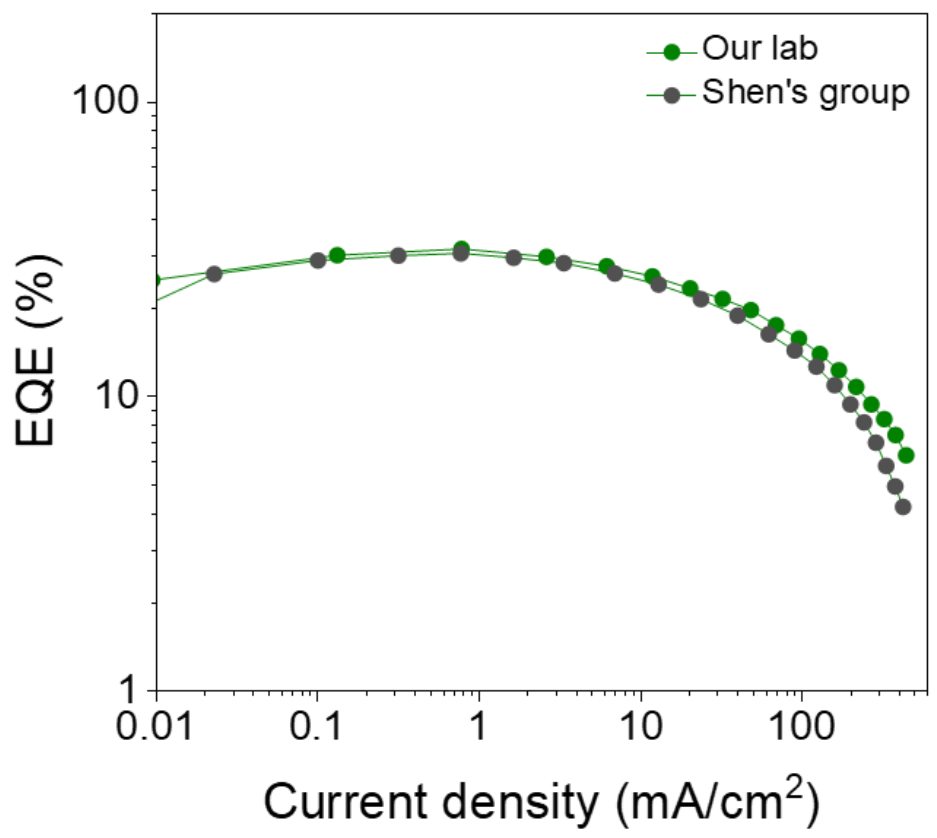

Supplementary. Fig. 30 EQE data verified by external laboratory measurements. The verified peak EQE of the BN-optimized device is 30.8%. This verification was conducted by the research group of Prof. Shen Huibin, Henan University, employing an Ocean Optics USB2000 spectrometer and a Keithley 2400 source meter.

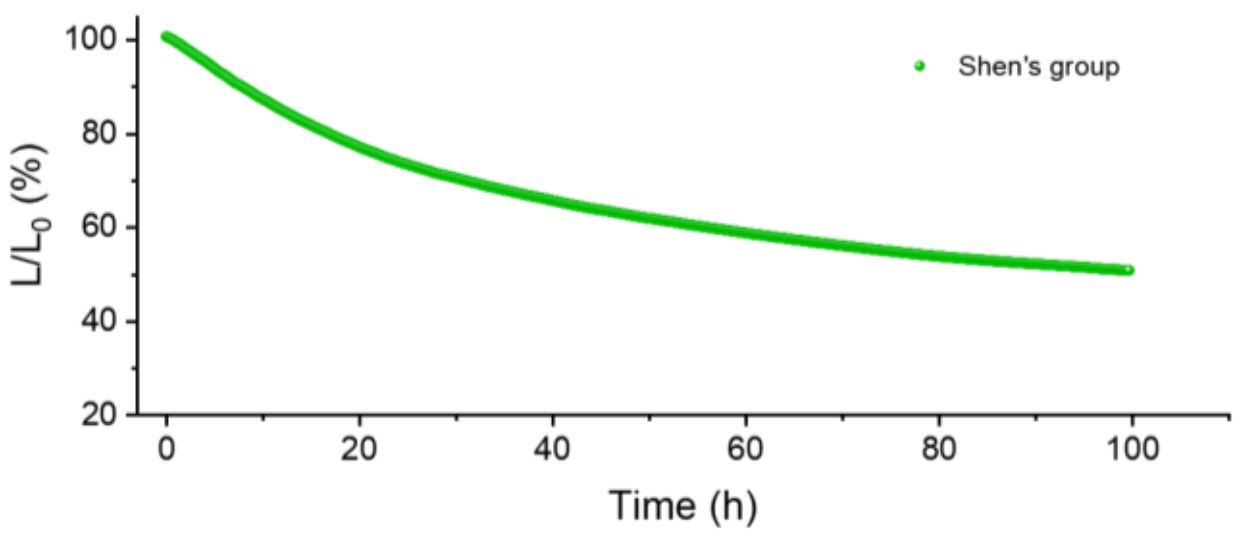

Supplementary. Fig. 39 The operational lifetime of BN-optimized QLEDs from external

laboratory measurements. The stability is about 99.7 hours at L_0 of 2000 cd/m^2. This verification was conducted by the research group of Prof. Shen Huibin, Henan University, employing a QD-LED Aging Life Test System (D3000-adv).

Question 2. The maximum efficiency measured in the third-party lab was 30.8%. But there is no mention of this result in the paper.

Response: We sincerely apologize for this omission. We have added the maximum efficiency value of 30.8% tested by the third-party laboratory into the relevant content of the manuscript for clear presentation.

Change made in the main text (Page 10)

After introducing BN, the QLEDs exhibited the champion peak EQE of 32% (**verified EQE of 30.8% by other laboratory**), which is 1.7-fold higher than that of the control device (18.5%) (Fig. 5f, Supplementary Fig. 29 and Supplementary Fig. 30).

Change made in the main text (Page 11)

the BN-optimized QLEDs exhibit a significant improvement in operational stability, with a stability of up to 102 hours at L_0 of 2000 cd/m^2 (**verified T_{50} of 99.7 hours at L_0 of 2000 cd/m^2 by other laboratory**) (Fig. 5g and Supplementary Fig. 39).

Question 3. The initial L/L_0 was labelled as 100 in the figures. How could this be true? Do you mean 100% or 1?

Response: Thank you for pointing out this question. The value of 100 for the initial L/L_0 in the figures denotes 100%, which was an inappropriate notation in the original manuscript. We sincerely apologize for this oversight and have revised all relevant content in Fig. 5g accordingly.

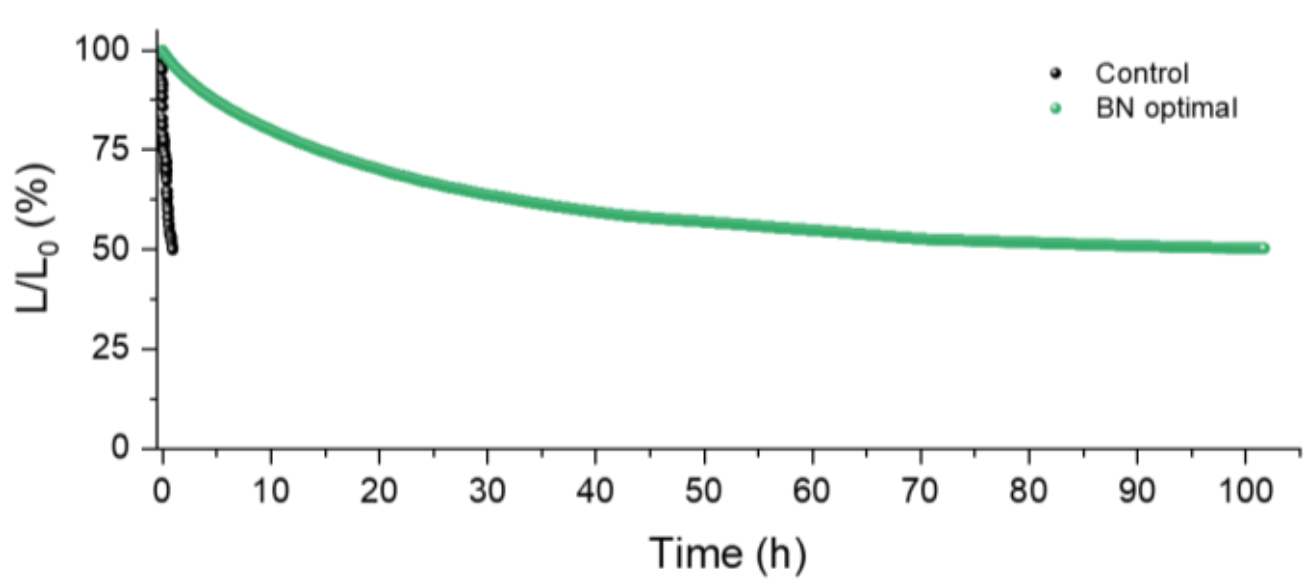

Fig. 5g The operation lifetime of the control and BN-optimized QLEDs under an initial luminance of 2000 cd/m^2.

Response to reviewer 3

General Comment: The authors revised the manuscript well based on the reviewer's comments, however, there remain insufficient details regarding the FDTD simulation of light extraction efficiency. Please provide references from high-impact journals that use FDTD to calculate outcoupling efficiency in PeLED/QLED/OLED devices, and explain how the present simulation methodology aligns with established practices. The authors should also provide the fractional distribution among all relevant optical loss channels, including air mode, substrate mode, waveguide mode, and SPP loss, to allow proper assessment of the LEE calculation. The full n, k spectra for all layers used in the simulation must be provided. In particular, the reviewer is concerned that the absorption from ITO and PEDOT:PSS is not considered, both of which have absorption at the emission wavelength. The reviewer is also concerned about the use of metal boundary conditions on the lateral walls. With the current metal sidewalls, the losses through substrate mode and waveguide mode might not be accurately estimated. Additionally, the SiO_2 layer is modeled as 1200 nm, which is far thinner than a real glass substrate. The authors should clarify whether this model is suitable for extracting the substrate mode loss. The reviewer still highly recommends using classical TMM simulation instead of FDTD.

Response: We sincerely thank the reviewers for the detailed review and constructive comments on the manuscript. The suggestions regarding the FDTD simulation method, optical loss channel analysis, and material optical constants are extremely valuable and will help us further improve the manuscript. Details of the reply are as follows:

(1) Regarding the FDTD simulation of light extraction efficiency from high-impact journals. Current research reveals that the finite-difference time-domain (FDTD) method has experienced rapid development in recent years and has now become a mature, and widely accepted mainstream numerical simulation approach for modeling light-extraction efficiency in perovskite light-emitting diodes (PeLEDs). Some representative studies published in top-tier journals rely on FDTD to analyze the evolution of internal optical fields within devices and quantitatively calculate light output efficiency.

In the field of PeLEDs, Wang Jianpu et al. employed FDTD simulations to investigate the optical coupling and emission behavior of $FAPbI_3$-based tandem devices, achieving a simulated theoretical light-extraction efficiency of 46.1%, which closely matches the experimentally measured EQE of 45.5% (*Nature* 2026, 649, 53). Yip Hin-Lap et al. used FDTD to systematically analyze the distribution patterns of optical modes inside PeLEDs, with the simulated trends in optical gain perfectly aligning with experimental EQE evolution (*Joule* 2021, 5, 456). Fan Zhiyong et al. successively applied FDTD to simulate two typical systems: first, a quantitative evaluation of light-extraction coefficients under different device

configurations (*Nat Commun* 2019, 10, 727); second, calculation of light-outcoupling efficiency in perovskite quantum wire array PeLEDs (*Nat Commun* 2025, 16, 6053).

In addition, in the field of QLED or OLED, FDTD simulation are also used to evaluate the light output performance of the devices (*Nature* 2020, 585, 379; *Light Sci Appl* 2021, 10, 180).

To sum up, these authoritative studies fully demonstrate that FDTD can accurately characterize photon transport dynamics and light-extraction characteristics within optoelectronic devices, with simulation results showing excellent consistency with experimental data, strongly validating the scientific soundness and reliability of this simulation methodology.

(2) Regarding the fractional distribution among all relevant optical loss channels. In the revised version, we have fully supplemented the energy distribution ratios for all optical loss channels, including substrate modes, waveguide modes, SPP losses, and external coupling modes, clearly illustrating the distribution patterns of each type of optical loss (Fig. R1). When the BN thickness is 1 nm, the substrate mode, waveguide mode, SPP mode, and out-coupled mode consume approximately 16.6%, 43.37%, 14.8%, and 25% of photons, respectively. **The theoretical outcoupling efficiency calculated from this mode decomposition reaches 35%, which shows great consistency with our experimental measurements (EQE=30.05%). The corresponding results of simulation and experiment have been supplemented in the revised manuscript (Supplementary Fig. 35).**

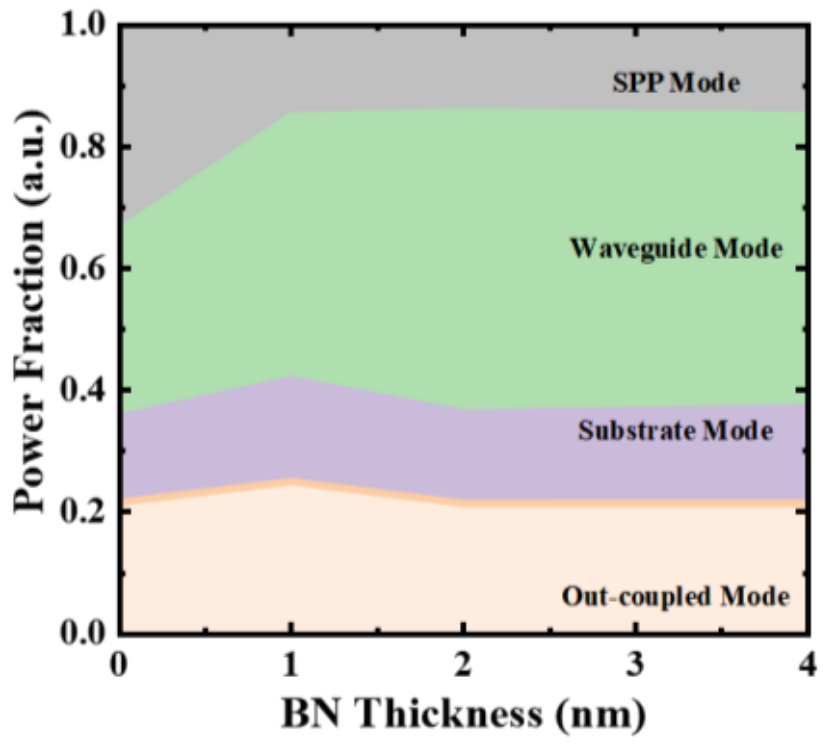

Fig. R1 Power fractions of different optical modes in our perovskite QLEDs.

(3) Regarding the *n*, *k* spectra for all layers. **We supplemented the complete *n* and *k* spectral parameters of all functional layers in Fig. R2 and Table 1**. The calculated n values only show slight deviations from the data adopted in our original simulations. Meanwhile, the weak absorption of each material at the emission wavelength was fully considered and reintroduced into the FDTD calculations, making the statistical optical loss

data more consistent with the actual operating conditions of the device. Notably, although ITO and PEDOT:PSS possess inherent light absorption, their *k* values at the device emission wavelength of 520 nm are extremely small, exerting negligible influence on the overall LEE. **After recalculation with updated parameters, the light outcoupling efficiency of BN-optimized QLED is also significantly higher than that of the reference devices without BN layers, which is consistent with our experimental observations** (Fig. R3)**.** All data has been updated and supplemented in the revised manuscript (Supplementary Fig. 33, Supplementary Fig. 34 and Supplementary Table.2).

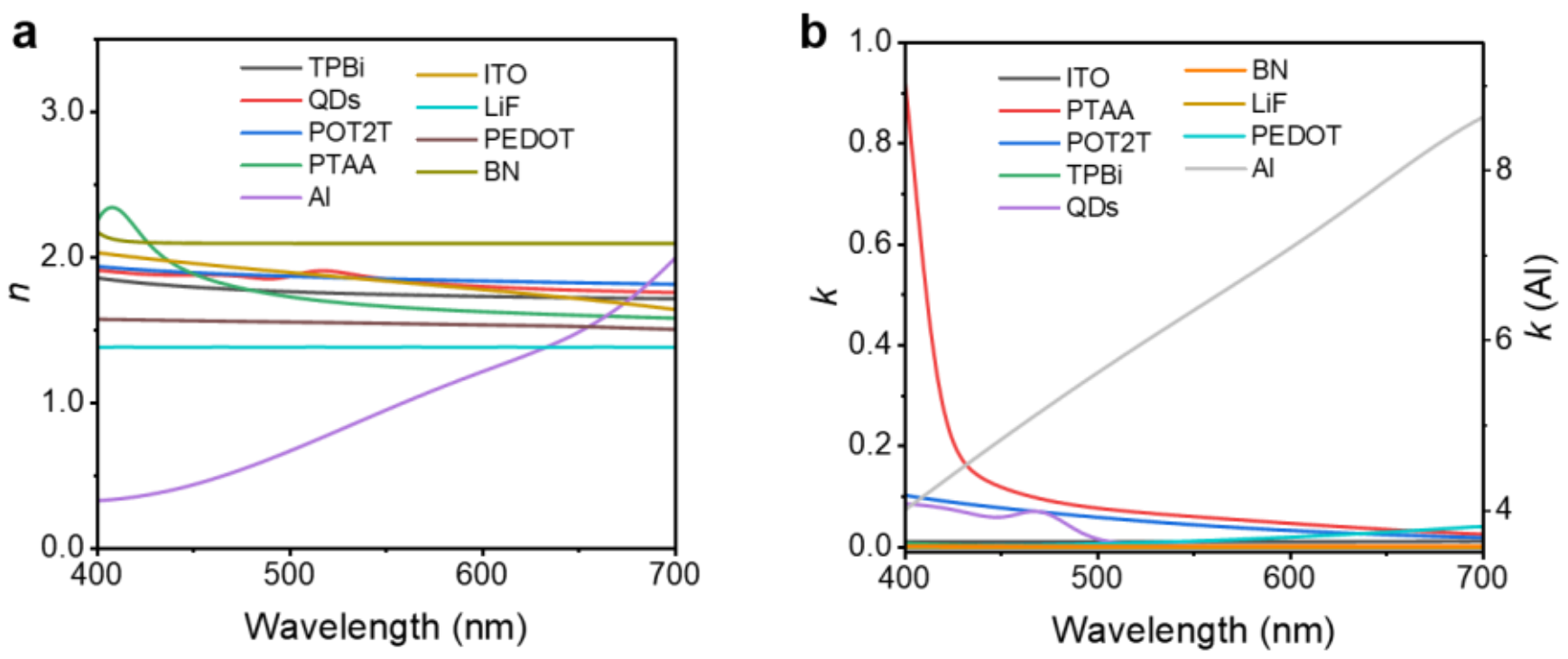

Fig. R2 Refractive indices (*n*) and extinction coefficients (*k*) of all layers in our perovskite QLEDs. The *n* values in (a) and *k* values in (b) are all extracted by ellipsometer.

Table R1. Refractive indices (*n*) and extinction coefficients (*k*) of all layers in perovskite QLEDs.

	ITO	PEDOT:PSS	PTAA	BN	QDs	TPBi	POT2T	LiF	Al
n	1.9	1.56	1.69	2.13	1.9	1.76	1.86	1.39	0.78
k	0.007	0.01	0.05	0.01	0.058	0.0005	0.05	0	7

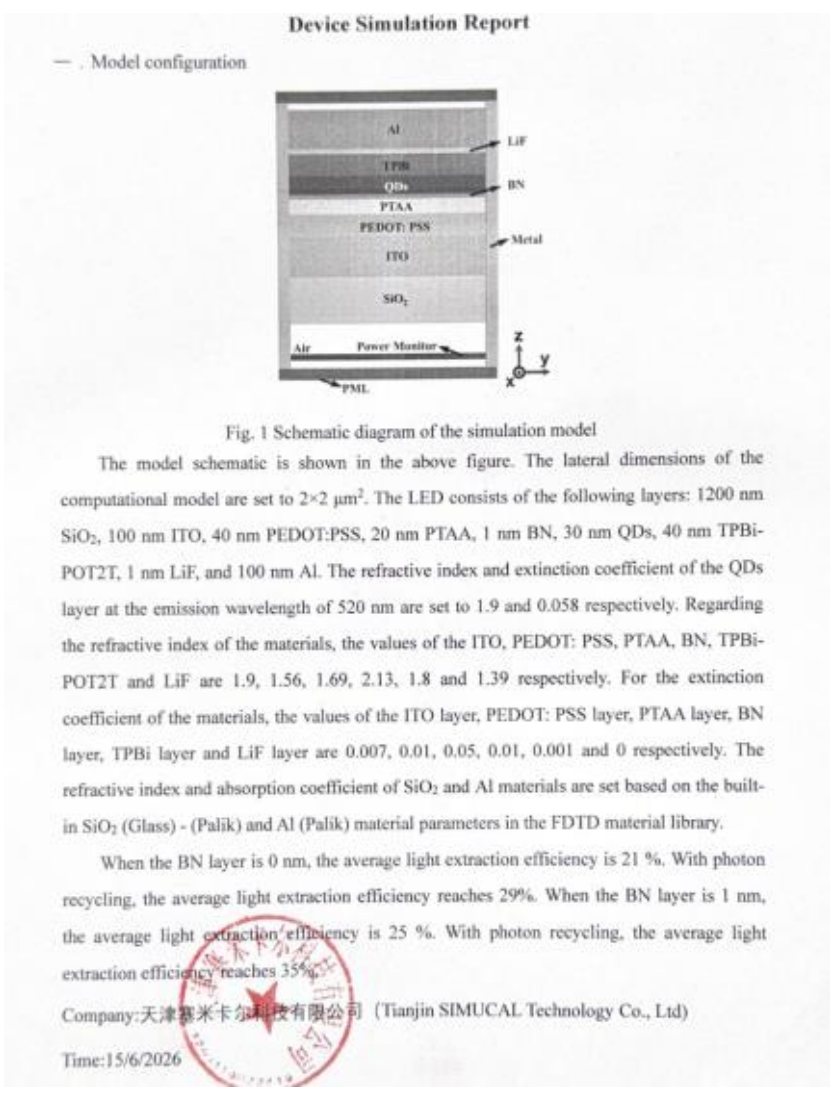

Device Simulation Report

一 . Model configuration

Fig. 1 Schematic diagram of the simulation model

The model schematic is shown in the above figure. The lateral dimensions of the computational model are set to 2×2 μm². The LED consists of the following layers: 1200 nm SiO_2, 100 nm ITO, 40 nm PEDOT:PSS, 20 nm PTAA, 1 nm BN, 30 nm QDs, 40 nm TPBi-POT2T, 1 nm LiF, and 100 nm Al. The refractive index and extinction coefficient of the QDs layer at the emission wavelength of 520 nm are set to 1.9 and 0.058 respectively. Regarding the refractive index of the materials, the values of the ITO, PEDOT: PSS, PTAA, BN, TPBi-POT2T and LiF are 1.9, 1.56, 1.69, 2.13, 1.8 and 1.39 respectively. For the extinction coefficient of the materials, the values of the ITO layer, PEDOT: PSS layer, PTAA layer, BN layer, TPBi layer and LiF layer are 0.007, 0.01, 0.05, 0.01, 0.001 and 0 respectively. The refractive index and absorption coefficient of SiO_2 and Al materials are set based on the built-in SiO_2 (Glass) - (Palik) and Al (Palik) material parameters in the FDTD material library.

When the BN layer is 0 nm, the average light extraction efficiency is 21 %. With photon recycling, the average light extraction efficiency reaches 29%. When the BN layer is 1 nm, the average light extraction efficiency is 25 %. With photon recycling, the average light extraction efficiency reaches 35%.

Company:天津赛米卡尔科技有限公司（Tianjin SIMUCAL Technology Co., Ltd)

Time:15/6/2026

Fig. R3 The simulation results from Tianjin SIMUCAL Technology Co., Ltd..

Change made in the main text (Page 16)

Optical Simulation

Finite-Difference Time-Domain (FDTD) simulation is conducted using the Lumerical FDTD software package[54-56]. The boundary conditions for the four lateral boundaries are assumed to be perfect mirrors, enabling the limited lateral dimension to be treated as infinite. The top and bottom boundary conditions are set as perfect-matched-layer (PML), which absorbs the incident electromagnetic energy. The lateral dimension of the computational domain is set to 2x2 μm^2. A single dipole source with a peak emission wavelength of 520 nm is placed in the QD layer.

(4) Regarding the concern over lateral metal boundary conditions. We fully acknowledge the reviewer's concern that total-reflection metal sidewalls may bring deviations to the quantitative calculation of substrate and waveguide mode losses. We conducted convergence tests on two lateral simulation domains (2×2 μm and 3×3 μm). Slight energy redistribution between substrate and waveguide modes is observed after expanding the simulation area, which confirms that sidewall reflection introduces weak optical coupling interference under limited lateral size.

Meanwhile, two physical mechanisms jointly greatly suppress such computational errors. First, **the lateral dimension of our model reaches the micrometer scale, where lateral mode coupling and coherent interference induced by boundary reflection are significantly suppressed**. Second, **QLEDs operate as incoherent multimode light sources, which further weakens the distortion of optical mode distribution originating from coherent lateral reflection**. The combination of these two effects suppresses the systematic error originating from metal sidewalls to an extremely low level (*Nanoscale Res. Lett.* 2019, 14, 149).

More importantly, all devices (with and without BN layers) in this work adopt identical simulation domain dimensions, metal lateral boundary conditions and all other equivalent simulation parameters. **The core objective of our FDTD simulation is to compare the relative variations of light extraction efficiency before and after BN insertion.** The simulation results demonstrate that **the incorporation of BN layers drastically elevates the light outcoupling efficiency of QLEDs**. Specifically, the TEE of devices without BN and with BN is 29% and 35%, respectively (Fig. R2 and Fig. R3). **Such values are also consistent with the reported theoretical simulation results of state-of-the-art perovskite LEDs (***Adv. Mater.* 2023, 35, 2302283; *Opt. Lett.* 2020, 45, 4786). Moreover, **the trend of changes obtained from simulation is consistent with the experimental test conclusion**,

that is, **the EQE of BN optimized QLED (30.05%) is much better than that of standard QLED (17.8%)**.

(5) Regarding the query about the thickness of the SiO_2 layer. In this work, **a 1200 nm-thick SiO_2 layer is adopted as an equivalent simplified substrate instead of a full-scale millimeter-thick real glass substrate.**

The central emission wavelength of the $CsPbBr_3$ green QLED in this work is approximately 520 nm, and the adopted 1200 nm-thick SiO_2 substrate exceeds twice the emission wavelength. **The evanescent field energy of substrate modes is mainly concentrated within the micrometer region adjacent to the thin-film stack.** The energy of reflected light reaching the bottom interface of the substrate is greatly attenuated and cannot be efficiently recoupled into the upper thin-film waveguide region. **Therefore, the interference effect from the bottom substrate interface exerts negligible influence on the quantified substrate mode loss.**

Theoretically, an even thicker substrate would be required if ultra-precise absolute values of substrate loss for a single device need to be obtained. However, **our study focuses on standardized comparative analysis: all devices with and without BN layers share the identical 1200 nm equivalent substrate configuration.** The systematic error introduced by this simplified model is uniform across all sample groups and can be mutually offset, **which will not affect our key comparative conclusion regarding the light extraction regulation effect of BN.**

As elaborated in response (4), **simulation results confirm that inserting BN layers remarkably improves the LEE of QLEDs**. The LEE of the control and BN-optimized device are 29% and 35%, respectively. The values agree well with the reported theoretical LEE levels of state-of-the-art perovskite LEDs, and the efficiency improvement trend presented by simulation is also consistent with our experimental test results. The measured EQE of the control and BN-optimized devices are 17.8% and 30.05%, respectively. The mutual verification between simulation predictions and experimental data fully demonstrates that the FDTD modeling method adopted in this manuscript is rational.

(6) Finally, we sincerely appreciate the reviewer's valuable suggestion of utilizing TMM simulation. TMM is a one-dimensional analytical simulation technique that offers high computational efficiency and is well-suited for regular layered film systems. However, **it has limitations** in handling laterally inhomogeneous microstructures or arbitrary-shaped nanostructures. FDTD is compatible with arbitrary geometries, nonlinear optical effects,

coupled photon-free carrier interactions, plasmonic responses, near-field enhancement and point-source emission. **Its main drawback lies** in the demand for refined spatial and temporal grids, which leads to a sharp rise in memory consumption and computational time. **Both methods have their respective strengths and weaknesses and can be applied to optical simulations of perovskite LEDs.** As mentioned in response (1), FDTD has matured significantly over the years and is currently widely accepted for modeling light extraction efficiency in PeLEDs (*Nature* 2026, 649, 53; *Joule* 2021, 5, 456; *Nat Commun* 2025, 16, 6053). **Given that the core focus of our work is the comparative analysis of light extraction efficiency between QLEDs with and without BN layers, the results obtained from FDTD simulations are sufficient to support our key conclusion that the incorporation of BN layers significantly improves the light extraction efficiency of QLEDs.** Accordingly, the selection of FDTD for numerical simulation in this work is rational.

Response to reviewer 1

General Comment: The authors have addressed the most of the issues raised.

Response: We sincerely appreciate the reviewer's careful evaluation and positive feedback on our revised manuscript.

Question 1. The only remaining suggestion I would offer is that the text label "T_{50} = 25263 h @ 100 cd/m^2" in Fig. 5h should be removed to avoid confusion, as it is an extrapolated lifetime. Alternatively, the authors should make it very clear in the figure that this is T_{50} (extrapolated) rather than T_{50}.

Response: We have revised Fig.5h to explicitly state that this T_{50} value is calculated via data extrapolation instead of direct experimental measurement (Fig. R1).

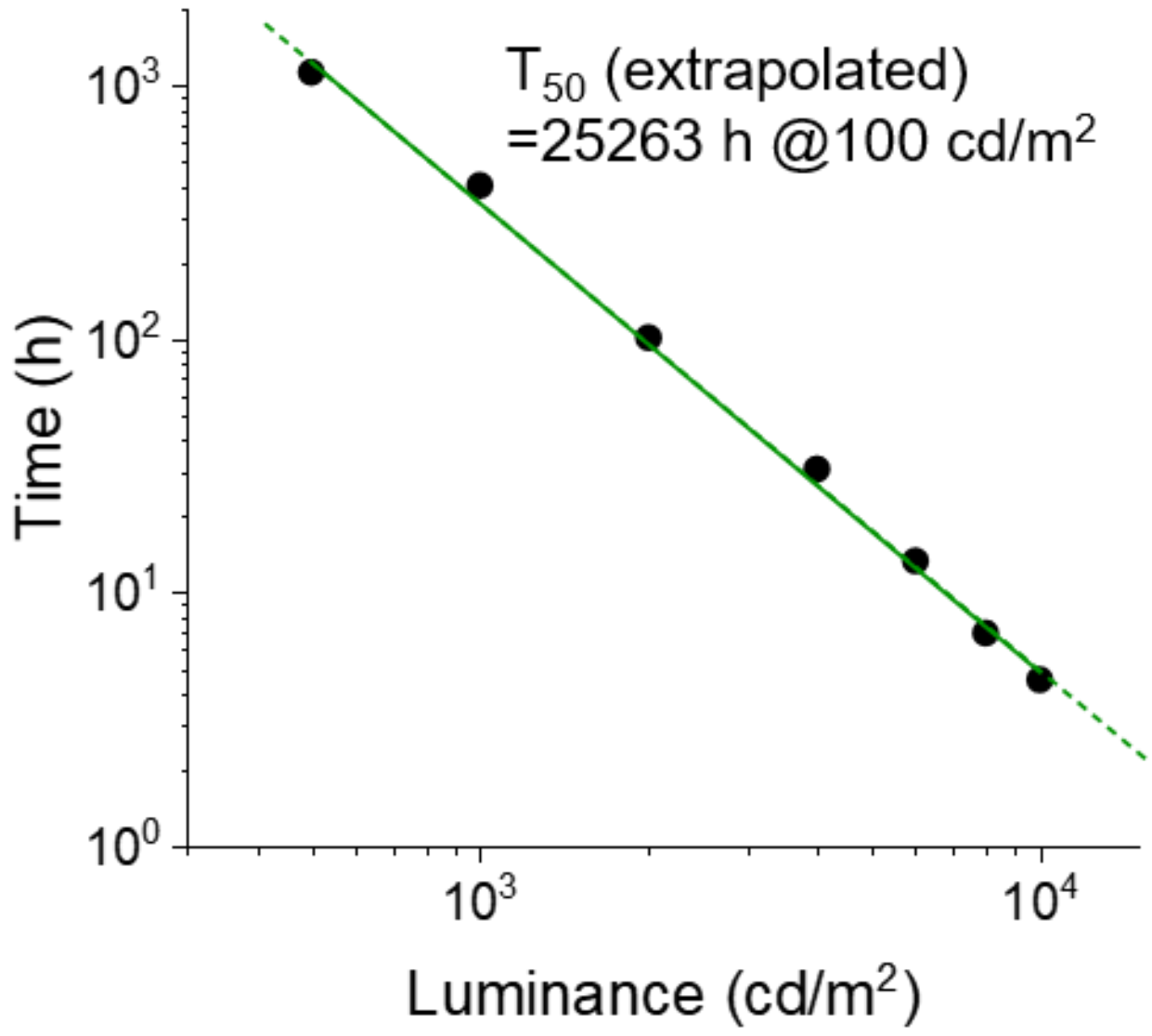

Fig. R1 The relationship between T_{50} and luminance in the accelerated lifetime experiment of BN-optimized QLEDs.

Response to reviewer 3

General Comment: The authors have adequately addressed the concern related to light outcoupling efficiency. I recommend that the manuscript be published in Nature Communications without further revision.

Response: We sincerely appreciate the reviewer's careful evaluation and positive feedback on our revised manuscript.
